# Specification, annotation, visualization and simulation of a large rule-based model for ERBB receptor signaling

**DOI:** 10.1186/1752-0509-6-107

**Published:** 2012-08-22

**Authors:** Matthew S Creamer, Edward C Stites, Meraj Aziz, James A Cahill, Chin Wee Tan, Michael E Berens, Haiyong Han, Kimberley J Bussey, Daniel D Von Hoff, William S Hlavacek, Richard G Posner

**Affiliations:** 1 Clinical Translational Research Division, Translational Genomics Research Institute, Phoenix, AZ 85004, USA; 2 Department of Biological Sciences, Northern Arizona University, Flagstaff, AZ 86011, USA; 3 Ludwig Institute for Cancer Research, Melbourne-Parkville Branch, Royal Melbourne Hospital, Parkville, Victoria, 3050, Australia; 4 Cancer and Cell Biology Division, Translational Genomics Research Institute, Phoenix, AZ 85004, USA; 5 Theoretical Biology and Biophysics Group, Theoretical Division and Center for Nonlinear Studies, Los Alamos National Laboratory, Los Alamos, NM 87545, USA; 6 Department of Biology, University of New Mexico, Albuquerque, NM 87131, USA

**Keywords:** Systems biology, Epidermal growth factor (EGF) receptor (EGFR), Rule-based modeling, Temporal phosphoproteomics

## Abstract

**Background:**

Mathematical/computational models are needed to understand cell signaling networks, which are complex. Signaling proteins contain multiple functional components and multiple sites of post-translational modification. The multiplicity of components and sites of modification ensures that interactions among signaling proteins have the potential to generate myriad protein complexes and post-translational modification states. As a result, the number of chemical species that can be populated in a cell signaling network, and hence the number of equations in an ordinary differential equation model required to capture the dynamics of these species, is prohibitively large. To overcome this problem, the rule-based modeling approach has been developed for representing interactions within signaling networks efficiently and compactly through coarse-graining of the chemical kinetics of molecular interactions.

**Results:**

Here, we provide a demonstration that the rule-based modeling approach can be used to specify and simulate a large model for ERBB receptor signaling that accounts for site-specific details of protein-protein interactions. The model is considered large because it corresponds to a reaction network containing more reactions than can be practically enumerated. The model encompasses activation of ERK and Akt, and it can be simulated using a network-free simulator, such as NFsim, to generate time courses of phosphorylation for 55 individual serine, threonine, and tyrosine residues. The model is annotated and visualized in the form of an extended contact map.

**Conclusions:**

With the development of software that implements novel computational methods for calculating the dynamics of large-scale rule-based representations of cellular signaling networks, it is now possible to build and analyze models that include a significant fraction of the protein interactions that comprise a signaling network, with incorporation of the site-specific details of the interactions. Modeling at this level of detail is important for understanding cellular signaling.

## Background

Modeling is an essential component of systems biology [1]. An important class of models is the class based on mass-action chemical kinetics. Models have the potential to elucidate the behaviors that logically follow from mechanistic knowledge and assumptions, which can often be reduced to a collection of reactions and the parameters that characterize the mass-action kinetics of these reactions [2,3]. The parameters of models for the chemical kinetics of molecular interactions can be measured independently, at least in principle, and must take on values consistent with physicochemical constraints. Models capturing mass-action chemical kinetics can be specified in various traditional forms, such as that of ordinary differential equations (ODEs). This approach has been quite useful for studying small modules at biochemical reaction resolution [4]. Coarser resolution models of larger networks have also been useful for studying systemic properties, for example, how processes such as feedback and internalization may influence receptor tyrosine kinase signaling [5,6].

Signaling proteins contain multiple functional components and multiple sites of post-translational modification. As a result the interactions among signaling proteins have the potential to generate myriad protein complexes and post-translational modification states [7,8]. This feature of cell signaling networks has been called combinatorial complexity. Because of combinatorial complexity, ODE models are poorly suited for representing the molecular interactions within a cell signaling network. The number of chemical species that can be populated in a cell signaling network, and hence the number of equations in an ODE model required to capture the dynamics of these species, is prohibitively large.

In part to deal with the issue of combinatorial complexity, the rule-based modeling approach was developed as a method for efficiently and compactly specifying the reactions that can arise from molecular interactions in signaling networks [9,10]. In a rule-based model, the structure of a reaction network is implicitly defined by rules that represent molecular interactions, whereas in a traditional model, network structure must be explicitly specified. A rule represents a class of reactions involving reactants with common components and component properties. An important simplification of the rule-based modeling approach is that all reactions within a class are assigned the same rate law. Thus, a key assumption underlying the rule-based modeling approach is that molecular interactions are modular, meaning that network dynamics are largely determined by local properties of protein components responsible for interactions. This coarse graining approach allows for more compact model specification than traditional modeling approaches. The rate law associated with a rule provides only a coarse-grained description of the kinetics of the reactions within the rule-defined reaction class. However, the coarseness of a rule can be adjusted by tuning the contextual elements of the rule. At the finest level, the contextual elements required for a reaction are highly specific and a rule defines a single unique chemical reaction. At the coarsest level, a rule indicates that a reaction center can undergo a reaction regardless of the molecular context in which that reaction center is found, and a single rule defines a set of reactions, one for each unique context in which the transformation of the rule can take place. Simulation of a rule-based model yields results consistent with principles of chemical reaction kinetics.

Although rules can be used to define large-scale biochemical reaction networks in a compact efficient manner, the shear size of such networks, has posed a formidable barrier to the development and analysis of models for signal-transduction systems that account for site-specific details of protein interactions (in terms of rules). To address this problem, we and others have developed software for simulating large-scale rule-based models. The key feature of these tools is that the computational cost is independent of the size of the reaction network implied by a set of rules [11-13]. Thus, it is now possible to consider building and analyzing rule-based models that include site-specific details about protein-protein interactions.

Here, we use the rule-based modeling approach to build a model for ERBB receptor signaling. The model includes the four members of the ERBB family of receptor tyrosine kinases, Ras, phosphoinositide 3-kinase (PI3K), and other signaling proteins that play a role in activation of extracellular signal-regulated kinase (ERK) and Akt. The model encompasses essentially the same proteins considered in the ODE model of Chen et al. [14] and it is related to a number of other ODE models reported in the literature, such as the model of Birtwistle et al. [15]. The model presented here accounts for site-specific details of molecular interactions, which would be impossible to simulate using an ODE model. A large number of models, of different types, have been reported in the literature for various aspects of ERBB receptor signaling [16-19], but the consideration of site-specific mechanistic details by modelers has so far been limited [20,21].

We apply the conventions of Chylek et al. [22] to visualize and annotate our model, and we demonstrate that the model can be simulated using recently developed software implementing a network-free simulation approach that enables the simulation of interactions marked by combinatorial complexity [13]. A key advance of the model presented here is avoidance of arbitrary simplifying assumptions about the molecular mechanisms of signaling that have the sole purpose of facilitating ODE model specification and/or simulation. The model accounts for over 50 sites of phosphorylation, which is far more than have been included in previous models of ERBB signaling. The ability to incorporate individual phosphorylation sites in a model enables mechanism-based interpretation of temporal phosphoproteomic data and provides an opportunity to use such data to identify parameter values.

We note that our report is intended as a demonstration of recently developed methodology, and does not represent an effort to gain insights into ERBB receptor signaling. Our hope is that integrated modeling and experimental efforts, focused on understanding how site-specific details impact network function, will be stimulated by the demonstrated specification, annotation, visualization and simulation capabilities. The novelty of this study lies in the demonstration of these capabilities at the scale considered. We note that demonstrating the usefulness of rule-based modeling is not a goal of the study reported here; the usefulness of this modeling approach is already established by numerous applications of the approach [23-32].

## Results

We specified a rule-based model for molecular interactions in the ERBB receptor signaling network (see Methods). The model specification, including nominal parameter values, is provided in the form of a BioNetGen input file [28], which is a plain-text file. The file comprises the “Full Model Specification” Tiddler of our TiddlyWiki, which is available online ( https://modeling.tgen.org). The BioNetGen input file, which is named “ERBB_model.bngl,” is also provided separately (Additional file 1). The collection of online materials is included in the Supplementary Material as an archive file (Additional file 2). The model is composed of 544 rules. It accounts for 18 proteins, over 30 protein domains, several linear motifs, and 56 sites of lipid and protein phosphorylation. The rules of the model represent interactions of ligands with ERBB receptors, receptor dimerization, phosphorylation-dependent interactions of adapter proteins with receptors, the MAPK cascade downstream of Ras, PI3K signaling events that regulate phosphorylation of Akt, multiple feedback loops, and phosphorylation events that regulate the above processes. Dephosphorylation reactions are included in the model, but the phosphatases that mediate these reactions (e.g., PTEN and SHP-2) are not explicitly considered.

**Figure 1:**
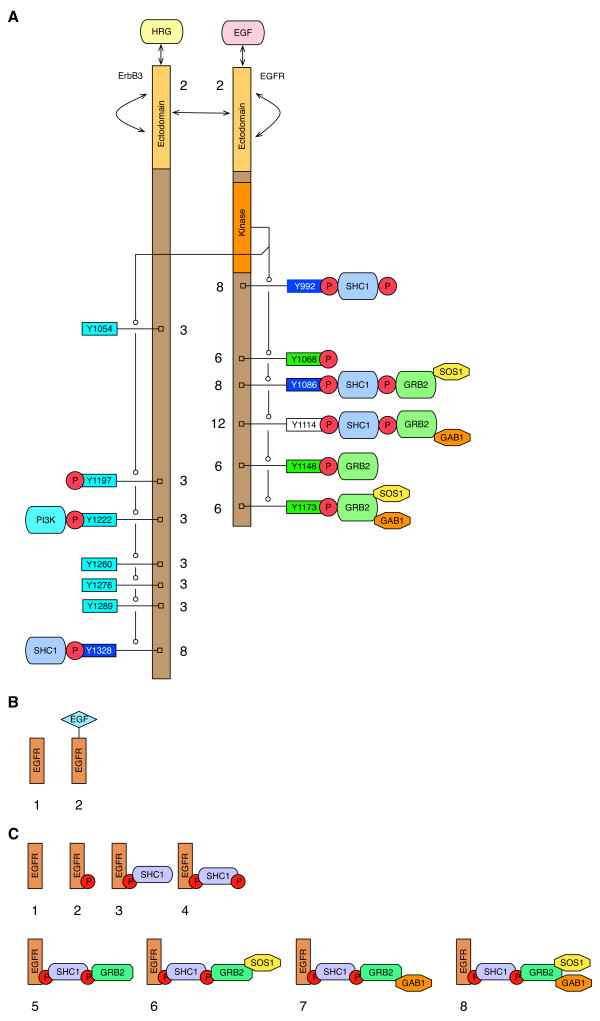
**Combinatorial complexity is a feature of ERBB receptor signaling.** (**A**) Diagram depicting a selected subset of interactions of epidermal growth factor (EGF), EGF receptor (EGFR), heregulin (HRG), ERBB3, GRB2, SOS1, GAB1, SHC1, and PI3K considered in the model. These binding partners of EGFR and ERBB3 are documented in NetPath [50] and HPRD [51]. This diagram also depicts substrates of the EGFR kinase domain (six tyrosine residues in EGFR and seven tyrosine residues in ERBB3). These sites of phosphorylation in EGFR and ERBB3 are documented in HPRD [51] and Phospho. ELM [52]. Next to each component of EGFR and ERBB3, the number of possible component states is indicated. These counts are based only on the proteins, sites of phosphorylation, and interactions depicted in this diagram. Note that additional interactions are considered in the model (cf. Figure 2). For example, in this diagram, we do not consider phosphorylation of SOS1 and GAB1. Ligand binding sites have two possible states (see below). Docking sites for SHC1 (blue rectangles) have eight possible states (see below). Docking sites for GRB2 (green rectangles) have six possible states. In the model, Y1114 in EGFR (white rectangle) is a docking site for both GRB2 and SHC1. Thus, this docking site has 12 possible states. Docking sites for PI3K (cyan rectangles) have three possible states. Based on these counts of possible component states, the number of possible states for an EGFR monomer is 2·8·6·8·12·6·6 = 331,776, and the number of possible states for an ERBB3 monomer is 2·3^6^·8 = 11,664. An EGFR: ERBB3 heterodimer has more than 3.8 × 10^9^ states, and an EGFR homodimer has more than 5.5 × 10^10^ states. When we consider the additional interactions included in the model but not shown here, we find that the number of possible states for an EGFR homodimer is much greater than a googol (10^100^). (**B**) As illustrated in this panel, the ectodomain of EGFR has two possible ligand-bound states: free or bound to EGF. (**C**) As illustrated in this panel, a docking site in EGFR for SHC1 has eight possible states: unphosphorylated, phosphorylated, bound to unphosphorylated SHC1, bound to phosphorylated SHC1, bound to SHC1 in complex with GRB2, bound to SHC1 in complex with GRB2: SOS1, bound to SHC1 in complex with GRB2: GAB1, and bound to SHC1 in complex with a ternary complex of GRB2, SOS1, and GAB1.

The model accounts *implicitly* for more chemical species (»10^100^) than there are molecules available to populate these species (Figure 1). The model is able to provide this description because of simplifying assumptions embedded in its rules [10], which derive from assumptions of modularity. We view such assumptions as reasonable because proteins are composed of modular parts [33]. The trade-off for concise model specification is coarse-graining of chemical kinetics, meaning that all reaction events implied by a rule are taken to have the kinetic rate law associated with the rule. This coarse graining is controllable, as rules can be refined as needed to capture empirical data. Indeed, the only essential difference between a rule-based model and an ODE model lies in the means of model specification; both types of models provide representations of chemical kinetics [34]. To specify an ODE model, a modeler must state which chemical species in a system are populated and how these species are connected and influence each other. In contrast, to specify a rule-based model, a modeler must state which interactions are occuring in a system and the contextual dependencies of these interactions. The latter approach is more convenient when interactions depend mostly on local properties of proteins, such as whether a site is phosphorylated and free. Rules for interactions, together with rate laws and parameter estimates, can be used to predict which of the potentially populated chemical species are populated, regardless of the number of potentially populated chemical species [11-13]. The main point of Figure 1 is to illustrate that known interactions and post-translational modifications of EGFR imply a number of potentially populated chemical species that is so large as to confound intuition and the ODE modeling approach, because the subset of populated chemical species, which is the information required to specify a mechanistic ODE model incorporating site-specific details about EGFR interactions, is impossible to identify through measurement or inference based on simple reasoning.

**Figure 2:**
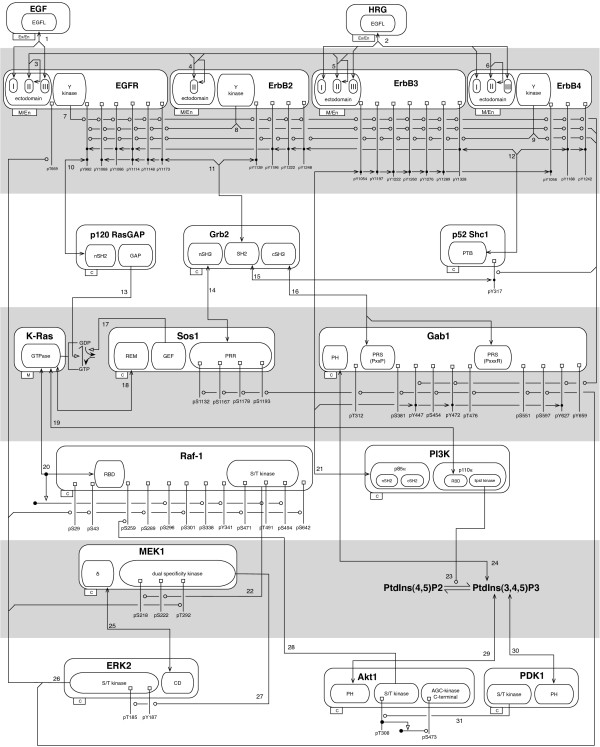
**A rule-based model for ERBB receptor signaling.** Boxes and nested boxes represent proteins and (sub)components of proteins. Only (sub)components considered in the model are shown. Boxes are decorated with post-translational modification flags, which are each labeled at one end and connected to a small box at the other. The prefix of the label indicates the modification (i.e., ‘p’ represents addition of a phosphate group), and the rest of the label indicates the site of modification. Compartmental locations of proteins are indicated in tabs at the lower left corners of boxes: M, membrane; C, cytoplasmic; Ex, extracellular; and En, endosomal. A line that begins and ends with an arrowhead represents a direct binding interaction. A line with a circle at one end identifies an enzyme-substrate relationship; the circle identifies the substrate. A line that ends with an open, triangular arrowhead represents activation. Numbers next to arrows refer to sets of rules, which are identified in an accompanying model guide. See Methods for additional details.

A challenge of developing a large model is communicating the substance of the model in such a way that it can be understood. In Figure 2, we present an extended contact map [22], which shows the proteins, protein components, and sites of phosphorylation as well as the direct interactions and enzyme-substrate relationships considered in the model. Proteins are represented as boxes and arranged in layers to suggest the causality of signaling events, with the top layer corresponding to ligands, the layer below corresponding to ERBB receptors, etc. Most of the 544 rules of the model can be mapped to one of the 31 interactions represented by arrows in Figure 2. The rules corresponding to a given arrow represent a common interaction but in different contexts. The correspondence between rules and arrows is indicated in a model guide [22], which is described below.

Making a large model reusable and extensible requires not only a means to understandably visualize the model but also annotation so that the basis of the model can be evaluated and updated as new knowledge is generated. To annotate our model, we prepared a model guide [22] (see Methods and Additional Materials). The guide links formal elements of the model (viz. graphs used to represent proteins and their component parts) to information about these components available in online resources, such as UniProt [35], OMIM ( http://omim.org), and Pfam [36]. This ability to easily connect formal model elements to information available in online resources, including sequences, is one of the advantageous and innovative features of rule-based modeling. For each protein included in the model, the guide includes a brief summary of available knowledge that was considered in the formulation of the model. Finally, as mentioned above, the guide links the arrows of Figure 2 to specific rules.

The parameters of our model, rate constants and protein copy numbers, are largely unknown. Identifying the values of these parameters to obtain a validated, predictive model would be a formidable challenge. Here, our intention is simply to demonstrate the feasibility of specifying, visualizing, annotating and simulating a model that captures the site-specific details of protein-protein interactions in a signaling network. Such a model can make predictions about time courses of phosphorylation for individual serine, threonine, and tyrosine (S/T/Y) residues, which is essential for mechanism-based interpretation of multiplex temporal phosphoproteomic data [37]. For the purpose of demonstrating that the model can be simulated, we divided the model parameters into several classes and estimated a range of feasible values for each class (Table 1). We then sampled within these ranges to randomly specify nominal parameter values (see Methods).

**Table 1:** Ranges considered for six classes of model parameters

**Parameter class**	**Estimated range**	**Units**
Rate constant for a bimolecular association reaction	10^-7^ – 10^-5^	(molecules/cell)^-1^·s^-1^
Rate constant for a unimolecular dissociation reaction	10^-2^ – 10^0^	s^-1^
Rate constant for a phosphatase-catalyzed reaction*	10^-3^ – 10^-1^	s^-1^
Rate constant for a receptor trafficking step (internalization or recycling)*	10^-3^ – 10^-1^	s^-1^
Rate constant for endocytic degradation*	10^-3^ – 10^-1^	s^-1^
Protein copy number	10^4^ – 10^6^	molecules/cell

Processes characterized by parameter classes marked by asterisks are taken to be first-order processes.

**Figure 3:**
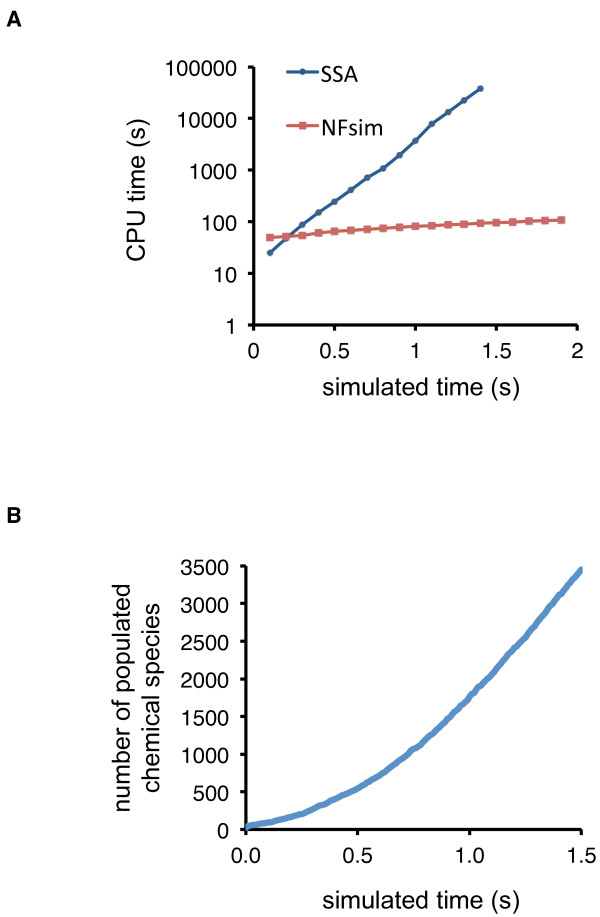
**System size and simulation performance. **(**A**) Cost of network-free simulation vs. cost of on-the-fly simulation. The CPU time required to perform the simulation specified in the BioNetGen input file of Supplemental Archive File 1, but without equilibration, was determined for the on-the-fly stochastic simulation algorithm (SSA) implemented in BioNetGen and also for the network-free stochastic simulation algorithm implemented in NFsim. Equilibration was not performed so that the initial condition would encompass a minimal number of populated species. Thus, in these simulations, all proteins were free and unphosphorylated at time *t* = 0. As can be seen, the computational cost for on-the-fly simulation increases exponentially as a function of time, whereas the computational cost of network-free simulation increases linearly as a function of time. There are no data points for t ≥ 1.5 s for the on-the-fly algorithm because the cost of network generation made simulating the model beyond this time impractical. (**B**) On-the-fly stochastic simulation of the model with BioNetGen (see Methods). The simulation results demonstrate that a large number of chemical species are populated in the ERBB receptor signaling network.

As stated above, the sheer size of the network captured (implicitly) in our model (in excess of 10^100^ reachable chemical species) has posed a barrier to simulation using conventional methods. Obviously if the cost of simulation scales with network size, then simulation of such large-scale reaction networks becomes impractical. On-the-fly stochastic simulations algorithms are an alternative to numerical integration of ODEs [38,39] but on-the-fly simulation also becomes prohibitively slow as the number of populated states increases and the number of reachable states explodes [40]. The CPU time required for simulation of the model using such a method increases exponentially as the number of reactions in a network grows (exponentially). In contrast, network-free simulation methods [11,12,40,41] have a constant cost of simulation per reaction event and hence the CPU time increases linearly with the number of reaction events in a system (Figure 3A).

Figure 3B illustrates that in our model a large number of chemical species quickly become populated after initiation of ERBB receptor signaling. Within 1 second after initiation of signaling, over 1,500 chemical species are populated. This number of species exceeds the number that can be practically considered in a manually specified ODE model in which one equation would be required for each reachable species. The results of Figure 3B suggest that dispersion of mass into a large number of chemically distinct states is an inherent feature of cell signaling networks and explains why the on-the-fly method becomes impractical (Figure 3A). It should be noted that the simulations of Figure 3 are not physiological, as the initial condition is artificial. The point of these simulations is simply to demonstrate that interactions of signaling proteins can be expected to lead to the population of more chemical species that can be practically tracked in an ODE model.

**Figure 4:**
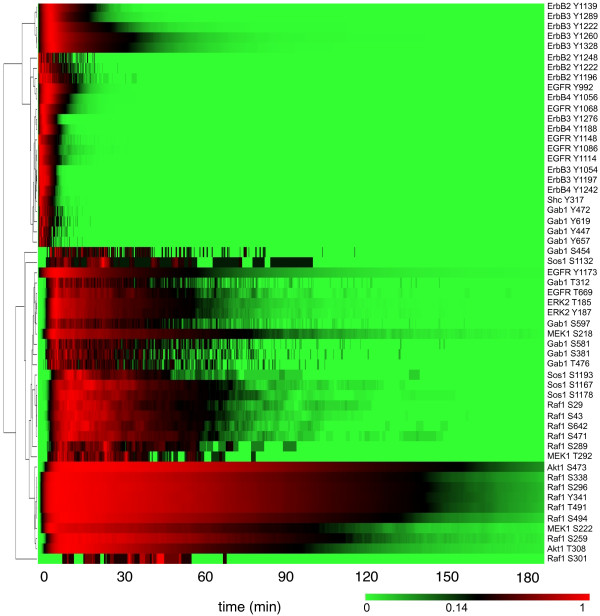
**Simulation of ERBB receptor signaling in response to addition of epidermal growth factor and heregulin.** Network-free stochastic simulation of the rule-based model illustrated in Figure 2 with NFsim (see Methods). The simulation results demonstrate the capability of the rule-based modeling approach to represent site-specific phosphorylation kinetics. For each time course, phosphorylation level is normalized to the maximum level. Time courses are clustered by similarity (see Methods).

To simulate the model we use NFsim [13], which implements a network-free simulation algorithm [40]. The simulation results are shown in Figure 4. The heat map of Figure 4 reports time courses of phosphorylation for the 55 S/T/Y residues considered in the model. The time courses, which are clustered by similarity, are representative of results obtained with other parameter values, in that the model consistently predicts distinct kinetics for different sites of phosphorylation. Thus, multiple sites of phosphorylation can be lumped together only with careful consideration, because in general, the kinetics of phosphorylation can be site specific. It is possible to generate the results of Figure 4 because the cost of network-free simulation, which was applied to obtain these results, depends on the number of rules in a model, not the number of reactions or chemical species implied by the rules.

## Discussion

Here, we have presented a dynamical model for ERBB receptor signaling that captures site-specific mechanistic details and demonstrated that the model can be visualized, annotated, and simulated. Many dynamical models have been formulated for ERBB receptor signaling through the traditional approach for modeling chemical kinetics, i.e., ODE modeling. In general, ODE models for cellular regulatory systems track the populations of only 10’s to 100’s of chemical species [42]. Our model accounts implicitly for many more species (Additional file 1). The discrepancy in size is attributable to omission of site-specific details about protein-protein interactions in ODE models and the simplifying assumptions of ODE models that are introduced for the sake of making model specification feasible. The simplifying assumptions typical of ODE models often conflict with our knowledge of cellular biochemistry (for further discussion, see [43]). An example of such an assumption is the use of a ‘virtual phosphorylation site’ to represent all sites of phosphorylation within a protein [15]. Such an assumption can be problematic or undesirable for a number of reasons. For example, for adaptor proteins that interact with different sites on a receptor, the virtual phosphorylation site assumption introduces a false competition.

Although our model is large when measured in terms of potentially populated chemical species, the number of parameters in the model is comparable to the number of parameters in an ODE model for ERBB receptor signaling [42]. For example, the model of Chen et al. [14], which tracks 499 chemical species, has 229 parameters. The number of parameters in a rule-based model depends on the number of rules comprising the model rather than the number of chemical species or reactions implied by the rules [10]. The model presented here has 543 parameters.

How should we view the increase in number of parameters from 299 to 543? Model selection criteria used in statistics, such as the Akaike information criterion, incorporate penalties for the number of parameters in a model. Thus, one might view our model as inferior to the model of Chen et al. [14]. However, this perspective ignores the fact that our model, like the model of Chen et al. [14], was formulated not to serve as a fitting function but rather to serve as a “vehicle of understanding” [44]. If only a fitting function is desired, neither of these models is likely to be a good choice given any typical collection of data. However, if one desires a model that can be used to reason about mechanism, then the model presented here better captures the site-specific details that are known from experimental studies of ERBB receptor signaling, and it is better able to connect to multiplex temporal phosphoproteomic data, which can be generated in principle. Moreover, a rule-based model that captures site-specific details may actually be a better fitting function than an ODE model. For example, consider a protein with multiple sites of phosphorylation. If we wish to model the phosphorylation dynamics of this protein, and we can only measure phosphorylation using a pan antibody, then a virtual phosphorylation site assumption and ODE model may be justified. However, if phosphospecific antibodies are available, and the different sites in the protein have different phosphorylation kinetics, then a (rule-based) model that treats the sites individually may be superior according to a model selection criterion, despite the introduction of additional parameters, because the best that a model that lumps sites together can do is reproduce the average phosphorylation dynamics, which may not represent the dynamics of any individual site. In the simple example considered, a rule-based model may be unnecessary, but the size of an ODE model tends to increase exponentially as components are added if the model incorporates site-specific details [7,8,10] and eventually a rule-based approach would be required.

An important aspect of the model presented here is its ability to make predictions about specific sites of phosphorylation. Phosphorylation of individual sites can be experimentally detected and monitored as a function of time after a perturbation of a cell signaling network using various multiplex techniques, such as reverse phase protein array (RPPA), high-throughput microwestern blotting, and quantitative mass spectrometry (MS). Time courses for many of the sites considered in the model have been measured using these techniques [45-47] (Table 2). Table 2 indicates which sites considered in the model were assayed in each of three proteomic studies. Although no single study has generated time courses for all 55 sites of phosphorylation (or a significant fraction of these sites with fine time resolution), it seems that, in principle, multiplex temporal phosphoproteomic data can be generated that would be useful for identifying the parameters of the model presented here, or other such models. ODE models cannot connect to multiplex phosphoproteomic data because of the limited ability of these models to track individual sites of phosphorylation.

**Table 2:** Summary of selected temporal phosphoproteomic data

**Protein**	**Residue**	**Wolf-Yadlin et al. (2007)**	**VanMeter et al. (2009)**	**Ciaccio et al. (2010)**
ERBB1 (EGFR)				
	Y845			X
	Y992		X	
	Y998	X		
	Y1045	X		
	Y1068		X	X
	Y1086			X
	Y1148	X	X	
	Y1173	X	X	X
ERBB2 (HER2)				
	Y1221/1222			X
	Y1248	X	X	
ERBB3 (HER3)				
	Y1289			X
	Y1328			
ERBB4				
	Y1284			X
Shc1				
	Y239/240	X		X
	Y317	X	X	X
Raf-1				
	S289/296/301			X
	S338			X
MEK1/2				
	S217/T221			X
ERK1/2				
	T202/Y204	X	X	X
Gab1				
	Y373	X		
	Y406	X		
	Y627	X	X	X
	Y659	X		
Akt1				
	T308		X	X
	S473		X	X

Time courses have been measured for many of the serine, threonine and tyrosine (S/T/Y) residues considered in the model presented here. Here, we focus on three studies that applied distinct experimental techniques to measure time courses of phosphorylation for specific S/T/Y sites. In the study of Wolf-Yadlin et al. [45], the technique of selected reaction monitoring and quantitative mass spectrometry was applied. In the study of VanMeter et al. [46], the technique of reverse phase protein array was applied. In the study of Ciaccio et al. [47], the technique of microwestern array was applied. An ‘X’ entry in this table signifies that a time course of phosphorylation for the indicated residue was measured in the indicated study.

Generation of data needed to begin validation of a large rule-based model would be a resource-intensive undertaking and one that ideally would involve not only use of multiplex data to estimate model parameter values but also carefully designed experimental tests of model predictions. It is unlikely that such an undertaking would ever start without a demonstration that modeling aspects of a study focused on site-specific mechanistic details are feasible. Providing a demonstration of key modeling capabilities needed for this type of study was the rationale behind this report. Models reported in the literature that are closest in character to that reported here are perhaps the models of Thomson et al. [28] and Tiger et al. [48], which are large rule-based models for cell signaling systems in yeast.

## Conclusion

In conclusion, with the development of network-free simulation tools, it is now possible to build and analyze rule-based models that capture a significant fraction of the proteins and protein-protein interactions in a cell signaling network with consideration of site-specific mechanistic details. The next challenge is to apply this type of modeling to gain new biological insights. The ERBB receptor signaling network is important in cancer, so it may be especially interesting to study how best to target the network when it is affected by known mutations. For an example of such a study, see Stites et al. [4]. In the future, we anticipate that rule-based modeling will become a tool routinely used in proteomic and systems biology studies, enabling the development of more mechanistic, validated, and predictive models for cell signaling networks.

## Methods

### Specification of the model structure

Our model was specified with the intention of extending the model of Chen et al. [14] by adding consideration of the site-specific details of protein interactions. Thus, the proteins considered in the model of Chen et al. [14] defined the scope of our modeling effort. Our model was specified on the basis of an extensive literature search. Electronic repositories of biological knowledge [35,36,49-55] and expert knowledge of the modeling team were also helpful. Mechanistic knowledge was formalized using the BioNetGen language (BNGL) [56]. We defined molecule type graphs (19 total graphs) to represent molecules (18 proteins and phosphatidylinositol) and rules (544) to represent molecular interactions and other processes (viz. transport and degradation). We also defined observables for the purpose of reporting time courses of phosphorylation for specific S/T/Y residues included in the model. The model accounts for several compartments of a single cell: surrounding extracellular fluid, the plasma membrane, the cytoplasm, and endosomes, where internalized ligands are degraded. A complete specification of the model is provided in the supplemental material (see ERBB_model.bngl, Additional file 1). The model specification is given in the form of a BioNetGen input file, which is a plain-text file [56]. The model specification includes a list of the 544 rules of the model as well as a list of nominal parameter values (see below). A BioNetGen input file can be processed by a number of software tools, including BioNetGen [9,56] and NFsim [13], which were used in this study.

### Model visualization

The extended contact map of Figure 2 is drawn according to the conventions of Chylek et al. [22]. The map was created manually using the OmniGraffle drawing tool (The Omni Group, Seattle, WA). An OmniGraffle stencil is available for drawing extended contact maps [22]. The stencil can be obtained from the BioNetGen wiki site ( http://bionetgen.org). A tool for automatic visualization of a rule-based model specification, *rxncon*, has recently become available [48]; this tool produces maps that have similarities with an extended contact map. The purpose of an extended contact map is to provide a high-level understandable illustration of the material components, post-translational modifications, and interactions included in a model. Only material components, post-translational modifications, and interactions explicitly included in our model are represented in Figure 2. Material components are represented by boxes, and nesting of boxes is used to illustrate structural relationships. Post-translational modifications are represented by flags (i.e., small square boxes connected to text labels). Interactions are represented by arrows, which are each linked to a set of rules (see below). In an extended contact map, for simplicity, no attempt is made to illustrate the contextual dependencies of interactions; instead, contextual dependencies are captured in the rules linked to arrows. Two types of arrows are used in Figure 2. Lines with two arrowheads are used to connect material components responsible for protein-protein and protein-lipid interactions. Lines that end with a circle are used to connect enzymes and substrates. The conventions of extended contact maps are further described, in great detail, elsewhere [22].

### Model annotation

As recommended by Chylek et al. [22], a model guide was prepared for the purpose of linking rules in the model to arrows of the extended contact map of Figure 2 and for the purpose of annotating the model. The guide is provided in the form of a TiddlyWiki ( http://tiddlywiki.com/), which is a single-page wiki application. The guide is available online ( https://modeling.tgen.org/). It can also be inspected by using a web browser to open the HTML document included in the supplementary archive file (Additional file 2). The guide includes cartoon illustrations of proteins, which were prepared using the DOG software tool [57]. The guide also includes links to a variety of information available in online resources, including UniProt [35], OMIM ( http://omim.org/), PubMed ( http://www.ncbi.nlm.nih.gov/pubmed), Pfam [36], and KEGG [49]. Additional resources used in model development and annotation included NetPath [50], HPRD [51], Phospho. ELM [52], PTMScout [53], ChEBI [54], and ELM [55]. The elements (pages) of a TiddlyWiki are called Tiddlers. Tiddlers are available within our TiddlyWiki that identify the compartments, proteins, domains, linear motifs, phosphorylation sites, metabolites, and interactions considered in the model. The formal elements of the model include molecule type definitions and rules, for which Tiddlers are also provided. Tiddlers for rules are cross-referenced with arrows in the extended contact map of Figure 2. For a full discussion of the concept of a model guide, as well as a different example of a guide, see Chylek et al. [22].

### Parameter values

In general, to simulate a rule-based model, one must assign copy numbers to molecules and rate constants to rate laws. In a rule-based model, a rate law is associated with every rule. For our model, each rate law has a form consistent with mass-action chemical reaction kinetics. The parameters of the model (543) were divided into six classes and a feasible range was estimated for each class (Table 1). It is possible to specify feasible ranges for parameter values because the types of interactions considered in our model have been systematically and quantitatively studied. For example, interactions of SH2 domain-containing proteins with phosphotyrosine binding partners in ERBB receptors have been characterized using a protein microarray-based approach [58]. Care was taken to respect physicochemical constraints on parameter values. For example, rate constants for bimolecular association reactions were not allowed to exceed the upper limit set by diffusion [59]. An ensemble of 1000 sets of parameter values was generated by sampling values from the estimated feasible ranges. Care was also taken to ensure satisfaction of detailed balance [60,61]. The nominal parameter values specified in the BioNetGen input file of Supplementary Archive File 1 were chosen arbitrarily from among the ensemble of parameter values considered, because with these parameter values, the model produces time courses of phosphorylation for ERK (T185 in ERK2) and Akt (S473 in Akt1) that are deemed to be reasonable. We caution that the nominal parameter values have not been validated; parameter estimation on the basis of empirical data is beyond the intended scope of our study. Parameter values in the BioNetGen input file of Supplementary Archive File 1 are specified using the unit system recommended by Faeder et al. [56].

### Simulation

Network-free simulation is a particle-based, or agent-based, approach that involves tracking individual molecules and molecular components; the cost of simulation depends on the number of molecules, molecular components, and rules considered but not the number of chemical species or reactions implied by rules [40,41]. Software that implements network-free simulation methods, such as RuleMonkey [12], NFsim [13], and KaSim ( http://kappalanguage.org), can be used for simulating large-scale reaction networks. The results of Figure 4 were generated using NFsim v1.09, which is an efficient implementation of the network-free simulation algorithm of Yang et al. [40]. In the simulations performed to produce the results of Figure 4, compartment sizes were scaled by a factor of 0.2 to decrease the computational expense of simulation. Before time *t* = 0, the system is in a steady state and no ligand is present. At *t* = 0, epidermal growth factor and heregulin are added (at a concentration of 5 nM each), as indicated in the BioNetGen input file that defines the model (Additional file 1). A single simulation run does not require special computational resources; a laptop can be used to reproduce the simulations performed with NFsim.

The results of Figure 3A were generated using the on-the-fly stochastic simulation algorithm implemented in BioNetGen [39,56]. On-the-fly simulation is a population-based simulation approach that involves lazy evaluation of rules to generate a partial list of possible reactions. We considered full compartment sizes for these simulations, which were expensive, requiring several days of computation and significant memory usage. For these simulations, we used an Altix 4700 machine with 576 GB of shared memory (SGI, Fremont, CA). Because the number of populated species and the size of the reaction network encompassing the populated species grow exponentially as a function of time, it quickly becomes impossible to simulate the model using on-the-fly simulation, even with a supercomputer. Figure 3A shows how the cost of on-the-fly simulation increases exponentially as simulated time and network size increase. In contrast, the cost of simulation via the network-free approach increases only linearly (Figure 3). *NB:* for the simulations of Figure 3B, equilibration (i.e., simulation for sufficient time to reach steady state before addition of ligands) was not performed so that the initial condition would encompass a minimal number of populated chemical species. In these simulations, at time *t* = 0, all proteins were free and unphosphorylated.

### Clustering

Time courses reported in Figure 4 were normalized by dividing each simulated phosphorylation level by the corresponding maximum phosphorylation level recorded over the course of simulation. Normalized time courses were then ordered using hierarchical clustering, average linkage, and the Pearson correlation metric. The heat map of Figure 4 was constructed using the GenePattern software tool [62].

## Abbreviations

BNGL: BioNetGen Language; ODE: Ordinary differential equation.

## Competing interests

The authors declare that they have no competing interests.

## Authors’ contributions

MSC, ECS, RGP, WSH, MEB, and DVH designed the research. MSC, ECS, JAC, CWT, RGP, HH, KJB and WSH contributed to specification, visualization and annotation of the model. ECS and MSC performed simulations. MA performed clustering. RGP, WSH, and ECS wrote the manuscript with input from the other authors. All authors read and approved the final manuscript.

## Supplementary Material

Additional file 1 **ErbB_model.bngl.** This plain-text file is a BioNetGen input file. It contains a specification of the model structure as well as nominal parameter values. (BNGL 123 kb) Click here for file

Additional file 2 **ModelGuideWiki.zip.** This archive file provides a copy of the files available online (https://modeling.tgen.org). These files serve to annotate the model. (ZIP 759 kb) Click here for file
