# Supplementary material for: Specification, annotation, visualization and simulation of a large rule-based model for ERBB receptor signaling
Source: BMC Syst Biol. 2012 Aug 22;6:107. doi: 10.1186/1752-0509-6-107 (PMC3485121; doi:10.1186/1752-0509-6-107)
Supplement: Additional file 2 — ModelGuideWiki.zip. This archive file provides a copy of the files available online (https://modeling.tgen.org). These files serve to annotate the model. (ZIP 759 kb) [file 1752-0509-6-107-S2.zip › ModelGuideWiki/ModelGuideWiki.html]

 ModelGuideWiki - annotation of a rule-based for ErbB-mediated activation of ERK and AKT 


Welcome to TiddlyWiki created by Jeremy Ruston; Copyright © 2004-2007 Jeremy Ruston, Copyright © 2007-2011 UnaMesa Association

This page requires JavaScript to function properly.  
  
If you are using Microsoft Internet Explorer you may need to click on the yellow bar above and select 'Allow Blocked Content'. You must then click 'Yes' on the following security warning.

```
<!--{{{-->
<link rel='alternate' type='application/rss+xml' title='RSS' href='index.xml' />
<!--}}}-->
```

```
Background: #fff
Foreground: #000
PrimaryPale: #8cf
PrimaryLight: #18f
PrimaryMid: #04b
PrimaryDark: #014
SecondaryPale: #ffc
SecondaryLight: #fe8
SecondaryMid: #db4
SecondaryDark: #841
TertiaryPale: #eee
TertiaryLight: #ccc
TertiaryMid: #999
TertiaryDark: #666
Error: #f88
```

```
/*{{{*/
body {background:[[ColorPalette::Background]]; color:[[ColorPalette::Foreground]];}

a {color:[[ColorPalette::PrimaryMid]];}
a:hover {background-color:[[ColorPalette::PrimaryMid]]; color:[[ColorPalette::Background]];}
a img {border:0;}

h1,h2,h3,h4,h5,h6 {color:[[ColorPalette::SecondaryDark]]; background:transparent;}
h1 {border-bottom:2px solid [[ColorPalette::TertiaryLight]];}
h2,h3 {border-bottom:1px solid [[ColorPalette::TertiaryLight]];}

.button {color:[[ColorPalette::PrimaryDark]]; border:1px solid [[ColorPalette::Background]];}
.button:hover {color:[[ColorPalette::PrimaryDark]]; background:[[ColorPalette::SecondaryLight]]; border-color:[[ColorPalette::SecondaryMid]];}
.button:active {color:[[ColorPalette::Background]]; background:[[ColorPalette::SecondaryMid]]; border:1px solid [[ColorPalette::SecondaryDark]];}

.header {background:[[ColorPalette::PrimaryMid]];}
.headerShadow {color:[[ColorPalette::Foreground]];}
.headerShadow a {font-weight:normal; color:[[ColorPalette::Foreground]];}
.headerForeground {color:[[ColorPalette::Background]];}
.headerForeground a {font-weight:normal; color:[[ColorPalette::PrimaryPale]];}

.tabSelected{color:[[ColorPalette::PrimaryDark]];
	background:[[ColorPalette::TertiaryPale]];
	border-left:1px solid [[ColorPalette::TertiaryLight]];
	border-top:1px solid [[ColorPalette::TertiaryLight]];
	border-right:1px solid [[ColorPalette::TertiaryLight]];
}
.tabUnselected {color:[[ColorPalette::Background]]; background:[[ColorPalette::TertiaryMid]];}
.tabContents {color:[[ColorPalette::PrimaryDark]]; background:[[ColorPalette::TertiaryPale]]; border:1px solid [[ColorPalette::TertiaryLight]];}
.tabContents .button {border:0;}

#sidebar {}
#sidebarOptions input {border:1px solid [[ColorPalette::PrimaryMid]];}
#sidebarOptions .sliderPanel {background:[[ColorPalette::PrimaryPale]];}
#sidebarOptions .sliderPanel a {border:none;color:[[ColorPalette::PrimaryMid]];}
#sidebarOptions .sliderPanel a:hover {color:[[ColorPalette::Background]]; background:[[ColorPalette::PrimaryMid]];}
#sidebarOptions .sliderPanel a:active {color:[[ColorPalette::PrimaryMid]]; background:[[ColorPalette::Background]];}

.wizard {background:[[ColorPalette::PrimaryPale]]; border:1px solid [[ColorPalette::PrimaryMid]];}
.wizard h1 {color:[[ColorPalette::PrimaryDark]]; border:none;}
.wizard h2 {color:[[ColorPalette::Foreground]]; border:none;}
.wizardStep {background:[[ColorPalette::Background]]; color:[[ColorPalette::Foreground]];
	border:1px solid [[ColorPalette::PrimaryMid]];}
.wizardStep.wizardStepDone {background:[[ColorPalette::TertiaryLight]];}
.wizardFooter {background:[[ColorPalette::PrimaryPale]];}
.wizardFooter .status {background:[[ColorPalette::PrimaryDark]]; color:[[ColorPalette::Background]];}
.wizard .button {color:[[ColorPalette::Foreground]]; background:[[ColorPalette::SecondaryLight]]; border: 1px solid;
	border-color:[[ColorPalette::SecondaryPale]] [[ColorPalette::SecondaryDark]] [[ColorPalette::SecondaryDark]] [[ColorPalette::SecondaryPale]];}
.wizard .button:hover {color:[[ColorPalette::Foreground]]; background:[[ColorPalette::Background]];}
.wizard .button:active {color:[[ColorPalette::Background]]; background:[[ColorPalette::Foreground]]; border: 1px solid;
	border-color:[[ColorPalette::PrimaryDark]] [[ColorPalette::PrimaryPale]] [[ColorPalette::PrimaryPale]] [[ColorPalette::PrimaryDark]];}

.wizard .notChanged {background:transparent;}
.wizard .changedLocally {background:#80ff80;}
.wizard .changedServer {background:#8080ff;}
.wizard .changedBoth {background:#ff8080;}
.wizard .notFound {background:#ffff80;}
.wizard .putToServer {background:#ff80ff;}
.wizard .gotFromServer {background:#80ffff;}

#messageArea {border:1px solid [[ColorPalette::SecondaryMid]]; background:[[ColorPalette::SecondaryLight]]; color:[[ColorPalette::Foreground]];}
#messageArea .button {color:[[ColorPalette::PrimaryMid]]; background:[[ColorPalette::SecondaryPale]]; border:none;}

.popupTiddler {background:[[ColorPalette::TertiaryPale]]; border:2px solid [[ColorPalette::TertiaryMid]];}

.popup {background:[[ColorPalette::TertiaryPale]]; color:[[ColorPalette::TertiaryDark]]; border-left:1px solid [[ColorPalette::TertiaryMid]]; border-top:1px solid [[ColorPalette::TertiaryMid]]; border-right:2px solid [[ColorPalette::TertiaryDark]]; border-bottom:2px solid [[ColorPalette::TertiaryDark]];}
.popup hr {color:[[ColorPalette::PrimaryDark]]; background:[[ColorPalette::PrimaryDark]]; border-bottom:1px;}
.popup li.disabled {color:[[ColorPalette::TertiaryMid]];}
.popup li a, .popup li a:visited {color:[[ColorPalette::Foreground]]; border: none;}
.popup li a:hover {background:[[ColorPalette::SecondaryLight]]; color:[[ColorPalette::Foreground]]; border: none;}
.popup li a:active {background:[[ColorPalette::SecondaryPale]]; color:[[ColorPalette::Foreground]]; border: none;}
.popupHighlight {background:[[ColorPalette::Background]]; color:[[ColorPalette::Foreground]];}
.listBreak div {border-bottom:1px solid [[ColorPalette::TertiaryDark]];}

.tiddler .defaultCommand {font-weight:bold;}

.shadow .title {color:[[ColorPalette::TertiaryDark]];}

.title {color:[[ColorPalette::SecondaryDark]];}
.subtitle {color:[[ColorPalette::TertiaryDark]];}

.toolbar {color:[[ColorPalette::PrimaryMid]];}
.toolbar a {color:[[ColorPalette::TertiaryLight]];}
.selected .toolbar a {color:[[ColorPalette::TertiaryMid]];}
.selected .toolbar a:hover {color:[[ColorPalette::Foreground]];}

.tagging, .tagged {border:1px solid [[ColorPalette::TertiaryPale]]; background-color:[[ColorPalette::TertiaryPale]];}
.selected .tagging, .selected .tagged {background-color:[[ColorPalette::TertiaryLight]]; border:1px solid [[ColorPalette::TertiaryMid]];}
.tagging .listTitle, .tagged .listTitle {color:[[ColorPalette::PrimaryDark]];}
.tagging .button, .tagged .button {border:none;}

.footer {color:[[ColorPalette::TertiaryLight]];}
.selected .footer {color:[[ColorPalette::TertiaryMid]];}

.sparkline {background:[[ColorPalette::PrimaryPale]]; border:0;}
.sparktick {background:[[ColorPalette::PrimaryDark]];}

.error, .errorButton {color:[[ColorPalette::Foreground]]; background:[[ColorPalette::Error]];}
.warning {color:[[ColorPalette::Foreground]]; background:[[ColorPalette::SecondaryPale]];}
.lowlight {background:[[ColorPalette::TertiaryLight]];}

.zoomer {background:none; color:[[ColorPalette::TertiaryMid]]; border:3px solid [[ColorPalette::TertiaryMid]];}

.imageLink, #displayArea .imageLink {background:transparent;}

.annotation {background:[[ColorPalette::SecondaryLight]]; color:[[ColorPalette::Foreground]]; border:2px solid [[ColorPalette::SecondaryMid]];}

.viewer .listTitle {list-style-type:none; margin-left:-2em;}
.viewer .button {border:1px solid [[ColorPalette::SecondaryMid]];}
.viewer blockquote {border-left:3px solid [[ColorPalette::TertiaryDark]];}

.viewer table, table.twtable {border:2px solid [[ColorPalette::TertiaryDark]];}
.viewer th, .viewer thead td, .twtable th, .twtable thead td {background:[[ColorPalette::SecondaryMid]]; border:1px solid [[ColorPalette::TertiaryDark]]; color:[[ColorPalette::Background]];}
.viewer td, .viewer tr, .twtable td, .twtable tr {border:1px solid [[ColorPalette::TertiaryDark]];}

.viewer pre {border:1px solid [[ColorPalette::SecondaryLight]]; background:[[ColorPalette::SecondaryPale]];}
.viewer code {color:[[ColorPalette::SecondaryDark]];}
.viewer hr {border:0; border-top:dashed 1px [[ColorPalette::TertiaryDark]]; color:[[ColorPalette::TertiaryDark]];}

.highlight, .marked {background:[[ColorPalette::SecondaryLight]];}

.editor input {border:1px solid [[ColorPalette::PrimaryMid]];}
.editor textarea {border:1px solid [[ColorPalette::PrimaryMid]]; width:100%;}
.editorFooter {color:[[ColorPalette::TertiaryMid]];}
.readOnly {background:[[ColorPalette::TertiaryPale]];}

#backstageArea {background:[[ColorPalette::Foreground]]; color:[[ColorPalette::TertiaryMid]];}
#backstageArea a {background:[[ColorPalette::Foreground]]; color:[[ColorPalette::Background]]; border:none;}
#backstageArea a:hover {background:[[ColorPalette::SecondaryLight]]; color:[[ColorPalette::Foreground]]; }
#backstageArea a.backstageSelTab {background:[[ColorPalette::Background]]; color:[[ColorPalette::Foreground]];}
#backstageButton a {background:none; color:[[ColorPalette::Background]]; border:none;}
#backstageButton a:hover {background:[[ColorPalette::Foreground]]; color:[[ColorPalette::Background]]; border:none;}
#backstagePanel {background:[[ColorPalette::Background]]; border-color: [[ColorPalette::Background]] [[ColorPalette::TertiaryDark]] [[ColorPalette::TertiaryDark]] [[ColorPalette::TertiaryDark]];}
.backstagePanelFooter .button {border:none; color:[[ColorPalette::Background]];}
.backstagePanelFooter .button:hover {color:[[ColorPalette::Foreground]];}
#backstageCloak {background:[[ColorPalette::Foreground]]; opacity:0.6; filter:'alpha(opacity=60)';}
/*}}}*/
```

```
/*{{{*/
* html .tiddler {height:1%;}

body {font-size:.75em; font-family:arial,helvetica; margin:0; padding:0;}

h1,h2,h3,h4,h5,h6 {font-weight:bold; text-decoration:none;}
h1,h2,h3 {padding-bottom:1px; margin-top:1.2em;margin-bottom:0.3em;}
h4,h5,h6 {margin-top:1em;}
h1 {font-size:1.35em;}
h2 {font-size:1.25em;}
h3 {font-size:1.1em;}
h4 {font-size:1em;}
h5 {font-size:.9em;}

hr {height:1px;}

a {text-decoration:none;}

dt {font-weight:bold;}

ol {list-style-type:decimal;}
ol ol {list-style-type:lower-alpha;}
ol ol ol {list-style-type:lower-roman;}
ol ol ol ol {list-style-type:decimal;}
ol ol ol ol ol {list-style-type:lower-alpha;}
ol ol ol ol ol ol {list-style-type:lower-roman;}
ol ol ol ol ol ol ol {list-style-type:decimal;}

.txtOptionInput {width:11em;}

#contentWrapper .chkOptionInput {border:0;}

.externalLink {text-decoration:underline;}

.indent {margin-left:3em;}
.outdent {margin-left:3em; text-indent:-3em;}
code.escaped {white-space:nowrap;}

.tiddlyLinkExisting {font-weight:bold;}
.tiddlyLinkNonExisting {font-style:italic;}

/* the 'a' is required for IE, otherwise it renders the whole tiddler in bold */
a.tiddlyLinkNonExisting.shadow {font-weight:bold;}

#mainMenu .tiddlyLinkExisting,
	#mainMenu .tiddlyLinkNonExisting,
	#sidebarTabs .tiddlyLinkNonExisting {font-weight:normal; font-style:normal;}
#sidebarTabs .tiddlyLinkExisting {font-weight:bold; font-style:normal;}

.header {position:relative;}
.header a:hover {background:transparent;}
.headerShadow {position:relative; padding:4.5em 0 1em 1em; left:-1px; top:-1px;}
.headerForeground {position:absolute; padding:4.5em 0 1em 1em; left:0px; top:0px;}

.siteTitle {font-size:3em;}
.siteSubtitle {font-size:1.2em;}

#mainMenu {position:absolute; left:0; width:10em; text-align:right; line-height:1.6em; padding:1.5em 0.5em 0.5em 0.5em; font-size:1.1em;}

#sidebar {position:absolute; right:3px; width:16em; font-size:.9em;}
#sidebarOptions {padding-top:0.3em;}
#sidebarOptions a {margin:0 0.2em; padding:0.2em 0.3em; display:block;}
#sidebarOptions input {margin:0.4em 0.5em;}
#sidebarOptions .sliderPanel {margin-left:1em; padding:0.5em; font-size:.85em;}
#sidebarOptions .sliderPanel a {font-weight:bold; display:inline; padding:0;}
#sidebarOptions .sliderPanel input {margin:0 0 0.3em 0;}
#sidebarTabs .tabContents {width:15em; overflow:hidden;}

.wizard {padding:0.1em 1em 0 2em;}
.wizard h1 {font-size:2em; font-weight:bold; background:none; padding:0; margin:0.4em 0 0.2em;}
.wizard h2 {font-size:1.2em; font-weight:bold; background:none; padding:0; margin:0.4em 0 0.2em;}
.wizardStep {padding:1em 1em 1em 1em;}
.wizard .button {margin:0.5em 0 0; font-size:1.2em;}
.wizardFooter {padding:0.8em 0.4em 0.8em 0;}
.wizardFooter .status {padding:0 0.4em; margin-left:1em;}
.wizard .button {padding:0.1em 0.2em;}

#messageArea {position:fixed; top:2em; right:0; margin:0.5em; padding:0.5em; z-index:2000; _position:absolute;}
.messageToolbar {display:block; text-align:right; padding:0.2em;}
#messageArea a {text-decoration:underline;}

.tiddlerPopupButton {padding:0.2em;}
.popupTiddler {position: absolute; z-index:300; padding:1em; margin:0;}

.popup {position:absolute; z-index:300; font-size:.9em; padding:0; list-style:none; margin:0;}
.popup .popupMessage {padding:0.4em;}
.popup hr {display:block; height:1px; width:auto; padding:0; margin:0.2em 0;}
.popup li.disabled {padding:0.4em;}
.popup li a {display:block; padding:0.4em; font-weight:normal; cursor:pointer;}
.listBreak {font-size:1px; line-height:1px;}
.listBreak div {margin:2px 0;}

.tabset {padding:1em 0 0 0.5em;}
.tab {margin:0 0 0 0.25em; padding:2px;}
.tabContents {padding:0.5em;}
.tabContents ul, .tabContents ol {margin:0; padding:0;}
.txtMainTab .tabContents li {list-style:none;}
.tabContents li.listLink { margin-left:.75em;}

#contentWrapper {display:block;}
#splashScreen {display:none;}

#displayArea {margin:1em 17em 0 14em;}

.toolbar {text-align:right; font-size:.9em;}

.tiddler {padding:1em 1em 0;}

.missing .viewer,.missing .title {font-style:italic;}

.title {font-size:1.6em; font-weight:bold;}

.missing .subtitle {display:none;}
.subtitle {font-size:1.1em;}

.tiddler .button {padding:0.2em 0.4em;}

.tagging {margin:0.5em 0.5em 0.5em 0; float:left; display:none;}
.isTag .tagging {display:block;}
.tagged {margin:0.5em; float:right;}
.tagging, .tagged {font-size:0.9em; padding:0.25em;}
.tagging ul, .tagged ul {list-style:none; margin:0.25em; padding:0;}
.tagClear {clear:both;}

.footer {font-size:.9em;}
.footer li {display:inline;}

.annotation {padding:0.5em; margin:0.5em;}

* html .viewer pre {width:99%; padding:0 0 1em 0;}
.viewer {line-height:1.4em; padding-top:0.5em;}
.viewer .button {margin:0 0.25em; padding:0 0.25em;}
.viewer blockquote {line-height:1.5em; padding-left:0.8em;margin-left:2.5em;}
.viewer ul, .viewer ol {margin-left:0.5em; padding-left:1.5em;}

.viewer table, table.twtable {border-collapse:collapse; margin:0.8em 1.0em;}
.viewer th, .viewer td, .viewer tr,.viewer caption,.twtable th, .twtable td, .twtable tr,.twtable caption {padding:3px;}
table.listView {font-size:0.85em; margin:0.8em 1.0em;}
table.listView th, table.listView td, table.listView tr {padding:0px 3px 0px 3px;}

.viewer pre {padding:0.5em; margin-left:0.5em; font-size:1.2em; line-height:1.4em; overflow:auto;}
.viewer code {font-size:1.2em; line-height:1.4em;}

.editor {font-size:1.1em;}
.editor input, .editor textarea {display:block; width:100%; font:inherit;}
.editorFooter {padding:0.25em 0; font-size:.9em;}
.editorFooter .button {padding-top:0px; padding-bottom:0px;}

.fieldsetFix {border:0; padding:0; margin:1px 0px;}

.sparkline {line-height:1em;}
.sparktick {outline:0;}

.zoomer {font-size:1.1em; position:absolute; overflow:hidden;}
.zoomer div {padding:1em;}

* html #backstage {width:99%;}
* html #backstageArea {width:99%;}
#backstageArea {display:none; position:relative; overflow: hidden; z-index:150; padding:0.3em 0.5em;}
#backstageToolbar {position:relative;}
#backstageArea a {font-weight:bold; margin-left:0.5em; padding:0.3em 0.5em;}
#backstageButton {display:none; position:absolute; z-index:175; top:0; right:0;}
#backstageButton a {padding:0.1em 0.4em; margin:0.1em;}
#backstage {position:relative; width:100%; z-index:50;}
#backstagePanel {display:none; z-index:100; position:absolute; width:90%; margin-left:3em; padding:1em;}
.backstagePanelFooter {padding-top:0.2em; float:right;}
.backstagePanelFooter a {padding:0.2em 0.4em;}
#backstageCloak {display:none; z-index:20; position:absolute; width:100%; height:100px;}

.whenBackstage {display:none;}
.backstageVisible .whenBackstage {display:block;}
/*}}}*/
```

```
/***
StyleSheet for use when a translation requires any css style changes.
This StyleSheet can be used directly by languages such as Chinese, Japanese and Korean which need larger font sizes.
***/
/*{{{*/
body {font-size:0.8em;}
#sidebarOptions {font-size:1.05em;}
#sidebarOptions a {font-style:normal;}
#sidebarOptions .sliderPanel {font-size:0.95em;}
.subtitle {font-size:0.8em;}
.viewer table.listView {font-size:0.95em;}
/*}}}*/
```

```
/*{{{*/
@media print {
#mainMenu, #sidebar, #messageArea, .toolbar, #backstageButton, #backstageArea {display: none !important;}
#displayArea {margin: 1em 1em 0em;}
noscript {display:none;} /* Fixes a feature in Firefox 1.5.0.2 where print preview displays the noscript content */
}
/*}}}*/
```

```
<!--{{{-->
<div class='header' macro='gradient vert [[ColorPalette::PrimaryLight]] [[ColorPalette::PrimaryMid]]'>
<div class='headerShadow'>
<span class='siteTitle' refresh='content' tiddler='SiteTitle'></span>&nbsp;
<span class='siteSubtitle' refresh='content' tiddler='SiteSubtitle'></span>
</div>
<div class='headerForeground'>
<span class='siteTitle' refresh='content' tiddler='SiteTitle'></span>&nbsp;
<span class='siteSubtitle' refresh='content' tiddler='SiteSubtitle'></span>
</div>
</div>
<div id='mainMenu' refresh='content' tiddler='MainMenu'></div>
<div id='sidebar'>
<div id='sidebarOptions' refresh='content' tiddler='SideBarOptions'></div>
<div id='sidebarTabs' refresh='content' force='true' tiddler='SideBarTabs'></div>
</div>
<div id='displayArea'>
<div id='messageArea'></div>
<div id='tiddlerDisplay'></div>
</div>
<!--}}}-->
```

```
<!--{{{-->
<div class='toolbar' macro='toolbar [[ToolbarCommands::ViewToolbar]]'></div>
<div class='title' macro='view title'></div>
<div class='subtitle'><span macro='view modifier link'></span>, <span macro='view modified date'></span> (<span macro='message views.wikified.createdPrompt'></span> <span macro='view created date'></span>)</div>
<div class='tagging' macro='tagging'></div>
<div class='tagged' macro='tags'></div>
<div class='viewer' macro='view text wikified'></div>
<div class='tagClear'></div>
<!--}}}-->
```

```
<!--{{{-->
<div class='toolbar' macro='toolbar [[ToolbarCommands::EditToolbar]]'></div>
<div class='title' macro='view title'></div>
<div class='editor' macro='edit title'></div>
<div macro='annotations'></div>
<div class='editor' macro='edit text'></div>
<div class='editor' macro='edit tags'></div><div class='editorFooter'><span macro='message views.editor.tagPrompt'></span><span macro='tagChooser excludeLists'></span></div>
<!--}}}-->
```

```
To get started with this blank [[TiddlyWiki]], you'll need to modify the following tiddlers:
* [[SiteTitle]] & [[SiteSubtitle]]: The title and subtitle of the site, as shown above (after saving, they will also appear in the browser title bar)
* [[MainMenu]]: The menu (usually on the left)
* [[DefaultTiddlers]]: Contains the names of the tiddlers that you want to appear when the TiddlyWiki is opened
You'll also need to enter your username for signing your edits: <<option txtUserName>>
```

```
These [[InterfaceOptions]] for customising [[TiddlyWiki]] are saved in your browser

Your username for signing your edits. Write it as a [[WikiWord]] (eg [[JoeBloggs]])

<<option txtUserName>>
<<option chkSaveBackups>> [[SaveBackups]]
<<option chkAutoSave>> [[AutoSave]]
<<option chkRegExpSearch>> [[RegExpSearch]]
<<option chkCaseSensitiveSearch>> [[CaseSensitiveSearch]]
<<option chkAnimate>> [[EnableAnimations]]

----
Also see [[AdvancedOptions]]
```

```
<<importTiddlers>>
```

```
See ''Akt1'' in [[Proteins]]
!BNGL
{{{Akt1(PH,STkinase,T308~O~P,S473~O~P,loc~C)}}}
!Summary
Akt1 (represented by {{{Akt1}}} in the model) is a member of a family of AGC S/T kinases.  The other family members are Akt2 and Akt3.  We take Akt1 to be representative of the entire family.  Akt1 contains a pleckstrin homology (PH) domain, a S/T kinase domain, and C-terminal region characteristic of AGC family kinases (see [[Domains]]).  The PH domain recognizes ~PtdIns(3,4,5)P3.  In the model, we track two sites of phosphorylation within Akt1: T308, a substrate of ~PDK1, and S473, a substrate of mTORC2. mTORC2 is not explicitly included in the model. S473 is located in a hydrophobic motif; T308 is located in the activation loop of the kinase domain. The {{{loc}}} component is included only to indicate that Akt1 freely diffuses in the cytosol.

!DOG
[img[DOGS/Akt1.jpg]]
!Reading
*[[OMIM ID 164730 | http://www.ncbi.nlm.nih.gov/omim/164730]]
*Manning BD, Cantley LC (2007) AKT/PKB signaling: navigating downstream. Cell 129:1261-1274. [[PMID: 17604717 | http://www.ncbi.nlm.nih.gov/pubmed/17604717]]
```

```
''Akt1 catalyzes phosphorylation of Raf1'' (see Arrow 28 in [[Contact Map]])
{{{Raf1(S259~O)->Raf1(S259~P)}}}
!Rules
{{{
535 Akt1(T308~P!?,S473~P,STkinase) + Raf1(S259~O) -> \
 Akt1(T308~P!?,S473~P,STkinase!1).Raf1(S259~O!1)	Akt1kp7

536 Akt1(T308~P!?,S473~P,STkinase!1).Raf1(S259~O!1) -> \
 Akt1(T308~P!?,S473~P,STkinase!1).Raf1(S259~P!1)	Akt1kp8

537 Akt1(STkinase!1).Raf1(S259!1) -> \
 Akt1(STkinase) + Raf1(S259)	Akt1kp9
}}}
```

```
''Akt1 reversibly binds ~PtdIns(3,4,5)P3'' (see Arrow 29 in [[Contact Map]])
{{{Akt1(PH)+PIP3(C3P)<->Akt1(PH!1).PIP3(C3P!1)}}}
!Rules
{{{
529 Akt1(PH) + PIP3(C3P,two~F) <-> \
 Akt1(PH!1).PIP3(C3P!1,two~F)         Akt1kp1,Akt1km1
}}}
```

```
William S. Hlavacek, Ph.D.
Visiting Randy Pausch Scholar
Clinical Translational Research Division
Translational Genomics Research Institute
13208 E. Shea Blvd., Suite 110
Scottsdale, AZ 85259
Tel: 602-358-8315
E-mail: [[bhlavacek@tgen.org | mailto:bhlavacek@tgen.org]]
```

```
The model accounts for extracellular (Ex), membrane (M), endocytic (En), and cytosolic (C) compartments.  Compartmental locations of molecules are indicated in location tabs attached to the bottom left corners of molecule boxes in the [[Contact Map]].
```

```
The extended contact map shown below can be magnified by opening the pdf file in a pdf viewing tool.  You can drag-and-drop or right-click to save the pdf file to your desktop.

[img[ContactMap.pdf]]

This map is drawn according to the conventions of [[Chylek et al. (2011) | http://pubs.rsc.org/en/content/articlelanding/2011/mb/c1mb05077j]] [//Mol. ~BioSyst.// doi:10.1039/c1mb05077j].  Boxes represent proteins.  Nested boxes represent (sub)components of proteins.  The compartmental locations of proteins are noted at the lower left corners of boxes.  A line that begins and ends with an arrowhead represents a direct physical interaction. Solid (dotted) lines represent //trans// (//cis//) interactions.  A line with a circle at one end identifies an enzyme-substrate relationship; the circle identifies the substrate.  Boxes are decorated with post-translational modification flags, which are each labeled at one end and connected to a small box at the other.  The prefix of the label indicates the modification (e.g., ‘p’ represents addition of a phosphate group), and the rest of the label indicates the site of modification.
```

```
[[Introduction]]
```

```
|The protein domains listed in this table are explicitly considered in the model|c
|Short Name |Pfam Family |Protein(s) with Domain |Full Domain Name |h
|EGFL |[[EGF | http://pfam.sanger.ac.uk/family/EGF]] |EGF, ~HRG-&alpha; |~EGF-like domain |
|I, III |[[Recep_L_domain | http://pfam.sanger.ac.uk//family/PF01030]] |EGFR, ~ErbB3, ~ErbB4 |Receptor L domain |
|II |[[Furin-like | http://pfam.sanger.ac.uk//family/PF00757]] |EGFR, ~ErbB2, ~ErbB3, ~ErbB4 |Furin-like cysteine rich region |
|Y kinase |[[Pkinase_Tyr | http://pfam.sanger.ac.uk/family/Pkinase_Tyr]] |EGFR, ~ErbB2, ~ErbB4 |Protein tyrosine kinase domain |
|~SH2 |[[SH2 | http://pfam.sanger.ac.uk/family/sh2]] |p120 ~RasGAP, Grb2, p85&alpha; (2x) |Src homology 2 domain |
|PTB |[[PID | http://pfam.sanger.ac.uk/family/PID]] |p52 Shc1 |Phosphotyrosine binding domain |
|~SH3 |[[SH3_1 | http://pfam.sanger.ac.uk/family/SH3_1]] |Grb2 (2x) |Src homology 3 domain |
|GAP |[[RasGAP | http://pfam.sanger.ac.uk/family/RasGAP]] |p120 ~RasGAP |~GTPase-activator protein |
|~GTPase |[[Ras | http://pfam.sanger.ac.uk//family/PF00071]] |~K-Ras |small ~GTPase |
|GEF |[[RasGEF | http://pfam.sanger.ac.uk/family/RasGEF]] |Sos1 |Guanine nucleotide exchange factor, ~CDC25 homology domain |
|REM |[[RasGEF_N | http://pfam.sanger.ac.uk/family/RasGEF_N]] |Sos1 |Ras exchange motif |
|PH |[[PH | http://pfam.sanger.ac.uk/family/PH]] |Gab1, Akt1, ~PDK1 |Pleckstrin homology domain |
|RBD |[[PI3K_rbd | http://pfam.sanger.ac.uk/family/PI3K_rbd]] |Raf-1, p110&alpha; |Ras-binding domain |
|S/T kinase |[[Pkinase | http://pfam.sanger.ac.uk//family/PF00069]] |Raf-1, ~ERK2, Akt1, ~PDK1 |Serine/threonine kinase |
|lipid kinase |[[PI3_PI4_kinase | http://pfam.sanger.ac.uk//family/PF00454]] |p110&alpha; |Phosphoinositide 3-kinase, catalytic subunit |
|dual specificity kinase |[[Pkinase | http://pfam.sanger.ac.uk//family/PF00069]] |~MEK1 |S/T/Y kinase |
|~AGC-kinase C-terminal |[[Pkinase_C | http://pfam.sanger.ac.uk//family/PF00433]] |Akt1 |C-terminal domain |

Note that the table above lists only the domains that are considered in the model.  Some proteins have domains that are not considered in the model.  For example, Sos1 contains a C2 domain, which is not listed in the table.  Also note that there are cases where a protein contains a domain listed in the table above but this protein is not identified in the table as containing the domain.  An example is p52 Shc1.  This protein contains a ~SH2 domain, but in the row for the ~SH2 domain, p52 Shc1 is not listed.  The reason is that the ~SH2 domain of p52 Shc1 is not included in the model.

The [[Pfam | http://pfam.sanger.ac.uk/]] database and the [[Pawson Lab | http://pawsonlab.mshri.on.ca/index.php?option=com_content&task=view&id=30&Itemid=63]] web site are excellent sources of additional information about protein domains.
```

```
See ''EGF (epidermal growth factor)'' in [[Proteins]]
!Definition of molecule type in BNGL
{{{EGF(EGFL,deg~F~T,loc~Ex~En)}}}
!Summary
EGF (represented by {{{EGF}}} in the model) is a ligand of EGFR.  EGF is composed of an ~EGF-like (EGFL) domain (see [[Domains]]).  The EGFL domain (represented by {{{EGFL}}} in the model) is also called the EGF motif.  The EGFL domain consists of two cysteine-rich modules, C2 and C1.  Three disulfide (SS) bonds are present.  In the model, EGF is found in one of two compartments: the extracellular (Ex) compartment or the endocytic (En) compartment.  The component {{{loc}}} is introduced to track the location of EGF.  The internal state of {{{loc}}} ({{{Ex}}} or {{{En}}}) indicates the location of EGF. In the model, EGF is internalized when it is bound to EGFR and degraded after it is internalized.  The component {{{deg}}} is introduced to track EGF degradation. The internal state of {{{deg}}} indicates whether EGF is intact ({{{F}}}) or degraded ({{{T}}}).
!DOG
[img[DOGS/EGF.jpg]]
!Reading
*[[OMIM ID 131530 | http://www.ncbi.nlm.nih.gov/omim/131530]]
*Burgess AW, Cho HS, Eigenbrot C, Ferguson KM, Garrett TPJ, Leahy DJ, Lemmon MA, Sliwkowski MX, Ward CW, Yokoyama S (2003) An open-and-shut case? Recent insights into the activation of the EGF/~ErbB receptors. Mol Cell 12:541-552. [[PMID: 14527402 | http://www.ncbi.nlm.nih.gov/pubmed/14527402]]
```

```
''EGF reversibly binds EGFR'' (see Arrow 1 in [[Contact Map]])
{{{EGF(EGFL)+EGFR(I_III)<->EGF(EGFL!1).EGFR(I_III!1)}}}
!Rules
{{{
1 EGFR(I_III,II,loc~M) + EGF(EGFL,deg~F,loc~Ex) <-> \
 EGFR(I_III!1,II,loc~M).EGF(EGFL!1,deg~F,loc~Ex)     EGFkp1,EGFkm1

4 EGFR(I_III,II!1,loc~M).EGFR(I_III,II!1,loc~M) + EGF(EGFL,deg~F,loc~Ex) <-> \
 EGFR(I_III,II!1,loc~M).EGFR(I_III!2,II!1,loc~M).EGF(EGFL!2,deg~F,loc~Ex)     EGFkp2,EGFkm2

5 EGF(EGFL!2,deg~F,loc~Ex).EGFR(I_III!2,II!1,loc~M).EGFR(I_III,II!1,loc~M) + EGF(EGFL,deg~F,loc~Ex) <-> \
 EGF(EGFL!2,deg~F,loc~Ex).EGFR(I_III!2,II!1,loc~M).EGFR(I_III!3,II!1,loc~M).EGF(EGFL!3,deg~F,loc~Ex)     EGFkp3,EGFkm3

6 ErbB2(II!1,loc~M).EGFR(I_III,II!1,loc~M) + EGF(EGFL,deg~F,loc~Ex) <-> \
 ErbB2(II!1,loc~M).EGFR(I_III!2,II!1,loc~M).EGF(EGFL!2,deg~F,loc~Ex)     EGFkp2,EGFkm2

7 EGF(EGFL,deg~F,loc~Ex) + EGFR(I_III,II!1,loc~M).ErbB3(I_III,II!1,loc~M) <-> \
 EGF(EGFL!2,deg~F,loc~Ex).EGFR(I_III!2,II!1,loc~M).ErbB3(I_III,II!1,loc~M)      EGFkp2,EGFkm2

10 EGF(EGFL,deg~F,loc~Ex) + EGFR(I_III,II!1,loc~M).ErbB3(I_III!3,II!1,loc~M).HRG(EGFL!3,deg~F,loc~Ex) <-> \
 EGF(EGFL!2,deg~F,loc~Ex).EGFR(I_III!2,II!1,loc~M).ErbB3(I_III!3,II!1,loc~M).HRG(EGFL!3,deg~F,loc~Ex)     EGFkp3,EGFkm3

11 EGF(EGFL,deg~F,loc~Ex) + EGFR(I_III,II!1,loc~M).ErbB4(I_III,II!1,loc~M) <-> \
 EGF(EGFL!2,deg~F,loc~Ex).EGFR(I_III!2,II!1,loc~M).ErbB4(I_III,II!1,loc~M)      EGFkp2,EGFkm2

14 EGF(EGFL,deg~F,loc~Ex) + EGFR(I_III,II!1,loc~M).ErbB4(I_III!3,II!1,loc~M).HRG(EGFL!3,deg~F,loc~Ex) <-> \
 EGF(EGFL!2,deg~F,loc~Ex).EGFR(I_III!2,II!1,loc~M).ErbB4(I_III!3,II!1,loc~M).HRG(EGFL!3,deg~F,loc~Ex)     EGFkp3,EGFkm3
}}}
```

```
See ''EGFR (epidermal growth factor receptor)'' in [[Proteins]]
!Definition of molecule type in BNGL
{{{EGFR(I_III,II,T669~O~P,Y992~O~P,Y1068~O~P,Y1086~O~P,Y1114~O~P,Y1148~O~P,Y1173~O~P,loc~M~En)}}}
!Summary
EGFR (represented by {{{EGFR}}} in the model) is a receptor tyrosine kinase.  The ectodomain of EGFR is composed of four subdomains, which are called domains ~I-IV (see [[Domains]]).  In the model, the component {{{I_III}}} represents domains I and III, the ligand-binding (L) domains of EGFR.  These domains are also called L1 and L2.  The interaction of EGF with EGFR involves two points of contact, one between EGF and domain I of the EGFR ectodomain and the other between EGF and domain III of the EGFR ectodomain.  The component {{{II}}} represents domain II of the EGFR ectodomain, which mediates ligand-induced receptor dimerization via receptor-receptor interaction.  In the model, EGFR is found in one of two compartments: the plasma membrane (M) compartment or the endocytic (En) compartment.  The component {{{loc}}} is introduced to track the location of EGFR.  The internal state of {{{loc}}} ({{{M}}} or {{{En}}}) indicates the location of EGFR.  The other components of {{{EGFR}}}, namely {{{T669}}}, {{{Y992}}}, {{{Y1068}}}, {{{Y1086}}}, {{{Y1148}}}, and {{{Y1173}}}, represent sites of phosphorylation.  The kinase responsible for phosphorylation of T669 is ~ERK1/2.  Phosphorylation of T669 inhibits EGFR signaling. Residues Y992 and Y1068 are minor sites of autophosphorylation, and residues Y1086, Y1148 and Y1173 are major sites of autophosphorylation.  The autophosphorylation sites are taken to also be substrates of ~ErbB2.  Internal states are introduced to represent the unphosphorylated ({{{O}}}) and phosphorylated ({{{P}}}) forms of amino acid residues.  Phosphotyrosines in EGFR serve as docking sites for signaling proteins.
!DOG
[img[DOGS/EGFR.jpg]]
!Reading
*[[OMIM ID 131550 | http://www.ncbi.nlm.nih.gov/omim/131550]]
*Burgess AW, Cho HS, Eigenbrot C, Ferguson KM, Garrett TPJ, Leahy DJ, Lemmon MA, Sliwkowski MX, Ward CW, Yokoyama S (2003) An open-and-shut case? Recent insights into the activation of the EGF/~ErbB receptors. Mol Cell 12:541-552. [[PMID: 14527402 | http://www.ncbi.nlm.nih.gov/pubmed/14527402]]
*Jorissen RN, Walker F, Pouliot N, Garrett TPJ, Ward CW, Burgess AW (2003) Epidermal growth factor receptor: mechanisms of activation and signalling. Exp Cell Res 284:31-53. [[PMID: 12648464 | http://www.ncbi.nlm.nih.gov/pubmed/12648464]]
```

```
''EGFR catalyzes phosphorylation of EGFR, ~ErbB2, ~ErbB3, ~ErbB4, p52Shc1, and Gab1'' (see Arrow 7 in [[Contact Map]])
{{{EGFR(Y992~O)->EGFR(Y992~P)}}}
{{{EGFR(Y1068~O)->EGFR(Y1068~P)}}}
{{{EGFR(Y1086~O)->EGFR(Y1086~P)}}}
{{{EGFR(Y1148~O)->EGFR(Y1148~P)}}}
{{{EGFR(Y1173~O)->EGFR(Y1173~P)}}}
{{{ErbB2(Y1139~O)->ErbB2(Y1139~P)}}}
{{{ErbB2(Y1222~O)->ErbB2(Y1222~P)}}}
{{{ErbB3(Y1051~O)->ErbB3(Y1051~P)}}}
{{{ErbB3(Y1194~O)->ErbB3(Y1194~P)}}}
{{{ErbB3(Y1219~O)->ErbB3(Y1219~P)}}}
{{{ErbB3(Y1257~O)->ErbB3(Y1257~P)}}}
{{{ErbB3(Y1273~O)->ErbB3(Y1273~P)}}}
{{{ErbB3(Y1286~O)->ErbB3(Y1286~P)}}}
{{{ErbB3(Y1325~O)->ErbB3(Y1325~P)}}}
{{{ErbB4(Y1056~O)->ErbB4(Y1056~P)}}}
{{{ErbB4(Y1188~O)->ErbB4(Y1188~P)}}}
{{{ErbB4(Y1242~O)->ErbB4(Y1242~P)}}}
{{{p52Shc1(Y317~O)->p52Shc1(Y317~P)}}}
{{{Gab1(Y447~O)->Gab1(Y447~P)}}}
{{{Gab1(Y472~O)->Gab1(Y472~P)}}}
{{{Gab1(Y589~O)->Gab1(Y589~P)}}}
!Rules
{{{
100 EGF(EGFL!2).EGFR(I_III!2,II!1,T669~O).EGFR(I_III!3,II!1,Y992~O).EGF(EGFL!3) -> \
  EGF(EGFL!2).EGFR(I_III!2,II!1,T669~O).EGFR(I_III!3,II!1,Y992~P).EGF(EGFL!3) Phosphokp1

101 EGF(EGFL!2).EGFR(I_III!2,II!1,T669~O).EGFR(I_III!3,II!1,Y1068~O).EGF(EGFL!3) -> \
  EGF(EGFL!2).EGFR(I_III!2,II!1,T669~O).EGFR(I_III!3,II!1,Y1068~P).EGF(EGFL!3) Phosphokp2

102 EGF(EGFL!2).EGFR(I_III!2,II!1,T669~O).EGFR(I_III!3,II!1,Y1086~O).EGF(EGFL!3) -> \
  EGF(EGFL!2).EGFR(I_III!2,II!1,T669~O).EGFR(I_III!3,II!1,Y1086~P).EGF(EGFL!3) Phosphokp3

103 EGF(EGFL!2).EGFR(I_III!2,II!1,T669~O).EGFR(I_III!3,II!1,Y1114~O).EGF(EGFL!3) -> \
  EGF(EGFL!2).EGFR(I_III!2,II!1,T669~O).EGFR(I_III!3,II!1,Y1114~P).EGF(EGFL!3) Phosphokp4

104 EGF(EGFL!2).EGFR(I_III!2,II!1,T669~O).EGFR(I_III!3,II!1,Y1148~O).EGF(EGFL!3) -> \
  EGF(EGFL!2).EGFR(I_III!2,II!1,T669~O).EGFR(I_III!3,II!1,Y1148~P).EGF(EGFL!3) Phosphokp4

105 EGF(EGFL!2).EGFR(I_III!2,II!1,T669~O).EGFR(I_III!3,II!1,Y1173~O).EGF(EGFL!3) -> \
  EGF(EGFL!2).EGFR(I_III!2,II!1,T669~O).EGFR(I_III!3,II!1,Y1173~P).EGF(EGFL!3) Phosphokp5

118 EGF(EGFL!2).EGFR(I_III!2,II!1,T669~O).ErbB2(II!1,Y1139~O) -> \
  EGF(EGFL!2).EGFR(I_III!2,II!1,T669~O).ErbB2(II!1,Y1139~P) Phosphokp18

119 EGF(EGFL!2).EGFR(I_III!2,II!1,T669~O).ErbB2(II!1,Y1196~O) -> \
  EGF(EGFL!2).EGFR(I_III!2,II!1,T669~O).ErbB2(II!1,Y1196~P) Phosphokp20

120 EGF(EGFL!2).EGFR(I_III!2,II!1,T669~O).ErbB2(II!1,Y1222~O) -> \
  EGF(EGFL!2).EGFR(I_III!2,II!1,T669~O).ErbB2(II!1,Y1222~P) Phosphokp21

121 EGF(EGFL!2).EGFR(I_III!2,II!1,T669~O).ErbB2(II!1,Y1248~O) -> \
  EGF(EGFL!2).EGFR(I_III!2,II!1,T669~O).ErbB2(II!1,Y1248~P) Phosphokp22

126 EGF(EGFL!2).EGFR(I_III!2,II!1,T669~O).ErbB3(I_III!3,II!1,Y1054~O).HRG(EGFL!3) -> \
  EGF(EGFL!2).EGFR(I_III!2,II!1,T669~O).ErbB3(I_III!3,II!1,Y1054~P).HRG(EGFL!3) Phosphokp27

127 EGF(EGFL!2).EGFR(I_III!2,II!1,T669~O).ErbB3(I_III!3,II!1,Y1197~O).HRG(EGFL!3) -> \
  EGF(EGFL!2).EGFR(I_III!2,II!1,T669~O).ErbB3(I_III!3,II!1,Y1197~P).HRG(EGFL!3) Phosphokp28

128 EGF(EGFL!2).EGFR(I_III!2,II!1,T669~O).ErbB3(I_III!3,II!1,Y1222~O).HRG(EGFL!3) -> \
  EGF(EGFL!2).EGFR(I_III!2,II!1,T669~O).ErbB3(I_III!3,II!1,Y1222~P).HRG(EGFL!3) Phosphokp29

129 EGF(EGFL!2).EGFR(I_III!2,II!1,T669~O).ErbB3(I_III!3,II!1,Y1260~O).HRG(EGFL!3) -> \
  EGF(EGFL!2).EGFR(I_III!2,II!1,T669~O).ErbB3(I_III!3,II!1,Y1260~P).HRG(EGFL!3) Phosphokp30

130 EGF(EGFL!2).EGFR(I_III!2,II!1,T669~O).ErbB3(I_III!3,II!1,Y1276~O).HRG(EGFL!3) -> \
  EGF(EGFL!2).EGFR(I_III!2,II!1,T669~O).ErbB3(I_III!3,II!1,Y1276~P).HRG(EGFL!3) Phosphokp31

131 EGF(EGFL!2).EGFR(I_III!2,II!1,T669~O).ErbB3(I_III!3,II!1,Y1289~O).HRG(EGFL!3) -> \
  EGF(EGFL!2).EGFR(I_III!2,II!1,T669~O).ErbB3(I_III!3,II!1,Y1289~P).HRG(EGFL!3) Phosphokp32

132 EGF(EGFL!2).EGFR(I_III!2,II!1,T669~O).ErbB3(I_III!3,II!1,Y1328~O).HRG(EGFL!3) -> \
  EGF(EGFL!2).EGFR(I_III!2,II!1,T669~O).ErbB3(I_III!3,II!1,Y1328~P).HRG(EGFL!3) Phosphokp33

147 EGF(EGFL!2).EGFR(I_III!2,II!1,T669~O).ErbB4(I_III!3,II!1,Y1056~O).HRG(EGFL!3) -> \
  EGF(EGFL!2).EGFR(I_III!2,II!1,T669~O).ErbB4(I_III!3,II!1,Y1056~P).HRG(EGFL!3) Phosphokp48

148 EGF(EGFL!2).EGFR(I_III!2,II!1,T669~O).ErbB4(I_III!3,II!1,Y1188~O).HRG(EGFL!3) -> \
  EGF(EGFL!2).EGFR(I_III!2,II!1,T669~O).ErbB4(I_III!3,II!1,Y1188~P).HRG(EGFL!3) Phosphokp49

149 EGF(EGFL!2).EGFR(I_III!2,II!1,T669~O).ErbB4(I_III!3,II!1,Y1242~O).HRG(EGFL!3) -> \
  EGF(EGFL!2).EGFR(I_III!2,II!1,T669~O).ErbB4(I_III!3,II!1,Y1242~P).HRG(EGFL!3) Phosphokp50

190 EGF(EGFL!2).EGFR(I_III!2,II!1,T669~O).p52Shc1(Y317~O,PTB!4).EGFR(I_III!3,II!1,Y992~P!4).EGF(EGFL!3) -> \
 EGF(EGFL!2).EGFR(I_III!2,II!1,T669~O).p52Shc1(Y317~P,PTB!4).EGFR(I_III!3,II!1,Y992~P!4).EGF(EGFL!3)    Shc1kp9

191 EGF(EGFL!2).EGFR(I_III!2,II!1,T669~O).p52Shc1(Y317~O,PTB!4).EGFR(I_III!3,II!1,Y1086~P!4).EGF(EGFL!3) -> \
 EGF(EGFL!2).EGFR(I_III!2,II!1,T669~O).p52Shc1(Y317~P,PTB!4).EGFR(I_III!3,II!1,Y1086~P!4).EGF(EGFL!3)    Shc1kp10

192 EGF(EGFL!2).EGFR(I_III!2,II!1,T669~O).p52Shc1(Y317~O,PTB!4).EGFR(I_III!3,II!1,Y1114~P!4).EGF(EGFL!3) -> \
 EGF(EGFL!2).EGFR(I_III!2,II!1,T669~O).p52Shc1(Y317~P,PTB!4).EGFR(I_III!3,II!1,Y1114~P!4).EGF(EGFL!3)    Shc1kp11

193 EGF(EGFL!2).EGFR(I_III!2,II!1,T669~O).p52Shc1(Y317~O,PTB!4).ErbB2(II!1,Y1196~P!4) -> \
 EGF(EGFL!2).EGFR(I_III!2,II!1,T669~O).p52Shc1(Y317~P,PTB!4).ErbB2(II!1,Y1196~P!4)    Shc1kp12

194 EGF(EGFL!2).EGFR(I_III!2,II!1,T669~O).p52Shc1(Y317~O,PTB!4).ErbB2(II!1,Y1222~P!4) -> \
 EGF(EGFL!2).EGFR(I_III!2,II!1,T669~O).p52Shc1(Y317~P,PTB!4).ErbB2(II!1,Y1222~P!4)    Shc1kp13

195 EGF(EGFL!2).EGFR(I_III!2,II!1,T669~O).p52Shc1(Y317~O,PTB!4).ErbB2(II!1,Y1248~P!4) -> \
 EGF(EGFL!2).EGFR(I_III!2,II!1,T669~O).p52Shc1(Y317~P,PTB!4).ErbB2(II!1,Y1248~P!4)    Shc1kp14

196 EGF(EGFL!2).EGFR(I_III!2,II!1,T669~O).p52Shc1(Y317~O,PTB!4).ErbB3(I_III!3,II!1,Y1328~P!4).HRG(EGFL!3) -> \
 EGF(EGFL!2).EGFR(I_III!2,II!1,T669~O).p52Shc1(Y317~P,PTB!4).ErbB3(I_III!3,II!1,Y1328~P!4).HRG(EGFL!3)    Shc1kp15

197 EGF(EGFL!2).EGFR(I_III!2,II!1,T669~O).p52Shc1(Y317~O,PTB!4).ErbB4(I_III!3,II!1,Y1188~P!4).HRG(EGFL!3) -> \
 EGF(EGFL!2).EGFR(I_III!2,II!1,T669~O).p52Shc1(Y317~P,PTB!4).ErbB4(I_III!3,II!1,Y1188~P!4).HRG(EGFL!3)    Shc1kp16

198 EGF(EGFL!2).EGFR(I_III!2,II!1,T669~O).p52Shc1(Y317~O,PTB!4).ErbB4(I_III!3,II!1,Y1242~P!4).HRG(EGFL!3) -> \
 EGF(EGFL!2).EGFR(I_III!2,II!1,T669~O).p52Shc1(Y317~P,PTB!4).ErbB4(I_III!3,II!1,Y1242~P!4).HRG(EGFL!3)    Shc1kp17

303 EGF(EGFL!5).EGFR(I_III!5,II!1,T669~O).Gab1(Y447~O,PRS1_PRS2!6).Grb2(cSH3!6,SH2s!2).p52Shc1(Y317~P!2,PTB!4).EGFR(I_III!3,II!1,Y992~P!4).EGF(EGFL!3) -> \
 EGF(EGFL!5).EGFR(I_III!5,II!1,T669~O).Gab1(Y447~P,PRS1_PRS2!6).Grb2(cSH3!6,SH2s!2).p52Shc1(Y317~P!2,PTB!4).EGFR(I_III!3,II!1,Y992~P!4).EGF(EGFL!3)    Gab1kp3

304 EGF(EGFL!5).EGFR(I_III!5,II!1,T669~O).Gab1(Y472~O,PRS1_PRS2!6).Grb2(cSH3!6,SH2s!2).p52Shc1(Y317~P!2,PTB!4).EGFR(I_III!3,II!1,Y992~P!4).EGF(EGFL!3) -> \
 EGF(EGFL!5).EGFR(I_III!5,II!1,T669~O).Gab1(Y472~P,PRS1_PRS2!6).Grb2(cSH3!6,SH2s!2).p52Shc1(Y317~P!2,PTB!4).EGFR(I_III!3,II!1,Y992~P!4).EGF(EGFL!3)    Gab1kp4

305 EGF(EGFL!5).EGFR(I_III!5,II!1,T669~O).Gab1(Y619~O,PRS1_PRS2!6).Grb2(cSH3!6,SH2s!2).p52Shc1(Y317~P!2,PTB!4).EGFR(I_III!3,II!1,Y992~P!4).EGF(EGFL!3) -> \
 EGF(EGFL!5).EGFR(I_III!5,II!1,T669~O).Gab1(Y619~P,PRS1_PRS2!6).Grb2(cSH3!6,SH2s!2).p52Shc1(Y317~P!2,PTB!4).EGFR(I_III!3,II!1,Y992~P!4).EGF(EGFL!3)    Gab1kp5

306 EGF(EGFL!5).EGFR(I_III!5,II!1,T669~O).Gab1(Y657~O,PRS1_PRS2!6).Grb2(cSH3!6,SH2s!2).p52Shc1(Y317~P!2,PTB!4).EGFR(I_III!3,II!1,Y992~P!4).EGF(EGFL!3) -> \
 EGF(EGFL!5).EGFR(I_III!5,II!1,T669~O).Gab1(Y657~P,PRS1_PRS2!6).Grb2(cSH3!6,SH2s!2).p52Shc1(Y317~P!2,PTB!4).EGFR(I_III!3,II!1,Y992~P!4).EGF(EGFL!3)    Gab1kp6

307 EGF(EGFL!5).EGFR(I_III!5,II!1,T669~O).Gab1(Y447~O,PRS1_PRS2!6).Grb2(cSH3!6,SH2s!2).p52Shc1(Y317~P!2,PTB!4).EGFR(I_III!3,II!1,Y1086~P!4).EGF(EGFL!3) -> \
 EGF(EGFL!5).EGFR(I_III!5,II!1,T669~O).Gab1(Y447~P,PRS1_PRS2!6).Grb2(cSH3!6,SH2s!2).p52Shc1(Y317~P!2,PTB!4).EGFR(I_III!3,II!1,Y1086~P!4).EGF(EGFL!3)    Gab1kp7

308 EGF(EGFL!5).EGFR(I_III!5,II!1,T669~O).Gab1(Y472~O,PRS1_PRS2!6).Grb2(cSH3!6,SH2s!2).p52Shc1(Y317~P!2,PTB!4).EGFR(I_III!3,II!1,Y1086~P!4).EGF(EGFL!3) -> \
 EGF(EGFL!5).EGFR(I_III!5,II!1,T669~O).Gab1(Y472~P,PRS1_PRS2!6).Grb2(cSH3!6,SH2s!2).p52Shc1(Y317~P!2,PTB!4).EGFR(I_III!3,II!1,Y1086~P!4).EGF(EGFL!3)    Gab1kp8

309 EGF(EGFL!5).EGFR(I_III!5,II!1,T669~O).Gab1(Y619~O,PRS1_PRS2!6).Grb2(cSH3!6,SH2s!2).p52Shc1(Y317~P!2,PTB!4).EGFR(I_III!3,II!1,Y1086~P!4).EGF(EGFL!3) -> \
 EGF(EGFL!5).EGFR(I_III!5,II!1,T669~O).Gab1(Y619~P,PRS1_PRS2!6).Grb2(cSH3!6,SH2s!2).p52Shc1(Y317~P!2,PTB!4).EGFR(I_III!3,II!1,Y1086~P!4).EGF(EGFL!3)    Gab1kp9

310 EGF(EGFL!5).EGFR(I_III!5,II!1,T669~O).Gab1(Y657~O,PRS1_PRS2!6).Grb2(cSH3!6,SH2s!2).p52Shc1(Y317~P!2,PTB!4).EGFR(I_III!3,II!1,Y1086~P!4).EGF(EGFL!3) -> \
 EGF(EGFL!5).EGFR(I_III!5,II!1,T669~O).Gab1(Y657~P,PRS1_PRS2!6).Grb2(cSH3!6,SH2s!2).p52Shc1(Y317~P!2,PTB!4).EGFR(I_III!3,II!1,Y1086~P!4).EGF(EGFL!3)    Gab1kp10

311 EGF(EGFL!5).EGFR(I_III!5,II!1,T669~O).Gab1(Y447~O,PRS1_PRS2!6).Grb2(cSH3!6,SH2s!2).p52Shc1(Y317~P!2,PTB!4).EGFR(I_III!3,II!1,Y1114~P!4).EGF(EGFL!3) -> \
 EGF(EGFL!5).EGFR(I_III!5,II!1,T669~O).Gab1(Y447~P,PRS1_PRS2!6).Grb2(cSH3!6,SH2s!2).p52Shc1(Y317~P!2,PTB!4).EGFR(I_III!3,II!1,Y1114~P!4).EGF(EGFL!3)    Gab1kp11

312 EGF(EGFL!5).EGFR(I_III!5,II!1,T669~O).Gab1(Y472~O,PRS1_PRS2!6).Grb2(cSH3!6,SH2s!2).p52Shc1(Y317~P!2,PTB!4).EGFR(I_III!3,II!1,Y1114~P!4).EGF(EGFL!3) -> \
 EGF(EGFL!5).EGFR(I_III!5,II!1,T669~O).Gab1(Y472~P,PRS1_PRS2!6).Grb2(cSH3!6,SH2s!2).p52Shc1(Y317~P!2,PTB!4).EGFR(I_III!3,II!1,Y1114~P!4).EGF(EGFL!3)    Gab1kp12

313 EGF(EGFL!5).EGFR(I_III!5,II!1,T669~O).Gab1(Y619~O,PRS1_PRS2!6).Grb2(cSH3!6,SH2s!2).p52Shc1(Y317~P!2,PTB!4).EGFR(I_III!3,II!1,Y1114~P!4).EGF(EGFL!3) -> \
 EGF(EGFL!5).EGFR(I_III!5,II!1,T669~O).Gab1(Y619~P,PRS1_PRS2!6).Grb2(cSH3!6,SH2s!2).p52Shc1(Y317~P!2,PTB!4).EGFR(I_III!3,II!1,Y1114~P!4).EGF(EGFL!3)    Gab1kp13

314 EGF(EGFL!5).EGFR(I_III!5,II!1,T669~O).Gab1(Y657~O,PRS1_PRS2!6).Grb2(cSH3!6,SH2s!2).p52Shc1(Y317~P!2,PTB!4).EGFR(I_III!3,II!1,Y1114~P!4).EGF(EGFL!3) -> \
 EGF(EGFL!5).EGFR(I_III!5,II!1,T669~O).Gab1(Y657~P,PRS1_PRS2!6).Grb2(cSH3!6,SH2s!2).p52Shc1(Y317~P!2,PTB!4).EGFR(I_III!3,II!1,Y1114~P!4).EGF(EGFL!3)    Gab1kp14

315 EGF(EGFL!5).EGFR(I_III!5,II!1,T669~O).Gab1(Y447~O,PRS1_PRS2!6).Grb2(cSH3!6,SH2r!4).EGFR(I_III!3,II!1,Y1068~P!4).EGF(EGFL!3) -> \
 EGF(EGFL!5).EGFR(I_III!5,II!1,T669~O).Gab1(Y447~P,PRS1_PRS2!6).Grb2(cSH3!6,SH2r!4).EGFR(I_III!3,II!1,Y1068~P!4).EGF(EGFL!3)    Gab1kp15

316 EGF(EGFL!5).EGFR(I_III!5,II!1,T669~O).Gab1(Y472~O,PRS1_PRS2!6).Grb2(cSH3!6,SH2r!4).EGFR(I_III!3,II!1,Y1068~P!4).EGF(EGFL!3) -> \
 EGF(EGFL!5).EGFR(I_III!5,II!1,T669~O).Gab1(Y472~P,PRS1_PRS2!6).Grb2(cSH3!6,SH2r!4).EGFR(I_III!3,II!1,Y1068~P!4).EGF(EGFL!3)    Gab1kp16

317 EGF(EGFL!5).EGFR(I_III!5,II!1,T669~O).Gab1(Y619~O,PRS1_PRS2!6).Grb2(cSH3!6,SH2r!4).EGFR(I_III!3,II!1,Y1068~P!4).EGF(EGFL!3) -> \
 EGF(EGFL!5).EGFR(I_III!5,II!1,T669~O).Gab1(Y619~P,PRS1_PRS2!6).Grb2(cSH3!6,SH2r!4).EGFR(I_III!3,II!1,Y1068~P!4).EGF(EGFL!3)    Gab1kp17

318 EGF(EGFL!5).EGFR(I_III!5,II!1,T669~O).Gab1(Y657~O,PRS1_PRS2!6).Grb2(cSH3!6,SH2r!4).EGFR(I_III!3,II!1,Y1068~P!4).EGF(EGFL!3) -> \
 EGF(EGFL!5).EGFR(I_III!5,II!1,T669~O).Gab1(Y657~P,PRS1_PRS2!6).Grb2(cSH3!6,SH2r!4).EGFR(I_III!3,II!1,Y1068~P!4).EGF(EGFL!3)    Gab1kp18

319 EGF(EGFL!5).EGFR(I_III!5,II!1,T669~O).Gab1(Y447~O,PRS1_PRS2!6).Grb2(cSH3!6,SH2r!4).EGFR(I_III!3,II!1,Y1114~P!4).EGF(EGFL!3) -> \
 EGF(EGFL!5).EGFR(I_III!5,II!1,T669~O).Gab1(Y447~P,PRS1_PRS2!6).Grb2(cSH3!6,SH2r!4).EGFR(I_III!3,II!1,Y1114~P!4).EGF(EGFL!3)    Gab1kp19

320 EGF(EGFL!5).EGFR(I_III!5,II!1,T669~O).Gab1(Y472~O,PRS1_PRS2!6).Grb2(cSH3!6,SH2r!4).EGFR(I_III!3,II!1,Y1114~P!4).EGF(EGFL!3) -> \
 EGF(EGFL!5).EGFR(I_III!5,II!1,T669~O).Gab1(Y472~P,PRS1_PRS2!6).Grb2(cSH3!6,SH2r!4).EGFR(I_III!3,II!1,Y1114~P!4).EGF(EGFL!3)    Gab1kp20

321 EGF(EGFL!5).EGFR(I_III!5,II!1,T669~O).Gab1(Y619~O,PRS1_PRS2!6).Grb2(cSH3!6,SH2r!4).EGFR(I_III!3,II!1,Y1114~P!4).EGF(EGFL!3) -> \
 EGF(EGFL!5).EGFR(I_III!5,II!1,T669~O).Gab1(Y619~P,PRS1_PRS2!6).Grb2(cSH3!6,SH2r!4).EGFR(I_III!3,II!1,Y1114~P!4).EGF(EGFL!3)    Gab1kp21

322 EGF(EGFL!5).EGFR(I_III!5,II!1,T669~O).Gab1(Y657~O,PRS1_PRS2!6).Grb2(cSH3!6,SH2r!4).EGFR(I_III!3,II!1,Y1114~P!4).EGF(EGFL!3) -> \
 EGF(EGFL!5).EGFR(I_III!5,II!1,T669~O).Gab1(Y657~P,PRS1_PRS2!6).Grb2(cSH3!6,SH2r!4).EGFR(I_III!3,II!1,Y1114~P!4).EGF(EGFL!3)    Gab1kp22

323 EGF(EGFL!5).EGFR(I_III!5,II!1,T669~O).Gab1(Y447~O,PRS1_PRS2!6).Grb2(cSH3!6,SH2r!4).EGFR(I_III!3,II!1,Y1148~P!4).EGF(EGFL!3) -> \
 EGF(EGFL!5).EGFR(I_III!5,II!1,T669~O).Gab1(Y447~P,PRS1_PRS2!6).Grb2(cSH3!6,SH2r!4).EGFR(I_III!3,II!1,Y1148~P!4).EGF(EGFL!3)    Gab1kp23

324 EGF(EGFL!5).EGFR(I_III!5,II!1,T669~O).Gab1(Y472~O,PRS1_PRS2!6).Grb2(cSH3!6,SH2r!4).EGFR(I_III!3,II!1,Y1148~P!4).EGF(EGFL!3) -> \
 EGF(EGFL!5).EGFR(I_III!5,II!1,T669~O).Gab1(Y472~P,PRS1_PRS2!6).Grb2(cSH3!6,SH2r!4).EGFR(I_III!3,II!1,Y1148~P!4).EGF(EGFL!3)    Gab1kp24

325 EGF(EGFL!5).EGFR(I_III!5,II!1,T669~O).Gab1(Y619~O,PRS1_PRS2!6).Grb2(cSH3!6,SH2r!4).EGFR(I_III!3,II!1,Y1148~P!4).EGF(EGFL!3) -> \
 EGF(EGFL!5).EGFR(I_III!5,II!1,T669~O).Gab1(Y619~P,PRS1_PRS2!6).Grb2(cSH3!6,SH2r!4).EGFR(I_III!3,II!1,Y1148~P!4).EGF(EGFL!3)    Gab1kp25

326 EGF(EGFL!5).EGFR(I_III!5,II!1,T669~O).Gab1(Y657~O,PRS1_PRS2!6).Grb2(cSH3!6,SH2r!4).EGFR(I_III!3,II!1,Y1148~P!4).EGF(EGFL!3) -> \
 EGF(EGFL!5).EGFR(I_III!5,II!1,T669~O).Gab1(Y657~P,PRS1_PRS2!6).Grb2(cSH3!6,SH2r!4).EGFR(I_III!3,II!1,Y1148~P!4).EGF(EGFL!3)    Gab1kp26

327 EGF(EGFL!5).EGFR(I_III!5,II!1,T669~O).Gab1(Y447~O,PRS1_PRS2!6).Grb2(cSH3!6,SH2r!4).EGFR(I_III!3,II!1,Y1173~P!4).EGF(EGFL!3) -> \
 EGF(EGFL!5).EGFR(I_III!5,II!1,T669~O).Gab1(Y447~P,PRS1_PRS2!6).Grb2(cSH3!6,SH2r!4).EGFR(I_III!3,II!1,Y1173~P!4).EGF(EGFL!3)    Gab1kp27

328 EGF(EGFL!5).EGFR(I_III!5,II!1,T669~O).Gab1(Y472~O,PRS1_PRS2!6).Grb2(cSH3!6,SH2r!4).EGFR(I_III!3,II!1,Y1173~P!4).EGF(EGFL!3) -> \
 EGF(EGFL!5).EGFR(I_III!5,II!1,T669~O).Gab1(Y472~P,PRS1_PRS2!6).Grb2(cSH3!6,SH2r!4).EGFR(I_III!3,II!1,Y1173~P!4).EGF(EGFL!3)    Gab1kp28

329 EGF(EGFL!5).EGFR(I_III!5,II!1,T669~O).Gab1(Y619~O,PRS1_PRS2!6).Grb2(cSH3!6,SH2r!4).EGFR(I_III!3,II!1,Y1173~P!4).EGF(EGFL!3) -> \
 EGF(EGFL!5).EGFR(I_III!5,II!1,T669~O).Gab1(Y619~P,PRS1_PRS2!6).Grb2(cSH3!6,SH2r!4).EGFR(I_III!3,II!1,Y1173~P!4).EGF(EGFL!3)    Gab1kp29

330 EGF(EGFL!5).EGFR(I_III!5,II!1,T669~O).Gab1(Y657~O,PRS1_PRS2!6).Grb2(cSH3!6,SH2r!4).EGFR(I_III!3,II!1,Y1173~P!4).EGF(EGFL!3) -> \
 EGF(EGFL!5).EGFR(I_III!5,II!1,T669~O).Gab1(Y657~P,PRS1_PRS2!6).Grb2(cSH3!6,SH2r!4).EGFR(I_III!3,II!1,Y1173~P!4).EGF(EGFL!3)    Gab1kp30

331 EGF(EGFL!5).EGFR(I_III!5,II!1,T669~O).Gab1(Y447~O,PRS1_PRS2!6).Grb2(cSH3!6,SH2s!2).p52Shc1(Y317~P!2,PTB!4).ErbB2(II!1,Y1196~P!4) -> \
 EGF(EGFL!5).EGFR(I_III!5,II!1,T669~O).Gab1(Y447~P,PRS1_PRS2!6).Grb2(cSH3!6,SH2s!2).p52Shc1(Y317~P!2,PTB!4).ErbB2(II!1,Y1196~P!4)    Gab1kp31

332 EGF(EGFL!5).EGFR(I_III!5,II!1,T669~O).Gab1(Y472~O,PRS1_PRS2!6).Grb2(cSH3!6,SH2s!2).p52Shc1(Y317~P!2,PTB!4).ErbB2(II!1,Y1196~P!4) -> \
 EGF(EGFL!5).EGFR(I_III!5,II!1,T669~O).Gab1(Y472~P,PRS1_PRS2!6).Grb2(cSH3!6,SH2s!2).p52Shc1(Y317~P!2,PTB!4).ErbB2(II!1,Y1196~P!4)    Gab1kp32

333 EGF(EGFL!5).EGFR(I_III!5,II!1,T669~O).Gab1(Y619~O,PRS1_PRS2!6).Grb2(cSH3!6,SH2s!2).p52Shc1(Y317~P!2,PTB!4).ErbB2(II!1,Y1196~P!4) -> \
 EGF(EGFL!5).EGFR(I_III!5,II!1,T669~O).Gab1(Y619~P,PRS1_PRS2!6).Grb2(cSH3!6,SH2s!2).p52Shc1(Y317~P!2,PTB!4).ErbB2(II!1,Y1196~P!4)    Gab1kp33

334 EGF(EGFL!5).EGFR(I_III!5,II!1,T669~O).Gab1(Y657~O,PRS1_PRS2!6).Grb2(cSH3!6,SH2s!2).p52Shc1(Y317~P!2,PTB!4).ErbB2(II!1,Y1196~P!4) -> \
 EGF(EGFL!5).EGFR(I_III!5,II!1,T669~O).Gab1(Y657~P,PRS1_PRS2!6).Grb2(cSH3!6,SH2s!2).p52Shc1(Y317~P!2,PTB!4).ErbB2(II!1,Y1196~P!4)    Gab1kp34

335 EGF(EGFL!5).EGFR(I_III!5,II!1,T669~O).Gab1(Y447~O,PRS1_PRS2!6).Grb2(cSH3!6,SH2s!2).p52Shc1(Y317~P!2,PTB!4).ErbB2(II!1,Y1222~P!4) -> \
 EGF(EGFL!5).EGFR(I_III!5,II!1,T669~O).Gab1(Y447~P,PRS1_PRS2!6).Grb2(cSH3!6,SH2s!2).p52Shc1(Y317~P!2,PTB!4).ErbB2(II!1,Y1222~P!4)    Gab1kp35

336 EGF(EGFL!5).EGFR(I_III!5,II!1,T669~O).Gab1(Y472~O,PRS1_PRS2!6).Grb2(cSH3!6,SH2s!2).p52Shc1(Y317~P!2,PTB!4).ErbB2(II!1,Y1222~P!4) -> \
 EGF(EGFL!5).EGFR(I_III!5,II!1,T669~O).Gab1(Y472~P,PRS1_PRS2!6).Grb2(cSH3!6,SH2s!2).p52Shc1(Y317~P!2,PTB!4).ErbB2(II!1,Y1222~P!4)    Gab1kp36

337 EGF(EGFL!5).EGFR(I_III!5,II!1,T669~O).Gab1(Y619~O,PRS1_PRS2!6).Grb2(cSH3!6,SH2s!2).p52Shc1(Y317~P!2,PTB!4).ErbB2(II!1,Y1222~P!4) -> \
 EGF(EGFL!5).EGFR(I_III!5,II!1,T669~O).Gab1(Y619~P,PRS1_PRS2!6).Grb2(cSH3!6,SH2s!2).p52Shc1(Y317~P!2,PTB!4).ErbB2(II!1,Y1222~P!4)    Gab1kp37

338 EGF(EGFL!5).EGFR(I_III!5,II!1,T669~O).Gab1(Y657~O,PRS1_PRS2!6).Grb2(cSH3!6,SH2s!2).p52Shc1(Y317~P!2,PTB!4).ErbB2(II!1,Y1222~P!4) -> \
 EGF(EGFL!5).EGFR(I_III!5,II!1,T669~O).Gab1(Y657~P,PRS1_PRS2!6).Grb2(cSH3!6,SH2s!2).p52Shc1(Y317~P!2,PTB!4).ErbB2(II!1,Y1222~P!4)    Gab1kp38

339 EGF(EGFL!5).EGFR(I_III!5,II!1,T669~O).Gab1(Y447~O,PRS1_PRS2!6).Grb2(cSH3!6,SH2s!2).p52Shc1(Y317~P!2,PTB!4).ErbB2(II!1,Y1248~P!4) -> \
 EGF(EGFL!5).EGFR(I_III!5,II!1,T669~O).Gab1(Y447~P,PRS1_PRS2!6).Grb2(cSH3!6,SH2s!2).p52Shc1(Y317~P!2,PTB!4).ErbB2(II!1,Y1248~P!4)    Gab1kp39

340 EGF(EGFL!5).EGFR(I_III!5,II!1,T669~O).Gab1(Y472~O,PRS1_PRS2!6).Grb2(cSH3!6,SH2s!2).p52Shc1(Y317~P!2,PTB!4).ErbB2(II!1,Y1248~P!4) -> \
 EGF(EGFL!5).EGFR(I_III!5,II!1,T669~O).Gab1(Y472~P,PRS1_PRS2!6).Grb2(cSH3!6,SH2s!2).p52Shc1(Y317~P!2,PTB!4).ErbB2(II!1,Y1248~P!4)    Gab1kp40

341 EGF(EGFL!5).EGFR(I_III!5,II!1,T669~O).Gab1(Y619~O,PRS1_PRS2!6).Grb2(cSH3!6,SH2s!2).p52Shc1(Y317~P!2,PTB!4).ErbB2(II!1,Y1248~P!4) -> \
 EGF(EGFL!5).EGFR(I_III!5,II!1,T669~O).Gab1(Y619~P,PRS1_PRS2!6).Grb2(cSH3!6,SH2s!2).p52Shc1(Y317~P!2,PTB!4).ErbB2(II!1,Y1248~P!4)    Gab1kp41

342 EGF(EGFL!5).EGFR(I_III!5,II!1,T669~O).Gab1(Y657~O,PRS1_PRS2!6).Grb2(cSH3!6,SH2s!2).p52Shc1(Y317~P!2,PTB!4).ErbB2(II!1,Y1248~P!4) -> \
 EGF(EGFL!5).EGFR(I_III!5,II!1,T669~O).Gab1(Y657~P,PRS1_PRS2!6).Grb2(cSH3!6,SH2s!2).p52Shc1(Y317~P!2,PTB!4).ErbB2(II!1,Y1248~P!4)    Gab1kp42

343 EGF(EGFL!5).EGFR(I_III!5,II!1,T669~O).Gab1(Y447~O,PRS1_PRS2!6).Grb2(cSH3!6,SH2r!4).ErbB2(II!1,Y1139~P!4) -> \
 EGF(EGFL!5).EGFR(I_III!5,II!1,T669~O).Gab1(Y447~P,PRS1_PRS2!6).Grb2(cSH3!6,SH2r!4).ErbB2(II!1,Y1139~P!4)    Gab1kp43

344 EGF(EGFL!5).EGFR(I_III!5,II!1,T669~O).Gab1(Y472~O,PRS1_PRS2!6).Grb2(cSH3!6,SH2r!4).ErbB2(II!1,Y1139~P!4) -> \
 EGF(EGFL!5).EGFR(I_III!5,II!1,T669~O).Gab1(Y472~P,PRS1_PRS2!6).Grb2(cSH3!6,SH2r!4).ErbB2(II!1,Y1139~P!4)    Gab1kp44

345 EGF(EGFL!5).EGFR(I_III!5,II!1,T669~O).Gab1(Y619~O,PRS1_PRS2!6).Grb2(cSH3!6,SH2r!4).ErbB2(II!1,Y1139~P!4) -> \
 EGF(EGFL!5).EGFR(I_III!5,II!1,T669~O).Gab1(Y619~P,PRS1_PRS2!6).Grb2(cSH3!6,SH2r!4).ErbB2(II!1,Y1139~P!4)    Gab1kp45

346 EGF(EGFL!5).EGFR(I_III!5,II!1,T669~O).Gab1(Y657~O,PRS1_PRS2!6).Grb2(cSH3!6,SH2r!4).ErbB2(II!1,Y1139~P!4) -> \
 EGF(EGFL!5).EGFR(I_III!5,II!1,T669~O).Gab1(Y657~P,PRS1_PRS2!6).Grb2(cSH3!6,SH2r!4).ErbB2(II!1,Y1139~P!4)    Gab1kp46

347 EGF(EGFL!5).EGFR(I_III!5,II!1,T669~O).Gab1(Y447~O,PRS1_PRS2!6).Grb2(cSH3!6,SH2s!2).p52Shc1(Y317~P!2,PTB!4).ErbB3(I_III!3,II!1,Y1328~P!4).HRG(EGFL!3) -> \
 EGF(EGFL!5).EGFR(I_III!5,II!1,T669~O).Gab1(Y447~P,PRS1_PRS2!6).Grb2(cSH3!6,SH2s!2).p52Shc1(Y317~P!2,PTB!4).ErbB3(I_III!3,II!1,Y1328~P!4).HRG(EGFL!3)    Gab1kp47

348 EGF(EGFL!5).EGFR(I_III!5,II!1,T669~O).Gab1(Y472~O,PRS1_PRS2!6).Grb2(cSH3!6,SH2s!2).p52Shc1(Y317~P!2,PTB!4).ErbB3(I_III!3,II!1,Y1328~P!4).HRG(EGFL!3) -> \
 EGF(EGFL!5).EGFR(I_III!5,II!1,T669~O).Gab1(Y472~P,PRS1_PRS2!6).Grb2(cSH3!6,SH2s!2).p52Shc1(Y317~P!2,PTB!4).ErbB3(I_III!3,II!1,Y1328~P!4).HRG(EGFL!3)	Gab1kp48

349 EGF(EGFL!5).EGFR(I_III!5,II!1,T669~O).Gab1(Y619~O,PRS1_PRS2!6).Grb2(cSH3!6,SH2s!2).p52Shc1(Y317~P!2,PTB!4).ErbB3(I_III!3,II!1,Y1328~P!4).HRG(EGFL!3) -> \
 EGF(EGFL!5).EGFR(I_III!5,II!1,T669~O).Gab1(Y619~P,PRS1_PRS2!6).Grb2(cSH3!6,SH2s!2).p52Shc1(Y317~P!2,PTB!4).ErbB3(I_III!3,II!1,Y1328~P!4).HRG(EGFL!3)    Gab1kp49

350 EGF(EGFL!5).EGFR(I_III!5,II!1,T669~O).Gab1(Y657~O,PRS1_PRS2!6).Grb2(cSH3!6,SH2s!2).p52Shc1(Y317~P!2,PTB!4).ErbB3(I_III!3,II!1,Y1328~P!4).HRG(EGFL!3) -> \
 EGF(EGFL!5).EGFR(I_III!5,II!1,T669~O).Gab1(Y657~P,PRS1_PRS2!6).Grb2(cSH3!6,SH2s!2).p52Shc1(Y317~P!2,PTB!4).ErbB3(I_III!3,II!1,Y1328~P!4).HRG(EGFL!3)    Gab1kp50

351 EGF(EGFL!5).EGFR(I_III!5,II!1,T669~O).Gab1(Y447~O,PRS1_PRS2!6).Grb2(cSH3!6,SH2s!2).p52Shc1(Y317~P!2,PTB!4).ErbB4(I_III!3,II!1,Y1188~P!4).HRG(EGFL!3) -> \
 EGF(EGFL!5).EGFR(I_III!5,II!1,T669~O).Gab1(Y447~P,PRS1_PRS2!6).Grb2(cSH3!6,SH2s!2).p52Shc1(Y317~P!2,PTB!4).ErbB4(I_III!3,II!1,Y1188~P!4).HRG(EGFL!3)    Gab1kp51

352 EGF(EGFL!5).EGFR(I_III!5,II!1,T669~O).Gab1(Y472~O,PRS1_PRS2!6).Grb2(cSH3!6,SH2s!2).p52Shc1(Y317~P!2,PTB!4).ErbB4(I_III!3,II!1,Y1188~P!4).HRG(EGFL!3) -> \
 EGF(EGFL!5).EGFR(I_III!5,II!1,T669~O).Gab1(Y472~P,PRS1_PRS2!6).Grb2(cSH3!6,SH2s!2).p52Shc1(Y317~P!2,PTB!4).ErbB4(I_III!3,II!1,Y1188~P!4).HRG(EGFL!3)	Gab1kp52

353 EGF(EGFL!5).EGFR(I_III!5,II!1,T669~O).Gab1(Y619~O,PRS1_PRS2!6).Grb2(cSH3!6,SH2s!2).p52Shc1(Y317~P!2,PTB!4).ErbB4(I_III!3,II!1,Y1188~P!4).HRG(EGFL!3) -> \
 EGF(EGFL!5).EGFR(I_III!5,II!1,T669~O).Gab1(Y619~P,PRS1_PRS2!6).Grb2(cSH3!6,SH2s!2).p52Shc1(Y317~P!2,PTB!4).ErbB4(I_III!3,II!1,Y1188~P!4).HRG(EGFL!3)    Gab1kp53

354 EGF(EGFL!5).EGFR(I_III!5,II!1,T669~O).Gab1(Y657~O,PRS1_PRS2!6).Grb2(cSH3!6,SH2s!2).p52Shc1(Y317~P!2,PTB!4).ErbB4(I_III!3,II!1,Y1188~P!4).HRG(EGFL!3) -> \
 EGF(EGFL!5).EGFR(I_III!5,II!1,T669~O).Gab1(Y657~P,PRS1_PRS2!6).Grb2(cSH3!6,SH2s!2).p52Shc1(Y317~P!2,PTB!4).ErbB4(I_III!3,II!1,Y1188~P!4).HRG(EGFL!3)    Gab1kp54

355 EGF(EGFL!5).EGFR(I_III!5,II!1,T669~O).Gab1(Y447~O,PRS1_PRS2!6).Grb2(cSH3!6,SH2s!2).p52Shc1(Y317~P!2,PTB!4).ErbB4(I_III!3,II!1,Y1242~P!4).HRG(EGFL!3) -> \
 EGF(EGFL!5).EGFR(I_III!5,II!1,T669~O).Gab1(Y447~P,PRS1_PRS2!6).Grb2(cSH3!6,SH2s!2).p52Shc1(Y317~P!2,PTB!4).ErbB4(I_III!3,II!1,Y1242~P!4).HRG(EGFL!3)    Gab1kp55

356 EGF(EGFL!5).EGFR(I_III!5,II!1,T669~O).Gab1(Y472~O,PRS1_PRS2!6).Grb2(cSH3!6,SH2s!2).p52Shc1(Y317~P!2,PTB!4).ErbB4(I_III!3,II!1,Y1242~P!4).HRG(EGFL!3) -> \
 EGF(EGFL!5).EGFR(I_III!5,II!1,T669~O).Gab1(Y472~P,PRS1_PRS2!6).Grb2(cSH3!6,SH2s!2).p52Shc1(Y317~P!2,PTB!4).ErbB4(I_III!3,II!1,Y1242~P!4).HRG(EGFL!3)	Gab1kp56

357 EGF(EGFL!5).EGFR(I_III!5,II!1,T669~O).Gab1(Y619~O,PRS1_PRS2!6).Grb2(cSH3!6,SH2s!2).p52Shc1(Y317~P!2,PTB!4).ErbB4(I_III!3,II!1,Y1242~P!4).HRG(EGFL!3) -> \
 EGF(EGFL!5).EGFR(I_III!5,II!1,T669~O).Gab1(Y619~P,PRS1_PRS2!6).Grb2(cSH3!6,SH2s!2).p52Shc1(Y317~P!2,PTB!4).ErbB4(I_III!3,II!1,Y1242~P!4).HRG(EGFL!3)    Gab1kp57

358 EGF(EGFL!5).EGFR(I_III!5,II!1,T669~O).Gab1(Y657~O,PRS1_PRS2!6).Grb2(cSH3!6,SH2s!2).p52Shc1(Y317~P!2,PTB!4).ErbB4(I_III!3,II!1,Y1242~P!4).HRG(EGFL!3) -> \
 EGF(EGFL!5).EGFR(I_III!5,II!1,T669~O).Gab1(Y657~P,PRS1_PRS2!6).Grb2(cSH3!6,SH2s!2).p52Shc1(Y317~P!2,PTB!4).ErbB4(I_III!3,II!1,Y1242~P!4).HRG(EGFL!3)    Gab1kp58
}}}
```

```
''EGFR homodimerization'' (see Arrow 3 in [[Contact Map]])
{{{EGFR(II)+EGFR(II)<->EGFR(II!1).EGFR(II!1)}}}
!Rules
{{{
25 EGFR(I_III,II,loc~M) + EGFR(I_III,II,loc~M) <-> \
 EGFR(I_III,II!1,loc~M).EGFR(I_III,II!1,loc~M)       Dimkp1,Dimkm1

26 EGFR(I_III!1,II,loc~M).EGF(EGFL!1,loc~Ex) + EGFR(I_III,II,loc~M) <-> \
 EGFR(I_III!1,II!2,loc~M).EGF(EGFL!1,loc~Ex).EGFR(I_III,II!2,loc~M)     Dimkp2,Dimkm2

27 EGFR(I_III!1,II,loc~M).EGF(EGFL!1,loc~Ex) + EGFR(I_III!2,II,loc~M).EGF(EGFL!2,loc~Ex) <-> \
 EGFR(I_III!1,II!3,loc~M).EGF(EGFL!1,loc~Ex).EGFR(I_III!2,II!3,loc~M).EGF(EGFL!2,loc~Ex)   Dimkp3,Dimkm3
}}}
```

```
''EGFR reversibly binds ~ErbB2'' (see Arrows 3 and 4 in [[Contact Map]])
{{{EGFR(II)+ErbB2(II)<->EGFR(II!1).ErbB2(II!1)}}}
!Rules
{{{
28 EGFR(I_III,II,loc~M) + ErbB2(II,loc~M) <-> \
 EGFR(I_III,II!1,loc~M).ErbB2(II!1,loc~M)       Dimkp4,Dimkm4

29 EGFR(I_III!1,II,loc~M).EGF(EGFL!1,loc~Ex) + ErbB2(II,loc~M) <-> \
 EGFR(I_III!1,II!2,loc~M).EGF(EGFL!1,loc~Ex).ErbB2(II!2,loc~M)     Dimkp5,Dimkm5
}}}
```

```
''EGFR reversibly binds ~ErbB3'' (see Arrows 3 and 5 in [[Contact Map]])
{{{EGFR(II)+ErbB3(II)<->EGFR(II!1).ErbB3(II!1)}}}
!Rules
{{{
30 EGFR(I_III,II,loc~M) + ErbB3(I_III,II,loc~M) <-> \
 EGFR(I_III,II!1,loc~M).ErbB3(I_III,II!1,loc~M)       Dimkp6,Dimkm6

31 EGFR(I_III!1,II,loc~M).EGF(EGFL!1,loc~Ex) + ErbB3(I_III,II,loc~M) <-> \
 EGFR(I_III!1,II!2,loc~M).EGF(EGFL!1,loc~Ex).ErbB3(I_III,II!2,loc~M)     Dimkp7,Dimkm7

32 ErbB3(I_III!1,II,loc~M).HRG(EGFL!1,loc~Ex) + EGFR(I_III,II,loc~M) <-> \
 ErbB3(I_III!1,II!2,loc~M).HRG(EGFL!1,loc~Ex).EGFR(I_III,II!2,loc~M)     Dimkp8,Dimkm8

33 EGFR(I_III!1,II,loc~M).EGF(EGFL!1,loc~Ex) + ErbB3(I_III!2,II,loc~M).HRG(EGFL!2,loc~Ex) <-> \
 EGFR(I_III!1,II!3,loc~M).EGF(EGFL!1,loc~Ex).ErbB3(I_III!2,II!3,loc~M).HRG(EGFL!2,loc~Ex)   Dimkp9,Dimkm9
}}}
```

```
''EGFR reversibly binds ~ErbB4'' (see Arrow 6 in [[Contact Map]])
{{{EGFR(II)+ErbB4(II)<->EGFR(II!1).ErbB4(II!1)}}}
!Rules
{{{
34 EGFR(I_III,II,loc~M) + ErbB4(I_III,II,loc~M) <-> \
 EGFR(I_III,II!1,loc~M).ErbB4(I_III,II!1,loc~M)       Dimkp10,Dimkm10

35 EGFR(I_III!1,II,loc~M).EGF(EGFL!1,loc~Ex) + ErbB4(I_III,II,loc~M) <-> \
 EGFR(I_III!1,II!2,loc~M).EGF(EGFL!1,loc~Ex).ErbB4(I_III,II!2,loc~M)     Dimkp11,Dimkm11

36 ErbB4(I_III!1,II,loc~M).HRG(EGFL!1,loc~Ex) + EGFR(I_III,II,loc~M) <-> \
 ErbB4(I_III!1,II!2,loc~M).HRG(EGFL!1,loc~Ex).EGFR(I_III,II!2,loc~M)     Dimkp12,Dimkm12

37 EGFR(I_III!1,II,loc~M).EGF(EGFL!1,loc~Ex) + ErbB4(I_III!2,II,loc~M).HRG(EGFL!2,loc~Ex) <-> \
 EGFR(I_III!1,II!3,loc~M).EGF(EGFL!1,loc~Ex).ErbB4(I_III!2,II!3,loc~M).HRG(EGFL!2,loc~Ex)   Dimkp13,Dimkm13
}}}
```

```
''p120RasGAP reversibly binds EGFR'' (see Arrow 10 in [[Contact Map]])
{{{p120RasGAP(nSH2)+EGFR(Y992~P)<->p120RasGAP(nSH2!1).EGFR(Y992~P!1)}}}
!Rules
{{{
541 p120RasGAP(nSH2) + EGFR(Y992~P) <-> \
 p120RasGAP(nSH2!1).EGFR(Y992~P!1)	rGAPkp1,rGAPkm1
}}}
```

```
See ''~ERK2 (extracellular signal-regulated kinase 2)'' in [[Proteins]]
!BNGL
{{{ERK2(CD,STkinase,T185~O~P,Y187~O~P,loc~C)}}}
!Summary
~ERK2 is a MAP kinase. The residues T185 and Y187 are part of a ~TxY motif that is characteristic of MAP kinases.  The corresponding residues in ~ERK1 are T202 and Y204. The C-terminal region of ~ERK2 outside of the kinase domain contains a so-called CD domain (Tanoue et al., 2000), which is actually a linear motif.  The CD domain, which begins at L313 and ends at E326, is responsible for interaction with D or ? domain-containing proteins (Sharrocks et al., 2000).
!DOG
[img[DOGS/ERK2.jpg]]
!Reading
*[[OMIM ID 176948 | http://www.ncbi.nlm.nih.gov/omim/176948 ]]
*Roberts PJ, Der CJ (2007) Targeting the ~Raf-MEK-ERK mitogen-activated protein kinase cascade for the treatment of cancer. Oncogene 26:3291-3310. [[PMID: 17496923 | http://www.ncbi.nlm.nih.gov/pubmed/17496923]]
*Sharrocks AD, Yang SH, Galanis A (2000) Docking domains and substrate-specificity determination for MAP kinases. Trends Biochem Sci 25:448-453. [[PMID: 10973059 | http://www.ncbi.nlm.nih.gov/pubmed/10973059]]
*Tanoue T, Adachi M, Moriguchi T, Nishida E (2000) A conserved docking motif in MAP kinases common to substrates, activators and regulators. Nat Cell Biol 2:110-116. [[PMID: 10655591 | http://www.ncbi.nlm.nih.gov/pubmed/10655591]]
```

```
''~ERK2 catalyzes phosphorylation of EGFR, Raf1, ~MEK1, Gab1, and Sos1'' (see Arrow 26 in [[Contact Map]])
{{{EGFR(T669~O)->EGFR(T669~P)}}}
{{{Raf1(S29~O)->Raf1(S29~P)}}}
{{{Raf1(S43~O)->Raf1(S43~P)}}}
{{{Raf1(S289~O)->Raf1(S289~P)}}}
{{{Raf1(S301~O)->Raf1(S301~P)}}}
{{{Raf1(S471~O)->Raf1(S471~P)}}}
{{{Raf1(S642~O)->Raf1(S642~P)}}}
{{{MEK1(T292~O)->MEK1(T292~P)}}}
{{{Gab1(T312~O)->Gab1(T312~P)}}}
{{{Gab1(S381~O)->Gab1(S381~P)}}}
{{{Gab1(S454~O)->Gab1(S454~P)}}}
{{{Gab1(T476~O)->Gab1(T476~P)}}}
{{{Gab1(S552~O)->Gab1(S552~P)}}}
{{{Gab1(S551~O)->Gab1(S551~P)}}}
{{{Gab1(S597~O)->Gab1(S597~P)}}}
{{{Sos1(S1132~O)->Sos1(S1132~P)}}}
{{{Sos1(S1167~O)->Sos1(S1167~P)}}}
{{{Sos1(S1178~O)->Sos1(S1178~P)}}}
{{{Sos1(S1193~O)->Sos1(S1193~P)}}}
!Rules
{{{
265 ERK2(STkinase!1,T185~P,Y187~P).Sos1(S1132~O!1) -> \
 ERK2(STkinase!1,T185~P,Y187~P).Sos1(S1132~P!1)        MAPKkp33

266 ERK2(STkinase!1,T185~P,Y187~P).Sos1(S1167~O!1) -> \
 ERK2(STkinase!1,T185~P,Y187~P).Sos1(S1167~P!1)        MAPKkp34

267 ERK2(STkinase!1,T185~P,Y187~P).Sos1(S1178~O!1) -> \
 ERK2(STkinase!1,T185~P,Y187~P).Sos1(S1178~P!1)        MAPKkp35

268 ERK2(STkinase!1,T185~P,Y187~P).Sos1(S1193~O!1) -> \
 ERK2(STkinase!1,T185~P,Y187~P).Sos1(S1193~P!1)        MAPKkp36

269 ERK2(STkinase!1,T185~P,Y187~P).EGFR(T669~O!1) -> \
 ERK2(STkinase!1,T185~P,Y187~P).EGFR(T669~P!1)      MAPKkp37

270 ERK2(STkinase!1,T185~P,Y187~P).Raf1(S29~O!1) -> \
 ERK2(STkinase!1,T185~P,Y187~P).Raf1(S29~P!1)   MAPKkp38

271 ERK2(STkinase!1,T185~P,Y187~P).Raf1(S43~O!1) -> \
 ERK2(STkinase!1,T185~P,Y187~P).Raf1(S43~P!1)   MAPKkp39

272 ERK2(STkinase!1,T185~P,Y187~P).Raf1(S289~O!1) -> \
 ERK2(STkinase!1,T185~P,Y187~P).Raf1(S289~P!1)   MAPKkp40

273 ERK2(STkinase!1,T185~P,Y187~P).Raf1(S301~O!1) -> \
 ERK2(STkinase!1,T185~P,Y187~P).Raf1(S301~P!1)   MAPKkp41

274 ERK2(STkinase!1,T185~P,Y187~P).Raf1(S471~O!1) -> \
 ERK2(STkinase!1,T185~P,Y187~P).Raf1(S471~P!1)   MAPKkp42

275 ERK2(STkinase!1,T185~P,Y187~P).Raf1(S642~O!1) -> \
 ERK2(STkinase!1,T185~P,Y187~P).Raf1(S642~P!1)   MAPKkp43

276 ERK2(STkinase!1,T185~P,Y187~P).MEK1(T292~O!1) -> \
 ERK2(STkinase!1,T185~P,Y187~P).MEK1(T292~P!1)        MAPKkp44

462 ERK2(STkinase!1,T185~P,Y187~P).Gab1(T312~O!1) -> \
 ERK2(STkinase!1,T185~P,Y187~P).Gab1(T312~P!1)    Gab1kp162

463 ERK2(STkinase!1,T185~P,Y187~P).Gab1(S381~O!1) -> \
 ERK2(STkinase!1,T185~P,Y187~P).Gab1(S381~P!1)    Gab1kp163

464 ERK2(STkinase!1,T185~P,Y187~P).Gab1(S454~O!1) -> \
 ERK2(STkinase!1,T185~P,Y187~P).Gab1(S454~P!1)    Gab1kp164

465 ERK2(STkinase!1,T185~P,Y187~P).Gab1(T476~O!1) -> \
 ERK2(STkinase!1,T185~P,Y187~P).Gab1(T476~P!1)    Gab1kp165

466 ERK2(STkinase!1,T185~P,Y187~P).Gab1(S581~O!1) -> \
 ERK2(STkinase!1,T185~P,Y187~P).Gab1(S581~P!1)    Gab1kp166

467 ERK2(STkinase!1,T185~P,Y187~P).Gab1(S597~O!1) -> \
 ERK2(STkinase!1,T185~P,Y187~P).Gab1(S597~P!1)    Gab1kp167
}}}
```

```
''~MEK1 reversibly binds ~ERK2'' (see Arrow 25 in [[Contact Map]])
{{{MEK1(delta)+ERK2(CD)<->MEK1(delta!1).ERK2(CD!1)}}}
!Rules
{{{
264 ERK2(STkinase,CD,T185~P,Y187~P) + MEK1(T292~O) -> \
 ERK2(STkinase!1,CD,T185~P,Y187~P).MEK1(T292~O!1)      MAPKkp32

300 ERK2(STkinase!1).MEK1(T292!1) -> \
 ERK2(STkinase) + MEK1(T292)	MAPKkp52
}}}
```

```
See ''~ErbB2'' in [[Proteins]]
!BNGL
{{{ErbB2(II,Y1139~O~P,Y1196~O~P,Y1222~O~P,Y1248~O~P,loc~M~En)}}}
!Summary
~ErbB2 (represented by {{{ErbB2}}} in the model) is an orphan receptor that forms heterodimers with other members of the ~ErbB family of receptors.  The component {{{II}}} represents domain II of the ectodomain; this domain mediates receptor dimerization.  The ectodomains of ~ErbB2 and EGFR are similar. In the model, ~ErbB2 is found in one of two compartments: the plasma membrane (M) compartment or the endocytic (En) compartment.  The component {{{loc}}} is introduced to track the location of ~ErbB2.  The internal state of {{{loc}}} ({{{M}}} or {{{En}}}) indicates the location of ~ErbB2. The other components of {{{ErbB2}}}, namely {{{Y1139}}} and {{{Y1222}}}, represent sites of phosphorylation.  Hazan et al. (1990) identified ~ErbB2 autophosphorylation sites.  Dankort et al. (2001) reported that pY1139 is a docking site of Grb2 and pY1222 is a docking site of Shc.

!DOG
[img[DOGS/ErbB2.jpg]]
!Reading
*[[OMIM ID 164870 | http://www.ncbi.nlm.nih.gov/omim/164870 ]]
*Dankort D, Jeyabalan N, Jones N, Dumont DJ, Muller WJ (2001) Multiple ~ErbB-2/Neu phosphorylation sites mediate transformation through distinct effector proteins. J Biol Chem 276:38921-38928. [[PMID: 11500516 | http://www.ncbi.nlm.nih.gov/pubmed/11500516]]
*Hazan R, Margolis B, Dombalagian M, Ullrich A, Zilberstein A, Schlessinger J (1990) Identification of autophosphorylation sites of ~HER2/neu. Cell Growth Differ 1:3-7. [[PMID: 1706616 | http://www.ncbi.nlm.nih.gov/pubmed/1706616]]
```

```
''~ErbB2 catalyzes phosphorylation of EGFR, ~ErbB3, ~ErbB4, p52Shc1, and Gab1'' (see Arrow 8 in [[Contact Map]])
{{{EGFR(Y992~O)->EGFR(Y992~P)}}}
{{{EGFR(Y1068~O)->EGFR(Y1068~P)}}}
{{{EGFR(Y1086~O)->EGFR(Y1086~P)}}}
{{{EGFR(Y1148~O)->EGFR(Y1148~P)}}}
{{{EGFR(Y1173~O)->EGFR(Y1173~P)}}}
{{{ErbB3(Y1051~O)->ErbB3(Y1051~P)}}}
{{{ErbB3(Y1194~O)->ErbB3(Y1194~P)}}}
{{{ErbB3(Y1219~O)->ErbB3(Y1219~P)}}}
{{{ErbB3(Y1257~O)->ErbB3(Y1257~P)}}}
{{{ErbB3(Y1273~O)->ErbB3(Y1273~P)}}}
{{{ErbB3(Y1286~O)->ErbB3(Y1286~P)}}}
{{{ErbB3(Y1325~O)->ErbB3(Y1325~P)}}}
{{{ErbB4(Y1056~O)->ErbB4(Y1056~P)}}}
{{{ErbB4(Y1188~O)->ErbB4(Y1188~P)}}}
{{{ErbB4(Y1242~O)->ErbB4(Y1242~P)}}}
{{{p52Shc1(Y317~O)->p52Shc1(Y317~P)}}}
{{{Gab1(Y447~O)->Gab1(Y447~P)}}}
{{{Gab1(Y472~O)->Gab1(Y472~P)}}}
{{{Gab1(Y589~O)->Gab1(Y589~P)}}}
!Rules
{{{
106 ErbB2(II!1).EGFR(I_III!3,II!1,Y992~O).EGF(EGFL!3) -> \
  ErbB2(II!1).EGFR(I_III!3,II!1,Y992~P).EGF(EGFL!3) Phosphokp6

107 ErbB2(II!1).EGFR(I_III!3,II!1,Y1068~O).EGF(EGFL!3) -> \
  ErbB2(II!1).EGFR(I_III!3,II!1,Y1068~P).EGF(EGFL!3) Phosphokp7

108 ErbB2(II!1).EGFR(I_III!3,II!1,Y1086~O).EGF(EGFL!3) -> \
  ErbB2(II!1).EGFR(I_III!3,II!1,Y1086~P).EGF(EGFL!3) Phosphokp8

109 ErbB2(II!1).EGFR(I_III!3,II!1,Y1114~O).EGF(EGFL!3) -> \
  ErbB2(II!1).EGFR(I_III!3,II!1,Y1114~P).EGF(EGFL!3) Phosphokp9

110 ErbB2(II!1).EGFR(I_III!3,II!1,Y1148~O).EGF(EGFL!3) -> \
  ErbB2(II!1).EGFR(I_III!3,II!1,Y1148~P).EGF(EGFL!3) Phosphokp10

111 ErbB2(II!1).EGFR(I_III!3,II!1,Y1173~O).EGF(EGFL!3) -> \
  ErbB2(II!1).EGFR(I_III!3,II!1,Y1173~P).EGF(EGFL!3) Phosphokp11

133 ErbB2(II!1).ErbB3(I_III!3,II!1,Y1054~O).HRG(EGFL!3) -> \
  ErbB2(II!1).ErbB3(I_III!3,II!1,Y1054~P).HRG(EGFL!3) Phosphokp34

134 ErbB2(II!1).ErbB3(I_III!3,II!1,Y1197~O).HRG(EGFL!3) -> \
  ErbB2(II!1).ErbB3(I_III!3,II!1,Y1197~P).HRG(EGFL!3) Phosphokp35

135 ErbB2(II!1).ErbB3(I_III!3,II!1,Y1222~O).HRG(EGFL!3) -> \
  ErbB2(II!1).ErbB3(I_III!3,II!1,Y1222~P).HRG(EGFL!3) Phosphokp36

136 ErbB2(II!1).ErbB3(I_III!3,II!1,Y1260~O).HRG(EGFL!3) -> \
  ErbB2(II!1).ErbB3(I_III!3,II!1,Y1260~P).HRG(EGFL!3) Phosphokp37

137 ErbB2(II!1).ErbB3(I_III!3,II!1,Y1276~O).HRG(EGFL!3) -> \
  ErbB2(II!1).ErbB3(I_III!3,II!1,Y1276~P).HRG(EGFL!3) Phosphokp38

138 ErbB2(II!1).ErbB3(I_III!3,II!1,Y1289~O).HRG(EGFL!3) -> \
  ErbB2(II!1).ErbB3(I_III!3,II!1,Y1289~P).HRG(EGFL!3) Phosphokp39

139 ErbB2(II!1).ErbB3(I_III!3,II!1,Y1328~O).HRG(EGFL!3) -> \
  ErbB2(II!1).ErbB3(I_III!3,II!1,Y1328~P).HRG(EGFL!3) Phosphokp40

150 ErbB2(II!1).ErbB4(I_III!3,II!1,Y1056~O).HRG(EGFL!3) -> \
  ErbB2(II!1).ErbB4(I_III!3,II!1,Y1056~P).HRG(EGFL!3) Phosphokp51

151 ErbB2(II!1).ErbB4(I_III!3,II!1,Y1188~O).HRG(EGFL!3) -> \
  ErbB2(II!1).ErbB4(I_III!3,II!1,Y1188~P).HRG(EGFL!3) Phosphokp52

152 ErbB2(II!1).ErbB4(I_III!3,II!1,Y1242~O).HRG(EGFL!3) -> \
  ErbB2(II!1).ErbB4(I_III!3,II!1,Y1242~P).HRG(EGFL!3) Phosphokp53

199 ErbB2(II!1).p52Shc1(Y317~O,PTB!4).EGFR(I_III!2,II!1,Y992~P!4).EGF(EGFL!2) -> \
 ErbB2(II!1).p52Shc1(Y317~P,PTB!4).EGFR(I_III!2,II!1,Y992~P!4).EGF(EGFL!2) Shc1kp18

200 ErbB2(II!1).p52Shc1(Y317~O,PTB!4).EGFR(I_III!2,II!1,Y1086~P!4).EGF(EGFL!2) -> \
 ErbB2(II!1).p52Shc1(Y317~P,PTB!4).EGFR(I_III!2,II!1,Y1086~P!4).EGF(EGFL!2) Shc1kp19

201 ErbB2(II!1).p52Shc1(Y317~O,PTB!4).EGFR(I_III!2,II!1,Y1114~P!4).EGF(EGFL!2) -> \
 ErbB2(II!1).p52Shc1(Y317~P,PTB!4).EGFR(I_III!2,II!1,Y1114~P!4).EGF(EGFL!2) Shc1kp20

202 ErbB2(II!1).p52Shc1(Y317~O,PTB!4).ErbB3(I_III!2,II!1,Y1328~P!4).HRG(EGFL!2) -> \
 ErbB2(II!1).p52Shc1(Y317~P,PTB!4).ErbB3(I_III!2,II!1,Y1328~P!4).HRG(EGFL!2) Shc1kp21

203 ErbB2(II!1).p52Shc1(Y317~O,PTB!4).ErbB4(I_III!3,II!1,Y1188~P!4).HRG(EGFL!3) -> \
 ErbB2(II!1).p52Shc1(Y317~P,PTB!4).ErbB4(I_III!3,II!1,Y1188~P!4).HRG(EGFL!3)    Shc1kp22

204 ErbB2(II!1).p52Shc1(Y317~O,PTB!4).ErbB4(I_III!3,II!1,Y1242~P!4).HRG(EGFL!3) -> \
 ErbB2(II!1).p52Shc1(Y317~P,PTB!4).ErbB4(I_III!3,II!1,Y1242~P!4).HRG(EGFL!3)    Shc1kp23

359 ErbB2(II!1).Gab1(Y447~O,PRS1_PRS2!6).Grb2(cSH3!6,SH2s!2).p52Shc1(Y317~P!2,PTB!4).EGFR(I_III!3,II!1,Y992~P!4).EGF(EGFL!3) -> \
 ErbB2(II!1).Gab1(Y447~P,PRS1_PRS2!6).Grb2(cSH3!6,SH2s!2).p52Shc1(Y317~P!2,PTB!4).EGFR(I_III!3,II!1,Y992~P!4).EGF(EGFL!3)    Gab1kp59

360 ErbB2(II!1).Gab1(Y472~O,PRS1_PRS2!6).Grb2(cSH3!6,SH2s!2).p52Shc1(Y317~P!2,PTB!4).EGFR(I_III!3,II!1,Y992~P!4).EGF(EGFL!3) -> \
 ErbB2(II!1).Gab1(Y472~P,PRS1_PRS2!6).Grb2(cSH3!6,SH2s!2).p52Shc1(Y317~P!2,PTB!4).EGFR(I_III!3,II!1,Y992~P!4).EGF(EGFL!3)    Gab1kp60

361 ErbB2(II!1).Gab1(Y619~O,PRS1_PRS2!6).Grb2(cSH3!6,SH2s!2).p52Shc1(Y317~P!2,PTB!4).EGFR(I_III!3,II!1,Y992~P!4).EGF(EGFL!3) -> \
 ErbB2(II!1).Gab1(Y619~P,PRS1_PRS2!6).Grb2(cSH3!6,SH2s!2).p52Shc1(Y317~P!2,PTB!4).EGFR(I_III!3,II!1,Y992~P!4).EGF(EGFL!3)    Gab1kp61

362 ErbB2(II!1).Gab1(Y657~O,PRS1_PRS2!6).Grb2(cSH3!6,SH2s!2).p52Shc1(Y317~P!2,PTB!4).EGFR(I_III!3,II!1,Y992~P!4).EGF(EGFL!3) -> \
 ErbB2(II!1).Gab1(Y657~P,PRS1_PRS2!6).Grb2(cSH3!6,SH2s!2).p52Shc1(Y317~P!2,PTB!4).EGFR(I_III!3,II!1,Y992~P!4).EGF(EGFL!3)    Gab1kp62

363 ErbB2(II!1).Gab1(Y447~O,PRS1_PRS2!6).Grb2(cSH3!6,SH2s!2).p52Shc1(Y317~P!2,PTB!4).EGFR(I_III!3,II!1,Y1086~P!4).EGF(EGFL!3) -> \
 ErbB2(II!1).Gab1(Y447~P,PRS1_PRS2!6).Grb2(cSH3!6,SH2s!2).p52Shc1(Y317~P!2,PTB!4).EGFR(I_III!3,II!1,Y1086~P!4).EGF(EGFL!3)    Gab1kp63

364 ErbB2(II!1).Gab1(Y472~O,PRS1_PRS2!6).Grb2(cSH3!6,SH2s!2).p52Shc1(Y317~P!2,PTB!4).EGFR(I_III!3,II!1,Y1086~P!4).EGF(EGFL!3) -> \
 ErbB2(II!1).Gab1(Y472~P,PRS1_PRS2!6).Grb2(cSH3!6,SH2s!2).p52Shc1(Y317~P!2,PTB!4).EGFR(I_III!3,II!1,Y1086~P!4).EGF(EGFL!3)    Gab1kp64

365 ErbB2(II!1).Gab1(Y619~O,PRS1_PRS2!6).Grb2(cSH3!6,SH2s!2).p52Shc1(Y317~P!2,PTB!4).EGFR(I_III!3,II!1,Y1086~P!4).EGF(EGFL!3) -> \
 ErbB2(II!1).Gab1(Y619~P,PRS1_PRS2!6).Grb2(cSH3!6,SH2s!2).p52Shc1(Y317~P!2,PTB!4).EGFR(I_III!3,II!1,Y1086~P!4).EGF(EGFL!3)    Gab1kp65

366 ErbB2(II!1).Gab1(Y657~O,PRS1_PRS2!6).Grb2(cSH3!6,SH2s!2).p52Shc1(Y317~P!2,PTB!4).EGFR(I_III!3,II!1,Y1086~P!4).EGF(EGFL!3) -> \
 ErbB2(II!1).Gab1(Y657~P,PRS1_PRS2!6).Grb2(cSH3!6,SH2s!2).p52Shc1(Y317~P!2,PTB!4).EGFR(I_III!3,II!1,Y1086~P!4).EGF(EGFL!3)    Gab1kp66

367 ErbB2(II!1).Gab1(Y447~O,PRS1_PRS2!6).Grb2(cSH3!6,SH2s!2).p52Shc1(Y317~P!2,PTB!4).EGFR(I_III!3,II!1,Y1114~P!4).EGF(EGFL!3) -> \
 ErbB2(II!1).Gab1(Y447~P,PRS1_PRS2!6).Grb2(cSH3!6,SH2s!2).p52Shc1(Y317~P!2,PTB!4).EGFR(I_III!3,II!1,Y1114~P!4).EGF(EGFL!3)    Gab1kp67

368 ErbB2(II!1).Gab1(Y472~O,PRS1_PRS2!6).Grb2(cSH3!6,SH2s!2).p52Shc1(Y317~P!2,PTB!4).EGFR(I_III!3,II!1,Y1114~P!4).EGF(EGFL!3) -> \
 ErbB2(II!1).Gab1(Y472~P,PRS1_PRS2!6).Grb2(cSH3!6,SH2s!2).p52Shc1(Y317~P!2,PTB!4).EGFR(I_III!3,II!1,Y1114~P!4).EGF(EGFL!3)    Gab1kp68

369 ErbB2(II!1).Gab1(Y619~O,PRS1_PRS2!6).Grb2(cSH3!6,SH2s!2).p52Shc1(Y317~P!2,PTB!4).EGFR(I_III!3,II!1,Y1114~P!4).EGF(EGFL!3) -> \
 ErbB2(II!1).Gab1(Y619~P,PRS1_PRS2!6).Grb2(cSH3!6,SH2s!2).p52Shc1(Y317~P!2,PTB!4).EGFR(I_III!3,II!1,Y1114~P!4).EGF(EGFL!3)    Gab1kp69

370 ErbB2(II!1).Gab1(Y657~O,PRS1_PRS2!6).Grb2(cSH3!6,SH2s!2).p52Shc1(Y317~P!2,PTB!4).EGFR(I_III!3,II!1,Y1114~P!4).EGF(EGFL!3) -> \
 ErbB2(II!1).Gab1(Y657~P,PRS1_PRS2!6).Grb2(cSH3!6,SH2s!2).p52Shc1(Y317~P!2,PTB!4).EGFR(I_III!3,II!1,Y1114~P!4).EGF(EGFL!3)    Gab1kp70

371 ErbB2(II!1).Gab1(Y447~O,PRS1_PRS2!6).Grb2(cSH3!6,SH2r!4).EGFR(I_III!3,II!1,Y1068~P!4).EGF(EGFL!3) -> \
 ErbB2(II!1).Gab1(Y447~P,PRS1_PRS2!6).Grb2(cSH3!6,SH2r!4).EGFR(I_III!3,II!1,Y1068~P!4).EGF(EGFL!3)    Gab1kp71

372 ErbB2(II!1).Gab1(Y472~O,PRS1_PRS2!6).Grb2(cSH3!6,SH2r!4).EGFR(I_III!3,II!1,Y1068~P!4).EGF(EGFL!3) -> \
 ErbB2(II!1).Gab1(Y472~P,PRS1_PRS2!6).Grb2(cSH3!6,SH2r!4).EGFR(I_III!3,II!1,Y1068~P!4).EGF(EGFL!3)    Gab1kp72

373 ErbB2(II!1).Gab1(Y619~O,PRS1_PRS2!6).Grb2(cSH3!6,SH2r!4).EGFR(I_III!3,II!1,Y1068~P!4).EGF(EGFL!3) -> \
 ErbB2(II!1).Gab1(Y619~P,PRS1_PRS2!6).Grb2(cSH3!6,SH2r!4).EGFR(I_III!3,II!1,Y1068~P!4).EGF(EGFL!3)    Gab1kp73

374 ErbB2(II!1).Gab1(Y657~O,PRS1_PRS2!6).Grb2(cSH3!6,SH2r!4).EGFR(I_III!3,II!1,Y1068~P!4).EGF(EGFL!3) -> \
 ErbB2(II!1).Gab1(Y657~P,PRS1_PRS2!6).Grb2(cSH3!6,SH2r!4).EGFR(I_III!3,II!1,Y1068~P!4).EGF(EGFL!3)    Gab1kp74

375 ErbB2(II!1).Gab1(Y447~O,PRS1_PRS2!6).Grb2(cSH3!6,SH2r!4).EGFR(I_III!3,II!1,Y1114~P!4).EGF(EGFL!3) -> \
 ErbB2(II!1).Gab1(Y447~P,PRS1_PRS2!6).Grb2(cSH3!6,SH2r!4).EGFR(I_III!3,II!1,Y1114~P!4).EGF(EGFL!3)    Gab1kp75

376 ErbB2(II!1).Gab1(Y472~O,PRS1_PRS2!6).Grb2(cSH3!6,SH2r!4).EGFR(I_III!3,II!1,Y1114~P!4).EGF(EGFL!3) -> \
 ErbB2(II!1).Gab1(Y472~P,PRS1_PRS2!6).Grb2(cSH3!6,SH2r!4).EGFR(I_III!3,II!1,Y1114~P!4).EGF(EGFL!3)    Gab1kp76

377 ErbB2(II!1).Gab1(Y619~O,PRS1_PRS2!6).Grb2(cSH3!6,SH2r!4).EGFR(I_III!3,II!1,Y1114~P!4).EGF(EGFL!3) -> \
 ErbB2(II!1).Gab1(Y619~P,PRS1_PRS2!6).Grb2(cSH3!6,SH2r!4).EGFR(I_III!3,II!1,Y1114~P!4).EGF(EGFL!3)    Gab1kp77

378 ErbB2(II!1).Gab1(Y657~O,PRS1_PRS2!6).Grb2(cSH3!6,SH2r!4).EGFR(I_III!3,II!1,Y1114~P!4).EGF(EGFL!3) -> \
 ErbB2(II!1).Gab1(Y657~P,PRS1_PRS2!6).Grb2(cSH3!6,SH2r!4).EGFR(I_III!3,II!1,Y1114~P!4).EGF(EGFL!3)    Gab1kp78

379 ErbB2(II!1).Gab1(Y447~O,PRS1_PRS2!6).Grb2(cSH3!6,SH2r!4).EGFR(I_III!3,II!1,Y1148~P!4).EGF(EGFL!3) -> \
 ErbB2(II!1).Gab1(Y447~P,PRS1_PRS2!6).Grb2(cSH3!6,SH2r!4).EGFR(I_III!3,II!1,Y1148~P!4).EGF(EGFL!3)    Gab1kp79

380 ErbB2(II!1).Gab1(Y472~O,PRS1_PRS2!6).Grb2(cSH3!6,SH2r!4).EGFR(I_III!3,II!1,Y1148~P!4).EGF(EGFL!3) -> \
 ErbB2(II!1).Gab1(Y472~P,PRS1_PRS2!6).Grb2(cSH3!6,SH2r!4).EGFR(I_III!3,II!1,Y1148~P!4).EGF(EGFL!3)    Gab1kp80

381 ErbB2(II!1).Gab1(Y619~O,PRS1_PRS2!6).Grb2(cSH3!6,SH2r!4).EGFR(I_III!3,II!1,Y1148~P!4).EGF(EGFL!3) -> \
 ErbB2(II!1).Gab1(Y619~P,PRS1_PRS2!6).Grb2(cSH3!6,SH2r!4).EGFR(I_III!3,II!1,Y1148~P!4).EGF(EGFL!3)    Gab1kp81

382 ErbB2(II!1).Gab1(Y657~O,PRS1_PRS2!6).Grb2(cSH3!6,SH2r!4).EGFR(I_III!3,II!1,Y1148~P!4).EGF(EGFL!3) -> \
 ErbB2(II!1).Gab1(Y657~P,PRS1_PRS2!6).Grb2(cSH3!6,SH2r!4).EGFR(I_III!3,II!1,Y1148~P!4).EGF(EGFL!3)    Gab1kp82

383 ErbB2(II!1).Gab1(Y447~O,PRS1_PRS2!6).Grb2(cSH3!6,SH2r!4).EGFR(I_III!3,II!1,Y1173~P!4).EGF(EGFL!3) -> \
 ErbB2(II!1).Gab1(Y447~P,PRS1_PRS2!6).Grb2(cSH3!6,SH2r!4).EGFR(I_III!3,II!1,Y1173~P!4).EGF(EGFL!3)    Gab1kp83

384 ErbB2(II!1).Gab1(Y472~O,PRS1_PRS2!6).Grb2(cSH3!6,SH2r!4).EGFR(I_III!3,II!1,Y1173~P!4).EGF(EGFL!3) -> \
 ErbB2(II!1).Gab1(Y472~P,PRS1_PRS2!6).Grb2(cSH3!6,SH2r!4).EGFR(I_III!3,II!1,Y1173~P!4).EGF(EGFL!3)    Gab1kp84

385 ErbB2(II!1).Gab1(Y619~O,PRS1_PRS2!6).Grb2(cSH3!6,SH2r!4).EGFR(I_III!3,II!1,Y1173~P!4).EGF(EGFL!3) -> \
 ErbB2(II!1).Gab1(Y619~P,PRS1_PRS2!6).Grb2(cSH3!6,SH2r!4).EGFR(I_III!3,II!1,Y1173~P!4).EGF(EGFL!3)    Gab1kp85

386 ErbB2(II!1).Gab1(Y657~O,PRS1_PRS2!6).Grb2(cSH3!6,SH2r!4).EGFR(I_III!3,II!1,Y1173~P!4).EGF(EGFL!3) -> \
 ErbB2(II!1).Gab1(Y657~P,PRS1_PRS2!6).Grb2(cSH3!6,SH2r!4).EGFR(I_III!3,II!1,Y1173~P!4).EGF(EGFL!3)    Gab1kp86

387 ErbB2(II!1).Gab1(Y447~O,PRS1_PRS2!5).Grb2(cSH3!5,SH2s!2).p52Shc1(Y317~P!2,PTB!4).ErbB3(I_III!3,II!1,Y1328~P!4).HRG(EGFL!3) -> \
 ErbB2(II!1).Gab1(Y447~P,PRS1_PRS2!5).Grb2(cSH3!5,SH2s!2).p52Shc1(Y317~P!2,PTB!4).ErbB3(I_III!3,II!1,Y1328~P!4).HRG(EGFL!3)    Gab1kp87

388 ErbB2(II!1).Gab1(Y472~O,PRS1_PRS2!5).Grb2(cSH3!5,SH2s!2).p52Shc1(Y317~P!2,PTB!4).ErbB3(I_III!3,II!1,Y1328~P!4).HRG(EGFL!3) -> \
 ErbB2(II!1).Gab1(Y472~P,PRS1_PRS2!5).Grb2(cSH3!5,SH2s!2).p52Shc1(Y317~P!2,PTB!4).ErbB3(I_III!3,II!1,Y1328~P!4).HRG(EGFL!3)    Gab1kp88

389 ErbB2(II!1).Gab1(Y619~O,PRS1_PRS2!5).Grb2(cSH3!5,SH2s!2).p52Shc1(Y317~P!2,PTB!4).ErbB3(I_III!3,II!1,Y1328~P!4).HRG(EGFL!3) -> \
 ErbB2(II!1).Gab1(Y619~P,PRS1_PRS2!5).Grb2(cSH3!5,SH2s!2).p52Shc1(Y317~P!2,PTB!4).ErbB3(I_III!3,II!1,Y1328~P!4).HRG(EGFL!3)    Gab1kp89

390 ErbB2(II!1).Gab1(Y657~O,PRS1_PRS2!5).Grb2(cSH3!5,SH2s!2).p52Shc1(Y317~P!2,PTB!4).ErbB3(I_III!3,II!1,Y1328~P!4).HRG(EGFL!3) -> \
 ErbB2(II!1).Gab1(Y657~P,PRS1_PRS2!5).Grb2(cSH3!5,SH2s!2).p52Shc1(Y317~P!2,PTB!4).ErbB3(I_III!3,II!1,Y1328~P!4).HRG(EGFL!3)    Gab1kp90

391 ErbB2(II!1).Gab1(Y447~O,PRS1_PRS2!6).Grb2(cSH3!6,SH2s!2).p52Shc1(Y317~P!2,PTB!4).ErbB4(I_III!3,II!1,Y1188~P!4).HRG(EGFL!3) -> \
 ErbB2(II!1).Gab1(Y447~P,PRS1_PRS2!6).Grb2(cSH3!6,SH2s!2).p52Shc1(Y317~P!2,PTB!4).ErbB4(I_III!3,II!1,Y1188~P!4).HRG(EGFL!3)    Gab1kp91

392 ErbB2(II!1).Gab1(Y472~O,PRS1_PRS2!6).Grb2(cSH3!6,SH2s!2).p52Shc1(Y317~P!2,PTB!4).ErbB4(I_III!3,II!1,Y1188~P!4).HRG(EGFL!3) -> \
 ErbB2(II!1).Gab1(Y472~P,PRS1_PRS2!6).Grb2(cSH3!6,SH2s!2).p52Shc1(Y317~P!2,PTB!4).ErbB4(I_III!3,II!1,Y1188~P!4).HRG(EGFL!3)	Gab1kp92

393 ErbB2(II!1).Gab1(Y619~O,PRS1_PRS2!6).Grb2(cSH3!6,SH2s!2).p52Shc1(Y317~P!2,PTB!4).ErbB4(I_III!3,II!1,Y1188~P!4).HRG(EGFL!3) -> \
 ErbB2(II!1).Gab1(Y619~P,PRS1_PRS2!6).Grb2(cSH3!6,SH2s!2).p52Shc1(Y317~P!2,PTB!4).ErbB4(I_III!3,II!1,Y1188~P!4).HRG(EGFL!3)    Gab1kp93

394 ErbB2(II!1).Gab1(Y657~O,PRS1_PRS2!6).Grb2(cSH3!6,SH2s!2).p52Shc1(Y317~P!2,PTB!4).ErbB4(I_III!3,II!1,Y1188~P!4).HRG(EGFL!3) -> \
 ErbB2(II!1).Gab1(Y657~P,PRS1_PRS2!6).Grb2(cSH3!6,SH2s!2).p52Shc1(Y317~P!2,PTB!4).ErbB4(I_III!3,II!1,Y1188~P!4).HRG(EGFL!3)    Gab1kp94

395 ErbB2(II!1).Gab1(Y447~O,PRS1_PRS2!6).Grb2(cSH3!6,SH2s!2).p52Shc1(Y317~P!2,PTB!4).ErbB4(I_III!3,II!1,Y1242~P!4).HRG(EGFL!3) -> \
 ErbB2(II!1).Gab1(Y447~P,PRS1_PRS2!6).Grb2(cSH3!6,SH2s!2).p52Shc1(Y317~P!2,PTB!4).ErbB4(I_III!3,II!1,Y1242~P!4).HRG(EGFL!3)    Gab1kp95

396 ErbB2(II!1).Gab1(Y472~O,PRS1_PRS2!6).Grb2(cSH3!6,SH2s!2).p52Shc1(Y317~P!2,PTB!4).ErbB4(I_III!3,II!1,Y1242~P!4).HRG(EGFL!3) -> \
 ErbB2(II!1).Gab1(Y472~P,PRS1_PRS2!6).Grb2(cSH3!6,SH2s!2).p52Shc1(Y317~P!2,PTB!4).ErbB4(I_III!3,II!1,Y1242~P!4).HRG(EGFL!3)	Gab1kp96

397 ErbB2(II!1).Gab1(Y619~O,PRS1_PRS2!6).Grb2(cSH3!6,SH2s!2).p52Shc1(Y317~P!2,PTB!4).ErbB4(I_III!3,II!1,Y1242~P!4).HRG(EGFL!3) -> \
 ErbB2(II!1).Gab1(Y619~P,PRS1_PRS2!6).Grb2(cSH3!6,SH2s!2).p52Shc1(Y317~P!2,PTB!4).ErbB4(I_III!3,II!1,Y1242~P!4).HRG(EGFL!3)    Gab1kp97

398 ErbB2(II!1).Gab1(Y657~O,PRS1_PRS2!6).Grb2(cSH3!6,SH2s!2).p52Shc1(Y317~P!2,PTB!4).ErbB4(I_III!3,II!1,Y1242~P!4).HRG(EGFL!3) -> \
 ErbB2(II!1).Gab1(Y657~P,PRS1_PRS2!6).Grb2(cSH3!6,SH2s!2).p52Shc1(Y317~P!2,PTB!4).ErbB4(I_III!3,II!1,Y1242~P!4).HRG(EGFL!3)    Gab1kp98
}}}
```

```
''ErbB2 homodimerization'' (see Arrow 4 in [[Contact Map]])
{{{ErbB2(II)+ErbB2(II)<->ErbB2(II!1).ErbB2(II!1)}}}
!Rules
```

```
''~ErbB2 reversibly binds ~ErbB3'' (see Arrows 4 and 5 in [[Contact Map]])
{{{ErbB2(II)+ErbB3(II)<->ErbB2(II!1).ErbB3(II!1)}}}
!Rules
{{{
39 ErbB2(II,loc~M) + ErbB3(I_III,II,loc~M) <-> \
 ErbB2(II!1,loc~M).ErbB3(I_III,II!1,loc~M)       Dimkp15,Dimkm15

40 ErbB3(I_III!1,II,loc~M).HRG(EGFL!1,loc~Ex) + ErbB2(II,loc~M) <-> \
 ErbB3(I_III!1,II!2,loc~M).HRG(EGFL!1,loc~Ex).ErbB2(II!2,loc~M)     Dimkp16,Dimkm16
}}}
```

```
''~ErbB2 reversibly binds ErbB4'' (see Arrow 6 in [[Contact Map]])
{{{ErbB2(II)+ErbB4(II)<->ErbB2(II!1).ErbB4(II!1)}}}
!Rules
{{{
41 ErbB2(II,loc~M) + ErbB4(I_III,II,loc~M) <-> \
 ErbB2(II!1,loc~M).ErbB4(I_III,II!1,loc~M)       Dimkp17,Dimkm17

42 ErbB4(I_III!1,II,loc~M).HRG(EGFL!1,loc~Ex) + ErbB2(II,loc~M) <-> \
 ErbB4(I_III!1,II!2,loc~M).HRG(EGFL!1,loc~Ex).ErbB2(II!2,loc~M)     Dimkp18,Dimkm18
}}}
```

```
See ''~ErbB3'' in [[Proteins]]
!BNGL
{{{ErbB3(I_III,II,Y1054~O~P,Y1197~O~P,Y1222~O~P,Y1260~O~P,Y1276~O~P,Y1289~O~P,Y1328~O~P,\
loc~M~En)}}}
!Summary
The component {{{I_III}}} represents domains I (L1) and III (L2) of the ectodomain of ~ErbB3, which are responsible for the interaction with HRG.  The component {{{II}}} represents domain II (which is also called the ~CR1 domain) of the ectodomain of ~ErbB3, which is involved in ~ErbB3 dimerization via ligand-induced receptor-receptor interaction.  The remaining components of {{{ErbB3}}} are sites of phosphorylation, and most of these sites are p85 docking sites.
!DOG
[img[DOGS/ErbB3.jpg]]
!Reading
*[[OMIM ID 190151 | http://www.ncbi.nlm.nih.gov/omim/190151 ]]
*Sithanandam G, Anderson LM (2008) The ~ERBB3 receptor in cancer and caner gene therapy. Cancer Gene Ther 15:413-448. [[PMID: 18404164 | http://www.ncbi.nlm.nih.gov/pubmed/18404164]]
```

```
''~ErbB3 homodimerization'' (see Arrow  5 in [[Contact Map]])
{{{ErbB3(II)+ErbB3(II)<->ErbB3(II!1).ErbB3(II!1)}}}
!Rules
{{{
43 ErbB3(I_III,II,loc~M) + ErbB3(I_III,II,loc~M) <-> \
 ErbB3(I_III,II!1,loc~M).ErbB3(I_III,II!1,loc~M)       Dimkp19,Dimkm19

44 ErbB3(I_III!1,II,loc~M).HRG(EGFL!1,loc~Ex) + ErbB3(I_III,II,loc~M) <-> \
 ErbB3(I_III!1,II!2,loc~M).HRG(EGFL!1,loc~Ex).ErbB3(I_III,II!2,loc~M)     Dimkp20,Dimkm20

45 ErbB3(I_III!1,II,loc~M).HRG(EGFL!1,loc~Ex) + ErbB3(I_III!2,II,loc~M).HRG(EGFL!2,loc~Ex) <-> \
 ErbB3(I_III!1,II!3,loc~M).HRG(EGFL!1,loc~Ex).ErbB3(I_III!2,II!3,loc~M).HRG(EGFL!2,loc~Ex)   Dimkp21,Dimkm21
}}}
```

```
''~ErbB3 reversibly binds ~ErbB4'' (see Arrow 6 in [[Contact Map]])
{{{ErbB3(II)+ErbB4(II)<->ErbB3(II!1).ErbB4(II!1)}}}
!Rules
{{{
46 ErbB3(I_III,II,loc~M) + ErbB4(I_III,II,loc~M) <-> \
 ErbB3(I_III,II!1,loc~M).ErbB4(I_III,II!1,loc~M)       Dimkp22,Dimkm22

47 ErbB3(I_III!1,II,loc~M).HRG(EGFL!1,loc~Ex) + ErbB4(I_III,II,loc~M) <-> \
 ErbB3(I_III!1,II!2,loc~M).HRG(EGFL!1,loc~Ex).ErbB4(I_III,II!2,loc~M)     Dimkp23,Dimkm23

48 ErbB3(I_III,II,loc~M) + ErbB4(I_III!1,II,loc~M).HRG(EGFL!1,loc~Ex) <-> \
 ErbB3(I_III,II!2,loc~M).ErbB4(I_III!1,II!2,loc~M).HRG(EGFL!1,loc~Ex)     Dimkp24,Dimkm24

49 ErbB3(I_III!1,II,loc~M).HRG(EGFL!1,loc~Ex) + ErbB4(I_III!2,II,loc~M).HRG(EGFL!2,loc~Ex) <-> \
 ErbB3(I_III!1,II!3,loc~M).HRG(EGFL!1,loc~Ex).ErbB4(I_III!2,II!3,loc~M).HRG(EGFL!2,loc~Ex)   Dimkp25,Dimkm25
}}}
```

```
''HRG reversibly binds ~ErbB3'' (see Arrow 2 in [[Contact Map]])
{{{HRG(EGFL)+ErbB3(I_III)<->HRG(EGFL!1).ErbB3(I_III!1)}}}
!Rules
{{{
8 EGFR(I_III,II!1,loc~M).ErbB3(I_III,II!1,loc~M) + HRG(EGFL,deg~F,loc~Ex) <-> \
 EGFR(I_III,II!1,loc~M).ErbB3(I_III!2,II!1,loc~M).HRG(EGFL!2,deg~F,loc~Ex)     HRG3kp2,HRG3km2

9 EGF(EGFL!2,deg~F,loc~Ex).EGFR(I_III!2,II!1,loc~M).ErbB3(I_III,II!1,loc~M) + HRG(EGFL,deg~F,loc~Ex) <-> \
 EGF(EGFL!2,deg~F,loc~Ex).EGFR(I_III!2,II!1,loc~M).ErbB3(I_III!3,II!1,loc~M).HRG(EGFL!3,deg~F,loc~Ex)     HRG3kp3,HRG3km3

15 ErbB2(II!1,loc~M).ErbB3(I_III,II!1,loc~M) + HRG(EGFL,deg~F,loc~Ex) <-> \
 ErbB2(II!1,loc~M).ErbB3(I_III!2,II!1,loc~M).HRG(EGFL!2,deg~F,loc~Ex)     HRG3kp2,HRG3km2

17 ErbB3(I_III,II!1,loc~M).ErbB3(I_III,II!1,loc~M) + HRG(EGFL,deg~F,loc~Ex) <-> \
 ErbB3(I_III,II!1,loc~M).ErbB3(I_III!2,II!1,loc~M).HRG(EGFL!2,deg~F,loc~Ex)     HRG3kp2,HRG3km2

18 HRG(EGFL!2,deg~F,loc~Ex).ErbB3(I_III!2,II!1,loc~M).ErbB3(I_III,II!1,loc~M) + HRG(EGFL,deg~F,loc~Ex) <-> \
 HRG(EGFL!2,deg~F,loc~Ex).ErbB3(I_III!2,II!1,loc~M).ErbB3(I_III!3,II!1,loc~M).HRG(EGFL!3,deg~F,loc~Ex)     HRG3kp3,HRG3km3

20 HRG(EGFL,deg~F,loc~Ex) + ErbB3(I_III,II!1,loc~M).ErbB4(I_III,II!1,loc~M) <-> \
 HRG(EGFL!2,deg~F,loc~Ex).ErbB3(I_III!2,II!1,loc~M).ErbB4(I_III,II!1,loc~M)     HRG3kp2,HRG3km2

22 HRG(EGFL,deg~F,loc~Ex) + ErbB3(I_III,II!1,loc~M).ErbB4(I_III!3,II!1,loc~M).HRG(EGFL!3,deg~F,loc~Ex) <-> \
 HRG(EGFL!2,deg~F,loc~Ex).ErbB3(I_III!2,II!1,loc~M).ErbB4(I_III!3,II!1,loc~M).HRG(EGFL!3,deg~F,loc~Ex)     HRG3kp3,HRG3km3
}}}
```

```
''~PI3K reversibly binds~ErbB3'' (see Arrow 21 in [[Contact Map]])
{{{PI3K(R_p85_nSH2_cSH2)+ErbB3(Y1051~P)<->PI3K(R_p85_nSH2_cSH2!1).ErbB3(Y1051~P!1)}}}
{{{PI3K(R_p85_nSH2_cSH2)+ErbB3(Y1194~P)<->PI3K(R_p85_nSH2_cSH2!1).ErbB3(Y1194~P!1)}}}
{{{PI3K(R_p85_nSH2_cSH2)+ErbB3(Y1219~P)<->PI3K(R_p85_nSH2_cSH2!1).ErbB3(Y1219~P!1)}}}
{{{PI3K(R_p85_nSH2_cSH2)+ErbB3(Y1257~P)<->PI3K(R_p85_nSH2_cSH2!1).ErbB3(Y1257~P!1)}}}
{{{PI3K(R_p85_nSH2_cSH2)+ErbB3(Y1273~P)<->PI3K(R_p85_nSH2_cSH2!1).ErbB3(Y1273~P!1)}}}
{{{PI3K(R_p85_nSH2_cSH2)+ErbB3(Y1286~P)<->PI3K(R_p85_nSH2_cSH2!1).ErbB3(Y1286~P!1)}}}
!Rules
{{{
505 PI3K(R_p85_nSH2_cSH2,G_p85_nSH2_cSH2) + ErbB3(Y1054~P) <-> \
 PI3K(R_p85_nSH2_cSH2!1,G_p85_nSH2_cSH2).ErbB3(Y1054~P!1)	PI3Kkp7,PI3Kkm7

506 PI3K(R_p85_nSH2_cSH2,G_p85_nSH2_cSH2) + ErbB3(Y1197~P) <-> \
 PI3K(R_p85_nSH2_cSH2!1,G_p85_nSH2_cSH2).ErbB3(Y1197~P!1)	PI3Kkp8,PI3Kkm8

507 PI3K(R_p85_nSH2_cSH2,G_p85_nSH2_cSH2) + ErbB3(Y1222~P) <-> \
 PI3K(R_p85_nSH2_cSH2!1,G_p85_nSH2_cSH2).ErbB3(Y1222~P!1)	PI3Kkp9,PI3Kkm9

508 PI3K(R_p85_nSH2_cSH2,G_p85_nSH2_cSH2) + ErbB3(Y1260~P) <-> \
 PI3K(R_p85_nSH2_cSH2!1,G_p85_nSH2_cSH2).ErbB3(Y1260~P!1)	PI3Kkp10,PI3Kkm10

509 PI3K(R_p85_nSH2_cSH2,G_p85_nSH2_cSH2) + ErbB3(Y1276~P) <-> \
 PI3K(R_p85_nSH2_cSH2!1,G_p85_nSH2_cSH2).ErbB3(Y1276~P!1)	PI3Kkp11,PI3Kkm11

510 PI3K(R_p85_nSH2_cSH2,G_p85_nSH2_cSH2) + ErbB3(Y1289~P) <-> \
 PI3K(R_p85_nSH2_cSH2!1,G_p85_nSH2_cSH2).ErbB3(Y1289~P!1)	PI3Kkp12,PI3Kkm12
}}}
```

```
See ''~ErbB4'' in [[Proteins]]
!BNGL
{{{ErbB4(I_III,II,Y1056~O~P,Y1188~O~p,Y1242~O~P,loc~M~En)}}}
!Summary
We take ~ErbB4 to be the same as EGFR except that ~ErbB4 binds ~NRG1 instead of EGF.
!DOG
[img[DOGS/ErbB4.jpg]]
!Reading
*[[OMIM ID 600543 | http://www.ncbi.nlm.nih.gov/omim/600543 ]]
*Mei L, Xiong WC (2008) Neuregulin 1 in neural development, synaptic plasticity and schizophrenia. Nat Rev Neurosci 9:437-452. [[PMID: 18478032 | http://www.ncbi.nlm.nih.gov/pubmed/18478032]]
```

```
''ErbB4 catalyzes phosphorylation of EGFR, ~ErbB2, ~ErbB3, ErbB4, p52Shc1, and Gab1'' (see Arrow 9 in [[Contact Map]])
{{{EGFR(Y992~O)->EGFR(Y992~P)}}}
{{{EGFR(Y1068~O)->EGFR(Y1068~P)}}}
{{{EGFR(Y1086~O)->EGFR(Y1086~P)}}}
{{{EGFR(Y1148~O)->EGFR(Y1148~P)}}}
{{{EGFR(Y1173~O)->EGFR(Y1173~P)}}}
{{{ErbB2(Y1139~O)->ErbB2(Y1139~P)}}}
{{{ErbB2(Y1222~O)->ErbB2(Y1222~P)}}}
{{{ErbB3(Y1051~O)->ErbB3(Y1051~P)}}}
{{{ErbB3(Y1194~O)->ErbB3(Y1194~P)}}}
{{{ErbB3(Y1219~O)->ErbB3(Y1219~P)}}}
{{{ErbB3(Y1257~O)->ErbB3(Y1257~P)}}}
{{{ErbB3(Y1273~O)->ErbB3(Y1273~P)}}}
{{{ErbB3(Y1286~O)->ErbB3(Y1286~P)}}}
{{{ErbB3(Y1325~O)->ErbB3(Y1325~P)}}}
{{{ErbB4(Y1056~O)->ErbB4(Y1056~P)}}}
{{{ErbB4(Y1188~O)->ErbB4(Y1188~P)}}}
{{{ErbB4(Y1242~O)->ErbB4(Y1242~P)}}}
{{{p52Shc1(Y317~O)->p52Shc1(Y317~P)}}}
{{{Gab1(Y447~O)->Gab1(Y447~P)}}}
{{{Gab1(Y472~O)->Gab1(Y472~P)}}}
{{{Gab1(Y589~O)->Gab1(Y589~P)}}}
!Rules
{{{
112 HRG(EGFL!2).ErbB4(I_III!2,II!1).EGFR(I_III!3,II!1,Y992~O).EGF(EGFL!3) -> \
  HRG(EGFL!2).ErbB4(I_III!2,II!1).EGFR(I_III!3,II!1,Y992~P).EGF(EGFL!3) Phosphokp12

113 HRG(EGFL!2).ErbB4(I_III!2,II!1).EGFR(I_III!3,II!1,Y1068~O).EGF(EGFL!3) -> \
  HRG(EGFL!2).ErbB4(I_III!2,II!1).EGFR(I_III!3,II!1,Y1068~P).EGF(EGFL!3) Phosphokp13

114 HRG(EGFL!2).ErbB4(I_III!2,II!1).EGFR(I_III!3,II!1,Y1086~O).EGF(EGFL!3) -> \
  HRG(EGFL!2).ErbB4(I_III!2,II!1).EGFR(I_III!3,II!1,Y1086~P).EGF(EGFL!3) Phosphokp14

115 HRG(EGFL!2).ErbB4(I_III!2,II!1).EGFR(I_III!3,II!1,Y1114~O).EGF(EGFL!3) -> \
  HRG(EGFL!2).ErbB4(I_III!2,II!1).EGFR(I_III!3,II!1,Y1114~P).EGF(EGFL!3) Phosphokp15

116 HRG(EGFL!2).ErbB4(I_III!2,II!1).EGFR(I_III!3,II!1,Y1148~O).EGF(EGFL!3) -> \
  HRG(EGFL!2).ErbB4(I_III!2,II!1).EGFR(I_III!3,II!1,Y1148~P).EGF(EGFL!3) Phosphokp16

117 HRG(EGFL!2).ErbB4(I_III!2,II!1).EGFR(I_III!3,II!1,Y1173~O).EGF(EGFL!3) -> \
  HRG(EGFL!2).ErbB4(I_III!2,II!1).EGFR(I_III!3,II!1,Y1173~P).EGF(EGFL!3) Phosphokp17

122 HRG(EGFL!2).ErbB4(I_III!2,II!1).ErbB2(II!1,Y1139~O) -> \
  HRG(EGFL!2).ErbB4(I_III!2,II!1).ErbB2(II!1,Y1139~P) Phosphokp23

123 HRG(EGFL!2).ErbB4(I_III!2,II!1).ErbB2(II!1,Y1196~O) -> \
  HRG(EGFL!2).ErbB4(I_III!2,II!1).ErbB2(II!1,Y1196~P) Phosphokp24

124 HRG(EGFL!2).ErbB4(I_III!2,II!1).ErbB2(II!1,Y1222~O) -> \
  HRG(EGFL!2).ErbB4(I_III!2,II!1).ErbB2(II!1,Y1222~P) Phosphokp25

125 HRG(EGFL!2).ErbB4(I_III!2,II!1).ErbB2(II!1,Y1248~O) -> \
  HRG(EGFL!2).ErbB4(I_III!2,II!1).ErbB2(II!1,Y1248~P) Phosphokp26

140 HRG(EGFL!2).ErbB4(I_III!2,II!1).ErbB3(I_III!3,II!1,Y1054~O).HRG(EGFL!3) -> \
  HRG(EGFL!2).ErbB4(I_III!2,II!1).ErbB3(I_III!3,II!1,Y1054~P).HRG(EGFL!3) Phosphokp41

141 HRG(EGFL!2).ErbB4(I_III!2,II!1).ErbB3(I_III!3,II!1,Y1197~O).HRG(EGFL!3) -> \
  HRG(EGFL!2).ErbB4(I_III!2,II!1).ErbB3(I_III!3,II!1,Y1197~P).HRG(EGFL!3) Phosphokp42

142 HRG(EGFL!2).ErbB4(I_III!2,II!1).ErbB3(I_III!3,II!1,Y1222~O).HRG(EGFL!3) -> \
  HRG(EGFL!2).ErbB4(I_III!2,II!1).ErbB3(I_III!3,II!1,Y1222~P).HRG(EGFL!3) Phosphokp43

143 HRG(EGFL!2).ErbB4(I_III!2,II!1).ErbB3(I_III!3,II!1,Y1260~O).HRG(EGFL!3) -> \
  HRG(EGFL!2).ErbB4(I_III!2,II!1).ErbB3(I_III!3,II!1,Y1260~P).HRG(EGFL!3) Phosphokp44

144 HRG(EGFL!2).ErbB4(I_III!2,II!1).ErbB3(I_III!3,II!1,Y1276~O).HRG(EGFL!3) -> \
  HRG(EGFL!2).ErbB4(I_III!2,II!1).ErbB3(I_III!3,II!1,Y1276~P).HRG(EGFL!3) Phosphokp45

145 HRG(EGFL!2).ErbB4(I_III!2,II!1).ErbB3(I_III!3,II!1,Y1289~O).HRG(EGFL!3) -> \
  HRG(EGFL!2).ErbB4(I_III!2,II!1).ErbB3(I_III!3,II!1,Y1289~P).HRG(EGFL!3) Phosphokp46

146 HRG(EGFL!2).ErbB4(I_III!2,II!1).ErbB3(I_III!3,II!1,Y1328~O).HRG(EGFL!3) -> \
  HRG(EGFL!2).ErbB4(I_III!2,II!1).ErbB3(I_III!3,II!1,Y1328~P).HRG(EGFL!3) Phosphokp47

153 HRG(EGFL!2).ErbB4(I_III!2,II!1).ErbB4(I_III!3,II!1,Y1056~O).HRG(EGFL!3) -> \
  HRG(EGFL!2).ErbB4(I_III!2,II!1).ErbB4(I_III!3,II!1,Y1056~P).HRG(EGFL!3) Phosphokp54

154 HRG(EGFL!2).ErbB4(I_III!2,II!1).ErbB4(I_III!3,II!1,Y1188~O).HRG(EGFL!3) -> \
  HRG(EGFL!2).ErbB4(I_III!2,II!1).ErbB4(I_III!3,II!1,Y1188~P).HRG(EGFL!3) Phosphokp55

155 HRG(EGFL!2).ErbB4(I_III!2,II!1).ErbB4(I_III!3,II!1,Y1242~O).HRG(EGFL!3) -> \
  HRG(EGFL!2).ErbB4(I_III!2,II!1).ErbB4(I_III!3,II!1,Y1242~P).HRG(EGFL!3) Phosphokp56

205 HRG(EGFL!2).ErbB4(I_III!2,II!1).p52Shc1(Y317~O,PTB!4).EGFR(I_III!3,II!1,Y992~P!4).EGF(EGFL!3) -> \
 HRG(EGFL!2).ErbB4(I_III!2,II!1).p52Shc1(Y317~P,PTB!4).EGFR(I_III!3,II!1,Y992~P!4).EGF(EGFL!3)    Shc1kp24

206 HRG(EGFL!2).ErbB4(I_III!2,II!1).p52Shc1(Y317~O,PTB!4).EGFR(I_III!3,II!1,Y1086~P!4).EGF(EGFL!3) -> \
 HRG(EGFL!2).ErbB4(I_III!2,II!1).p52Shc1(Y317~P,PTB!4).EGFR(I_III!3,II!1,Y1086~P!4).EGF(EGFL!3)    Shc1kp25

207 HRG(EGFL!2).ErbB4(I_III!2,II!1).p52Shc1(Y317~O,PTB!4).EGFR(I_III!3,II!1,Y1114~P!4).EGF(EGFL!3) -> \
 HRG(EGFL!2).ErbB4(I_III!2,II!1).p52Shc1(Y317~P,PTB!4).EGFR(I_III!3,II!1,Y1114~P!4).EGF(EGFL!3)    Shc1kp26

208 HRG(EGFL!2).ErbB4(I_III!2,II!1).p52Shc1(Y317~O,PTB!4).ErbB2(II!1,Y1196~P!4) -> \
 HRG(EGFL!2).ErbB4(I_III!2,II!1).p52Shc1(Y317~P,PTB!4).ErbB2(II!1,Y1196~P!4)    Shc1kp27

209 HRG(EGFL!2).ErbB4(I_III!2,II!1).p52Shc1(Y317~O,PTB!4).ErbB2(II!1,Y1222~P!4) -> \
 HRG(EGFL!2).ErbB4(I_III!2,II!1).p52Shc1(Y317~P,PTB!4).ErbB2(II!1,Y1222~P!4)    Shc1kp28

210 HRG(EGFL!2).ErbB4(I_III!2,II!1).p52Shc1(Y317~O,PTB!4).ErbB2(II!1,Y1248~P!4) -> \
 HRG(EGFL!2).ErbB4(I_III!2,II!1).p52Shc1(Y317~P,PTB!4).ErbB2(II!1,Y1248~P!4)    Shc1kp29

211 HRG(EGFL!2).ErbB4(I_III!2,II!1).p52Shc1(Y317~O,PTB!4).ErbB3(I_III!3,II!1,Y1328~P!4).HRG(EGFL!3) -> \
 HRG(EGFL!2).ErbB4(I_III!2,II!1).p52Shc1(Y317~P,PTB!4).ErbB3(I_III!3,II!1,Y1328~P!4).HRG(EGFL!3)    Shc1kp30

212 HRG(EGFL!2).ErbB4(I_III!2,II!1).p52Shc1(Y317~O,PTB!4).ErbB4(I_III!3,II!1,Y1188~P!4).HRG(EGFL!3) -> \
 HRG(EGFL!2).ErbB4(I_III!2,II!1).p52Shc1(Y317~P,PTB!4).ErbB4(I_III!3,II!1,Y1188~P!4).HRG(EGFL!3)    Shc1kp31

213 HRG(EGFL!2).ErbB4(I_III!2,II!1).p52Shc1(Y317~O,PTB!4).ErbB4(I_III!3,II!1,Y1242~P!4).HRG(EGFL!3) -> \
 HRG(EGFL!2).ErbB4(I_III!2,II!1).p52Shc1(Y317~P,PTB!4).ErbB4(I_III!3,II!1,Y1242~P!4).HRG(EGFL!3)    Shc1kp32

399 HRG(EGFL!5).ErbB4(I_III!5,II!1).Gab1(Y447~O,PRS1_PRS2!6).Grb2(cSH3!6,SH2s!2).p52Shc1(Y317~P!2,PTB!4).EGFR(I_III!3,II!1,Y992~P!4).EGF(EGFL!3) -> \
 HRG(EGFL!5).ErbB4(I_III!5,II!1).Gab1(Y447~P,PRS1_PRS2!6).Grb2(cSH3!6,SH2s!2).p52Shc1(Y317~P!2,PTB!4).EGFR(I_III!3,II!1,Y992~P!4).EGF(EGFL!3)    Gab1kp99

400 HRG(EGFL!5).ErbB4(I_III!5,II!1).Gab1(Y472~O,PRS1_PRS2!6).Grb2(cSH3!6,SH2s!2).p52Shc1(Y317~P!2,PTB!4).EGFR(I_III!3,II!1,Y992~P!4).EGF(EGFL!3) -> \
 HRG(EGFL!5).ErbB4(I_III!5,II!1).Gab1(Y472~P,PRS1_PRS2!6).Grb2(cSH3!6,SH2s!2).p52Shc1(Y317~P!2,PTB!4).EGFR(I_III!3,II!1,Y992~P!4).EGF(EGFL!3)    Gab1kp100

401 HRG(EGFL!5).ErbB4(I_III!5,II!1).Gab1(Y619~O,PRS1_PRS2!6).Grb2(cSH3!6,SH2s!2).p52Shc1(Y317~P!2,PTB!4).EGFR(I_III!3,II!1,Y992~P!4).EGF(EGFL!3) -> \
 HRG(EGFL!5).ErbB4(I_III!5,II!1).Gab1(Y619~P,PRS1_PRS2!6).Grb2(cSH3!6,SH2s!2).p52Shc1(Y317~P!2,PTB!4).EGFR(I_III!3,II!1,Y992~P!4).EGF(EGFL!3)    Gab1kp101

402 HRG(EGFL!5).ErbB4(I_III!5,II!1).Gab1(Y657~O,PRS1_PRS2!6).Grb2(cSH3!6,SH2s!2).p52Shc1(Y317~P!2,PTB!4).EGFR(I_III!3,II!1,Y992~P!4).EGF(EGFL!3) -> \
 HRG(EGFL!5).ErbB4(I_III!5,II!1).Gab1(Y657~P,PRS1_PRS2!6).Grb2(cSH3!6,SH2s!2).p52Shc1(Y317~P!2,PTB!4).EGFR(I_III!3,II!1,Y992~P!4).EGF(EGFL!3)    Gab1kp102

403 HRG(EGFL!5).ErbB4(I_III!5,II!1).Gab1(Y447~O,PRS1_PRS2!6).Grb2(cSH3!6,SH2s!2).p52Shc1(Y317~P!2,PTB!4).EGFR(I_III!3,II!1,Y1086~P!4).EGF(EGFL!3) -> \
 HRG(EGFL!5).ErbB4(I_III!5,II!1).Gab1(Y447~P,PRS1_PRS2!6).Grb2(cSH3!6,SH2s!2).p52Shc1(Y317~P!2,PTB!4).EGFR(I_III!3,II!1,Y1086~P!4).EGF(EGFL!3)    Gab1kp103

404 HRG(EGFL!5).ErbB4(I_III!5,II!1).Gab1(Y472~O,PRS1_PRS2!6).Grb2(cSH3!6,SH2s!2).p52Shc1(Y317~P!2,PTB!4).EGFR(I_III!3,II!1,Y1086~P!4).EGF(EGFL!3) -> \
 HRG(EGFL!5).ErbB4(I_III!5,II!1).Gab1(Y472~P,PRS1_PRS2!6).Grb2(cSH3!6,SH2s!2).p52Shc1(Y317~P!2,PTB!4).EGFR(I_III!3,II!1,Y1086~P!4).EGF(EGFL!3)    Gab1kp104

405 HRG(EGFL!5).ErbB4(I_III!5,II!1).Gab1(Y619~O,PRS1_PRS2!6).Grb2(cSH3!6,SH2s!2).p52Shc1(Y317~P!2,PTB!4).EGFR(I_III!3,II!1,Y1086~P!4).EGF(EGFL!3) -> \
 HRG(EGFL!5).ErbB4(I_III!5,II!1).Gab1(Y619~P,PRS1_PRS2!6).Grb2(cSH3!6,SH2s!2).p52Shc1(Y317~P!2,PTB!4).EGFR(I_III!3,II!1,Y1086~P!4).EGF(EGFL!3)    Gab1kp105

406 HRG(EGFL!5).ErbB4(I_III!5,II!1).Gab1(Y657~O,PRS1_PRS2!6).Grb2(cSH3!6,SH2s!2).p52Shc1(Y317~P!2,PTB!4).EGFR(I_III!3,II!1,Y1086~P!4).EGF(EGFL!3) -> \
 HRG(EGFL!5).ErbB4(I_III!5,II!1).Gab1(Y657~P,PRS1_PRS2!6).Grb2(cSH3!6,SH2s!2).p52Shc1(Y317~P!2,PTB!4).EGFR(I_III!3,II!1,Y1086~P!4).EGF(EGFL!3)    Gab1kp106

407 HRG(EGFL!5).ErbB4(I_III!5,II!1).Gab1(Y447~O,PRS1_PRS2!6).Grb2(cSH3!6,SH2s!2).p52Shc1(Y317~P!2,PTB!4).EGFR(I_III!3,II!1,Y1114~P!4).EGF(EGFL!3) -> \
 HRG(EGFL!5).ErbB4(I_III!5,II!1).Gab1(Y447~P,PRS1_PRS2!6).Grb2(cSH3!6,SH2s!2).p52Shc1(Y317~P!2,PTB!4).EGFR(I_III!3,II!1,Y1114~P!4).EGF(EGFL!3)    Gab1kp107

408 HRG(EGFL!5).ErbB4(I_III!5,II!1).Gab1(Y472~O,PRS1_PRS2!6).Grb2(cSH3!6,SH2s!2).p52Shc1(Y317~P!2,PTB!4).EGFR(I_III!3,II!1,Y1114~P!4).EGF(EGFL!3) -> \
 HRG(EGFL!5).ErbB4(I_III!5,II!1).Gab1(Y472~P,PRS1_PRS2!6).Grb2(cSH3!6,SH2s!2).p52Shc1(Y317~P!2,PTB!4).EGFR(I_III!3,II!1,Y1114~P!4).EGF(EGFL!3)    Gab1kp108

409 HRG(EGFL!5).ErbB4(I_III!5,II!1).Gab1(Y619~O,PRS1_PRS2!6).Grb2(cSH3!6,SH2s!2).p52Shc1(Y317~P!2,PTB!4).EGFR(I_III!3,II!1,Y1114~P!4).EGF(EGFL!3) -> \
 HRG(EGFL!5).ErbB4(I_III!5,II!1).Gab1(Y619~P,PRS1_PRS2!6).Grb2(cSH3!6,SH2s!2).p52Shc1(Y317~P!2,PTB!4).EGFR(I_III!3,II!1,Y1114~P!4).EGF(EGFL!3)    Gab1kp109

410 HRG(EGFL!5).ErbB4(I_III!5,II!1).Gab1(Y657~O,PRS1_PRS2!6).Grb2(cSH3!6,SH2s!2).p52Shc1(Y317~P!2,PTB!4).EGFR(I_III!3,II!1,Y1114~P!4).EGF(EGFL!3) -> \
 HRG(EGFL!5).ErbB4(I_III!5,II!1).Gab1(Y657~P,PRS1_PRS2!6).Grb2(cSH3!6,SH2s!2).p52Shc1(Y317~P!2,PTB!4).EGFR(I_III!3,II!1,Y1114~P!4).EGF(EGFL!3)    Gab1kp110

411 HRG(EGFL!5).ErbB4(I_III!5,II!1).Gab1(Y447~O,PRS1_PRS2!6).Grb2(cSH3!6,SH2r!4).EGFR(I_III!3,II!1,Y1068~P!4).EGF(EGFL!3) -> \
 HRG(EGFL!5).ErbB4(I_III!5,II!1).Gab1(Y447~P,PRS1_PRS2!6).Grb2(cSH3!6,SH2r!4).EGFR(I_III!3,II!1,Y1068~P!4).EGF(EGFL!3)    Gab1kp111

412 HRG(EGFL!5).ErbB4(I_III!5,II!1).Gab1(Y472~O,PRS1_PRS2!6).Grb2(cSH3!6,SH2r!4).EGFR(I_III!3,II!1,Y1068~P!4).EGF(EGFL!3) -> \
 HRG(EGFL!5).ErbB4(I_III!5,II!1).Gab1(Y472~P,PRS1_PRS2!6).Grb2(cSH3!6,SH2r!4).EGFR(I_III!3,II!1,Y1068~P!4).EGF(EGFL!3)    Gab1kp112

413 HRG(EGFL!5).ErbB4(I_III!5,II!1).Gab1(Y619~O,PRS1_PRS2!6).Grb2(cSH3!6,SH2r!4).EGFR(I_III!3,II!1,Y1068~P!4).EGF(EGFL!3) -> \
 HRG(EGFL!5).ErbB4(I_III!5,II!1).Gab1(Y619~P,PRS1_PRS2!6).Grb2(cSH3!6,SH2r!4).EGFR(I_III!3,II!1,Y1068~P!4).EGF(EGFL!3)    Gab1kp113

414 HRG(EGFL!5).ErbB4(I_III!5,II!1).Gab1(Y657~O,PRS1_PRS2!6).Grb2(cSH3!6,SH2r!4).EGFR(I_III!3,II!1,Y1068~P!4).EGF(EGFL!3) -> \
 HRG(EGFL!5).ErbB4(I_III!5,II!1).Gab1(Y657~P,PRS1_PRS2!6).Grb2(cSH3!6,SH2r!4).EGFR(I_III!3,II!1,Y1068~P!4).EGF(EGFL!3)    Gab1kp114

415 HRG(EGFL!5).ErbB4(I_III!5,II!1).Gab1(Y447~O,PRS1_PRS2!6).Grb2(cSH3!6,SH2r!4).EGFR(I_III!3,II!1,Y1114~P!4).EGF(EGFL!3) -> \
 HRG(EGFL!5).ErbB4(I_III!5,II!1).Gab1(Y447~P,PRS1_PRS2!6).Grb2(cSH3!6,SH2r!4).EGFR(I_III!3,II!1,Y1114~P!4).EGF(EGFL!3)    Gab1kp115

416 HRG(EGFL!5).ErbB4(I_III!5,II!1).Gab1(Y472~O,PRS1_PRS2!6).Grb2(cSH3!6,SH2r!4).EGFR(I_III!3,II!1,Y1114~P!4).EGF(EGFL!3) -> \
 HRG(EGFL!5).ErbB4(I_III!5,II!1).Gab1(Y472~P,PRS1_PRS2!6).Grb2(cSH3!6,SH2r!4).EGFR(I_III!3,II!1,Y1114~P!4).EGF(EGFL!3)    Gab1kp116

417 HRG(EGFL!5).ErbB4(I_III!5,II!1).Gab1(Y619~O,PRS1_PRS2!6).Grb2(cSH3!6,SH2r!4).EGFR(I_III!3,II!1,Y1114~P!4).EGF(EGFL!3) -> \
 HRG(EGFL!5).ErbB4(I_III!5,II!1).Gab1(Y619~P,PRS1_PRS2!6).Grb2(cSH3!6,SH2r!4).EGFR(I_III!3,II!1,Y1114~P!4).EGF(EGFL!3)    Gab1kp117

418 HRG(EGFL!5).ErbB4(I_III!5,II!1).Gab1(Y657~O,PRS1_PRS2!6).Grb2(cSH3!6,SH2r!4).EGFR(I_III!3,II!1,Y1114~P!4).EGF(EGFL!3) -> \
 HRG(EGFL!5).ErbB4(I_III!5,II!1).Gab1(Y657~P,PRS1_PRS2!6).Grb2(cSH3!6,SH2r!4).EGFR(I_III!3,II!1,Y1114~P!4).EGF(EGFL!3)    Gab1kp118

419 HRG(EGFL!5).ErbB4(I_III!5,II!1).Gab1(Y447~O,PRS1_PRS2!6).Grb2(cSH3!6,SH2r!4).EGFR(I_III!3,II!1,Y1148~P!4).EGF(EGFL!3) -> \
 HRG(EGFL!5).ErbB4(I_III!5,II!1).Gab1(Y447~P,PRS1_PRS2!6).Grb2(cSH3!6,SH2r!4).EGFR(I_III!3,II!1,Y1148~P!4).EGF(EGFL!3)    Gab1kp119

420 HRG(EGFL!5).ErbB4(I_III!5,II!1).Gab1(Y472~O,PRS1_PRS2!6).Grb2(cSH3!6,SH2r!4).EGFR(I_III!3,II!1,Y1148~P!4).EGF(EGFL!3) -> \
 HRG(EGFL!5).ErbB4(I_III!5,II!1).Gab1(Y472~P,PRS1_PRS2!6).Grb2(cSH3!6,SH2r!4).EGFR(I_III!3,II!1,Y1148~P!4).EGF(EGFL!3)    Gab1kp120

421 HRG(EGFL!5).ErbB4(I_III!5,II!1).Gab1(Y619~O,PRS1_PRS2!6).Grb2(cSH3!6,SH2r!4).EGFR(I_III!3,II!1,Y1148~P!4).EGF(EGFL!3) -> \
 HRG(EGFL!5).ErbB4(I_III!5,II!1).Gab1(Y619~P,PRS1_PRS2!6).Grb2(cSH3!6,SH2r!4).EGFR(I_III!3,II!1,Y1148~P!4).EGF(EGFL!3)    Gab1kp121

422 HRG(EGFL!5).ErbB4(I_III!5,II!1).Gab1(Y657~O,PRS1_PRS2!6).Grb2(cSH3!6,SH2r!4).EGFR(I_III!3,II!1,Y1148~P!4).EGF(EGFL!3) -> \
 HRG(EGFL!5).ErbB4(I_III!5,II!1).Gab1(Y657~P,PRS1_PRS2!6).Grb2(cSH3!6,SH2r!4).EGFR(I_III!3,II!1,Y1148~P!4).EGF(EGFL!3)    Gab1kp122

423 HRG(EGFL!5).ErbB4(I_III!5,II!1).Gab1(Y447~O,PRS1_PRS2!6).Grb2(cSH3!6,SH2r!4).EGFR(I_III!3,II!1,Y1173~P!4).EGF(EGFL!3) -> \
 HRG(EGFL!5).ErbB4(I_III!5,II!1).Gab1(Y447~P,PRS1_PRS2!6).Grb2(cSH3!6,SH2r!4).EGFR(I_III!3,II!1,Y1173~P!4).EGF(EGFL!3)    Gab1kp123

424 HRG(EGFL!5).ErbB4(I_III!5,II!1).Gab1(Y472~O,PRS1_PRS2!6).Grb2(cSH3!6,SH2r!4).EGFR(I_III!3,II!1,Y1173~P!4).EGF(EGFL!3) -> \
 HRG(EGFL!5).ErbB4(I_III!5,II!1).Gab1(Y472~P,PRS1_PRS2!6).Grb2(cSH3!6,SH2r!4).EGFR(I_III!3,II!1,Y1173~P!4).EGF(EGFL!3)    Gab1kp124

425 HRG(EGFL!5).ErbB4(I_III!5,II!1).Gab1(Y619~O,PRS1_PRS2!6).Grb2(cSH3!6,SH2r!4).EGFR(I_III!3,II!1,Y1173~P!4).EGF(EGFL!3) -> \
 HRG(EGFL!5).ErbB4(I_III!5,II!1).Gab1(Y619~P,PRS1_PRS2!6).Grb2(cSH3!6,SH2r!4).EGFR(I_III!3,II!1,Y1173~P!4).EGF(EGFL!3)    Gab1kp125

426 HRG(EGFL!5).ErbB4(I_III!5,II!1).Gab1(Y657~O,PRS1_PRS2!6).Grb2(cSH3!6,SH2r!4).EGFR(I_III!3,II!1,Y1173~P!4).EGF(EGFL!3) -> \
 HRG(EGFL!5).ErbB4(I_III!5,II!1).Gab1(Y657~P,PRS1_PRS2!6).Grb2(cSH3!6,SH2r!4).EGFR(I_III!3,II!1,Y1173~P!4).EGF(EGFL!3)    Gab1kp126

427 HRG(EGFL!5).ErbB4(I_III!5,II!1).Gab1(Y447~O,PRS1_PRS2!6).Grb2(cSH3!6,SH2s!2).p52Shc1(Y317~P!2,PTB!4).ErbB2(II!1,Y1196~P!4) -> \
 HRG(EGFL!5).ErbB4(I_III!5,II!1).Gab1(Y447~P,PRS1_PRS2!6).Grb2(cSH3!6,SH2s!2).p52Shc1(Y317~P!2,PTB!4).ErbB2(II!1,Y1196~P!4)    Gab1kp127

428 HRG(EGFL!5).ErbB4(I_III!5,II!1).Gab1(Y472~O,PRS1_PRS2!6).Grb2(cSH3!6,SH2s!2).p52Shc1(Y317~P!2,PTB!4).ErbB2(II!1,Y1196~P!4) -> \
 HRG(EGFL!5).ErbB4(I_III!5,II!1).Gab1(Y472~P,PRS1_PRS2!6).Grb2(cSH3!6,SH2s!2).p52Shc1(Y317~P!2,PTB!4).ErbB2(II!1,Y1196~P!4)    Gab1kp128

429 HRG(EGFL!5).ErbB4(I_III!5,II!1).Gab1(Y619~O,PRS1_PRS2!6).Grb2(cSH3!6,SH2s!2).p52Shc1(Y317~P!2,PTB!4).ErbB2(II!1,Y1196~P!4) -> \
 HRG(EGFL!5).ErbB4(I_III!5,II!1).Gab1(Y619~P,PRS1_PRS2!6).Grb2(cSH3!6,SH2s!2).p52Shc1(Y317~P!2,PTB!4).ErbB2(II!1,Y1196~P!4)    Gab1kp129

430 HRG(EGFL!5).ErbB4(I_III!5,II!1).Gab1(Y657~O,PRS1_PRS2!6).Grb2(cSH3!6,SH2s!2).p52Shc1(Y317~P!2,PTB!4).ErbB2(II!1,Y1196~P!4) -> \
 HRG(EGFL!5).ErbB4(I_III!5,II!1).Gab1(Y657~P,PRS1_PRS2!6).Grb2(cSH3!6,SH2s!2).p52Shc1(Y317~P!2,PTB!4).ErbB2(II!1,Y1196~P!4)    Gab1kp130

431 HRG(EGFL!5).ErbB4(I_III!5,II!1).Gab1(Y447~O,PRS1_PRS2!6).Grb2(cSH3!6,SH2s!2).p52Shc1(Y317~P!2,PTB!4).ErbB2(II!1,Y1222~P!4) -> \
 HRG(EGFL!5).ErbB4(I_III!5,II!1).Gab1(Y447~P,PRS1_PRS2!6).Grb2(cSH3!6,SH2s!2).p52Shc1(Y317~P!2,PTB!4).ErbB2(II!1,Y1222~P!4)    Gab1kp131

432 HRG(EGFL!5).ErbB4(I_III!5,II!1).Gab1(Y472~O,PRS1_PRS2!6).Grb2(cSH3!6,SH2s!2).p52Shc1(Y317~P!2,PTB!4).ErbB2(II!1,Y1222~P!4) -> \
 HRG(EGFL!5).ErbB4(I_III!5,II!1).Gab1(Y472~P,PRS1_PRS2!6).Grb2(cSH3!6,SH2s!2).p52Shc1(Y317~P!2,PTB!4).ErbB2(II!1,Y1222~P!4)    Gab1kp132

433 HRG(EGFL!5).ErbB4(I_III!5,II!1).Gab1(Y619~O,PRS1_PRS2!6).Grb2(cSH3!6,SH2s!2).p52Shc1(Y317~P!2,PTB!4).ErbB2(II!1,Y1222~P!4) -> \
 HRG(EGFL!5).ErbB4(I_III!5,II!1).Gab1(Y619~P,PRS1_PRS2!6).Grb2(cSH3!6,SH2s!2).p52Shc1(Y317~P!2,PTB!4).ErbB2(II!1,Y1222~P!4)    Gab1kp133

434 HRG(EGFL!5).ErbB4(I_III!5,II!1).Gab1(Y657~O,PRS1_PRS2!6).Grb2(cSH3!6,SH2s!2).p52Shc1(Y317~P!2,PTB!4).ErbB2(II!1,Y1222~P!4) -> \
 HRG(EGFL!5).ErbB4(I_III!5,II!1).Gab1(Y657~P,PRS1_PRS2!6).Grb2(cSH3!6,SH2s!2).p52Shc1(Y317~P!2,PTB!4).ErbB2(II!1,Y1222~P!4)    Gab1kp134

435 HRG(EGFL!5).ErbB4(I_III!5,II!1).Gab1(Y447~O,PRS1_PRS2!6).Grb2(cSH3!6,SH2s!2).p52Shc1(Y317~P!2,PTB!4).ErbB2(II!1,Y1248~P!4) -> \
 HRG(EGFL!5).ErbB4(I_III!5,II!1).Gab1(Y447~P,PRS1_PRS2!6).Grb2(cSH3!6,SH2s!2).p52Shc1(Y317~P!2,PTB!4).ErbB2(II!1,Y1248~P!4)    Gab1kp135

436 HRG(EGFL!5).ErbB4(I_III!5,II!1).Gab1(Y472~O,PRS1_PRS2!6).Grb2(cSH3!6,SH2s!2).p52Shc1(Y317~P!2,PTB!4).ErbB2(II!1,Y1248~P!4) -> \
 HRG(EGFL!5).ErbB4(I_III!5,II!1).Gab1(Y472~P,PRS1_PRS2!6).Grb2(cSH3!6,SH2s!2).p52Shc1(Y317~P!2,PTB!4).ErbB2(II!1,Y1248~P!4)    Gab1kp136

437 HRG(EGFL!5).ErbB4(I_III!5,II!1).Gab1(Y619~O,PRS1_PRS2!6).Grb2(cSH3!6,SH2s!2).p52Shc1(Y317~P!2,PTB!4).ErbB2(II!1,Y1248~P!4) -> \
 HRG(EGFL!5).ErbB4(I_III!5,II!1).Gab1(Y619~P,PRS1_PRS2!6).Grb2(cSH3!6,SH2s!2).p52Shc1(Y317~P!2,PTB!4).ErbB2(II!1,Y1248~P!4)    Gab1kp137

438 HRG(EGFL!5).ErbB4(I_III!5,II!1).Gab1(Y657~O,PRS1_PRS2!6).Grb2(cSH3!6,SH2s!2).p52Shc1(Y317~P!2,PTB!4).ErbB2(II!1,Y1248~P!4) -> \
 HRG(EGFL!5).ErbB4(I_III!5,II!1).Gab1(Y657~P,PRS1_PRS2!6).Grb2(cSH3!6,SH2s!2).p52Shc1(Y317~P!2,PTB!4).ErbB2(II!1,Y1248~P!4)    Gab1kp138

439 HRG(EGFL!5).ErbB4(I_III!5,II!1).Gab1(Y447~O,PRS1_PRS2!6).Grb2(cSH3!6,SH2r!4).ErbB2(II!1,Y1139~P!4) -> \
 HRG(EGFL!5).ErbB4(I_III!5,II!1).Gab1(Y447~P,PRS1_PRS2!6).Grb2(cSH3!6,SH2r!4).ErbB2(II!1,Y1139~P!4)    Gab1kp139

440 HRG(EGFL!5).ErbB4(I_III!5,II!1).Gab1(Y472~O,PRS1_PRS2!6).Grb2(cSH3!6,SH2r!4).ErbB2(II!1,Y1139~P!4) -> \
 HRG(EGFL!5).ErbB4(I_III!5,II!1).Gab1(Y472~P,PRS1_PRS2!6).Grb2(cSH3!6,SH2r!4).ErbB2(II!1,Y1139~P!4)    Gab1kp140

441 HRG(EGFL!5).ErbB4(I_III!5,II!1).Gab1(Y619~O,PRS1_PRS2!6).Grb2(cSH3!6,SH2r!4).ErbB2(II!1,Y1139~P!4) -> \
 HRG(EGFL!5).ErbB4(I_III!5,II!1).Gab1(Y619~P,PRS1_PRS2!6).Grb2(cSH3!6,SH2r!4).ErbB2(II!1,Y1139~P!4)    Gab1kp141

442 HRG(EGFL!5).ErbB4(I_III!5,II!1).Gab1(Y657~O,PRS1_PRS2!6).Grb2(cSH3!6,SH2r!4).ErbB2(II!1,Y1139~P!4) -> \
 HRG(EGFL!5).ErbB4(I_III!5,II!1).Gab1(Y657~P,PRS1_PRS2!6).Grb2(cSH3!6,SH2r!4).ErbB2(II!1,Y1139~P!4)    Gab1kp142

443 HRG(EGFL!5).ErbB4(I_III!5,II!1).Gab1(Y447~O,PRS1_PRS2!6).Grb2(cSH3!6,SH2s!2).p52Shc1(Y317~P!2,PTB!4).ErbB3(I_III!3,II!1,Y1328~P!4).HRG(EGFL!3) -> \
 HRG(EGFL!5).ErbB4(I_III!5,II!1).Gab1(Y447~P,PRS1_PRS2!6).Grb2(cSH3!6,SH2s!2).p52Shc1(Y317~P!2,PTB!4).ErbB3(I_III!3,II!1,Y1328~P!4).HRG(EGFL!3)    Gab1kp143

444 HRG(EGFL!5).ErbB4(I_III!5,II!1).Gab1(Y472~O,PRS1_PRS2!6).Grb2(cSH3!6,SH2s!2).p52Shc1(Y317~P!2,PTB!4).ErbB3(I_III!3,II!1,Y1328~P!4).HRG(EGFL!3) -> \
 HRG(EGFL!5).ErbB4(I_III!5,II!1).Gab1(Y472~P,PRS1_PRS2!6).Grb2(cSH3!6,SH2s!2).p52Shc1(Y317~P!2,PTB!4).ErbB3(I_III!3,II!1,Y1328~P!4).HRG(EGFL!3)	Gab1kp144

445 HRG(EGFL!5).ErbB4(I_III!5,II!1).Gab1(Y619~O,PRS1_PRS2!6).Grb2(cSH3!6,SH2s!2).p52Shc1(Y317~P!2,PTB!4).ErbB3(I_III!3,II!1,Y1328~P!4).HRG(EGFL!3) -> \
 HRG(EGFL!5).ErbB4(I_III!5,II!1).Gab1(Y619~P,PRS1_PRS2!6).Grb2(cSH3!6,SH2s!2).p52Shc1(Y317~P!2,PTB!4).ErbB3(I_III!3,II!1,Y1328~P!4).HRG(EGFL!3)    Gab1kp145

446 HRG(EGFL!5).ErbB4(I_III!5,II!1).Gab1(Y657~O,PRS1_PRS2!6).Grb2(cSH3!6,SH2s!2).p52Shc1(Y317~P!2,PTB!4).ErbB3(I_III!3,II!1,Y1328~P!4).HRG(EGFL!3) -> \
 HRG(EGFL!5).ErbB4(I_III!5,II!1).Gab1(Y657~P,PRS1_PRS2!6).Grb2(cSH3!6,SH2s!2).p52Shc1(Y317~P!2,PTB!4).ErbB3(I_III!3,II!1,Y1328~P!4).HRG(EGFL!3)    Gab1kp146

447 HRG(EGFL!5).ErbB4(I_III!5,II!1).Gab1(Y447~O,PRS1_PRS2!6).Grb2(cSH3!6,SH2s!2).p52Shc1(Y317~P!2,PTB!4).ErbB4(I_III!3,II!1,Y1188~P!4).HRG(EGFL!3) -> \
 HRG(EGFL!5).ErbB4(I_III!5,II!1).Gab1(Y447~P,PRS1_PRS2!6).Grb2(cSH3!6,SH2s!2).p52Shc1(Y317~P!2,PTB!4).ErbB4(I_III!3,II!1,Y1188~P!4).HRG(EGFL!3)    Gab1kp147

448 HRG(EGFL!5).ErbB4(I_III!5,II!1).Gab1(Y472~O,PRS1_PRS2!6).Grb2(cSH3!6,SH2s!2).p52Shc1(Y317~P!2,PTB!4).ErbB4(I_III!3,II!1,Y1188~P!4).HRG(EGFL!3) -> \
 HRG(EGFL!5).ErbB4(I_III!5,II!1).Gab1(Y472~P,PRS1_PRS2!6).Grb2(cSH3!6,SH2s!2).p52Shc1(Y317~P!2,PTB!4).ErbB4(I_III!3,II!1,Y1188~P!4).HRG(EGFL!3)	Gab1kp148

449 HRG(EGFL!5).ErbB4(I_III!5,II!1).Gab1(Y619~O,PRS1_PRS2!6).Grb2(cSH3!6,SH2s!2).p52Shc1(Y317~P!2,PTB!4).ErbB4(I_III!3,II!1,Y1188~P!4).HRG(EGFL!3) -> \
 HRG(EGFL!5).ErbB4(I_III!5,II!1).Gab1(Y619~P,PRS1_PRS2!6).Grb2(cSH3!6,SH2s!2).p52Shc1(Y317~P!2,PTB!4).ErbB4(I_III!3,II!1,Y1188~P!4).HRG(EGFL!3)    Gab1kp149

450 HRG(EGFL!5).ErbB4(I_III!5,II!1).Gab1(Y657~O,PRS1_PRS2!6).Grb2(cSH3!6,SH2s!2).p52Shc1(Y317~P!2,PTB!4).ErbB4(I_III!3,II!1,Y1188~P!4).HRG(EGFL!3) -> \
 HRG(EGFL!5).ErbB4(I_III!5,II!1).Gab1(Y657~P,PRS1_PRS2!6).Grb2(cSH3!6,SH2s!2).p52Shc1(Y317~P!2,PTB!4).ErbB4(I_III!3,II!1,Y1188~P!4).HRG(EGFL!3)    Gab1kp150

451 HRG(EGFL!5).ErbB4(I_III!5,II!1).Gab1(Y447~O,PRS1_PRS2!6).Grb2(cSH3!6,SH2s!2).p52Shc1(Y317~P!2,PTB!4).ErbB4(I_III!3,II!1,Y1242~P!4).HRG(EGFL!3) -> \
 HRG(EGFL!5).ErbB4(I_III!5,II!1).Gab1(Y447~P,PRS1_PRS2!6).Grb2(cSH3!6,SH2s!2).p52Shc1(Y317~P!2,PTB!4).ErbB4(I_III!3,II!1,Y1242~P!4).HRG(EGFL!3)    Gab1kp151

452 HRG(EGFL!5).ErbB4(I_III!5,II!1).Gab1(Y472~O,PRS1_PRS2!6).Grb2(cSH3!6,SH2s!2).p52Shc1(Y317~P!2,PTB!4).ErbB4(I_III!3,II!1,Y1242~P!4).HRG(EGFL!3) -> \
 HRG(EGFL!5).ErbB4(I_III!5,II!1).Gab1(Y472~P,PRS1_PRS2!6).Grb2(cSH3!6,SH2s!2).p52Shc1(Y317~P!2,PTB!4).ErbB4(I_III!3,II!1,Y1242~P!4).HRG(EGFL!3)	Gab1kp152

453 HRG(EGFL!5).ErbB4(I_III!5,II!1).Gab1(Y619~O,PRS1_PRS2!6).Grb2(cSH3!6,SH2s!2).p52Shc1(Y317~P!2,PTB!4).ErbB4(I_III!3,II!1,Y1242~P!4).HRG(EGFL!3) -> \
 HRG(EGFL!5).ErbB4(I_III!5,II!1).Gab1(Y619~P,PRS1_PRS2!6).Grb2(cSH3!6,SH2s!2).p52Shc1(Y317~P!2,PTB!4).ErbB4(I_III!3,II!1,Y1242~P!4).HRG(EGFL!3)    Gab1kp153

454 HRG(EGFL!5).ErbB4(I_III!5,II!1).Gab1(Y657~O,PRS1_PRS2!6).Grb2(cSH3!6,SH2s!2).p52Shc1(Y317~P!2,PTB!4).ErbB4(I_III!3,II!1,Y1242~P!4).HRG(EGFL!3) -> \
 HRG(EGFL!5).ErbB4(I_III!5,II!1).Gab1(Y657~P,PRS1_PRS2!6).Grb2(cSH3!6,SH2s!2).p52Shc1(Y317~P!2,PTB!4).ErbB4(I_III!3,II!1,Y1242~P!4).HRG(EGFL!3)    Gab1kp154
}}}
```

```
''~ErbB4 homodimerization'' (see Arrow  6 in [[Contact Map]])
{{{ErbB4(II)+ErbB4(II)<->ErbB4(II!1).ErbB4(II!1)}}}
!Rules
{{{
50 ErbB4(I_III,II,loc~M) + ErbB4(I_III,II,loc~M) <-> \
 ErbB4(I_III,II!1,loc~M).ErbB4(I_III,II!1,loc~M)       Dimkp26,Dimkm26

51 ErbB4(I_III!1,II,loc~M).HRG(EGFL!1,loc~Ex) + ErbB4(I_III,II,loc~M) <-> \
 ErbB4(I_III!1,II!2,loc~M).HRG(EGFL!1,loc~Ex).ErbB4(I_III,II!2,loc~M)     Dimkp27,Dimkm27

52 ErbB4(I_III!1,II,loc~M).HRG(EGFL!1,loc~Ex) + ErbB4(I_III!2,II,loc~M).HRG(EGFL!2,loc~Ex) <-> \
 ErbB4(I_III!1,II!3,loc~M).HRG(EGFL!1,loc~Ex).ErbB4(I_III!2,II!3,loc~M).HRG(EGFL!2,loc~Ex)   Dimkp28,Dimkm28
}}}
```

```
''HRG reversibly binds ~ErbB4'' (see Arrow 2 in [[Contact Map]])
{{{HRG(EGFL)+ErbB4(I_III)<->HRG(EGFL!1).ErbB4(I_III!1)}}}
!Rules
{{{
3 ErbB4(I_III,II,loc~M) + HRG(EGFL,deg~F,loc~Ex) <-> \
 ErbB4(I_III!1,II,loc~M).HRG(EGFL!1,deg~F,loc~Ex)     HRG4kp1,HRG4km1

12 EGFR(I_III,II!1,loc~M).ErbB4(I_III,II!1,loc~M) + HRG(EGFL,deg~F,loc~Ex) <-> \
 EGFR(I_III,II!1,loc~M).ErbB4(I_III!2,II!1,loc~M).HRG(EGFL!2,deg~F,loc~Ex)     HRG4kp2,HRG4km2

13 EGF(EGFL!2,deg~F,loc~Ex).EGFR(I_III!2,II!1,loc~M).ErbB4(I_III,II!1,loc~M) + HRG(EGFL,deg~F,loc~Ex) <-> \
 EGF(EGFL!2,deg~F,loc~Ex).EGFR(I_III!2,II!1,loc~M).ErbB4(I_III!3,II!1,loc~M).HRG(EGFL!3,deg~F,loc~Ex)     HRG4kp3,HRG4km3

16 ErbB2(II!1,loc~M).ErbB4(I_III,II!1,loc~M) + HRG(EGFL,deg~F,loc~Ex) <-> \
 ErbB2(II!1,loc~M).ErbB4(I_III!2,II!1,loc~M).HRG(EGFL!2,deg~F,loc~Ex)     HRG4kp2,HRG4km2

19 ErbB3(I_III,II!1,loc~M).ErbB4(I_III,II!1,loc~M) + HRG(EGFL,deg~F,loc~Ex) <-> \
 ErbB3(I_III,II!1,loc~M).ErbB4(I_III!2,II!1,loc~M).HRG(EGFL!2,deg~F,loc~Ex)     HRG4kp2,HRG4km2

21 HRG(EGFL!2,deg~F,loc~Ex).ErbB3(I_III!2,II!1,loc~M).ErbB4(I_III,II!1,loc~M) + HRG(EGFL,deg~F,loc~Ex) <-> \
 HRG(EGFL!2,deg~F,loc~Ex).ErbB3(I_III!2,II!1,loc~M).ErbB4(I_III!3,II!1,loc~M).HRG(EGFL!3,deg~F,loc~Ex)     HRG4kp3,HRG4km3

23 ErbB4(I_III,II!1,loc~M).ErbB4(I_III,II!1,loc~M) + HRG(EGFL,deg~F,loc~Ex) <-> \
 ErbB4(I_III,II!1,loc~M).ErbB4(I_III!2,II!1,loc~M).HRG(EGFL!2,deg~F,loc~Ex)     HRG4kp2,HRG4km2

24 HRG(EGFL!2,deg~F,loc~Ex).ErbB4(I_III!2,II!1,loc~M).ErbB4(I_III,II!1,loc~M) + HRG(EGFL,deg~F,loc~Ex) <-> \
 HRG(EGFL!2,deg~F,loc~Ex).ErbB4(I_III!2,II!1,loc~M).ErbB4(I_III!3,II!1,loc~M).HRG(EGFL!3,deg~F,loc~Ex)     HRG4kp3,HRG4km3
}}}
```

```
''~PI3K reversibly binds~ErbB4'' (see Arrow 21 in [[Contact Map]])
{{{PI3K(R_p85_nSH2_cSH2)+ErbB4(Y1056~P)<->PI3K(R_p85_nSH2_cSH2!1).ErbB4(Y1056~P!1)}}}
!Rules
{{{
511 PI3K(R_p85_nSH2_cSH2,G_p85_nSH2_cSH2) + ErbB4(Y1056~P) <-> PI3K(R_p85_nSH2_cSH2!1,G_p85_nSH2_cSH2).ErbB4(Y1056~P!1)	PI3Kkp13,PI3Kkm13
}}}
```

```
''Grb2 reversibly binds EGFR, ~ErbB2, and ~ErbB3'' (see Arrow 11 in [[Contact Map]])
{{{Grb2(SH2)+EGFR(Y1068~P)<->Grb2(SH2!1).EGFR(Y1068~P!1)}}}
{{{Grb2(SH2)+EGFR(Y1086~P)<->Grb2(SH2!1).EGFR(Y1086~P!1)}}}
{{{Grb2(SH2)+ErbB2(Y1139~P)<->Grb2(SH2!1).EGFR(Y1139~P!1)}}}
{{{Grb2(SH2)+ErbB3(Y1194~P)<->Grb2(SH2!1).EGFR(Y1194~P!1)}}}
{{{Grb2(SH2)+ErbB3(Y1257~P)<->Grb2(SH2!1).EGFR(Y1257~P!1)}}}
!Rules
{{{
176 EGFR(Y1068~P) + Grb2(SH2r,SH2s) <-> \
 EGFR(Y1068~P!1).Grb2(SH2r!1,SH2s) Grb2kp1,Grb2km1

177 EGFR(Y1114~P) + Grb2(SH2r,SH2s) <-> \
 EGFR(Y1114~P!1).Grb2(SH2r!1,SH2s) Grb2kp2,Grb2km2

178 EGFR(Y1148~P) + Grb2(SH2r,SH2s) <-> \
 EGFR(Y1148~P!1).Grb2(SH2r!1,SH2s) Grb2kp3,Grb2km3

179 EGFR(Y1173~P) + Grb2(SH2r,SH2s) <-> \
 EGFR(Y1173~P!1).Grb2(SH2r!1,SH2s) Grb2kp4,Grb2km4

180 ErbB2(Y1139~P) + Grb2(SH2r,SH2s) <-> \
 ErbB2(Y1139~P!1).Grb2(SH2r!1,SH2s)         Grb2kp5,Grb2km5
}}}
```

```
''p52Shc1 reversibly binds EGFF, ~ErbB2, and ~ErbB3'' (see Arrow 12 in [[Contact Map]])
{{{p52Shc1(PTB)+EGFR(Y1148~P)<->p52Shc1(PTB!1).EGFR(Y1148~P!1)}}}
{{{p52Shc1(PTB)+EGFR(Y1173~P)<->p52Shc1(PTB!1).EGFR(Y1173~P!1)}}}
{{{p52Shc1(PTB)+ErbB2(Y1139~P)<->p52Shc1(PTB!1).ErbB2(Y1139~P!1)}}}
{{{p52Shc1(PTB)+ErbB2(Y1222~P)<->p52Shc1(PTB!1).ErbB2(Y1222~P!1)}}}
{{{p52Shc1(PTB)+ErbB3(Y1325~P)<->p52Shc1(PTB!1).ErbB3(Y1325~P!1)}}}
!Rules
{{{
181 EGFR(Y992~P) + p52Shc1(PTB) <-> \
 EGFR(Y992~P!1).p52Shc1(PTB!1)       Shc1kp0,Shc1km0

182 EGFR(Y1086~P) + p52Shc1(PTB) <-> \
 EGFR(Y1086~P!1).p52Shc1(PTB!1)       Shc1kp1,Shc1km1

183 EGFR(Y1114~P) + p52Shc1(PTB) <-> \
 EGFR(Y1114~P!1).p52Shc1(PTB!1)       Shc1kp2,Shc1km2

184 ErbB2(Y1196~P) + p52Shc1(PTB) <-> \
 ErbB2(Y1196~P!1).p52Shc1(PTB!1)       Shc1kp3,Shc1km3

185 ErbB2(Y1222~P) + p52Shc1(PTB) <-> \
 ErbB2(Y1222~P!1).p52Shc1(PTB!1)       Shc1kp4,Shc1km4

186 ErbB2(Y1248~P) + p52Shc1(PTB) <-> \
 ErbB2(Y1248~P!1).p52Shc1(PTB!1)       Shc1kp5,Shc1km5

187 ErbB3(Y1328~P) + p52Shc1(PTB) <-> \
 ErbB3(Y1328~P!1).p52Shc1(PTB!1)       Shc1kp6,Shc1km6

188 ErbB4(Y1188~P) + p52Shc1(PTB) <-> \
 ErbB4(Y1188~P!1).p52Shc1(PTB!1)       Shc1kp7,Shc1km7

189 ErbB4(Y1242~P) + p52Shc1(PTB) <-> \
 ErbB4(Y1242~P!1).p52Shc1(PTB!1)       Shc1kp8,Shc1km8
}}}
```

```
A complete listing of the ~BioNetGen model-specification file {{{ErbB_model.bngl}}} is given below.

{{{
begin parameters

NA 6.0221415e23 # Avogadro's number (molecues/mol)
Vo 1.0e-10 # Extracellular volume=1/cell_density (L)

#Initial values

EGF_tot 		5*1.0e-9*NA*Vo
HRG_tot 		5*1.0e-9*NA*Vo
EGFR_tot        4.27672520173064e5
ErbB2_tot       1.42093237149675e4
ErbB3_tot       1.74843554666751e5
ErbB4_tot       8.91594202782983e4
p52Shc1_tot 	6.01531854462274e4
Grb2_tot        5.91055062496997e4
Sos1_tot 		4.09301535229042e4
Gab1_tot        4.80466223642856e4
PI3K_tot        3.56731320547328e5
PDK1_tot		8.92880892502848e5
Akt1_tot 		3.4017537819372e5
RAS_tot 		8.65718002843658e5
p120RasGAP_tot	9.32786886419808e4
Raf1_tot 		4.80188361349246e4
MEK1_tot 		6.63956495470586e4
ERK2_tot 		6.16394706849877e5


#Setting K values

EGFequil1 = 1.82539211740452e-6  # EGFkp1/EGFkm1
EGFequil2 = 3.24395882030410e-6 # EGFkp2/EGFkm2
EGFequil3 = 2.88434511422028e-6 # EGFkp3/EGFkm3

HRG3equil1 = 2.7585877061741e-6 # HRG3kp1/HRG3km1
HRG3equil2 = 3.4169236535230e-5 # HRG3kp2/HRG3km2
HRG3equil3 = 3.03813568286261e-5 # HRG3kp3/HRG3km3

HRG4equil1 =  3.55574858868965e-6# HRG4kp1/HRG4km1
HRG4equil2 =  3.58838215596531e-5# HRG4kp2/HRG4km2
HRG4equil3 =  3.19058690718630e-5# HRG4kp3/HRG4km3

EGFkp1		0 #1.6752764844066e-6
EGFkp2		0 #1.31766379762354e-6
EGFkp3		0 #2.82831275074602e-7

EGFkm1		9.17762528080067e-1
EGFkm2		4.06190050680118e-1
EGFkm3		9.80573627199459e-2

HRG3kp1		0 #2.25177730824231e-7
HRG3kp2		0 #5.48266629995157e-6
HRG3kp3		0 #1.56149921651503e-5 #HRG3km3*EGFequil3*HRG3equil2/EGFequil2

HRG3km1		8.16279034087813e-2
HRG3km2		1.60456213128985e-1
HRG3km3		5.13966254148249e-1

HRG4kp1		0 #2.88261678079618e-6
HRG4kp2		0 #5.1027110057525e-6
HRG4kp3		0 #7.73131997833758e-6 #HRG4km3*EGFequil3*HRG4equil2/EGFequil2

HRG4km1		8.10691956671343e-1
HRG4km2		1.422008800615e-1
HRG4km3		2.42316545614977e-1

DimEquil1 = Dimkp1/Dimkm1
DimEquil2 = Dimkp2/Dimkm2
DimEquil4 = Dimkp4/Dimkm4
DimEquil6 = Dimkp6/Dimkm6
DimEquil7 = Dimkp7/Dimkm7
DimEquil10 = Dimkp10/Dimkm10
DimEquil11 = Dimkp11/Dimkm11
DimEquil15 = Dimkp15/Dimkm15
DimEquil17 = Dimkp17/Dimkm17
DimEquil19 = Dimkp19/Dimkm19
DimEquil20 = Dimkp20/Dimkm20
DimEquil22 = Dimkp22/Dimkm22
DimEquil23 = Dimkp23/Dimkm23
DimEquil26 = Dimkp26/Dimkm26
DimEquil27 = Dimkp27/Dimkm27

Dimkp1		5.0959890629278e-6
Dimkp2		Dimkm2*DimEquil1*EGFequil2/EGFequil1
Dimkp3		Dimkm3*DimEquil2*EGFequil3/EGFequil1
Dimkp4		3.55124048645837e-7
Dimkp5		Dimkm5*DimEquil4*EGFequil2/EGFequil1
Dimkp6		5.93863482345085e-7
Dimkp7		Dimkm7*DimEquil6*EGFequil2/EGFequil1
Dimkp8		Dimkm8*DimEquil6*HRG3equil2/HRG3equil1
Dimkp9		Dimkm9*DimEquil7*EGFequil3/EGFequil1
Dimkp10		5.95858524293305e-6
Dimkp11		Dimkm11*DimEquil10*EGFequil2/EGFequil1
Dimkp12		Dimkm12*DimEquil10*HRG4equil2/HRG4equil1
Dimkp13		Dimkm13*DimEquil11*EGFequil3/EGFequil1
Dimkp14		9.40444138151105e-7
Dimkp15		8.64565481767477e-6
Dimkp16		Dimkm16*DimEquil15*HRG3equil2/HRG3equil1
Dimkp17		6.60964194053378e-7
Dimkp18		Dimkm18*DimEquil17*HRG4equil2/HRG4equil1
Dimkp19		2.34931041558104e-6
Dimkp20		Dimkm20*DimEquil19*HRG3equil2/HRG3equil1
Dimkp21		Dimkm21*DimEquil20*HRG3equil3/HRG3equil1
Dimkp22		9.44327814569465e-6
Dimkp23		Dimkm23*DimEquil22*HRG3equil2/HRG3equil1
Dimkp24		Dimkm24*DimEquil22*HRG4equil2/HRG4equil1
Dimkp25		Dimkm25*DimEquil23*HRG3equil3/HRG3equil1
Dimkp26		2.43135131855894e-6
Dimkp27		Dimkm27*DimEquil26*HRG4equil2/HRG4equil1
Dimkp28		Dimkm28*DimEquil27*HRG4equil3/HRG4equil1


Dimkm1		6.82335881150971e-1
Dimkm2		8.60446362688242e-1
Dimkm3		5.30901820046591e-1
Dimkm4		3.2906848719399e-2
Dimkm5		3.61328871705183e-1
Dimkm6		2.91209182767864e-2
Dimkm7		4.10248490881162e-2
Dimkm8		7.06334928569754e-1
Dimkm9		5.064620571136e-1
Dimkm10		5.4868243150637e-2
Dimkm11		2.09675101809484e-1
Dimkm12		2.87423426816554e-1
Dimkm13		5.48638129462967e-1
Dimkm14		5.35056553608581e-2
Dimkm15		4.45127253423715e-1
Dimkm16		2.24099123790756e-1
Dimkm17		3.39468196721401e-1
Dimkm18		7.43735228789512e-2
Dimkm19		1.51427204579608e-2
Dimkm20		6.47926498517387e-2
Dimkm21		7.82869781142506e-2
Dimkm22		8.48650355450826e-1
Dimkm23		6.61204708354308e-1
Dimkm24		9.19541811794608e-1
Dimkm25		3.37299538300942e-2
Dimkm26		2.04189992563573e-1
Dimkm27		7.29286884022918e-1
Dimkm28		7.43108065474286e-1


Intkp1		8.32705126548996e-2
Intkp15		5.86246294682792e-3

iLigkp1		8.93979864006803e-1
iLigkp2		7.56616609711335e-1

iLigkp3		1.40929275291501e-2
iLigkp4		6.46661221517235e-2

iDimkp1		3.5207168888922e-2
iDimkp2		9.17315380364937e-1
iDimkp3		7.93734840774044e-1
iDimkp4		9.78655334522932e-2
iDimkp5		3.65206163171712e-1
iDimkp6		6.86255010679577e-1
iDimkp7		1.99168704665789e-1
iDimkp8		9.6335351868629e-2
iDimkp9		7.87528140525939e-1
iDimkp10	1.66421484864486e-2

Phosphokp1		8.88772107233018e-1
Phosphokp2		4.2878246307489e-1
Phosphokp3		1.95396201245302e-2
Phosphokp4		2.00161517127765e-1
Phosphokp5		7.06003182769046e-1
Phosphokp6		7.6616659390412e-2
Phosphokp7		1.80218044939915e-1
Phosphokp8		4.34778121114391e-1
Phosphokp9		6.39089429079519e-1
Phosphokp10		5.9756050915588e-2
Phosphokp11		2.53799737608887e-1
Phosphokp12		5.63302566441462e-2
Phosphokp13		2.82790620799852e-1
Phosphokp14		1.22324552773364e-1
Phosphokp15		9.67835541778362e-1
Phosphokp16		3.51414620272385e-2
Phosphokp17		5.21022165483879e-2
Phosphokp18		3.1157068215756e-2
Phosphokp19		8.443340013154e-2
Phosphokp20		6.80116300490647e-2
Phosphokp21		4.51367418859485e-1
Phosphokp22		7.74887216709453e-2
Phosphokp23		4.60083703581773e-1
Phosphokp24		2.05792755059326e-1
Phosphokp25		1.9661113965264e-2
Phosphokp26		2.95134086701501e-1
Phosphokp27		9.07050520292188e-1
Phosphokp28		1.21049084190338e-2
Phosphokp29		9.50488082789428e-2
Phosphokp30		9.35460962603844e-2
Phosphokp31		6.54846548500461e-2
Phosphokp32		3.94313272466951e-1
Phosphokp33		3.14072666810171e-1
Phosphokp34		8.45442806483862e-1
Phosphokp35		6.99553162146515e-1
Phosphokp36		3.93438369234849e-2
Phosphokp37		1.4547403805293e-2
Phosphokp38		1.17107729876903e-2
Phosphokp39		4.02838224075356e-2
Phosphokp40		2.78079467363796e-2
Phosphokp41		6.21195843955167e-2
Phosphokp42		3.84566849273881e-2
Phosphokp43		6.53045295503028e-2
Phosphokp44		5.56999926968433e-1
Phosphokp45		1.5472022436839e-2
Phosphokp46		2.78694500019026e-2
Phosphokp47		5.72714409115287e-2
Phosphokp48		7.71759002007548e-1
Phosphokp49		6.01611442493851e-2
Phosphokp50		4.87347060541873e-2
Phosphokp51		4.35687421786884e-2
Phosphokp52		1.18362096686366e-2
Phosphokp53		6.36787651207112e-1
Phosphokp54		3.54646060845124e-1
Phosphokp55		5.90317591027224e-1
Phosphokp56		8.4467134352829e-2

Phosphokm1		7.26993260956493e-3
Phosphokm2		9.74974910327381e-2
Phosphokm3		2.68584632202874e-1
Phosphokm4		5.50094046126816e-1
Phosphokm5		7.67126639405035e-1
Phosphokm6		2.75612797520029e-3
Phosphokm7		6.85030301720076e-3
Phosphokm8		4.87439992623973e-2
Phosphokm9		5.72269724841397e-1
Phosphokm10		9.44031999570728e-1
Phosphokm11		8.81635543298421e-2
Phosphokm12		6.65205478076501e-2
Phosphokm13		7.3601652035394e-2
Phosphokm14		1.5084976290579e-1
Phosphokm15		2.74034930234802e-2
Phosphokm16		4.79850299988658e-2
Phosphokm17		3.67233415392832e-3
Phosphokm18		7.13755713699078e-2
Phosphokm19		1.20814195649541e-2
Phosphokm20		5.91549834797695e-2

Grb2kp1		9.48751042996452e-6
Grb2kp2		4.49259489482017e-7
Grb2kp3		6.03697072678887e-6
Grb2kp4		7.26714190078701e-7
Grb2kp5		7.80974367513346e-6

Grb2km1		6.74470404588556e-2
Grb2km2		3.21266715315215e-1
Grb2km3		9.2695034884224e-1
Grb2km4		1.27959374406095e-2
Grb2km5		7.21220480153556e-1

Shc1kp0		1.97883551485319e-7
Shc1kp1		4.57411536575194e-6
Shc1kp2		4.74191085720764e-6
Shc1kp3		3.28710008853551e-6
Shc1kp4		4.34278757626282e-7
Shc1kp5		4.57221757547808e-6
Shc1kp6		5.72021770430524e-6
Shc1kp7		3.23935077662424e-7
Shc1kp8		9.25660200452455e-6

Shc1km0		4.49575735437162e-2
Shc1km1		6.67230763160359e-2
Shc1km2		5.79656246540662e-2
Shc1km3		7.47800677001061e-1
Shc1km4		9.51265970211182e-1
Shc1km5		6.50096102370838e-1
Shc1km6		1.55457060924141e-1
Shc1km7		1.24736961289233e-1
Shc1km8		3.83019454603652e-1

Shc1kp9		5.69833036091512e-1
Shc1kp10	5.59482986809144e-1
Shc1kp11	2.42858250499268e-1
Shc1kp12	1.5443440933692e-2
Shc1kp13		9.89657844783909e-1
Shc1kp14		1.53725031821727e-2
Shc1kp15		4.22356483806294e-2
Shc1kp16		7.44568988338312e-1
Shc1kp17		1.14399259149258e-2
Shc1kp18		6.63532129002988e-1
Shc1kp19		8.5178640861298e-2
Shc1kp20		5.94148145316552e-1
Shc1kp21		2.0700566805906e-1
Shc1kp22		6.23710111799534e-1
Shc1kp23		2.63775121304968e-1
Shc1kp24		5.32149459123519e-1
Shc1kp25		9.49394026611117e-1
Shc1kp26		4.953415509737e-1
Shc1kp27		7.6819206332508e-1
Shc1kp28		4.85997292760425e-1
Shc1kp29		7.82487101445372e-1
Shc1kp30		3.00808468223011e-1
Shc1kp31		2.65779406020073e-2
Shc1kp32		5.29010839763077e-2


Shc1km14	8.88151462022222e-2

Scafoldkp1	7.81993810926309e-7
Scafoldkp2	5.2905293019769e-6
Scafoldkp3	5*Scafoldkp2

Scafoldkm1	3.39662220127485e-1
Scafoldkm2	6.38794564439524e-2
Scafoldkm3	Scafoldkm2


Sos1kp1		2.83307444159129e-6
Sos1kp3		1.10049191751608e-6

Sos1kp5		2.32751420105001e-2
Sos1kp6		5*Sos1kp5
Sos1kp7		3.68445191357574e-2
Sos1kp8		1.58767045993943e-1
Sos1kp9		7.79377967231056e-2

Sos1km5		3.82133045165284e-2
Sos1km6		5*Sos1km5


MAPKkp1		2.34858647510049e-6
MAPKkp2		7.97187030086059e-1
MAPKkp3		3.76471948496902e-1
MAPKkp4		8.40146299281042e-1
MAPKkp5		8.65474093754534e-1
MAPKkp6		8.25953667549936e-2
MAPKkp7		7.41979697938558e-2
MAPKkp8		1.37135935836113e-3
MAPKkp9		8.18059543701685e-7
MAPKkp10	8.54707689482359e-6
MAPKkp11	9.11852147803069e-2
MAPKkp12	9.50469680832043e-1
MAPKkp13	4.20150542888183e-2
MAPKkp15	2.33197003501971e-2
MAPKkp16	8.50946608676458e-7
MAPKkp17	4.62874983163874e-1
MAPKkp18	3.35431298757868e-2
MAPKkp19	3.32559533601363e-1
MAPKkp20	2.09334798311488e-2
MAPKkp21	3.06698724199856e-7
MAPKkp22	8.88161417106301e-6
MAPKkp23	7.08681212084929e-6
MAPKkp24	4.08615417714418e-7
MAPKkp25	4.90729612515253e-6
MAPKkp26	1.84517732382585e-6
MAPKkp27	2.24786074034722e-6
MAPKkp28	1.96454339477236e-7
MAPKkp29	2.22526846482702e-7
MAPKkp30	1.90968360095593e-6
MAPKkp31	7.79954799619554e-7
MAPKkp32	7.75963929608389e-7
MAPKkp33	2.59665864412602e-2
MAPKkp34	2.43591083356245e-2
MAPKkp35	7.16980303326022e-2
MAPKkp36	2.49429517376035e-1
MAPKkp37	8.55283324348136e-2
MAPKkp38	4.45729674507919e-2
MAPKkp39	4.2054915619633e-1
MAPKkp40	7.34095564918618e-2
MAPKkp41	1.10316513094398e-2
MAPKkp42	5.88228995495206e-2
MAPKkp43	9.61443776159791e-1
MAPKkp44	5.54780338440048e-2
MAPKkp45	4.48285458762001e-3
MAPKkp46	1.80883788097742e-2
MAPKkp47	4.99203801450802e-3
MAPKkp48	8.31336309299819e-3
MAPKkp49	8.56531321402417e-2
MAPKkp50	4.51531525768462e-1
MAPKkp51	2.41716382509588e-2
MAPKkp52	4.80306028262771e-1


Gab1kp1		1.96995906486126e-6
Gab1kp3		1.26884248252077e-1
Gab1kp4		6.59955402545501e-2
Gab1kp5		2.74649622535037e-1
Gab1kp6		4.3149249204576e-2
Gab1kp7		7.43735567137946e-1
Gab1kp8		5.09433307106832e-1
Gab1kp9		7.84981187122992e-1
Gab1kp10		9.58526974679023e-2
Gab1kp11		6.89178573211149e-1
Gab1kp12		9.68556225363977e-1
Gab1kp13		9.43317263519674e-2
Gab1kp14		4.94750548020704e-2
Gab1kp15		8.06143925513339e-2
Gab1kp16		2.19269118931788e-1
Gab1kp17		1.89942763529712e-2
Gab1kp18		1.89528365333723e-1
Gab1kp19		6.03030348115124e-2
Gab1kp20		3.54071892177262e-2
Gab1kp21		3.99232860454623e-1
Gab1kp22		5.63319907552716e-2
Gab1kp23		3.10843424122953e-2
Gab1kp24		6.84002889697763e-2
Gab1kp25		6.39876833170325e-1
Gab1kp26		9.82736084289934e-2
Gab1kp27		9.18433441172982e-2
Gab1kp28		3.82822878829575e-1
Gab1kp29		8.21161423705763e-1
Gab1kp30		9.20446819241408e-2
Gab1kp31		2.21644107443666e-1
Gab1kp32		3.63754251476098e-2
Gab1kp33		2.3667792426341e-2
Gab1kp34		4.44827588111809e-2
Gab1kp35		3.08440990344997e-1
Gab1kp36		5.54549199955773e-2
Gab1kp37		7.74552310273276e-1
Gab1kp38		7.50355932645846e-2
Gab1kp39		5.76021606139506e-2
Gab1kp40		4.53089771642914e-1
Gab1kp41		8.99505991878266e-2
Gab1kp42		3.78959951192263e-1
Gab1kp43		6.50754783263528e-1
Gab1kp44		6.81419244007412e-1
Gab1kp45		1.89643033383857e-1
Gab1kp46		1.02351033220879e-2
Gab1kp47		4.73869586053684e-1
Gab1kp48		2.45137939979401e-1
Gab1kp49		7.44917984527444e-1
Gab1kp50		1.26045363101139e-2
Gab1kp51		3.16955880399047e-1
Gab1kp52		4.33161963713894e-2
Gab1kp53		5.17343683338817e-2
Gab1kp54		7.48601277549528e-2
Gab1kp55		6.92340425584058e-1
Gab1kp56		4.07780811638111e-2
Gab1kp57		1.40041104876053e-2
Gab1kp58		9.48753480457526e-2
Gab1kp59		7.5016080657968e-1
Gab1kp60		3.66672032784976e-2
Gab1kp61		1.79009642048845e-2
Gab1kp62		6.38332656408699e-2
Gab1kp63		6.84007275429656e-2
Gab1kp64		1.0710450029589e-1
Gab1kp65		5.41563482962238e-2
Gab1kp66		8.84462541321437e-1
Gab1kp67		7.47350965386988e-1
Gab1kp68		5.65594739491638e-2
Gab1kp69		7.17689305501494e-1
Gab1kp70		1.79330734762095e-1
Gab1kp71		6.88931712557604e-1
Gab1kp72		9.22136524885445e-2
Gab1kp73		2.98344535037069e-1
Gab1kp74		7.64644004450076e-2
Gab1kp75		9.3958164303171e-2
Gab1kp76		8.8252420049796e-1
Gab1kp77		2.65565223728305e-1
Gab1kp78		8.4993010543607e-1
Gab1kp79		9.93832547054416e-1
Gab1kp80		2.85970169779949e-2
Gab1kp81		8.60664808545255e-1
Gab1kp82		3.64938764618722e-1
Gab1kp83		3.97312566269352e-2
Gab1kp84		3.20288574330021e-1
Gab1kp85		9.43054858692847e-1
Gab1kp86		1.51265490820168e-1
Gab1kp87		8.35850377271163e-1
Gab1kp88		3.9780775924408e-2
Gab1kp89		6.30932503134102e-1
Gab1kp90		3.32391948687405e-2
Gab1kp91		9.02017698365222e-2
Gab1kp92		5.37650024214315e-1
Gab1kp93		1.52462712529331e-1
Gab1kp94		7.44822529477142e-2
Gab1kp95		3.81255548764085e-2
Gab1kp96		6.72974270318821e-2
Gab1kp97		5.72918654990821e-2
Gab1kp98		5.45264200264478e-1
Gab1kp99		7.84350181988844e-2
Gab1kp100		1.34235843242573e-2
Gab1kp101		3.65112322534793e-1
Gab1kp102		4.42764099548992e-1
Gab1kp103		3.82546347527449e-1
Gab1kp104		6.35883074076855e-1
Gab1kp105		2.59756886335555e-2
Gab1kp106		8.67259547156402e-1
Gab1kp107		4.86156447305251e-1
Gab1kp108		3.77418118675064e-2
Gab1kp109		3.90671913515649e-1
Gab1kp110		3.56725621880395e-2
Gab1kp111		3.82074278974778e-1
Gab1kp112		5.25107067249229e-2
Gab1kp113		5.39435189730705e-1
Gab1kp114		9.34735231509552e-1
Gab1kp115		1.45329501078618e-2
Gab1kp116		8.51311537687985e-2
Gab1kp117		3.54169909715336e-1
Gab1kp118		4.33863429051934e-1
Gab1kp119		3.39300906504241e-1
Gab1kp120		3.85128293076843e-1
Gab1kp121		3.6412969122229e-2
Gab1kp122		3.42139323434344e-1
Gab1kp123		1.25890011675489e-1
Gab1kp124		1.94813470549195e-1
Gab1kp125		9.8003934982591e-2
Gab1kp126		4.92423817966665e-2
Gab1kp127		9.76560811665314e-1
Gab1kp128		3.92729076406397e-2
Gab1kp129		5.53728123038621e-1
Gab1kp130		7.69883583742472e-2
Gab1kp131		7.27273779712493e-2
Gab1kp132		2.73106646626792e-2
Gab1kp133		8.93155681304097e-1
Gab1kp134		4.5877312169635e-2
Gab1kp135		2.02635815571568e-2
Gab1kp136		3.65005005212328e-1
Gab1kp137		3.62453153129877e-2
Gab1kp138		6.56010933793874e-1
Gab1kp139		1.20687516377745e-2
Gab1kp140		9.0068597109921e-1
Gab1kp141		7.94889774029508e-1
Gab1kp142		1.47879843065186e-2
Gab1kp143		3.82145997625815e-1
Gab1kp144		7.39492359635028e-1
Gab1kp145		3.14858173375256e-1
Gab1kp146		9.07136916358093e-1
Gab1kp147		2.13946826210275e-1
Gab1kp148		2.22305968575301e-1
Gab1kp149		8.14790581270913e-1
Gab1kp150		3.52672941805116e-1
Gab1kp151		2.3375040442304e-1
Gab1kp152		3.75503845672807e-2
Gab1kp153		2.22274772107301e-1
Gab1kp154		8.4191964204494e-2
Gab1kp155		8.54033761596293e-2

Gab1kp156		1.46657846706313e-6
Gab1kp157		6.834203762122e-7
Gab1kp158		2.28068860916886e-7
Gab1kp159		2.38343153678147e-6
Gab1kp160		7.4594943271976e-6
Gab1kp161		4.58433700010636e-6
Gab1kp162		9.21239244666731e-1
Gab1kp163		4.83857466703586e-1
Gab1kp164		3.68569830054378e-1
Gab1kp165		3.04402959768407e-2
Gab1kp166		1.4605078022521e-2
Gab1kp167		1.5471681936429e-1
Gab1kp168		9.42829803930488e-2
Gab1kp169		7.09585670734958e-1
Gab1kp170		9.99307828310231e-2
Gab1kp171		4.60012468602565e-7
Gab1kp172		4.62955624807586e-2


PI3Kkp1		5.88410107982645e-7
PI3Kkp2		9.80542618619103e-6
PI3Kkp3		6.66744738668615e-7
PI3Kkp4		3.10295546030824e-1
PI3Kkp5		8.27527892544981e-6
PI3Kkp6		4.44842583219685e-2

PI3Kkp7		1.94595846478239e-6
PI3Kkp8		1.76813220419643e-6
PI3Kkp9		6.386829800952e-6
PI3Kkp10		5.15064097908112e-6
PI3Kkp11		7.85287947262356e-7
PI3Kkp12		4.37909434779328e-6
PI3Kkp13		4.60027889585098e-7

PI3Kkm7		7.0508917514033e-1
PI3Kkm8		6.72236279174069e-1
PI3Kkm9		4.97861425615832e-2
PI3Kkm10		1.41917372721451e-2
PI3Kkm11		5.77649463506566e-1
PI3Kkm12		8.78805058758934e-2
PI3Kkm13		1.7532444673634e-2

PIP3kp1		5.48782230800754e-2
PIP3kp1_5	5*PIP3kp1
PIP3kp3		9.67988792384551e-3


Akt1kp1		9.88533437962741e-7
Akt1kp2		2.21983420789547e-6
Akt1kp3		1.02753819132869e-6
Akt1kp4		5.0119136591926e-1
Akt1kp5		8.34868759634759e-1
Akt1kp6		3.61676724413996e-2
Akt1kp7		3.69211236144935e-7
Akt1kp8		2.62409039589227e-2
Akt1kp9		4.76384581518204e-2
Akt1kp10	2.05533084211891e-2
Akt1kp11	5.36964405281211e-2
Akt1kp12	2.74620431360807e-3


Akt1km1		1.73047023274262e-2
Akt1km2		1.43226416524573e-1


rGAPkp1		7.97543429085357e-7
rGAPkp2		7.27732102199911e-6
rGAPkp3		7.89216962179904e-2
rGAPkp4		4.1163628948874e-1

rGAPkm1		9.63672004244575e-1
end parameters


begin molecule types

# Each species needs a location tag (outside,membrane,cytoplasms,destroyed etc..)
EGF(EGFL,deg~F~T,loc~Ex~En)
HRG(EGFL,deg~F~T,loc~Ex~En)
EGFR(I_III,II,T669~O~P,Y992~O~P,Y1068~O~P,Y1086~O~P,Y1114~O~P,Y1148~O~P,Y1173~O~P,loc~M~En)
ErbB2(II,Y1139~O~P,Y1196~O~P,Y1222~O~P,Y1248~O~P,loc~M~En)
ErbB3(I_III,II,Y1054~O~P,Y1197~O~P,Y1222~O~P,Y1260~O~P,Y1276~O~P,Y1289~O~P,Y1328~O~P,loc~M~En)
ErbB4(I_III,II,Y1056~O~P,Y1188~O~P,Y1242~O~P,loc~M~En)
p52Shc1(PTB,Y317~O~P,loc~C)
Grb2(SH2r,SH2s,cSH3,nSH3,loc~C)
Sos1(PRS,REM,GEF,S1132~O~P,S1167~O~P,S1178~O~P,S1193~O~P,loc~C)
#S581 means S551, Y619 means Y627, Y657 means Y659
Gab1(PH,PRS1_PRS2,T312~O~P,S381~O~P,Y447~O~P,S454~O~P,Y472~O~P,T476~O~P,S581~O~P,S597~O~P,Y619~O~P,Y657~O~P,loc~C)
PI3K(lipid_kinase,R_p85_nSH2_cSH2,G_p85_nSH2_cSH2,p110_RBD,loc~C)
PIP3(C3P,two~F~T,loc~M)
PDK1(PH,STkinase,loc~C)
Akt1(PH,STkinase,T308~O~P,S473~O~P,loc~C)
KRas(GTPase,g~GDP~GTP,loc~M)
p120RasGAP(nSH2,GAP)
Raf1(RBD,STkinase,S29~O~P,S43~O~P,S259~O~P,S289~O~P,S296~O~P,S301~O~P,S338~O~P,Y341~O~P,S471~O~P,T491~O~P,S494~O~P,S642~O~P,loc~C)
MEK1(delta,S218~O~P,S222~O~P,T292~O~P,loc~C)
ERK2(CD,STkinase,T185~O~P,Y187~O~P,loc~C)

end molecule types


begin species

EGF(EGFL,deg~F,loc~Ex)	EGF_tot
HRG(EGFL,deg~F,loc~Ex) HRG_tot
EGFR(I_III,II,T669~O,Y992~O,Y1068~O,Y1086~O,Y1114~O,Y1148~O,Y1173~O,loc~M)	EGFR_tot
ErbB2(II,Y1139~O,Y1196~O,Y1222~O,Y1248~O,loc~M)	ErbB2_tot
ErbB3(I_III,II,Y1054~O,Y1197~O,Y1222~O,Y1260~O,Y1276~O,Y1289~O,Y1328~O,loc~M)	ErbB3_tot
ErbB4(I_III,II,Y1056~O,Y1188~O,Y1242~O,loc~M)    ErbB4_tot
p52Shc1(PTB,Y317~O,loc~C)	p52Shc1_tot
Grb2(SH2r,SH2s,cSH3,nSH3,loc~C)	Grb2_tot
Sos1(PRS,REM,GEF,S1132~O,S1167~O,S1178~O,S1193~O,loc~C)	Sos1_tot
Gab1(PH,PRS1_PRS2,T312~O,S381~O,Y447~O,S454~O,Y472~O,T476~O,S581~O,S597~O,Y619~O,Y657~O,loc~C)	Gab1_tot
PI3K(lipid_kinase,G_p85_nSH2_cSH2,R_p85_nSH2_cSH2,p110_RBD,loc~C)	PI3K_tot
PIP3(C3P,two~F,loc~M) 0
PDK1(PH,STkinase,loc~C)	PDK1_tot
Akt1(PH,STkinase,T308~O,S473~O,loc~C)	Akt1_tot
KRas(GTPase,g~GDP,loc~M)	RAS_tot
p120RasGAP(nSH2,GAP)	p120RasGAP_tot
Raf1(RBD,STkinase,S29~O,S43~O,S259~O,S289~O,S296~O,S301~O,S338~O,Y341~O,S471~O,T491~O,S494~O,S642~O,loc~C)	Raf1_tot
MEK1(delta,S218~O,S222~O,T292~O,loc~C)	MEK1_tot
ERK2(CD,STkinase,T185~O,Y187~O,loc~C)	ERK2_tot

end species

#Species that are being observed

begin observables
Molecules EGFREGFR  EGFR(II!1,loc~M).EGFR(II!1,loc~M)
Molecules EGFRErbB2 EGFR(II!1,loc~M).ErbB2(II!1,loc~M)
Molecules EGFRErbB3 EGFR(II!1,loc~M).ErbB3(II!1,loc~M)
Molecules EGFRErbB4 EGFR(II!1,loc~M).ErbB4(II!1,loc~M)
Molecules ErbB2ErbB2 ErbB2(II!1,loc~M).ErbB2(II!1,loc~M)
Molecules ErbB2ErbB3 ErbB2(II!1,loc~M).ErbB3(II!1,loc~M)
Molecules ErbB2ErbB4 ErbB2(II!1,loc~M).ErbB4(II!1,loc~M)
Molecules ErbB3ErbB3 ErbB3(II!1,loc~M).ErbB3(II!1,loc~M)
Molecules ErbB3ErbB4 ErbB3(II!1,loc~M).ErbB4(II!1,loc~M)
Molecules ErbB4ErbB4 ErbB4(II!1,loc~M).ErbB4(II!1,loc~M)

Molecules EGFREGFREn  EGFR(II!1,loc~En).EGFR(II!1,loc~En)
Molecules EGFRErbB2En EGFR(II!1,loc~En).ErbB2(II!1,loc~En)
Molecules EGFRErbB3En EGFR(II!1,loc~En).ErbB3(II!1,loc~En)
Molecules EGFRErbB4En EGFR(II!1,loc~En).ErbB4(II!1,loc~En)
Molecules ErbB2ErbB2En ErbB2(II!1,loc~En).ErbB2(II!1,loc~En)
Molecules ErbB2ErbB3En ErbB2(II!1,loc~En).ErbB3(II!1,loc~En)
Molecules ErbB2ErbB4En ErbB2(II!1,loc~En).ErbB4(II!1,loc~En)
Molecules ErbB3ErbB3En ErbB3(II!1,loc~En).ErbB3(II!1,loc~En)
Molecules ErbB3ErbB4En ErbB3(II!1,loc~En).ErbB4(II!1,loc~En)
Molecules ErbB4ErbB4En ErbB4(II!1,loc~En).ErbB4(II!1,loc~En)


Molecules ERK_T185 ERK2(T185~P!?)
Molecules AKT_S473 Akt1(S473~P!?)


Molecules EGFR_669 EGFR(T669~P!?)
Molecules EGFR_992 EGFR(Y992~P!?)
Molecules EGFR_1068 EGFR(Y1068~P!?)
Molecules EGFR_1086 EGFR(Y1086~P!?)
Molecules EGFR_1114 EGFR(Y1114~P!?)
Molecules EGFR_1148 EGFR(Y1148~P!?)
Molecules EGFR_1173 EGFR(Y1173~P!?)

Molecules ErbB2_1139 ErbB2(Y1139~P!?)
Molecules ErbB2_1196 ErbB2(Y1196~P!?)
Molecules ErbB2_1222 ErbB2(Y1222~P!?)
Molecules ErbB2_1248 ErbB2(Y1248~P!?)

Molecules ErbB3_1054 ErbB3(Y1054~P!?)
Molecules ErbB3_1197 ErbB3(Y1197~P!?)
Molecules ErbB3_1222 ErbB3(Y1222~P!?)
Molecules ErbB3_1260 ErbB3(Y1260~P!?)
Molecules ErbB3_1276 ErbB3(Y1276~P!?)
Molecules ErbB3_1289 ErbB3(Y1289~P!?)
Molecules ErbB3_1328 ErbB3(Y1328~P!?)

Molecules ErbB4_1056 ErbB4(Y1056~P!?)
Molecules ErbB4_1188 ErbB4(Y1188~P!?)
Molecules ErbB4_1242 ErbB4(Y1242~P!?)

Molecules pSHC p52Shc1(Y317~P!?)

Molecules Sos1_1132 Sos1(S1132~P!?)
Molecules Sos1_1167 Sos1(S1167~P!?)
Molecules Sos1_1178 Sos1(S1178~P!?)
Molecules Sos1_1193 Sos1(S1193~P!?)

Molecules Gab1_312 Gab1(T312~P!?)
Molecules Gab1_381 Gab1(S381~P!?)
Molecules Gab1_447 Gab1(Y447~P!?)
Molecules Gab1_454 Gab1(S454~P!?)
Molecules Gab1_472 Gab1(Y472~P!?)
Molecules Gab1_476 Gab1(T476~P!?)
Molecules Gab1_581 Gab1(S581~P!?)
Molecules Gab1_597 Gab1(S597~P!?)
Molecules Gab1_619 Gab1(Y619~P!?)
Molecules Gab1_657 Gab1(Y657~P!?)


Molecules Raf1_29 Raf1(S29~P!?)
Molecules Raf1_43 Raf1(S43~P!?)
Molecules Raf1_259 Raf1(S259~P!?)
Molecules Raf1_289 Raf1(S289~P!?)
Molecules Raf1_296 Raf1(S296~P!?)
Molecules Raf1_301 Raf1(S301~P!?)
Molecules Raf1_338 Raf1(S338~P!?)
Molecules Raf1_341 Raf1(Y341~P!?)
Molecules Raf1_471 Raf1(S471~P!?)
Molecules Raf1_491 Raf1(T491~P!?)
Molecules Raf1_494 Raf1(S494~P!?)
Molecules Raf1_642 Raf1(S642~P!?)

Molecules MEK1_218 MEK1(S218~P!?)
Molecules MEK1_222 MEK1(S222~P!?)
Molecules MEK1_292 MEK1(T292~P!?)

Molecules pERK_Y187 ERK2(Y187~P!?)

Molecules AKT_T308 Akt1(T308~P!?)
end observables


#######################   RULES BEGIN HERE   #######################
begin reaction rules
############## LIGAND BINDING ##############

# EGF binding to an EGFR monomer
1 EGFR(I_III,II,loc~M) + EGF(EGFL,deg~F,loc~Ex) <-> EGFR(I_III!1,II,loc~M).EGF(EGFL!1,deg~F,loc~Ex)     EGFkp1,EGFkm1

# HRG binding to an ErbB3 monomer
2 ErbB3(I_III,II,loc~M) + HRG(EGFL,deg~F,loc~Ex) <-> ErbB3(I_III!1,II,loc~M).HRG(EGFL!1,deg~F,loc~Ex)     HRG3kp1,HRG3km1

# HRG binding to an ErbB4 monomer
3 ErbB4(I_III,II,loc~M) + HRG(EGFL,deg~F,loc~Ex) <-> ErbB4(I_III!1,II,loc~M).HRG(EGFL!1,deg~F,loc~Ex)     HRG4kp1,HRG4km1


#EGF binding to an EGFR homodimer
4 EGFR(I_III,II!1,loc~M).EGFR(I_III,II!1,loc~M) + EGF(EGFL,deg~F,loc~Ex) <-> EGFR(I_III,II!1,loc~M).EGFR(I_III!2,II!1,loc~M).EGF(EGFL!2,deg~F,loc~Ex)     EGFkp2,EGFkm2
5 EGF(EGFL!2,deg~F,loc~Ex).EGFR(I_III!2,II!1,loc~M).EGFR(I_III,II!1,loc~M) + EGF(EGFL,deg~F,loc~Ex) <-> EGF(EGFL!2,deg~F,loc~Ex).EGFR(I_III!2,II!1,loc~M).EGFR(I_III!3,II!1,loc~M).EGF(EGFL!3,deg~F,loc~Ex)     EGFkp3,EGFkm3

#EGF binding to an EGFR-ErbB2 heterodimer
6 ErbB2(II!1,loc~M).EGFR(I_III,II!1,loc~M) + EGF(EGFL,deg~F,loc~Ex) <-> ErbB2(II!1,loc~M).EGFR(I_III!2,II!1,loc~M).EGF(EGFL!2,deg~F,loc~Ex)     EGFkp2,EGFkm2

#Ligand binding to an EGFR-ErbB3 heterodimer
7 EGF(EGFL,deg~F,loc~Ex) + EGFR(I_III,II!1,loc~M).ErbB3(I_III,II!1,loc~M) <-> EGF(EGFL!2,deg~F,loc~Ex).EGFR(I_III!2,II!1,loc~M).ErbB3(I_III,II!1,loc~M)      EGFkp2,EGFkm2
8 EGFR(I_III,II!1,loc~M).ErbB3(I_III,II!1,loc~M) + HRG(EGFL,deg~F,loc~Ex) <-> EGFR(I_III,II!1,loc~M).ErbB3(I_III!2,II!1,loc~M).HRG(EGFL!2,deg~F,loc~Ex)     HRG3kp2,HRG3km2
9 EGF(EGFL!2,deg~F,loc~Ex).EGFR(I_III!2,II!1,loc~M).ErbB3(I_III,II!1,loc~M) + HRG(EGFL,deg~F,loc~Ex) <-> EGF(EGFL!2,deg~F,loc~Ex).EGFR(I_III!2,II!1,loc~M).ErbB3(I_III!3,II!1,loc~M).HRG(EGFL!3,deg~F,loc~Ex)     HRG3kp3,HRG3km3
10 EGF(EGFL,deg~F,loc~Ex) + EGFR(I_III,II!1,loc~M).ErbB3(I_III!3,II!1,loc~M).HRG(EGFL!3,deg~F,loc~Ex) <-> EGF(EGFL!2,deg~F,loc~Ex).EGFR(I_III!2,II!1,loc~M).ErbB3(I_III!3,II!1,loc~M).HRG(EGFL!3,deg~F,loc~Ex)     EGFkp3,EGFkm3

#Ligand binding to an EGFR-ErbB4 heterodimer
11 EGF(EGFL,deg~F,loc~Ex) + EGFR(I_III,II!1,loc~M).ErbB4(I_III,II!1,loc~M) <-> EGF(EGFL!2,deg~F,loc~Ex).EGFR(I_III!2,II!1,loc~M).ErbB4(I_III,II!1,loc~M)      EGFkp2,EGFkm2
12 EGFR(I_III,II!1,loc~M).ErbB4(I_III,II!1,loc~M) + HRG(EGFL,deg~F,loc~Ex) <-> EGFR(I_III,II!1,loc~M).ErbB4(I_III!2,II!1,loc~M).HRG(EGFL!2,deg~F,loc~Ex)     HRG4kp2,HRG4km2
13 EGF(EGFL!2,deg~F,loc~Ex).EGFR(I_III!2,II!1,loc~M).ErbB4(I_III,II!1,loc~M) + HRG(EGFL,deg~F,loc~Ex) <-> EGF(EGFL!2,deg~F,loc~Ex).EGFR(I_III!2,II!1,loc~M).ErbB4(I_III!3,II!1,loc~M).HRG(EGFL!3,deg~F,loc~Ex)     HRG4kp3,HRG4km3
14 EGF(EGFL,deg~F,loc~Ex) + EGFR(I_III,II!1,loc~M).ErbB4(I_III!3,II!1,loc~M).HRG(EGFL!3,deg~F,loc~Ex) <-> EGF(EGFL!2,deg~F,loc~Ex).EGFR(I_III!2,II!1,loc~M).ErbB4(I_III!3,II!1,loc~M).HRG(EGFL!3,deg~F,loc~Ex)     EGFkp3,EGFkm3


#HRG binding to an ErbB2-ErbB3 heterodimer
15 ErbB2(II!1,loc~M).ErbB3(I_III,II!1,loc~M) + HRG(EGFL,deg~F,loc~Ex) <-> ErbB2(II!1,loc~M).ErbB3(I_III!2,II!1,loc~M).HRG(EGFL!2,deg~F,loc~Ex)     HRG3kp2,HRG3km2

#HRG binding to an ErbB2-ErbB4 heterodimer
16 ErbB2(II!1,loc~M).ErbB4(I_III,II!1,loc~M) + HRG(EGFL,deg~F,loc~Ex) <-> ErbB2(II!1,loc~M).ErbB4(I_III!2,II!1,loc~M).HRG(EGFL!2,deg~F,loc~Ex)     HRG4kp2,HRG4km2


#HRG binding to an ErbB3 homodimer
17 ErbB3(I_III,II!1,loc~M).ErbB3(I_III,II!1,loc~M) + HRG(EGFL,deg~F,loc~Ex) <-> ErbB3(I_III,II!1,loc~M).ErbB3(I_III!2,II!1,loc~M).HRG(EGFL!2,deg~F,loc~Ex)     HRG3kp2,HRG3km2
18 HRG(EGFL!2,deg~F,loc~Ex).ErbB3(I_III!2,II!1,loc~M).ErbB3(I_III,II!1,loc~M) + HRG(EGFL,deg~F,loc~Ex) <-> HRG(EGFL!2,deg~F,loc~Ex).ErbB3(I_III!2,II!1,loc~M).ErbB3(I_III!3,II!1,loc~M).HRG(EGFL!3,deg~F,loc~Ex)     HRG3kp3,HRG3km3

#Ligand binding to an ErbB3-ErbB4 heterodimer
19 ErbB3(I_III,II!1,loc~M).ErbB4(I_III,II!1,loc~M) + HRG(EGFL,deg~F,loc~Ex) <-> ErbB3(I_III,II!1,loc~M).ErbB4(I_III!2,II!1,loc~M).HRG(EGFL!2,deg~F,loc~Ex)     HRG4kp2,HRG4km2
20 HRG(EGFL,deg~F,loc~Ex) + ErbB3(I_III,II!1,loc~M).ErbB4(I_III,II!1,loc~M) <-> HRG(EGFL!2,deg~F,loc~Ex).ErbB3(I_III!2,II!1,loc~M).ErbB4(I_III,II!1,loc~M)     HRG3kp2,HRG3km2
21 HRG(EGFL!2,deg~F,loc~Ex).ErbB3(I_III!2,II!1,loc~M).ErbB4(I_III,II!1,loc~M) + HRG(EGFL,deg~F,loc~Ex) <-> HRG(EGFL!2,deg~F,loc~Ex).ErbB3(I_III!2,II!1,loc~M).ErbB4(I_III!3,II!1,loc~M).HRG(EGFL!3,deg~F,loc~Ex)     HRG4kp3,HRG4km3
22 HRG(EGFL,deg~F,loc~Ex) + ErbB3(I_III,II!1,loc~M).ErbB4(I_III!3,II!1,loc~M).HRG(EGFL!3,deg~F,loc~Ex) <-> HRG(EGFL!2,deg~F,loc~Ex).ErbB3(I_III!2,II!1,loc~M).ErbB4(I_III!3,II!1,loc~M).HRG(EGFL!3,deg~F,loc~Ex)     HRG3kp3,HRG3km3

#HRG binding to an ErbB4 homodimer
23 ErbB4(I_III,II!1,loc~M).ErbB4(I_III,II!1,loc~M) + HRG(EGFL,deg~F,loc~Ex) <-> ErbB4(I_III,II!1,loc~M).ErbB4(I_III!2,II!1,loc~M).HRG(EGFL!2,deg~F,loc~Ex)     HRG4kp2,HRG4km2
24 HRG(EGFL!2,deg~F,loc~Ex).ErbB4(I_III!2,II!1,loc~M).ErbB4(I_III,II!1,loc~M) + HRG(EGFL,deg~F,loc~Ex) <-> HRG(EGFL!2,deg~F,loc~Ex).ErbB4(I_III!2,II!1,loc~M).ErbB4(I_III!3,II!1,loc~M).HRG(EGFL!3,deg~F,loc~Ex)     HRG4kp3,HRG4km3


############## RECEPTOR DIMERIZATION ##############

# EGFR-EGFR
# EGFR-EGFR dimerization with no EGF bound
25 EGFR(I_III,II,loc~M) + EGFR(I_III,II,loc~M) <-> EGFR(I_III,II!1,loc~M).EGFR(I_III,II!1,loc~M)       Dimkp1,Dimkm1

# EGFR-EGFR dimerization with one EGF bound
26 EGFR(I_III!1,II,loc~M).EGF(EGFL!1,loc~Ex) + EGFR(I_III,II,loc~M) <-> EGFR(I_III!1,II!2,loc~M).EGF(EGFL!1,loc~Ex).EGFR(I_III,II!2,loc~M)     Dimkp2,Dimkm2

# EGFR-EGFR dimerization with two EGF bound
27 EGFR(I_III!1,II,loc~M).EGF(EGFL!1,loc~Ex) + EGFR(I_III!2,II,loc~M).EGF(EGFL!2,loc~Ex) <-> EGFR(I_III!1,II!3,loc~M).EGF(EGFL!1,loc~Ex).EGFR(I_III!2,II!3,loc~M).EGF(EGFL!2,loc~Ex)   Dimkp3,Dimkm3


# EGFR-ErbB2
# EGFR-ErbB2 dimerization with no EGF bound
28 EGFR(I_III,II,loc~M) + ErbB2(II,loc~M) <-> EGFR(I_III,II!1,loc~M).ErbB2(II!1,loc~M)       Dimkp4,Dimkm4

# EGFR-ErbB2 dimerization with EGF bound
29 EGFR(I_III!1,II,loc~M).EGF(EGFL!1,loc~Ex) + ErbB2(II,loc~M) <-> EGFR(I_III!1,II!2,loc~M).EGF(EGFL!1,loc~Ex).ErbB2(II!2,loc~M)     Dimkp5,Dimkm5


# EGFR-ErbB3
# EGFR-ErbB3 dimerization with no ligand bound
30 EGFR(I_III,II,loc~M) + ErbB3(I_III,II,loc~M) <-> EGFR(I_III,II!1,loc~M).ErbB3(I_III,II!1,loc~M)       Dimkp6,Dimkm6

# EGFR-ErbB3 dimerization with EGF bound
31 EGFR(I_III!1,II,loc~M).EGF(EGFL!1,loc~Ex) + ErbB3(I_III,II,loc~M) <-> EGFR(I_III!1,II!2,loc~M).EGF(EGFL!1,loc~Ex).ErbB3(I_III,II!2,loc~M)     Dimkp7,Dimkm7

# EGFR-ErbB3 dimerization with HRG bound
32 ErbB3(I_III!1,II,loc~M).HRG(EGFL!1,loc~Ex) + EGFR(I_III,II,loc~M) <-> ErbB3(I_III!1,II!2,loc~M).HRG(EGFL!1,loc~Ex).EGFR(I_III,II!2,loc~M)     Dimkp8,Dimkm8

# EGFR-ErbB3 dimerization with EGF and HRG bound
33 EGFR(I_III!1,II,loc~M).EGF(EGFL!1,loc~Ex) + ErbB3(I_III!2,II,loc~M).HRG(EGFL!2,loc~Ex) <-> EGFR(I_III!1,II!3,loc~M).EGF(EGFL!1,loc~Ex).ErbB3(I_III!2,II!3,loc~M).HRG(EGFL!2,loc~Ex)   Dimkp9,Dimkm9


# EGFR-ErbB4
# EGFR-ErbB4 dimerization with no ligand bound
34 EGFR(I_III,II,loc~M) + ErbB4(I_III,II,loc~M) <-> EGFR(I_III,II!1,loc~M).ErbB4(I_III,II!1,loc~M)       Dimkp10,Dimkm10

# EGFR-ErbB4 dimerization with EGF bound
35 EGFR(I_III!1,II,loc~M).EGF(EGFL!1,loc~Ex) + ErbB4(I_III,II,loc~M) <-> EGFR(I_III!1,II!2,loc~M).EGF(EGFL!1,loc~Ex).ErbB4(I_III,II!2,loc~M)     Dimkp11,Dimkm11

# EGFR-ErbB4 dimerization with HRG bound
36 ErbB4(I_III!1,II,loc~M).HRG(EGFL!1,loc~Ex) + EGFR(I_III,II,loc~M) <-> ErbB4(I_III!1,II!2,loc~M).HRG(EGFL!1,loc~Ex).EGFR(I_III,II!2,loc~M)     Dimkp12,Dimkm12

# EGFR-ErbB4 dimerization with EGF and HRG bound
37 EGFR(I_III!1,II,loc~M).EGF(EGFL!1,loc~Ex) + ErbB4(I_III!2,II,loc~M).HRG(EGFL!2,loc~Ex) <-> EGFR(I_III!1,II!3,loc~M).EGF(EGFL!1,loc~Ex).ErbB4(I_III!2,II!3,loc~M).HRG(EGFL!2,loc~Ex)   Dimkp13,Dimkm13


# ErbB2-ErbB2 dimerization
38 ErbB2(II,loc~M) + ErbB2(II,loc~M) <-> ErbB2(II!1,loc~M).ErbB2(II!1,loc~M)       Dimkp14,Dimkm14


# ErbB2-ErbB3
# ErbB2-ErbB3 dimerization with no ligand bound
39 ErbB2(II,loc~M) + ErbB3(I_III,II,loc~M) <-> ErbB2(II!1,loc~M).ErbB3(I_III,II!1,loc~M)       Dimkp15,Dimkm15

# ErbB2-ErbB3 dimerization with HRG bound
40 ErbB3(I_III!1,II,loc~M).HRG(EGFL!1,loc~Ex) + ErbB2(II,loc~M) <-> ErbB3(I_III!1,II!2,loc~M).HRG(EGFL!1,loc~Ex).ErbB2(II!2,loc~M)     Dimkp16,Dimkm16


# ErbB2-ErbB4
# ErbB2-ErbB4 dimerization with no ligand bound
41 ErbB2(II,loc~M) + ErbB4(I_III,II,loc~M) <-> ErbB2(II!1,loc~M).ErbB4(I_III,II!1,loc~M)       Dimkp17,Dimkm17

# ErbB2-ErbB4 dimerization with HRG bound
42 ErbB4(I_III!1,II,loc~M).HRG(EGFL!1,loc~Ex) + ErbB2(II,loc~M) <-> ErbB4(I_III!1,II!2,loc~M).HRG(EGFL!1,loc~Ex).ErbB2(II!2,loc~M)     Dimkp18,Dimkm18


# ErbB3-ErbB3
# ErbB3-ErbB3 dimerization with no HRG bound
43 ErbB3(I_III,II,loc~M) + ErbB3(I_III,II,loc~M) <-> ErbB3(I_III,II!1,loc~M).ErbB3(I_III,II!1,loc~M)       Dimkp19,Dimkm19

# ErbB3-ErbB3 dimerization with one HRG bound
44 ErbB3(I_III!1,II,loc~M).HRG(EGFL!1,loc~Ex) + ErbB3(I_III,II,loc~M) <-> ErbB3(I_III!1,II!2,loc~M).HRG(EGFL!1,loc~Ex).ErbB3(I_III,II!2,loc~M)     Dimkp20,Dimkm20

# ErbB3-ErbB3 dimerization with two HRG bound
45 ErbB3(I_III!1,II,loc~M).HRG(EGFL!1,loc~Ex) + ErbB3(I_III!2,II,loc~M).HRG(EGFL!2,loc~Ex) <-> ErbB3(I_III!1,II!3,loc~M).HRG(EGFL!1,loc~Ex).ErbB3(I_III!2,II!3,loc~M).HRG(EGFL!2,loc~Ex)   Dimkp21,Dimkm21


# ErbB3-ErbB4
# ErbB3-ErbB4 dimerization with no HRG bound
46 ErbB3(I_III,II,loc~M) + ErbB4(I_III,II,loc~M) <-> ErbB3(I_III,II!1,loc~M).ErbB4(I_III,II!1,loc~M)       Dimkp22,Dimkm22

# ErbB3-ErbB4 dimerization with HRG bound on ErbB3
47 ErbB3(I_III!1,II,loc~M).HRG(EGFL!1,loc~Ex) + ErbB4(I_III,II,loc~M) <-> ErbB3(I_III!1,II!2,loc~M).HRG(EGFL!1,loc~Ex).ErbB4(I_III,II!2,loc~M)     Dimkp23,Dimkm23

# ErbB3-ErbB4 dimerization with HRG bound on ErbB4
48 ErbB3(I_III,II,loc~M) + ErbB4(I_III!1,II,loc~M).HRG(EGFL!1,loc~Ex) <-> ErbB3(I_III,II!2,loc~M).ErbB4(I_III!1,II!2,loc~M).HRG(EGFL!1,loc~Ex)     Dimkp24,Dimkm24

# ErbB3-ErbB4 dimerization with HRG bound on both receptors
49 ErbB3(I_III!1,II,loc~M).HRG(EGFL!1,loc~Ex) + ErbB4(I_III!2,II,loc~M).HRG(EGFL!2,loc~Ex) <-> ErbB3(I_III!1,II!3,loc~M).HRG(EGFL!1,loc~Ex).ErbB4(I_III!2,II!3,loc~M).HRG(EGFL!2,loc~Ex)   Dimkp25,Dimkm25


# ErbB4-ErbB4
# ErbB4-ErbB4 dimerization with no HRG bound
50 ErbB4(I_III,II,loc~M) + ErbB4(I_III,II,loc~M) <-> ErbB4(I_III,II!1,loc~M).ErbB4(I_III,II!1,loc~M)       Dimkp26,Dimkm26

# ErbB4-ErbB4 dimerization with one HRG bound
51 ErbB4(I_III!1,II,loc~M).HRG(EGFL!1,loc~Ex) + ErbB4(I_III,II,loc~M) <-> ErbB4(I_III!1,II!2,loc~M).HRG(EGFL!1,loc~Ex).ErbB4(I_III,II!2,loc~M)     Dimkp27,Dimkm27

# ErbB4-ErbB4 dimerization with two HRG bound
52 ErbB4(I_III!1,II,loc~M).HRG(EGFL!1,loc~Ex) + ErbB4(I_III!2,II,loc~M).HRG(EGFL!2,loc~Ex) <-> ErbB4(I_III!1,II!3,loc~M).HRG(EGFL!1,loc~Ex).ErbB4(I_III!2,II!3,loc~M).HRG(EGFL!2,loc~Ex)   Dimkp28,Dimkm28


############## RECEPTOR INTERNALIZATION ##############

#EGFR-EGFR
#EGFR-EGFR dimers get internalized with two EGF bound
53 EGFR(I_III!1,II!3,loc~M).EGF(EGFL!1,loc~Ex).EGFR(I_III!2,II!3,loc~M).EGF(EGFL!2,loc~Ex) -> EGFR(I_III!1,II!3,loc~En).EGF(EGFL!1,loc~En).EGFR(I_III!2,II!3,loc~En).EGF(EGFL!2,loc~En)	Intkp1

#EGFR-EGFR dimers get internalized with one EGF bound
54 EGFR(I_III!1,II!2,loc~M).EGF(EGFL!1,loc~Ex).EGFR(I_III,II!2,loc~M) -> EGFR(I_III!1,II!2,loc~En).EGF(EGFL!1,loc~En).EGFR(I_III,II!2,loc~En)	Intkp1

#EGFR-EGFR dimers get internalized with no ligand bound
55 EGFR(I_III,II!2,loc~M).EGFR(I_III,II!2,loc~M) -> EGFR(I_III,II!2,loc~En).EGFR(I_III,II!2,loc~En)	Intkp1


#EGFR-ErbB2 dimers get internalized with EGF bound
56 EGFR(I_III!1,II!2,loc~M).EGF(EGFL!1,loc~Ex).ErbB2(II!2,loc~M) -> EGFR(I_III!1,II!2,loc~En).EGF(EGFL!1,loc~En).ErbB2(II!2,loc~En)	Intkp1

#EGFR-ErbB2 dimers get internalized with no ligand bound
57 EGFR(I_III,II!2,loc~M).ErbB2(II!2,loc~M) -> EGFR(I_III,II!2,loc~En).ErbB2(II!2,loc~En)	Intkp1


#EGFR-ErbB3 dimers get internalized with EGF and HRG bound
58 EGFR(I_III!1,II!3,loc~M).EGF(EGFL!1,loc~Ex).ErbB3(I_III!2,II!3,loc~M).HRG(EGFL!2,loc~Ex) -> EGFR(I_III!1,II!3,loc~En).EGF(EGFL!1,loc~En).ErbB3(I_III!2,II!3,loc~En).HRG(EGFL!2,loc~En)	Intkp1

#EGFR-ErbB3 dimers get internalized with HRG bound
59 EGFR(I_III,II!3,loc~M).ErbB3(I_III!2,II!3,loc~M).HRG(EGFL!2,loc~Ex) -> EGFR(I_III,II!3,loc~En).ErbB3(I_III!2,II!3,loc~En).HRG(EGFL!2,loc~En)	Intkp1

#EGFR-ErbB3 dimers get internalized with EGF bound
60 EGFR(I_III!1,II!3,loc~M).EGF(EGFL!1,loc~Ex).ErbB3(I_III,II!3,loc~M) -> EGFR(I_III!1,II!3,loc~En).EGF(EGFL!1,loc~En).ErbB3(I_III,II!3,loc~En)	Intkp1

#EGFR-ErbB3 dimers get internalized with no ligand bound
61 EGFR(I_III,II!3,loc~M).ErbB3(I_III,II!3,loc~M) -> EGFR(I_III,II!3,loc~En).ErbB3(I_III,II!3,loc~En)	Intkp1


#EGFR-ErbB4 dimers get internalized with EGF and HRG bound
62 EGFR(I_III!1,II!3,loc~M).EGF(EGFL!1,loc~Ex).ErbB4(I_III!2,II!3,loc~M).HRG(EGFL!2,loc~Ex) -> EGFR(I_III!1,II!3,loc~En).EGF(EGFL!1,loc~En).ErbB4(I_III!2,II!3,loc~En).HRG(EGFL!2,loc~En)	Intkp1

#EGFR-ErbB4 dimers get internalized with HRG bound
63 EGFR(I_III,II!3,loc~M).ErbB4(I_III!2,II!3,loc~M).HRG(EGFL!2,loc~Ex) -> EGFR(I_III,II!3,loc~En).ErbB4(I_III!2,II!3,loc~En).HRG(EGFL!2,loc~En)	Intkp1

#EGFR-ErbB4 dimers get internalized with EGF bound
64 EGFR(I_III!1,II!3,loc~M).EGF(EGFL!1,loc~Ex).ErbB4(I_III,II!3,loc~M) -> EGFR(I_III!1,II!3,loc~En).EGF(EGFL!1,loc~En).ErbB4(I_III,II!3,loc~En)	Intkp1

#EGFR-ErbB4 dimers get internalized with no ligand bound
65 EGFR(I_III,II!3,loc~M).ErbB4(I_III,II!3,loc~M) -> EGFR(I_III,II!3,loc~En).ErbB4(I_III,II!3,loc~En)	Intkp1


#ErbB2-ErbB2 dimers get internalized
66 ErbB2(II!1,loc~M).ErbB2(II!1,loc~M) -> ErbB2(II!1,loc~En).ErbB2(II!1,loc~En) Intkp1


#ErbB3-ErbB2 dimers get internalized with HRG bound
67 ErbB3(I_III!1,II!2,loc~M).HRG(EGFL!1,loc~Ex).ErbB2(II!2,loc~M) -> ErbB3(I_III!1,II!2,loc~En).HRG(EGFL!1,loc~En).ErbB2(II!2,loc~En)	Intkp1

#ErbB3-ErbB2 dimers get internalized with no ligand bound
68 ErbB3(I_III,II!2,loc~M).ErbB2(II!2,loc~M) -> ErbB3(I_III,II!2,loc~En).ErbB2(II!2,loc~En)	Intkp1


#ErbB4-ErbB2 dimers get internalized with HRG bound
69 ErbB4(I_III!1,II!2,loc~M).HRG(EGFL!1,loc~Ex).ErbB2(II!2,loc~M) -> ErbB4(I_III!1,II!2,loc~En).HRG(EGFL!1,loc~En).ErbB2(II!2,loc~En)	Intkp1

#ErbB4-ErbB2 dimers get internalized with no ligand bound
70 ErbB4(I_III,II!2,loc~M).ErbB2(II!2,loc~M) -> ErbB4(I_III,II!2,loc~En).ErbB2(II!2,loc~En)	Intkp1


#ErbB3-ErbB3 dimers get internalized with two HRG bound
71 ErbB3(I_III!1,II!2,loc~M).HRG(EGFL!1,loc~Ex).ErbB3(I_III!3,II!2,loc~M).HRG(EGFL!3,loc~Ex) -> ErbB3(I_III!1,II!2,loc~En).HRG(EGFL!1,loc~En).ErbB3(I_III!3,II!2,loc~En).HRG(EGFL!3,loc~En)	Intkp1

#ErbB3-ErbB3 dimers get internalized with one HRG bound
72 ErbB3(I_III!1,II!2,loc~M).HRG(EGFL!1,loc~Ex).ErbB3(I_III,II!2,loc~M) -> ErbB3(I_III!1,II!2,loc~En).HRG(EGFL!1,loc~En).ErbB3(I_III,II!2,loc~En)	Intkp1

#ErbB3-ErbB3 dimers get internalized with no HRG bound
73 ErbB3(I_III,II!2,loc~M).ErbB3(I_III,II!2,loc~M) -> ErbB3(I_III,II!2,loc~En).ErbB3(I_III,II!2,loc~En)	Intkp1


#ErbB3-ErbB4 dimers get internalized with two HRG bound
74 ErbB3(I_III!1,II!2,loc~M).HRG(EGFL!1,loc~Ex).ErbB4(I_III!3,II!2,loc~M).HRG(EGFL!3,loc~Ex) -> ErbB3(I_III!1,II!2,loc~En).HRG(EGFL!1,loc~En).ErbB4(I_III!3,II!2,loc~En).HRG(EGFL!3,loc~En)	Intkp1

#ErbB3-ErbB4 dimers get internalized with HRG bound to ErbB3
75 ErbB3(I_III!1,II!2,loc~M).HRG(EGFL!1,loc~Ex).ErbB4(I_III,II!2,loc~M) -> ErbB3(I_III!1,II!2,loc~En).HRG(EGFL!1,loc~En).ErbB4(I_III,II!2,loc~En)	Intkp1

#ErbB3-ErbB4 dimers get internalized with HRG bound to ErbB4
76 ErbB3(I_III,II!2,loc~M).ErbB4(I_III!1,II!2,loc~M).HRG(EGFL!1,loc~Ex) -> ErbB3(I_III,II!2,loc~En).ErbB4(I_III!1,II!2,loc~En).HRG(EGFL!1,loc~En)	Intkp1

#ErbB3-ErbB4 dimers get internalized with no HRG bound to both receptors
77 ErbB3(I_III,II!2,loc~M).ErbB4(I_III,II!2,loc~M) -> ErbB3(I_III,II!2,loc~En).ErbB4(I_III,II!2,loc~En)	Intkp1


#ErbB4-ErbB4 dimers get internalized with two HRG bound
78 ErbB4(I_III!1,II!2,loc~M).HRG(EGFL!1,loc~Ex).ErbB4(I_III!3,II!2,loc~M).HRG(EGFL!3,loc~Ex) -> ErbB4(I_III!1,II!2,loc~En).HRG(EGFL!1,loc~En).ErbB4(I_III!3,II!2,loc~En).HRG(EGFL!3,loc~En)	Intkp1

#ErbB4-ErbB4 dimers get internalized with one HRG bound
79 ErbB4(I_III!1,II!2,loc~M).HRG(EGFL!1,loc~Ex).ErbB4(I_III,II!2,loc~M) -> ErbB4(I_III!1,II!2,loc~En).HRG(EGFL!1,loc~En).ErbB4(I_III,II!2,loc~En)	Intkp1

#ErbB4-ErbB4 dimers get internalized with no HRG bound
80 ErbB4(I_III,II!2,loc~M).ErbB4(I_III,II!2,loc~M) -> ErbB4(I_III,II!2,loc~En).ErbB4(I_III,II!2,loc~En)	Intkp1


#internalized EGFR releases ligand
81 EGFR(I_III!1,loc~En).EGF(EGFL!1,deg~F,loc~En) -> EGFR(I_III,loc~En) + EGF(EGFL,deg~F,loc~En)	iLigkp1

#internalized ErbB3 releases ligand
82 ErbB3(I_III!1,loc~En).HRG(EGFL!1,deg~F,loc~En) -> ErbB3(I_III,loc~En) + HRG(EGFL,deg~F,loc~En)	iLigkp2

#internalized ErbB4 releases ligand
83 ErbB4(I_III!1,loc~En).HRG(EGFL!1,deg~F,loc~En) -> ErbB4(I_III,loc~En) + HRG(EGFL,deg~F,loc~En)	iLigkp3

#internalized ligand gets degraded
84 EGF(EGFL,deg~F,loc~En) -> EGF(EGFL,deg~T,loc~En)	iLigkp4
85 HRG(EGFL,deg~F,loc~En) -> HRG(EGFL,deg~T,loc~En)	iLigkp4


#internalized receptors dissociate
86 EGFR(II!3,loc~En).EGFR(II!3,loc~En) -> EGFR(II,loc~En) + EGFR(II,loc~En)		iDimkp1
87 EGFR(II!2,loc~En).ErbB2(II!2,loc~En) -> EGFR(II,loc~En) + ErbB2(II,loc~En)	iDimkp2
88 EGFR(II!3,loc~En).ErbB3(II!3,loc~En) -> EGFR(II,loc~En) + ErbB3(II,loc~En)	iDimkp3
89 EGFR(II!3,loc~En).ErbB4(II!3,loc~En) -> EGFR(II,loc~En) + ErbB4(II,loc~En)	iDimkp4

90 ErbB2(II!2,loc~En).ErbB2(II!2,loc~En) -> ErbB2(II,loc~En) + ErbB2(II,loc~En)	iDimkp5
91 ErbB2(II!2,loc~En).ErbB3(II!2,loc~En) -> ErbB2(II,loc~En) + ErbB3(II,loc~En)	iDimkp6
92 ErbB2(II!2,loc~En).ErbB4(II!2,loc~En) -> ErbB2(II,loc~En) + ErbB4(II,loc~En)	iDimkp7

93 ErbB3(II!2,loc~En).ErbB3(II!2,loc~En) -> ErbB3(II,loc~En) + ErbB3(II,loc~En)	iDimkp8
94 ErbB3(II!2,loc~En).ErbB4(II!2,loc~En) -> ErbB3(II,loc~En) + ErbB4(II,loc~En)	iDimkp9

95 ErbB4(II!2,loc~En).ErbB4(II!2,loc~En) -> ErbB4(II,loc~En) + ErbB4(II,loc~En)	iDimkp10

#non-dimerized receptors with no ligand bound return to the membrane
96 EGFR(I_III,II,loc~En) -> EGFR(I_III,II,loc~M)	Intkp15
97 ErbB2(II,loc~En) -> ErbB2(II,loc~M)				Intkp15
98 ErbB3(I_III,II,loc~En) -> ErbB3(I_III,II,loc~M)	Intkp15
99 ErbB4(I_III,II,loc~En) -> ErbB4(I_III,II,loc~M)	Intkp15

############## RECEPTOR CROSS-PHOSPHORYLATION ##############

# Receptor Phosphorylation Reactions

# NOTE: The philosophy of these rules is that both ligand binding sites in a dimer must be occupied in order for kinase domains to be active

## EGFR
## (EGFR-EGFR dimers)(992,1068,1086,1114,1148,1173)
## (ErbB2-EGFR dimers)(992,1068,1086,1114,1148,1173)
## (ErbB4-EGFR dimers)(992,1068,1086,1114,1148,1173)

## ErbB2
## (EGFR-ErbB2 dimers)(1139,1196,1222,1248)
## (ErbB4-ErbB2 dimers)(1139,1196,1222,1248)

## ErbB3
## (EGFR-ErbB3 dimers)(1054,1197,1222,Y1260,1276,1289,1328)
## (ErbB2-ErbB3 dimers)(1054,1197,1222,Y1260,1276,1289,1328)
## (ErbB4-ErbB3 dimers)(1054,1197,1222,Y1260,1276,1289,1328)

## ErbB4
## (EGFR-ErbB4 dimers)(1056,1188,1242)
## (ErbB2-ErbB4 dimers)(1056,1188,1242)
## (ErbB4-ErbB4 dimers)(1056,1188,1242)

## -----------------------------------------

# cross-phosphorylation of EGFR
# (EGFR-EGFR dimers) Cross-Phosphorylation of EGFR by EGFR at
# (992,1068,1086,1114,1148,1173)
100 EGF(EGFL!2).EGFR(I_III!2,II!1,T669~O).EGFR(I_III!3,II!1,Y992~O).EGF(EGFL!3) ->  EGF(EGFL!2).EGFR(I_III!2,II!1,T669~O).EGFR(I_III!3,II!1,Y992~P).EGF(EGFL!3) Phosphokp1
101 EGF(EGFL!2).EGFR(I_III!2,II!1,T669~O).EGFR(I_III!3,II!1,Y1068~O).EGF(EGFL!3) ->  EGF(EGFL!2).EGFR(I_III!2,II!1,T669~O).EGFR(I_III!3,II!1,Y1068~P).EGF(EGFL!3) Phosphokp2
102 EGF(EGFL!2).EGFR(I_III!2,II!1,T669~O).EGFR(I_III!3,II!1,Y1086~O).EGF(EGFL!3) ->  EGF(EGFL!2).EGFR(I_III!2,II!1,T669~O).EGFR(I_III!3,II!1,Y1086~P).EGF(EGFL!3) Phosphokp3
103 EGF(EGFL!2).EGFR(I_III!2,II!1,T669~O).EGFR(I_III!3,II!1,Y1114~O).EGF(EGFL!3) ->  EGF(EGFL!2).EGFR(I_III!2,II!1,T669~O).EGFR(I_III!3,II!1,Y1114~P).EGF(EGFL!3) Phosphokp4
104 EGF(EGFL!2).EGFR(I_III!2,II!1,T669~O).EGFR(I_III!3,II!1,Y1148~O).EGF(EGFL!3) ->  EGF(EGFL!2).EGFR(I_III!2,II!1,T669~O).EGFR(I_III!3,II!1,Y1148~P).EGF(EGFL!3) Phosphokp5
105 EGF(EGFL!2).EGFR(I_III!2,II!1,T669~O).EGFR(I_III!3,II!1,Y1173~O).EGF(EGFL!3) ->  EGF(EGFL!2).EGFR(I_III!2,II!1,T669~O).EGFR(I_III!3,II!1,Y1173~P).EGF(EGFL!3) Phosphokp6


# (ErbB2-EGFR dimers) Cross-Phosphorylation of EGFR by ErbB2 at
# (992,1068,1086,1114,1148,1173)
106 ErbB2(II!1).EGFR(I_III!3,II!1,Y992~O).EGF(EGFL!3) ->  ErbB2(II!1).EGFR(I_III!3,II!1,Y992~P).EGF(EGFL!3) Phosphokp7
107 ErbB2(II!1).EGFR(I_III!3,II!1,Y1068~O).EGF(EGFL!3) ->  ErbB2(II!1).EGFR(I_III!3,II!1,Y1068~P).EGF(EGFL!3) Phosphokp8
108 ErbB2(II!1).EGFR(I_III!3,II!1,Y1086~O).EGF(EGFL!3) ->  ErbB2(II!1).EGFR(I_III!3,II!1,Y1086~P).EGF(EGFL!3) Phosphokp9
109 ErbB2(II!1).EGFR(I_III!3,II!1,Y1114~O).EGF(EGFL!3) ->  ErbB2(II!1).EGFR(I_III!3,II!1,Y1114~P).EGF(EGFL!3) Phosphokp10
110 ErbB2(II!1).EGFR(I_III!3,II!1,Y1148~O).EGF(EGFL!3) ->  ErbB2(II!1).EGFR(I_III!3,II!1,Y1148~P).EGF(EGFL!3) Phosphokp11
111 ErbB2(II!1).EGFR(I_III!3,II!1,Y1173~O).EGF(EGFL!3) ->  ErbB2(II!1).EGFR(I_III!3,II!1,Y1173~P).EGF(EGFL!3) Phosphokp12

# (ErbB4-EGFR dimers) Cross-Phosphorylation of EGFR by ErbB4 at
# (992,1068,1086,1114,1148,1173)
112 HRG(EGFL!2).ErbB4(I_III!2,II!1).EGFR(I_III!3,II!1,Y992~O).EGF(EGFL!3) ->  HRG(EGFL!2).ErbB4(I_III!2,II!1).EGFR(I_III!3,II!1,Y992~P).EGF(EGFL!3) Phosphokp13
113 HRG(EGFL!2).ErbB4(I_III!2,II!1).EGFR(I_III!3,II!1,Y1068~O).EGF(EGFL!3) ->  HRG(EGFL!2).ErbB4(I_III!2,II!1).EGFR(I_III!3,II!1,Y1068~P).EGF(EGFL!3) Phosphokp14
114 HRG(EGFL!2).ErbB4(I_III!2,II!1).EGFR(I_III!3,II!1,Y1086~O).EGF(EGFL!3) ->  HRG(EGFL!2).ErbB4(I_III!2,II!1).EGFR(I_III!3,II!1,Y1086~P).EGF(EGFL!3) Phosphokp15
115 HRG(EGFL!2).ErbB4(I_III!2,II!1).EGFR(I_III!3,II!1,Y1114~O).EGF(EGFL!3) ->  HRG(EGFL!2).ErbB4(I_III!2,II!1).EGFR(I_III!3,II!1,Y1114~P).EGF(EGFL!3) Phosphokp16
116 HRG(EGFL!2).ErbB4(I_III!2,II!1).EGFR(I_III!3,II!1,Y1148~O).EGF(EGFL!3) ->  HRG(EGFL!2).ErbB4(I_III!2,II!1).EGFR(I_III!3,II!1,Y1148~P).EGF(EGFL!3) Phosphokp17
117 HRG(EGFL!2).ErbB4(I_III!2,II!1).EGFR(I_III!3,II!1,Y1173~O).EGF(EGFL!3) ->  HRG(EGFL!2).ErbB4(I_III!2,II!1).EGFR(I_III!3,II!1,Y1173~P).EGF(EGFL!3) Phosphokp18


# Cross-phosphorylation of ErbB2
# (EGFR-ErbB2 dimers) Cross-Phosphorylation of ErbB2 by EGFR at
# (1139,1196,1222,1248)
118 EGF(EGFL!2).EGFR(I_III!2,II!1,T669~O).ErbB2(II!1,Y1139~O) ->  EGF(EGFL!2).EGFR(I_III!2,II!1,T669~O).ErbB2(II!1,Y1139~P) Phosphokp19
119 EGF(EGFL!2).EGFR(I_III!2,II!1,T669~O).ErbB2(II!1,Y1196~O) ->  EGF(EGFL!2).EGFR(I_III!2,II!1,T669~O).ErbB2(II!1,Y1196~P) Phosphokp20
120 EGF(EGFL!2).EGFR(I_III!2,II!1,T669~O).ErbB2(II!1,Y1222~O) ->  EGF(EGFL!2).EGFR(I_III!2,II!1,T669~O).ErbB2(II!1,Y1222~P) Phosphokp21
121 EGF(EGFL!2).EGFR(I_III!2,II!1,T669~O).ErbB2(II!1,Y1248~O) ->  EGF(EGFL!2).EGFR(I_III!2,II!1,T669~O).ErbB2(II!1,Y1248~P) Phosphokp22


# Cross-phosphorylation of ErbB2
# (ErbB4-ErbB2 dimers) Cross-Phosphorylation of ErbB2 by ErbB4 at
# (1139,1196,1222,1248)
122 HRG(EGFL!2).ErbB4(I_III!2,II!1).ErbB2(II!1,Y1139~O) ->  HRG(EGFL!2).ErbB4(I_III!2,II!1).ErbB2(II!1,Y1139~P) Phosphokp23
123 HRG(EGFL!2).ErbB4(I_III!2,II!1).ErbB2(II!1,Y1196~O) ->  HRG(EGFL!2).ErbB4(I_III!2,II!1).ErbB2(II!1,Y1196~P) Phosphokp24
124 HRG(EGFL!2).ErbB4(I_III!2,II!1).ErbB2(II!1,Y1222~O) ->  HRG(EGFL!2).ErbB4(I_III!2,II!1).ErbB2(II!1,Y1222~P) Phosphokp25
125 HRG(EGFL!2).ErbB4(I_III!2,II!1).ErbB2(II!1,Y1248~O) ->  HRG(EGFL!2).ErbB4(I_III!2,II!1).ErbB2(II!1,Y1248~P) Phosphokp26


# cross-phosphorylation of ErbB3
# (EGFR-ErbB3 dimers) cross-phosphorylation of ErbB3 by EGFR at
# (1054,1197,1222,Y1260,1276,1289,1328)
126 EGF(EGFL!2).EGFR(I_III!2,II!1,T669~O).ErbB3(I_III!3,II!1,Y1054~O).HRG(EGFL!3) ->  EGF(EGFL!2).EGFR(I_III!2,II!1,T669~O).ErbB3(I_III!3,II!1,Y1054~P).HRG(EGFL!3) Phosphokp27
127 EGF(EGFL!2).EGFR(I_III!2,II!1,T669~O).ErbB3(I_III!3,II!1,Y1197~O).HRG(EGFL!3) ->  EGF(EGFL!2).EGFR(I_III!2,II!1,T669~O).ErbB3(I_III!3,II!1,Y1197~P).HRG(EGFL!3) Phosphokp28
128 EGF(EGFL!2).EGFR(I_III!2,II!1,T669~O).ErbB3(I_III!3,II!1,Y1222~O).HRG(EGFL!3) ->  EGF(EGFL!2).EGFR(I_III!2,II!1,T669~O).ErbB3(I_III!3,II!1,Y1222~P).HRG(EGFL!3) Phosphokp29
129 EGF(EGFL!2).EGFR(I_III!2,II!1,T669~O).ErbB3(I_III!3,II!1,Y1260~O).HRG(EGFL!3) ->  EGF(EGFL!2).EGFR(I_III!2,II!1,T669~O).ErbB3(I_III!3,II!1,Y1260~P).HRG(EGFL!3) Phosphokp30
130 EGF(EGFL!2).EGFR(I_III!2,II!1,T669~O).ErbB3(I_III!3,II!1,Y1276~O).HRG(EGFL!3) ->  EGF(EGFL!2).EGFR(I_III!2,II!1,T669~O).ErbB3(I_III!3,II!1,Y1276~P).HRG(EGFL!3) Phosphokp31
131 EGF(EGFL!2).EGFR(I_III!2,II!1,T669~O).ErbB3(I_III!3,II!1,Y1289~O).HRG(EGFL!3) ->  EGF(EGFL!2).EGFR(I_III!2,II!1,T669~O).ErbB3(I_III!3,II!1,Y1289~P).HRG(EGFL!3) Phosphokp32
132 EGF(EGFL!2).EGFR(I_III!2,II!1,T669~O).ErbB3(I_III!3,II!1,Y1328~O).HRG(EGFL!3) ->  EGF(EGFL!2).EGFR(I_III!2,II!1,T669~O).ErbB3(I_III!3,II!1,Y1328~P).HRG(EGFL!3) Phosphokp33


# (ErbB2-ErbB3 dimers) cross-phosphorylation of Erbb3 by ErbB2 at
# (1054,1197,1222,Y1260,1276,1289,1328)
133 ErbB2(II!1).ErbB3(I_III!3,II!1,Y1054~O).HRG(EGFL!3) ->  ErbB2(II!1).ErbB3(I_III!3,II!1,Y1054~P).HRG(EGFL!3) Phosphokp34
134 ErbB2(II!1).ErbB3(I_III!3,II!1,Y1197~O).HRG(EGFL!3) ->  ErbB2(II!1).ErbB3(I_III!3,II!1,Y1197~P).HRG(EGFL!3) Phosphokp35
135 ErbB2(II!1).ErbB3(I_III!3,II!1,Y1222~O).HRG(EGFL!3) ->  ErbB2(II!1).ErbB3(I_III!3,II!1,Y1222~P).HRG(EGFL!3) Phosphokp36
136 ErbB2(II!1).ErbB3(I_III!3,II!1,Y1260~O).HRG(EGFL!3) ->  ErbB2(II!1).ErbB3(I_III!3,II!1,Y1260~P).HRG(EGFL!3) Phosphokp37
137 ErbB2(II!1).ErbB3(I_III!3,II!1,Y1276~O).HRG(EGFL!3) ->  ErbB2(II!1).ErbB3(I_III!3,II!1,Y1276~P).HRG(EGFL!3) Phosphokp38
138 ErbB2(II!1).ErbB3(I_III!3,II!1,Y1289~O).HRG(EGFL!3) ->  ErbB2(II!1).ErbB3(I_III!3,II!1,Y1289~P).HRG(EGFL!3) Phosphokp39
139 ErbB2(II!1).ErbB3(I_III!3,II!1,Y1328~O).HRG(EGFL!3) ->  ErbB2(II!1).ErbB3(I_III!3,II!1,Y1328~P).HRG(EGFL!3) Phosphokp40


# (ErbB4-ErbB3 dimers) cross-phosphorylation of Erbb3 by ErbB4 at
# (1054,1197,1222,Y1260,1276,1289,1328)
140 HRG(EGFL!2).ErbB4(I_III!2,II!1).ErbB3(I_III!3,II!1,Y1054~O).HRG(EGFL!3) ->  HRG(EGFL!2).ErbB4(I_III!2,II!1).ErbB3(I_III!3,II!1,Y1054~P).HRG(EGFL!3) Phosphokp41
141 HRG(EGFL!2).ErbB4(I_III!2,II!1).ErbB3(I_III!3,II!1,Y1197~O).HRG(EGFL!3) ->  HRG(EGFL!2).ErbB4(I_III!2,II!1).ErbB3(I_III!3,II!1,Y1197~P).HRG(EGFL!3) Phosphokp42
142 HRG(EGFL!2).ErbB4(I_III!2,II!1).ErbB3(I_III!3,II!1,Y1222~O).HRG(EGFL!3) ->  HRG(EGFL!2).ErbB4(I_III!2,II!1).ErbB3(I_III!3,II!1,Y1222~P).HRG(EGFL!3) Phosphokp43
143 HRG(EGFL!2).ErbB4(I_III!2,II!1).ErbB3(I_III!3,II!1,Y1260~O).HRG(EGFL!3) ->  HRG(EGFL!2).ErbB4(I_III!2,II!1).ErbB3(I_III!3,II!1,Y1260~P).HRG(EGFL!3) Phosphokp44
144 HRG(EGFL!2).ErbB4(I_III!2,II!1).ErbB3(I_III!3,II!1,Y1276~O).HRG(EGFL!3) ->  HRG(EGFL!2).ErbB4(I_III!2,II!1).ErbB3(I_III!3,II!1,Y1276~P).HRG(EGFL!3) Phosphokp45
145 HRG(EGFL!2).ErbB4(I_III!2,II!1).ErbB3(I_III!3,II!1,Y1289~O).HRG(EGFL!3) ->  HRG(EGFL!2).ErbB4(I_III!2,II!1).ErbB3(I_III!3,II!1,Y1289~P).HRG(EGFL!3) Phosphokp46
146 HRG(EGFL!2).ErbB4(I_III!2,II!1).ErbB3(I_III!3,II!1,Y1328~O).HRG(EGFL!3) ->  HRG(EGFL!2).ErbB4(I_III!2,II!1).ErbB3(I_III!3,II!1,Y1328~P).HRG(EGFL!3) Phosphokp47


# cross-phosphorylation of ErbB4
# (EGFR-ErbB4 dimers) cross-phosphorylation of ErbB4 by EGFR at
# (1056,1188,1242)
147 EGF(EGFL!2).EGFR(I_III!2,II!1,T669~O).ErbB4(I_III!3,II!1,Y1056~O).HRG(EGFL!3) ->  EGF(EGFL!2).EGFR(I_III!2,II!1,T669~O).ErbB4(I_III!3,II!1,Y1056~P).HRG(EGFL!3) Phosphokp48
148 EGF(EGFL!2).EGFR(I_III!2,II!1,T669~O).ErbB4(I_III!3,II!1,Y1188~O).HRG(EGFL!3) ->  EGF(EGFL!2).EGFR(I_III!2,II!1,T669~O).ErbB4(I_III!3,II!1,Y1188~P).HRG(EGFL!3) Phosphokp49
149 EGF(EGFL!2).EGFR(I_III!2,II!1,T669~O).ErbB4(I_III!3,II!1,Y1242~O).HRG(EGFL!3) ->  EGF(EGFL!2).EGFR(I_III!2,II!1,T669~O).ErbB4(I_III!3,II!1,Y1242~P).HRG(EGFL!3) Phosphokp50


# (ErbB2-ErbB4 dimers) cross-phosphorylation of ErbB4 by ErbB2 at
# (1056,1188,1242)
150 ErbB2(II!1).ErbB4(I_III!3,II!1,Y1056~O).HRG(EGFL!3) ->  ErbB2(II!1).ErbB4(I_III!3,II!1,Y1056~P).HRG(EGFL!3) Phosphokp51
151 ErbB2(II!1).ErbB4(I_III!3,II!1,Y1188~O).HRG(EGFL!3) ->  ErbB2(II!1).ErbB4(I_III!3,II!1,Y1188~P).HRG(EGFL!3) Phosphokp52
152 ErbB2(II!1).ErbB4(I_III!3,II!1,Y1242~O).HRG(EGFL!3) ->  ErbB2(II!1).ErbB4(I_III!3,II!1,Y1242~P).HRG(EGFL!3) Phosphokp53


# (ErbB4-ErbB4 dimers) cross-phosphorylation of ErbB4 by ErbB4 at
# (1056,1188,1242)
153 HRG(EGFL!2).ErbB4(I_III!2,II!1).ErbB4(I_III!3,II!1,Y1056~O).HRG(EGFL!3) ->  HRG(EGFL!2).ErbB4(I_III!2,II!1).ErbB4(I_III!3,II!1,Y1056~P).HRG(EGFL!3) Phosphokp54
154 HRG(EGFL!2).ErbB4(I_III!2,II!1).ErbB4(I_III!3,II!1,Y1188~O).HRG(EGFL!3) ->  HRG(EGFL!2).ErbB4(I_III!2,II!1).ErbB4(I_III!3,II!1,Y1188~P).HRG(EGFL!3) Phosphokp55
155 HRG(EGFL!2).ErbB4(I_III!2,II!1).ErbB4(I_III!3,II!1,Y1242~O).HRG(EGFL!3) ->  HRG(EGFL!2).ErbB4(I_III!2,II!1).ErbB4(I_III!3,II!1,Y1242~P).HRG(EGFL!3) Phosphokp56

############## INTRINSIC RECEPTOR DEPHOSPHORYLATION ##############

# EGFR (992,1068,1086,1114,1148,1173)
156 EGFR(Y992~P) -> EGFR(Y992~O) Phosphokm1
157 EGFR(Y1068~P) -> EGFR(Y1068~O) Phosphokm2
158 EGFR(Y1086~P) -> EGFR(Y1086~O) Phosphokm3
159 EGFR(Y1114~P) -> EGFR(Y1114~O) Phosphokm4
160 EGFR(Y1148~P) -> EGFR(Y1148~O) Phosphokm5
161 EGFR(Y1173~P) -> EGFR(Y1173~O) Phosphokm6


# ErbB2 (1139,1196,1222,1248)
162 ErbB2(Y1139~P) -> ErbB2(Y1139~O) Phosphokm7
163 ErbB2(Y1196~P) -> ErbB2(Y1196~O) Phosphokm8
164 ErbB2(Y1222~P) -> ErbB2(Y1222~O) Phosphokm9
165 ErbB2(Y1248~P) -> ErbB2(Y1248~O) Phosphokm10


# ErbB3 (1054,1197,1222,Y1260,1276,1289,1328)
166 ErbB3(Y1054~P) -> ErbB3(Y1054~O) Phosphokm11
167 ErbB3(Y1197~P) -> ErbB3(Y1197~O) Phosphokm12
168 ErbB3(Y1222~P) -> ErbB3(Y1222~O) Phosphokm13
169 ErbB3(Y1260~P) -> ErbB3(Y1260~O) Phosphokm14
170 ErbB3(Y1276~P) -> ErbB3(Y1276~O) Phosphokm15
171 ErbB3(Y1289~P) -> ErbB3(Y1289~O) Phosphokm16
172 ErbB3(Y1328~P) -> ErbB3(Y1328~O) Phosphokm17


# ErbB3 (1056,1188,1242)
173 ErbB4(Y1056~P) -> ErbB4(Y1056~O) Phosphokm18
174 ErbB4(Y1188~P) -> ErbB4(Y1188~O) Phosphokm19
175 ErbB4(Y1242~P) -> ErbB4(Y1242~O) Phosphokm20

############## Grb2 BINDING TO A RECEPTOR ##############

## EGFR = 1068,1114,1148,1173
## ErbB2 = 1139

# EGFR
# Y1068,Y1086,Y1148,Y1173
176 EGFR(Y1068~P) + Grb2(SH2r,SH2s) <-> EGFR(Y1068~P!1).Grb2(SH2r!1,SH2s) Grb2kp1,Grb2km1
177 EGFR(Y1114~P) + Grb2(SH2r,SH2s) <-> EGFR(Y1114~P!1).Grb2(SH2r!1,SH2s) Grb2kp2,Grb2km2
178 EGFR(Y1148~P) + Grb2(SH2r,SH2s) <-> EGFR(Y1148~P!1).Grb2(SH2r!1,SH2s) Grb2kp3,Grb2km3
179 EGFR(Y1173~P) + Grb2(SH2r,SH2s) <-> EGFR(Y1173~P!1).Grb2(SH2r!1,SH2s) Grb2kp4,Grb2km4

# ErbB2
# Y1139
180 ErbB2(Y1139~P) + Grb2(SH2r,SH2s) <-> ErbB2(Y1139~P!1).Grb2(SH2r!1,SH2s)         Grb2kp5,Grb2km5


############## Shc BINDING TO A RECEPTOR ##############

## EGFR - 992,1086,1114
## ErbB2 - 1196,1222,1248
## ErbB3 - 1328
## ErbB4 - 1188,1242

# EGFR
#Y992,Y086,Y1114
181 EGFR(Y992~P) + p52Shc1(PTB) <-> EGFR(Y992~P!1).p52Shc1(PTB!1)       Shc1kp0,Shc1km0
182 EGFR(Y1086~P) + p52Shc1(PTB) <-> EGFR(Y1086~P!1).p52Shc1(PTB!1)       Shc1kp1,Shc1km1
183 EGFR(Y1114~P) + p52Shc1(PTB) <-> EGFR(Y1114~P!1).p52Shc1(PTB!1)       Shc1kp2,Shc1km2

# ErbB2
#Y1196,Y1222,Y1248
184 ErbB2(Y1196~P) + p52Shc1(PTB) <-> ErbB2(Y1196~P!1).p52Shc1(PTB!1)       Shc1kp3,Shc1km3
185 ErbB2(Y1222~P) + p52Shc1(PTB) <-> ErbB2(Y1222~P!1).p52Shc1(PTB!1)       Shc1kp4,Shc1km4
186 ErbB2(Y1248~P) + p52Shc1(PTB) <-> ErbB2(Y1248~P!1).p52Shc1(PTB!1)       Shc1kp5,Shc1km5

# ErbB3
#Y1328
187 ErbB3(Y1328~P) + p52Shc1(PTB) <-> ErbB3(Y1328~P!1).p52Shc1(PTB!1)       Shc1kp6,Shc1km6

#ErbB4
#Y1188,Y1242
188 ErbB4(Y1188~P) + p52Shc1(PTB) <-> ErbB4(Y1188~P!1).p52Shc1(PTB!1)       Shc1kp7,Shc1km7
189 ErbB4(Y1242~P) + p52Shc1(PTB) <-> ErbB4(Y1242~P!1).p52Shc1(PTB!1)       Shc1kp8,Shc1km8

# Transphosphorylation of Shc by a receptor
# EGFR-EGFR where EGFR transphosphorylates Shc
#Y992,Y086,Y1114
190 EGF(EGFL!2).EGFR(I_III!2,II!1,T669~O).p52Shc1(Y317~O,PTB!4).EGFR(I_III!3,II!1,Y992~P!4).EGF(EGFL!3) -> EGF(EGFL!2).EGFR(I_III!2,II!1,T669~O).p52Shc1(Y317~P,PTB!4).EGFR(I_III!3,II!1,Y992~P!4).EGF(EGFL!3)    Shc1kp9
191 EGF(EGFL!2).EGFR(I_III!2,II!1,T669~O).p52Shc1(Y317~O,PTB!4).EGFR(I_III!3,II!1,Y1086~P!4).EGF(EGFL!3) -> EGF(EGFL!2).EGFR(I_III!2,II!1,T669~O).p52Shc1(Y317~P,PTB!4).EGFR(I_III!3,II!1,Y1086~P!4).EGF(EGFL!3)    Shc1kp10
192 EGF(EGFL!2).EGFR(I_III!2,II!1,T669~O).p52Shc1(Y317~O,PTB!4).EGFR(I_III!3,II!1,Y1114~P!4).EGF(EGFL!3) -> EGF(EGFL!2).EGFR(I_III!2,II!1,T669~O).p52Shc1(Y317~P,PTB!4).EGFR(I_III!3,II!1,Y1114~P!4).EGF(EGFL!3)    Shc1kp11

# EGFR-ErbB2 where EGFR transphosphorylates Shc
#Y1196,Y1222,Y1248
193 EGF(EGFL!2).EGFR(I_III!2,II!1,T669~O).p52Shc1(Y317~O,PTB!4).ErbB2(II!1,Y1196~P!4) -> EGF(EGFL!2).EGFR(I_III!2,II!1,T669~O).p52Shc1(Y317~P,PTB!4).ErbB2(II!1,Y1196~P!4)    Shc1kp12
194 EGF(EGFL!2).EGFR(I_III!2,II!1,T669~O).p52Shc1(Y317~O,PTB!4).ErbB2(II!1,Y1222~P!4) -> EGF(EGFL!2).EGFR(I_III!2,II!1,T669~O).p52Shc1(Y317~P,PTB!4).ErbB2(II!1,Y1222~P!4)    Shc1kp13
195 EGF(EGFL!2).EGFR(I_III!2,II!1,T669~O).p52Shc1(Y317~O,PTB!4).ErbB2(II!1,Y1248~P!4) -> EGF(EGFL!2).EGFR(I_III!2,II!1,T669~O).p52Shc1(Y317~P,PTB!4).ErbB2(II!1,Y1248~P!4)    Shc1kp14

# EGFR-ErbB3 where EGFR transphosphorylates Shc
#Y1328
196 EGF(EGFL!2).EGFR(I_III!2,II!1,T669~O).p52Shc1(Y317~O,PTB!4).ErbB3(I_III!3,II!1,Y1328~P!4).HRG(EGFL!3) -> EGF(EGFL!2).EGFR(I_III!2,II!1,T669~O).p52Shc1(Y317~P,PTB!4).ErbB3(I_III!3,II!1,Y1328~P!4).HRG(EGFL!3)    Shc1kp15

# EGFR-ErbB4 where EGFR transphosphorylates Shc
#Y1188,Y1242
197 EGF(EGFL!2).EGFR(I_III!2,II!1,T669~O).p52Shc1(Y317~O,PTB!4).ErbB4(I_III!3,II!1,Y1188~P!4).HRG(EGFL!3) -> EGF(EGFL!2).EGFR(I_III!2,II!1,T669~O).p52Shc1(Y317~P,PTB!4).ErbB4(I_III!3,II!1,Y1188~P!4).HRG(EGFL!3)    Shc1kp16
198 EGF(EGFL!2).EGFR(I_III!2,II!1,T669~O).p52Shc1(Y317~O,PTB!4).ErbB4(I_III!3,II!1,Y1242~P!4).HRG(EGFL!3) -> EGF(EGFL!2).EGFR(I_III!2,II!1,T669~O).p52Shc1(Y317~P,PTB!4).ErbB4(I_III!3,II!1,Y1242~P!4).HRG(EGFL!3)    Shc1kp17


# ErbB2-EGFR where ErbB2 transphosphorylates Shc
#Y992,Y086,Y1114
199 ErbB2(II!1).p52Shc1(Y317~O,PTB!4).EGFR(I_III!2,II!1,Y992~P!4).EGF(EGFL!2) -> ErbB2(II!1).p52Shc1(Y317~P,PTB!4).EGFR(I_III!2,II!1,Y992~P!4).EGF(EGFL!2) Shc1kp18
200 ErbB2(II!1).p52Shc1(Y317~O,PTB!4).EGFR(I_III!2,II!1,Y1086~P!4).EGF(EGFL!2) -> ErbB2(II!1).p52Shc1(Y317~P,PTB!4).EGFR(I_III!2,II!1,Y1086~P!4).EGF(EGFL!2) Shc1kp19
201 ErbB2(II!1).p52Shc1(Y317~O,PTB!4).EGFR(I_III!2,II!1,Y1114~P!4).EGF(EGFL!2) -> ErbB2(II!1).p52Shc1(Y317~P,PTB!4).EGFR(I_III!2,II!1,Y1114~P!4).EGF(EGFL!2) Shc1kp20

# ErbB2-ErbB3 where ErbB2 transphosphorylates Shc
#Y1328
202 ErbB2(II!1).p52Shc1(Y317~O,PTB!4).ErbB3(I_III!2,II!1,Y1328~P!4).HRG(EGFL!2) -> ErbB2(II!1).p52Shc1(Y317~P,PTB!4).ErbB3(I_III!2,II!1,Y1328~P!4).HRG(EGFL!2) Shc1kp21

# ErbB2-ErbB4 where ErbB2 transphosphorylates Shc
#Y1188,Y1242
203 ErbB2(II!1).p52Shc1(Y317~O,PTB!4).ErbB4(I_III!3,II!1,Y1188~P!4).HRG(EGFL!3) -> ErbB2(II!1).p52Shc1(Y317~P,PTB!4).ErbB4(I_III!3,II!1,Y1188~P!4).HRG(EGFL!3)    Shc1kp22
204 ErbB2(II!1).p52Shc1(Y317~O,PTB!4).ErbB4(I_III!3,II!1,Y1242~P!4).HRG(EGFL!3) -> ErbB2(II!1).p52Shc1(Y317~P,PTB!4).ErbB4(I_III!3,II!1,Y1242~P!4).HRG(EGFL!3)    Shc1kp23


# ErbB4-EGFR where ErbB4 transphosphorylates Shc
#Y086,Y1114
205 HRG(EGFL!2).ErbB4(I_III!2,II!1).p52Shc1(Y317~O,PTB!4).EGFR(I_III!3,II!1,Y992~P!4).EGF(EGFL!3) -> HRG(EGFL!2).ErbB4(I_III!2,II!1).p52Shc1(Y317~P,PTB!4).EGFR(I_III!3,II!1,Y992~P!4).EGF(EGFL!3)    Shc1kp24
206 HRG(EGFL!2).ErbB4(I_III!2,II!1).p52Shc1(Y317~O,PTB!4).EGFR(I_III!3,II!1,Y1086~P!4).EGF(EGFL!3) -> HRG(EGFL!2).ErbB4(I_III!2,II!1).p52Shc1(Y317~P,PTB!4).EGFR(I_III!3,II!1,Y1086~P!4).EGF(EGFL!3)    Shc1kp25
207 HRG(EGFL!2).ErbB4(I_III!2,II!1).p52Shc1(Y317~O,PTB!4).EGFR(I_III!3,II!1,Y1114~P!4).EGF(EGFL!3) -> HRG(EGFL!2).ErbB4(I_III!2,II!1).p52Shc1(Y317~P,PTB!4).EGFR(I_III!3,II!1,Y1114~P!4).EGF(EGFL!3)    Shc1kp26

# ErbB4-ErbB2 where ErbB4 transphosphorylates Shc
#Y1196,Y1222,Y1248
208 HRG(EGFL!2).ErbB4(I_III!2,II!1).p52Shc1(Y317~O,PTB!4).ErbB2(II!1,Y1196~P!4) -> HRG(EGFL!2).ErbB4(I_III!2,II!1).p52Shc1(Y317~P,PTB!4).ErbB2(II!1,Y1196~P!4)    Shc1kp27
209 HRG(EGFL!2).ErbB4(I_III!2,II!1).p52Shc1(Y317~O,PTB!4).ErbB2(II!1,Y1222~P!4) -> HRG(EGFL!2).ErbB4(I_III!2,II!1).p52Shc1(Y317~P,PTB!4).ErbB2(II!1,Y1222~P!4)    Shc1kp28
210 HRG(EGFL!2).ErbB4(I_III!2,II!1).p52Shc1(Y317~O,PTB!4).ErbB2(II!1,Y1248~P!4) -> HRG(EGFL!2).ErbB4(I_III!2,II!1).p52Shc1(Y317~P,PTB!4).ErbB2(II!1,Y1248~P!4)    Shc1kp29

# ErbB4-ErbB3 where ErbB4 transphosphorylates Shc
#Y1328
211 HRG(EGFL!2).ErbB4(I_III!2,II!1).p52Shc1(Y317~O,PTB!4).ErbB3(I_III!3,II!1,Y1328~P!4).HRG(EGFL!3) -> HRG(EGFL!2).ErbB4(I_III!2,II!1).p52Shc1(Y317~P,PTB!4).ErbB3(I_III!3,II!1,Y1328~P!4).HRG(EGFL!3)    Shc1kp30

# ErbB4-ErbB4 where ErbB4 transphosphorylates Shc
#Y1188,Y1242
212 HRG(EGFL!2).ErbB4(I_III!2,II!1).p52Shc1(Y317~O,PTB!4).ErbB4(I_III!3,II!1,Y1188~P!4).HRG(EGFL!3) -> HRG(EGFL!2).ErbB4(I_III!2,II!1).p52Shc1(Y317~P,PTB!4).ErbB4(I_III!3,II!1,Y1188~P!4).HRG(EGFL!3)    Shc1kp31
213 HRG(EGFL!2).ErbB4(I_III!2,II!1).p52Shc1(Y317~O,PTB!4).ErbB4(I_III!3,II!1,Y1242~P!4).HRG(EGFL!3) -> HRG(EGFL!2).ErbB4(I_III!2,II!1).p52Shc1(Y317~P,PTB!4).ErbB4(I_III!3,II!1,Y1242~P!4).HRG(EGFL!3)    Shc1kp32


# Intrinsic dephosphorylation of Phospho-Shc
214 p52Shc1(Y317~P) -> p52Shc1(Y317~O)    Shc1km14


############## SCAFFOLDING REACTIONS ##############

## These are general reactions of getting from Receptor to RAS via Sos1

# Grb2 binds to Shc
215 Grb2(SH2r,SH2s) + p52Shc1(Y317~P) <-> Grb2(SH2r,SH2s!1).p52Shc1(Y317~P!1)       Scafoldkp1,Scafoldkm1

# Sos1 binds Grb2
216 Sos1(PRS) + Grb2(nSH3,cSH3) <-> Sos1(PRS!1).Grb2(nSH3!1,cSH3)	Scafoldkp2,Scafoldkm2

#NOTE: this reaction's rate is increased to demonstrate positive feedback from the AKT pathway to the Erk pathway
# Binding of Sos1 to Gab1-Grb2
217 Sos1(PRS) + Grb2(nSH3,cSH3!2).Gab1(PRS1_PRS2!2) <-> Sos1(PRS!1).Grb2(nSH3!1,cSH3!2).Gab1(PRS1_PRS2!2)   Scafoldkp3,Scafoldkm3


############## KRAS RECRUITEMENT TO RECEPTOR ##############

# Binding of KRas to receptor bound Sos1-Grb2-rec at the GEF domain
218 Sos1(PRS!2,GEF,S1132~O,S1167~O,S1178~O,S1193~O).Grb2(nSH3!2,SH2r!+) + KRas(GTPase,g~GDP) -> Sos1(PRS!2,GEF!3,S1132~O,S1167~O,S1178~O,S1193~O).Grb2(nSH3!2,SH2r!+).KRas(GTPase!3,g~GDP)       Sos1kp1

# Binding of KRas to membrane bound Sos1-Grb2-Shc-rec at the GEF domain
219 Sos1(PRS!2,GEF,S1132~O,S1167~O,S1178~O,S1193~O).Grb2(nSH3!2,SH2s!1).p52Shc1(PTB!+,Y317~P!1) + KRas(GTPase,g~GDP) -> Sos1(PRS!2,GEF!3,S1132~O,S1167~O,S1178~O,S1193~O).Grb2(nSH3!2,SH2s!1).p52Shc1(PTB!+,Y317~P!1).KRas(GTPase!3,g~GDP)     Sos1kp1

# Binding of KRas to membrane bound Sos1-Grb2-rec at the REM domain
220 Sos1(PRS!2,REM,S1132~O,S1167~O,S1178~O,S1193~O).Grb2(nSH3!2,SH2r!+) + KRas(GTPase,g~GTP) -> Sos1(PRS!2,REM!3,S1132~O,S1167~O,S1178~O,S1193~O).Grb2(nSH3!2,SH2r!+).KRas(GTPase!3,g~GTP)     Sos1kp3

# Binding of KRas to membrane bound Sos1-Grb2-Shc-rec at the REM domain
221 Sos1(PRS!2,REM,S1132~O,S1167~O,S1178~O,S1193~O).Grb2(nSH3!2,SH2s!1).p52Shc1(PTB!+,Y317~P!1) + KRas(GTPase,g~GTP) -> Sos1(PRS!2,REM!3,S1132~O,S1167~O,S1178~O,S1193~O).Grb2(nSH3!2,SH2s!1).p52Shc1(PTB!+,Y317~P!1).KRas(GTPase!3,g~GTP)     Sos1kp3


# Ras activation by Sos
# REM domain unbound
222 Sos1(REM,GEF!3,S1132~O,S1167~O,S1178~O,S1193~O).KRas(GTPase!3,g~GDP) <-> Sos1(REM,GEF!3,S1132~O,S1167~O,S1178~O,S1193~O).KRas(GTPase!3,g~GTP)     Sos1kp5,Sos1km5

# Ras activation by Sos
# REM domain bound to Ras-GTP, this is a positive feedback loop which increases Ras activation
223 Sos1(REM!+,GEF!3,S1132~O,S1167~O,S1178~O,S1193~O).KRas(GTPase!3,g~GDP) <-> Sos1(REM!+,GEF!3,S1132~O,S1167~O,S1178~O,S1193~O).KRas(GTPase!3,g~GTP)     Sos1kp6,Sos1km6

# Intrinsic deactivation of Ras
224 KRas(GTPase,g~GTP) -> KRas(GTPase,g~GDP) Sos1kp7

# Dissociation of KRas from Sos1
# at GEF domain
225 Sos1(GEF!3).KRas(GTPase!3) -> Sos1(GEF) + KRas(GTPase)       Sos1kp8

# at REM domain
226 Sos1(REM!3).KRas(GTPase!3) -> Sos1(REM) + KRas(GTPase)       Sos1kp9

############## MAP Kinase Cascade ##############

# Activated RAS (RAS-GTP) binds Raf1
227 KRas(g~GTP,GTPase) + Raf1(RBD) -> KRas(g~GTP,GTPase!1).Raf1(RBD!1)       MAPKkp1

# Activation of Raf1 bound to Ras
228 KRas(GTPase!1,g~GTP).Raf1(RBD!1,S296~O) -> KRas(GTPase!1,g~GTP).Raf1(RBD!1,S296~P)          MAPKkp2
229 KRas(GTPase!1,g~GTP).Raf1(RBD!1,S338~O) -> KRas(GTPase!1,g~GTP).Raf1(RBD!1,S338~P)          MAPKkp3
230 KRas(GTPase!1,g~GTP).Raf1(RBD!1,Y341~O) -> KRas(GTPase!1,g~GTP).Raf1(RBD!1,Y341~P)          MAPKkp4
231 KRas(GTPase!1,g~GTP).Raf1(RBD!1,T491~O) -> KRas(GTPase!1,g~GTP).Raf1(RBD!1,T491~P)          MAPKkp5
232 KRas(GTPase!1,g~GTP).Raf1(RBD!1,S494~O) -> KRas(GTPase!1,g~GTP).Raf1(RBD!1,S494~P)          MAPKkp6

# Dissociation of activated Raf1 (MKKK) from Raf1-RAS Complex
233 Raf1(RBD!1).KRas(GTPase!1) ->  Raf1(RBD) + KRas(GTPase)              MAPKkp7

### Raf1 intrinsic dephosphorylation of activation sites
234 Raf1(S296~P) -> Raf1(S296~O)     MAPKkp8
235 Raf1(S338~P) -> Raf1(S338~O)     MAPKkp8
236 Raf1(Y341~P) -> Raf1(Y341~O)     MAPKkp8
237 Raf1(T491~P) -> Raf1(T491~O)     MAPKkp8
238 Raf1(S494~P) -> Raf1(S494~O)     MAPKkp8


### Raf1 activates MEK1

# Activated Raf1 binds to MEK1
239 Raf1(STkinase,S29~O,S43~O,S259~O!?,S289~O,S296~P,S301~O,S338~P,Y341~P,S471~O,T491~P,S494~P,S642~O) + MEK1(S218~O) -> Raf1(STkinase!1,S29~O,S43~O,S259~O!?,S289~O,S296~P,S301~O,S338~P,Y341~P,S471~O,T491~P,S494~P,S642~O).MEK1(S218~O!1)       MAPKkp9
240 Raf1(STkinase,S29~O,S43~O,S259~O!?,S289~O,S296~P,S301~O,S338~P,Y341~P,S471~O,T491~P,S494~P,S642~O) + MEK1(S222~O) -> Raf1(STkinase!1,S29~O,S43~O,S259~O!?,S289~O,S296~P,S301~O,S338~P,Y341~P,S471~O,T491~P,S494~P,S642~O).MEK1(S222~O!1)       MAPKkp10

# Activated Raf1 activates Mek1
241 Raf1(STkinase!1,S29~O,S43~O,S259~O!?,S289~O,S296~P,S301~O,S338~P,Y341~P,S471~O,T491~P,S494~P,S642~O).MEK1(S218~O!1) -> Raf1(STkinase!1,S29~O,S43~O,S259~O!?,S289~O,S296~P,S301~O,S338~P,Y341~P,S471~O,T491~P,S494~P,S642~O).MEK1(S218~P!1) MAPKkp11
242 Raf1(STkinase!1,S29~O,S43~O,S259~O!?,S289~O,S296~P,S301~O,S338~P,Y341~P,S471~O,T491~P,S494~P,S642~O).MEK1(S222~O!1) -> Raf1(STkinase!1,S29~O,S43~O,S259~O!?,S289~O,S296~P,S301~O,S338~P,Y341~P,S471~O,T491~P,S494~P,S642~O).MEK1(S222~P!1) MAPKkp12

#Raf1-Mek Dissociation
243 Raf1(STkinase!1).MEK1(S218!1) -> Raf1(STkinase) + MEK1(S218)	MAPKkp13
244 Raf1(STkinase!1).MEK1(S222!1) -> Raf1(STkinase) + MEK1(S222)	MAPKkp13

### MEK1 intrinsic dephosphorylation of activation sites
245 MEK1(S218~P) -> MEK1(S218~O)     MAPKkp15
246 MEK1(S222~P) -> MEK1(S222~O)       MAPKkp15


### MEK1 activates ERK2
# Activated MEK1 binds to ERK2
247 MEK1(delta,S218~P!?,S222~P!?,T292~O) + ERK2(CD,STkinase) -> MEK1(delta!1,S218~P!?,S222~P!?,T292~O).ERK2(CD!1,STkinase)     MAPKkp16

# Activated Mek1 phosphorylates Erk2
248 ERK2(CD!1,T185~O).MEK1(delta!1,S218~P!?,S222~P!?,T292~O) -> ERK2(CD!1,T185~P).MEK1(delta!1,S218~P!?,S222~P!?,T292~O)        MAPKkp17
249 ERK2(CD!1,Y187~O).MEK1(delta!1,S218~P!?,S222~P!?,T292~O) -> ERK2(CD!1,Y187~P).MEK1(delta!1,S218~P!?,S222~P!?,T292~O)        MAPKkp18

# Dissociation of ERK2 from MEK1
250 ERK2(CD!1).MEK1(delta!1) -> ERK2(CD) + MEK1(delta)            MAPKkp19

## ERK2 dephosphorylation
251 ERK2(Y187~P) -> ERK2(Y187~O)       MAPKkp20
252 ERK2(T185~P) -> ERK2(T185~O)       MAPKkp20


############## ERK2'S NEGATIVE FEEDBACK LOOPS ##############

## Erk2 inhibits Sos1, EGFR, Raf1, and Mek
#BINDING

#  Activated ERK2 binds with Sos1
253 ERK2(STkinase,CD,T185~P,Y187~P) + Sos1(S1132~O) -> ERK2(STkinase!1,CD,T185~P,Y187~P).Sos1(S1132~O!1)   MAPKkp21
254 ERK2(STkinase,CD,T185~P,Y187~P) + Sos1(S1167~O) -> ERK2(STkinase!1,CD,T185~P,Y187~P).Sos1(S1167~O!1)   MAPKkp22
255 ERK2(STkinase,CD,T185~P,Y187~P) + Sos1(S1178~O) -> ERK2(STkinase!1,CD,T185~P,Y187~P).Sos1(S1178~O!1)   MAPKkp23
256 ERK2(STkinase,CD,T185~P,Y187~P) + Sos1(S1193~O) -> ERK2(STkinase!1,CD,T185~P,Y187~P).Sos1(S1193~O!1)   MAPKkp24

#  ERK2 binds with EGFR
257 ERK2(STkinase,CD,T185~P,Y187~P) + EGFR(T669~O) -> ERK2(STkinase!1,CD,T185~P,Y187~P).EGFR(T669~O!1)       MAPKkp25

#  ERK2 Binds to Raf1-1
258 ERK2(STkinase,CD,T185~P,Y187~P) + Raf1(S29~O) -> ERK2(STkinase!1,CD,T185~P,Y187~P).Raf1(S29~O!1)   MAPKkp26
259 ERK2(STkinase,CD,T185~P,Y187~P) + Raf1(S43~O) -> ERK2(STkinase!1,CD,T185~P,Y187~P).Raf1(S43~O!1)   MAPKkp27
260 ERK2(STkinase,CD,T185~P,Y187~P) + Raf1(S289~O) -> ERK2(STkinase!1,CD,T185~P,Y187~P).Raf1(S289~O!1)   MAPKkp28
261 ERK2(STkinase,CD,T185~P,Y187~P) + Raf1(S301~O) -> ERK2(STkinase!1,CD,T185~P,Y187~P).Raf1(S301~O!1)   MAPKkp29
262 ERK2(STkinase,CD,T185~P,Y187~P) + Raf1(S471~O) -> ERK2(STkinase!1,CD,T185~P,Y187~P).Raf1(S471~O!1)   MAPKkp30
263 ERK2(STkinase,CD,T185~P,Y187~P) + Raf1(S642~O) -> ERK2(STkinase!1,CD,T185~P,Y187~P).Raf1(S642~O!1)   MAPKkp31

#  ERK2 Binds to MEK1
264 ERK2(STkinase,CD,T185~P,Y187~P) + MEK1(T292~O) -> ERK2(STkinase!1,CD,T185~P,Y187~P).MEK1(T292~O!1)      MAPKkp32


#  ERK2 inhibits Sos1
265 ERK2(STkinase!1,T185~P,Y187~P).Sos1(S1132~O!1) -> ERK2(STkinase!1,T185~P,Y187~P).Sos1(S1132~P!1)        MAPKkp33
266 ERK2(STkinase!1,T185~P,Y187~P).Sos1(S1167~O!1) -> ERK2(STkinase!1,T185~P,Y187~P).Sos1(S1167~P!1)        MAPKkp34
267 ERK2(STkinase!1,T185~P,Y187~P).Sos1(S1178~O!1) -> ERK2(STkinase!1,T185~P,Y187~P).Sos1(S1178~P!1)        MAPKkp35
268 ERK2(STkinase!1,T185~P,Y187~P).Sos1(S1193~O!1) -> ERK2(STkinase!1,T185~P,Y187~P).Sos1(S1193~P!1)        MAPKkp36

# ERK2 inhibits EGFR dimers.
269 ERK2(STkinase!1,T185~P,Y187~P).EGFR(T669~O!1) -> ERK2(STkinase!1,T185~P,Y187~P).EGFR(T669~P!1)      MAPKkp37

#  ERK2 inhibits Raf1-1
270 ERK2(STkinase!1,T185~P,Y187~P).Raf1(S29~O!1) -> ERK2(STkinase!1,T185~P,Y187~P).Raf1(S29~P!1)   MAPKkp38
271 ERK2(STkinase!1,T185~P,Y187~P).Raf1(S43~O!1) -> ERK2(STkinase!1,T185~P,Y187~P).Raf1(S43~P!1)   MAPKkp39
272 ERK2(STkinase!1,T185~P,Y187~P).Raf1(S289~O!1) -> ERK2(STkinase!1,T185~P,Y187~P).Raf1(S289~P!1)   MAPKkp40
273 ERK2(STkinase!1,T185~P,Y187~P).Raf1(S301~O!1) -> ERK2(STkinase!1,T185~P,Y187~P).Raf1(S301~P!1)   MAPKkp41
274 ERK2(STkinase!1,T185~P,Y187~P).Raf1(S471~O!1) -> ERK2(STkinase!1,T185~P,Y187~P).Raf1(S471~P!1)   MAPKkp42
275 ERK2(STkinase!1,T185~P,Y187~P).Raf1(S642~O!1) -> ERK2(STkinase!1,T185~P,Y187~P).Raf1(S642~P!1)   MAPKkp43

# ERK2 inhibits MEK1.
276 ERK2(STkinase!1,T185~P,Y187~P).MEK1(T292~O!1) -> ERK2(STkinase!1,T185~P,Y187~P).MEK1(T292~P!1)        MAPKkp44


#inhibitory sites intrinsic dephosphorylation

#Sos1 sites
277 Sos1(S1132~P) -> Sos1(S1132~O) MAPKkp45
278 Sos1(S1167~P) -> Sos1(S1167~O) MAPKkp45
279 Sos1(S1178~P) -> Sos1(S1178~O) MAPKkp45
280 Sos1(S1193~P) -> Sos1(S1193~O) MAPKkp45

#EGFR site
281 EGFR(T669~P) -> EGFR(T669~O)	MAPKkp46

#Raf1 sites
282 Raf1(S29~P) -> Raf1(S29~O)	MAPKkp47
283 Raf1(S43~P) -> Raf1(S43~O)	MAPKkp47
284 Raf1(S289~P) -> Raf1(S289~O)	MAPKkp47
285 Raf1(S301~P) -> Raf1(S301~O)	MAPKkp47
286 Raf1(S471~P) -> Raf1(S471~O)	MAPKkp47
287 Raf1(S642~P) -> Raf1(S642~O)	MAPKkp47

#Mek site
288 MEK1(T292~P) -> MEK1(T292~O) MAPKkp48


#ERK2 regulatory complex Dissociation
#  Erk dissasociates with Sos1
289 ERK2(STkinase!1).Sos1(S1132!1) -> ERK2(STkinase) + Sos1(S1132)	MAPKkp49
290 ERK2(STkinase!1).Sos1(S1167!1) -> ERK2(STkinase) + Sos1(S1167)	MAPKkp49
291 ERK2(STkinase!1).Sos1(S1178!1) -> ERK2(STkinase) + Sos1(S1178)	MAPKkp49
292 ERK2(STkinase!1).Sos1(S1193!1) -> ERK2(STkinase) + Sos1(S1193)	MAPKkp49

#  Erk dissasociates with EGFR
293 ERK2(STkinase!1).EGFR(T669!1) -> ERK2(STkinase) + EGFR(T669)	MAPKkp50

#  Erk dissasociates with Raf1
294 ERK2(STkinase!1).Raf1(S29!1) -> ERK2(STkinase) + Raf1(S29)	MAPKkp51
295 ERK2(STkinase!1).Raf1(S43!1) -> ERK2(STkinase) + Raf1(S43)	MAPKkp51
296 ERK2(STkinase!1).Raf1(S289!1) -> ERK2(STkinase) + Raf1(S289)	MAPKkp51
297 ERK2(STkinase!1).Raf1(S301!1) -> ERK2(STkinase) + Raf1(S301)	MAPKkp51
298 ERK2(STkinase!1).Raf1(S471!1) -> ERK2(STkinase) + Raf1(S471)	MAPKkp51
299 ERK2(STkinase!1).Raf1(S642!1) -> ERK2(STkinase) + Raf1(S642)	MAPKkp51

#  Erk dissasociates with MEK1
300 ERK2(STkinase!1).MEK1(T292!1) -> ERK2(STkinase) + MEK1(T292)	MAPKkp52


############## Gab1 interactions ##############

#Gab1 binds to membrane localized Grb2 (Grb2-p52Shc1-rec)
301 Gab1(PRS1_PRS2) + Grb2(cSH3,SH2s!2).p52Shc1(Y317~P!2,PTB!+) -> Gab1(PRS1_PRS2!1).Grb2(cSH3!1,SH2s!2).p52Shc1(Y317~P!2,PTB!+)     Gab1kp1

#Gab1 binds to membrane localized Grb2 (Grb2-rec)
302 Gab1(PRS1_PRS2) + Grb2(cSH3,SH2r!+) -> Gab1(PRS1_PRS2!1).Grb2(cSH3!1,SH2r!+)       Gab1kp1

# Transphosphorylation of Gab1 by a receptor
# EGFR-EGFR where EGFR transphosphorylates Gab1-Grb2-Shc
303 EGF(EGFL!5).EGFR(I_III!5,II!1,T669~O).Gab1(Y447~O,PRS1_PRS2!6).Grb2(cSH3!6,SH2s!2).p52Shc1(Y317~P!2,PTB!4).EGFR(I_III!3,II!1,Y992~P!4).EGF(EGFL!3) -> EGF(EGFL!5).EGFR(I_III!5,II!1,T669~O).Gab1(Y447~P,PRS1_PRS2!6).Grb2(cSH3!6,SH2s!2).p52Shc1(Y317~P!2,PTB!4).EGFR(I_III!3,II!1,Y992~P!4).EGF(EGFL!3)    Gab1kp3
304 EGF(EGFL!5).EGFR(I_III!5,II!1,T669~O).Gab1(Y472~O,PRS1_PRS2!6).Grb2(cSH3!6,SH2s!2).p52Shc1(Y317~P!2,PTB!4).EGFR(I_III!3,II!1,Y992~P!4).EGF(EGFL!3) -> EGF(EGFL!5).EGFR(I_III!5,II!1,T669~O).Gab1(Y472~P,PRS1_PRS2!6).Grb2(cSH3!6,SH2s!2).p52Shc1(Y317~P!2,PTB!4).EGFR(I_III!3,II!1,Y992~P!4).EGF(EGFL!3)    Gab1kp4
305 EGF(EGFL!5).EGFR(I_III!5,II!1,T669~O).Gab1(Y619~O,PRS1_PRS2!6).Grb2(cSH3!6,SH2s!2).p52Shc1(Y317~P!2,PTB!4).EGFR(I_III!3,II!1,Y992~P!4).EGF(EGFL!3) -> EGF(EGFL!5).EGFR(I_III!5,II!1,T669~O).Gab1(Y619~P,PRS1_PRS2!6).Grb2(cSH3!6,SH2s!2).p52Shc1(Y317~P!2,PTB!4).EGFR(I_III!3,II!1,Y992~P!4).EGF(EGFL!3)    Gab1kp5
306 EGF(EGFL!5).EGFR(I_III!5,II!1,T669~O).Gab1(Y657~O,PRS1_PRS2!6).Grb2(cSH3!6,SH2s!2).p52Shc1(Y317~P!2,PTB!4).EGFR(I_III!3,II!1,Y992~P!4).EGF(EGFL!3) -> EGF(EGFL!5).EGFR(I_III!5,II!1,T669~O).Gab1(Y657~P,PRS1_PRS2!6).Grb2(cSH3!6,SH2s!2).p52Shc1(Y317~P!2,PTB!4).EGFR(I_III!3,II!1,Y992~P!4).EGF(EGFL!3)    Gab1kp6

307 EGF(EGFL!5).EGFR(I_III!5,II!1,T669~O).Gab1(Y447~O,PRS1_PRS2!6).Grb2(cSH3!6,SH2s!2).p52Shc1(Y317~P!2,PTB!4).EGFR(I_III!3,II!1,Y1086~P!4).EGF(EGFL!3) -> EGF(EGFL!5).EGFR(I_III!5,II!1,T669~O).Gab1(Y447~P,PRS1_PRS2!6).Grb2(cSH3!6,SH2s!2).p52Shc1(Y317~P!2,PTB!4).EGFR(I_III!3,II!1,Y1086~P!4).EGF(EGFL!3)    Gab1kp7
308 EGF(EGFL!5).EGFR(I_III!5,II!1,T669~O).Gab1(Y472~O,PRS1_PRS2!6).Grb2(cSH3!6,SH2s!2).p52Shc1(Y317~P!2,PTB!4).EGFR(I_III!3,II!1,Y1086~P!4).EGF(EGFL!3) -> EGF(EGFL!5).EGFR(I_III!5,II!1,T669~O).Gab1(Y472~P,PRS1_PRS2!6).Grb2(cSH3!6,SH2s!2).p52Shc1(Y317~P!2,PTB!4).EGFR(I_III!3,II!1,Y1086~P!4).EGF(EGFL!3)    Gab1kp8
309 EGF(EGFL!5).EGFR(I_III!5,II!1,T669~O).Gab1(Y619~O,PRS1_PRS2!6).Grb2(cSH3!6,SH2s!2).p52Shc1(Y317~P!2,PTB!4).EGFR(I_III!3,II!1,Y1086~P!4).EGF(EGFL!3) -> EGF(EGFL!5).EGFR(I_III!5,II!1,T669~O).Gab1(Y619~P,PRS1_PRS2!6).Grb2(cSH3!6,SH2s!2).p52Shc1(Y317~P!2,PTB!4).EGFR(I_III!3,II!1,Y1086~P!4).EGF(EGFL!3)    Gab1kp9
310 EGF(EGFL!5).EGFR(I_III!5,II!1,T669~O).Gab1(Y657~O,PRS1_PRS2!6).Grb2(cSH3!6,SH2s!2).p52Shc1(Y317~P!2,PTB!4).EGFR(I_III!3,II!1,Y1086~P!4).EGF(EGFL!3) -> EGF(EGFL!5).EGFR(I_III!5,II!1,T669~O).Gab1(Y657~P,PRS1_PRS2!6).Grb2(cSH3!6,SH2s!2).p52Shc1(Y317~P!2,PTB!4).EGFR(I_III!3,II!1,Y1086~P!4).EGF(EGFL!3)    Gab1kp10

311 EGF(EGFL!5).EGFR(I_III!5,II!1,T669~O).Gab1(Y447~O,PRS1_PRS2!6).Grb2(cSH3!6,SH2s!2).p52Shc1(Y317~P!2,PTB!4).EGFR(I_III!3,II!1,Y1114~P!4).EGF(EGFL!3) -> EGF(EGFL!5).EGFR(I_III!5,II!1,T669~O).Gab1(Y447~P,PRS1_PRS2!6).Grb2(cSH3!6,SH2s!2).p52Shc1(Y317~P!2,PTB!4).EGFR(I_III!3,II!1,Y1114~P!4).EGF(EGFL!3)    Gab1kp11
312 EGF(EGFL!5).EGFR(I_III!5,II!1,T669~O).Gab1(Y472~O,PRS1_PRS2!6).Grb2(cSH3!6,SH2s!2).p52Shc1(Y317~P!2,PTB!4).EGFR(I_III!3,II!1,Y1114~P!4).EGF(EGFL!3) -> EGF(EGFL!5).EGFR(I_III!5,II!1,T669~O).Gab1(Y472~P,PRS1_PRS2!6).Grb2(cSH3!6,SH2s!2).p52Shc1(Y317~P!2,PTB!4).EGFR(I_III!3,II!1,Y1114~P!4).EGF(EGFL!3)    Gab1kp12
313 EGF(EGFL!5).EGFR(I_III!5,II!1,T669~O).Gab1(Y619~O,PRS1_PRS2!6).Grb2(cSH3!6,SH2s!2).p52Shc1(Y317~P!2,PTB!4).EGFR(I_III!3,II!1,Y1114~P!4).EGF(EGFL!3) -> EGF(EGFL!5).EGFR(I_III!5,II!1,T669~O).Gab1(Y619~P,PRS1_PRS2!6).Grb2(cSH3!6,SH2s!2).p52Shc1(Y317~P!2,PTB!4).EGFR(I_III!3,II!1,Y1114~P!4).EGF(EGFL!3)    Gab1kp13
314 EGF(EGFL!5).EGFR(I_III!5,II!1,T669~O).Gab1(Y657~O,PRS1_PRS2!6).Grb2(cSH3!6,SH2s!2).p52Shc1(Y317~P!2,PTB!4).EGFR(I_III!3,II!1,Y1114~P!4).EGF(EGFL!3) -> EGF(EGFL!5).EGFR(I_III!5,II!1,T669~O).Gab1(Y657~P,PRS1_PRS2!6).Grb2(cSH3!6,SH2s!2).p52Shc1(Y317~P!2,PTB!4).EGFR(I_III!3,II!1,Y1114~P!4).EGF(EGFL!3)    Gab1kp14

# EGFR-EGFR where EGFR transphosphorylates Gab1-Grb2
315 EGF(EGFL!5).EGFR(I_III!5,II!1,T669~O).Gab1(Y447~O,PRS1_PRS2!6).Grb2(cSH3!6,SH2r!4).EGFR(I_III!3,II!1,Y1068~P!4).EGF(EGFL!3) -> EGF(EGFL!5).EGFR(I_III!5,II!1,T669~O).Gab1(Y447~P,PRS1_PRS2!6).Grb2(cSH3!6,SH2r!4).EGFR(I_III!3,II!1,Y1068~P!4).EGF(EGFL!3)    Gab1kp15
316 EGF(EGFL!5).EGFR(I_III!5,II!1,T669~O).Gab1(Y472~O,PRS1_PRS2!6).Grb2(cSH3!6,SH2r!4).EGFR(I_III!3,II!1,Y1068~P!4).EGF(EGFL!3) -> EGF(EGFL!5).EGFR(I_III!5,II!1,T669~O).Gab1(Y472~P,PRS1_PRS2!6).Grb2(cSH3!6,SH2r!4).EGFR(I_III!3,II!1,Y1068~P!4).EGF(EGFL!3)    Gab1kp16
317 EGF(EGFL!5).EGFR(I_III!5,II!1,T669~O).Gab1(Y619~O,PRS1_PRS2!6).Grb2(cSH3!6,SH2r!4).EGFR(I_III!3,II!1,Y1068~P!4).EGF(EGFL!3) -> EGF(EGFL!5).EGFR(I_III!5,II!1,T669~O).Gab1(Y619~P,PRS1_PRS2!6).Grb2(cSH3!6,SH2r!4).EGFR(I_III!3,II!1,Y1068~P!4).EGF(EGFL!3)    Gab1kp17
318 EGF(EGFL!5).EGFR(I_III!5,II!1,T669~O).Gab1(Y657~O,PRS1_PRS2!6).Grb2(cSH3!6,SH2r!4).EGFR(I_III!3,II!1,Y1068~P!4).EGF(EGFL!3) -> EGF(EGFL!5).EGFR(I_III!5,II!1,T669~O).Gab1(Y657~P,PRS1_PRS2!6).Grb2(cSH3!6,SH2r!4).EGFR(I_III!3,II!1,Y1068~P!4).EGF(EGFL!3)    Gab1kp18

319 EGF(EGFL!5).EGFR(I_III!5,II!1,T669~O).Gab1(Y447~O,PRS1_PRS2!6).Grb2(cSH3!6,SH2r!4).EGFR(I_III!3,II!1,Y1114~P!4).EGF(EGFL!3) -> EGF(EGFL!5).EGFR(I_III!5,II!1,T669~O).Gab1(Y447~P,PRS1_PRS2!6).Grb2(cSH3!6,SH2r!4).EGFR(I_III!3,II!1,Y1114~P!4).EGF(EGFL!3)    Gab1kp19
320 EGF(EGFL!5).EGFR(I_III!5,II!1,T669~O).Gab1(Y472~O,PRS1_PRS2!6).Grb2(cSH3!6,SH2r!4).EGFR(I_III!3,II!1,Y1114~P!4).EGF(EGFL!3) -> EGF(EGFL!5).EGFR(I_III!5,II!1,T669~O).Gab1(Y472~P,PRS1_PRS2!6).Grb2(cSH3!6,SH2r!4).EGFR(I_III!3,II!1,Y1114~P!4).EGF(EGFL!3)    Gab1kp20
321 EGF(EGFL!5).EGFR(I_III!5,II!1,T669~O).Gab1(Y619~O,PRS1_PRS2!6).Grb2(cSH3!6,SH2r!4).EGFR(I_III!3,II!1,Y1114~P!4).EGF(EGFL!3) -> EGF(EGFL!5).EGFR(I_III!5,II!1,T669~O).Gab1(Y619~P,PRS1_PRS2!6).Grb2(cSH3!6,SH2r!4).EGFR(I_III!3,II!1,Y1114~P!4).EGF(EGFL!3)    Gab1kp21
322 EGF(EGFL!5).EGFR(I_III!5,II!1,T669~O).Gab1(Y657~O,PRS1_PRS2!6).Grb2(cSH3!6,SH2r!4).EGFR(I_III!3,II!1,Y1114~P!4).EGF(EGFL!3) -> EGF(EGFL!5).EGFR(I_III!5,II!1,T669~O).Gab1(Y657~P,PRS1_PRS2!6).Grb2(cSH3!6,SH2r!4).EGFR(I_III!3,II!1,Y1114~P!4).EGF(EGFL!3)    Gab1kp22

323 EGF(EGFL!5).EGFR(I_III!5,II!1,T669~O).Gab1(Y447~O,PRS1_PRS2!6).Grb2(cSH3!6,SH2r!4).EGFR(I_III!3,II!1,Y1148~P!4).EGF(EGFL!3) -> EGF(EGFL!5).EGFR(I_III!5,II!1,T669~O).Gab1(Y447~P,PRS1_PRS2!6).Grb2(cSH3!6,SH2r!4).EGFR(I_III!3,II!1,Y1148~P!4).EGF(EGFL!3)    Gab1kp23
324 EGF(EGFL!5).EGFR(I_III!5,II!1,T669~O).Gab1(Y472~O,PRS1_PRS2!6).Grb2(cSH3!6,SH2r!4).EGFR(I_III!3,II!1,Y1148~P!4).EGF(EGFL!3) -> EGF(EGFL!5).EGFR(I_III!5,II!1,T669~O).Gab1(Y472~P,PRS1_PRS2!6).Grb2(cSH3!6,SH2r!4).EGFR(I_III!3,II!1,Y1148~P!4).EGF(EGFL!3)    Gab1kp24
325 EGF(EGFL!5).EGFR(I_III!5,II!1,T669~O).Gab1(Y619~O,PRS1_PRS2!6).Grb2(cSH3!6,SH2r!4).EGFR(I_III!3,II!1,Y1148~P!4).EGF(EGFL!3) -> EGF(EGFL!5).EGFR(I_III!5,II!1,T669~O).Gab1(Y619~P,PRS1_PRS2!6).Grb2(cSH3!6,SH2r!4).EGFR(I_III!3,II!1,Y1148~P!4).EGF(EGFL!3)    Gab1kp25
326 EGF(EGFL!5).EGFR(I_III!5,II!1,T669~O).Gab1(Y657~O,PRS1_PRS2!6).Grb2(cSH3!6,SH2r!4).EGFR(I_III!3,II!1,Y1148~P!4).EGF(EGFL!3) -> EGF(EGFL!5).EGFR(I_III!5,II!1,T669~O).Gab1(Y657~P,PRS1_PRS2!6).Grb2(cSH3!6,SH2r!4).EGFR(I_III!3,II!1,Y1148~P!4).EGF(EGFL!3)    Gab1kp26

327 EGF(EGFL!5).EGFR(I_III!5,II!1,T669~O).Gab1(Y447~O,PRS1_PRS2!6).Grb2(cSH3!6,SH2r!4).EGFR(I_III!3,II!1,Y1173~P!4).EGF(EGFL!3) -> EGF(EGFL!5).EGFR(I_III!5,II!1,T669~O).Gab1(Y447~P,PRS1_PRS2!6).Grb2(cSH3!6,SH2r!4).EGFR(I_III!3,II!1,Y1173~P!4).EGF(EGFL!3)    Gab1kp27
328 EGF(EGFL!5).EGFR(I_III!5,II!1,T669~O).Gab1(Y472~O,PRS1_PRS2!6).Grb2(cSH3!6,SH2r!4).EGFR(I_III!3,II!1,Y1173~P!4).EGF(EGFL!3) -> EGF(EGFL!5).EGFR(I_III!5,II!1,T669~O).Gab1(Y472~P,PRS1_PRS2!6).Grb2(cSH3!6,SH2r!4).EGFR(I_III!3,II!1,Y1173~P!4).EGF(EGFL!3)    Gab1kp28
329 EGF(EGFL!5).EGFR(I_III!5,II!1,T669~O).Gab1(Y619~O,PRS1_PRS2!6).Grb2(cSH3!6,SH2r!4).EGFR(I_III!3,II!1,Y1173~P!4).EGF(EGFL!3) -> EGF(EGFL!5).EGFR(I_III!5,II!1,T669~O).Gab1(Y619~P,PRS1_PRS2!6).Grb2(cSH3!6,SH2r!4).EGFR(I_III!3,II!1,Y1173~P!4).EGF(EGFL!3)    Gab1kp29
330 EGF(EGFL!5).EGFR(I_III!5,II!1,T669~O).Gab1(Y657~O,PRS1_PRS2!6).Grb2(cSH3!6,SH2r!4).EGFR(I_III!3,II!1,Y1173~P!4).EGF(EGFL!3) -> EGF(EGFL!5).EGFR(I_III!5,II!1,T669~O).Gab1(Y657~P,PRS1_PRS2!6).Grb2(cSH3!6,SH2r!4).EGFR(I_III!3,II!1,Y1173~P!4).EGF(EGFL!3)    Gab1kp30


# EGFR-ErbB2 where EGFR transphosphorylates Gab1-Grb2-Shc
331 EGF(EGFL!5).EGFR(I_III!5,II!1,T669~O).Gab1(Y447~O,PRS1_PRS2!6).Grb2(cSH3!6,SH2s!2).p52Shc1(Y317~P!2,PTB!4).ErbB2(II!1,Y1196~P!4) -> EGF(EGFL!5).EGFR(I_III!5,II!1,T669~O).Gab1(Y447~P,PRS1_PRS2!6).Grb2(cSH3!6,SH2s!2).p52Shc1(Y317~P!2,PTB!4).ErbB2(II!1,Y1196~P!4)    Gab1kp31
332 EGF(EGFL!5).EGFR(I_III!5,II!1,T669~O).Gab1(Y472~O,PRS1_PRS2!6).Grb2(cSH3!6,SH2s!2).p52Shc1(Y317~P!2,PTB!4).ErbB2(II!1,Y1196~P!4) -> EGF(EGFL!5).EGFR(I_III!5,II!1,T669~O).Gab1(Y472~P,PRS1_PRS2!6).Grb2(cSH3!6,SH2s!2).p52Shc1(Y317~P!2,PTB!4).ErbB2(II!1,Y1196~P!4)    Gab1kp32
333 EGF(EGFL!5).EGFR(I_III!5,II!1,T669~O).Gab1(Y619~O,PRS1_PRS2!6).Grb2(cSH3!6,SH2s!2).p52Shc1(Y317~P!2,PTB!4).ErbB2(II!1,Y1196~P!4) -> EGF(EGFL!5).EGFR(I_III!5,II!1,T669~O).Gab1(Y619~P,PRS1_PRS2!6).Grb2(cSH3!6,SH2s!2).p52Shc1(Y317~P!2,PTB!4).ErbB2(II!1,Y1196~P!4)    Gab1kp33
334 EGF(EGFL!5).EGFR(I_III!5,II!1,T669~O).Gab1(Y657~O,PRS1_PRS2!6).Grb2(cSH3!6,SH2s!2).p52Shc1(Y317~P!2,PTB!4).ErbB2(II!1,Y1196~P!4) -> EGF(EGFL!5).EGFR(I_III!5,II!1,T669~O).Gab1(Y657~P,PRS1_PRS2!6).Grb2(cSH3!6,SH2s!2).p52Shc1(Y317~P!2,PTB!4).ErbB2(II!1,Y1196~P!4)    Gab1kp34

335 EGF(EGFL!5).EGFR(I_III!5,II!1,T669~O).Gab1(Y447~O,PRS1_PRS2!6).Grb2(cSH3!6,SH2s!2).p52Shc1(Y317~P!2,PTB!4).ErbB2(II!1,Y1222~P!4) -> EGF(EGFL!5).EGFR(I_III!5,II!1,T669~O).Gab1(Y447~P,PRS1_PRS2!6).Grb2(cSH3!6,SH2s!2).p52Shc1(Y317~P!2,PTB!4).ErbB2(II!1,Y1222~P!4)    Gab1kp35
336 EGF(EGFL!5).EGFR(I_III!5,II!1,T669~O).Gab1(Y472~O,PRS1_PRS2!6).Grb2(cSH3!6,SH2s!2).p52Shc1(Y317~P!2,PTB!4).ErbB2(II!1,Y1222~P!4) -> EGF(EGFL!5).EGFR(I_III!5,II!1,T669~O).Gab1(Y472~P,PRS1_PRS2!6).Grb2(cSH3!6,SH2s!2).p52Shc1(Y317~P!2,PTB!4).ErbB2(II!1,Y1222~P!4)    Gab1kp36
337 EGF(EGFL!5).EGFR(I_III!5,II!1,T669~O).Gab1(Y619~O,PRS1_PRS2!6).Grb2(cSH3!6,SH2s!2).p52Shc1(Y317~P!2,PTB!4).ErbB2(II!1,Y1222~P!4) -> EGF(EGFL!5).EGFR(I_III!5,II!1,T669~O).Gab1(Y619~P,PRS1_PRS2!6).Grb2(cSH3!6,SH2s!2).p52Shc1(Y317~P!2,PTB!4).ErbB2(II!1,Y1222~P!4)    Gab1kp37
338 EGF(EGFL!5).EGFR(I_III!5,II!1,T669~O).Gab1(Y657~O,PRS1_PRS2!6).Grb2(cSH3!6,SH2s!2).p52Shc1(Y317~P!2,PTB!4).ErbB2(II!1,Y1222~P!4) -> EGF(EGFL!5).EGFR(I_III!5,II!1,T669~O).Gab1(Y657~P,PRS1_PRS2!6).Grb2(cSH3!6,SH2s!2).p52Shc1(Y317~P!2,PTB!4).ErbB2(II!1,Y1222~P!4)    Gab1kp38

339 EGF(EGFL!5).EGFR(I_III!5,II!1,T669~O).Gab1(Y447~O,PRS1_PRS2!6).Grb2(cSH3!6,SH2s!2).p52Shc1(Y317~P!2,PTB!4).ErbB2(II!1,Y1248~P!4) -> EGF(EGFL!5).EGFR(I_III!5,II!1,T669~O).Gab1(Y447~P,PRS1_PRS2!6).Grb2(cSH3!6,SH2s!2).p52Shc1(Y317~P!2,PTB!4).ErbB2(II!1,Y1248~P!4)    Gab1kp39
340 EGF(EGFL!5).EGFR(I_III!5,II!1,T669~O).Gab1(Y472~O,PRS1_PRS2!6).Grb2(cSH3!6,SH2s!2).p52Shc1(Y317~P!2,PTB!4).ErbB2(II!1,Y1248~P!4) -> EGF(EGFL!5).EGFR(I_III!5,II!1,T669~O).Gab1(Y472~P,PRS1_PRS2!6).Grb2(cSH3!6,SH2s!2).p52Shc1(Y317~P!2,PTB!4).ErbB2(II!1,Y1248~P!4)    Gab1kp40
341 EGF(EGFL!5).EGFR(I_III!5,II!1,T669~O).Gab1(Y619~O,PRS1_PRS2!6).Grb2(cSH3!6,SH2s!2).p52Shc1(Y317~P!2,PTB!4).ErbB2(II!1,Y1248~P!4) -> EGF(EGFL!5).EGFR(I_III!5,II!1,T669~O).Gab1(Y619~P,PRS1_PRS2!6).Grb2(cSH3!6,SH2s!2).p52Shc1(Y317~P!2,PTB!4).ErbB2(II!1,Y1248~P!4)    Gab1kp41
342 EGF(EGFL!5).EGFR(I_III!5,II!1,T669~O).Gab1(Y657~O,PRS1_PRS2!6).Grb2(cSH3!6,SH2s!2).p52Shc1(Y317~P!2,PTB!4).ErbB2(II!1,Y1248~P!4) -> EGF(EGFL!5).EGFR(I_III!5,II!1,T669~O).Gab1(Y657~P,PRS1_PRS2!6).Grb2(cSH3!6,SH2s!2).p52Shc1(Y317~P!2,PTB!4).ErbB2(II!1,Y1248~P!4)    Gab1kp42


# EGFR-ErbB2 where EGFR transphosphorylates Gab1-Grb2
343 EGF(EGFL!5).EGFR(I_III!5,II!1,T669~O).Gab1(Y447~O,PRS1_PRS2!6).Grb2(cSH3!6,SH2r!4).ErbB2(II!1,Y1139~P!4) -> EGF(EGFL!5).EGFR(I_III!5,II!1,T669~O).Gab1(Y447~P,PRS1_PRS2!6).Grb2(cSH3!6,SH2r!4).ErbB2(II!1,Y1139~P!4)    Gab1kp43
344 EGF(EGFL!5).EGFR(I_III!5,II!1,T669~O).Gab1(Y472~O,PRS1_PRS2!6).Grb2(cSH3!6,SH2r!4).ErbB2(II!1,Y1139~P!4) -> EGF(EGFL!5).EGFR(I_III!5,II!1,T669~O).Gab1(Y472~P,PRS1_PRS2!6).Grb2(cSH3!6,SH2r!4).ErbB2(II!1,Y1139~P!4)    Gab1kp44
345 EGF(EGFL!5).EGFR(I_III!5,II!1,T669~O).Gab1(Y619~O,PRS1_PRS2!6).Grb2(cSH3!6,SH2r!4).ErbB2(II!1,Y1139~P!4) -> EGF(EGFL!5).EGFR(I_III!5,II!1,T669~O).Gab1(Y619~P,PRS1_PRS2!6).Grb2(cSH3!6,SH2r!4).ErbB2(II!1,Y1139~P!4)    Gab1kp45
346 EGF(EGFL!5).EGFR(I_III!5,II!1,T669~O).Gab1(Y657~O,PRS1_PRS2!6).Grb2(cSH3!6,SH2r!4).ErbB2(II!1,Y1139~P!4) -> EGF(EGFL!5).EGFR(I_III!5,II!1,T669~O).Gab1(Y657~P,PRS1_PRS2!6).Grb2(cSH3!6,SH2r!4).ErbB2(II!1,Y1139~P!4)    Gab1kp46

# EGFR-ErbB3 where EGFR transphosphorylates Gab1-Grb2-Shc
347 EGF(EGFL!5).EGFR(I_III!5,II!1,T669~O).Gab1(Y447~O,PRS1_PRS2!6).Grb2(cSH3!6,SH2s!2).p52Shc1(Y317~P!2,PTB!4).ErbB3(I_III!3,II!1,Y1328~P!4).HRG(EGFL!3) -> EGF(EGFL!5).EGFR(I_III!5,II!1,T669~O).Gab1(Y447~P,PRS1_PRS2!6).Grb2(cSH3!6,SH2s!2).p52Shc1(Y317~P!2,PTB!4).ErbB3(I_III!3,II!1,Y1328~P!4).HRG(EGFL!3)    Gab1kp47
348 EGF(EGFL!5).EGFR(I_III!5,II!1,T669~O).Gab1(Y472~O,PRS1_PRS2!6).Grb2(cSH3!6,SH2s!2).p52Shc1(Y317~P!2,PTB!4).ErbB3(I_III!3,II!1,Y1328~P!4).HRG(EGFL!3) -> EGF(EGFL!5).EGFR(I_III!5,II!1,T669~O).Gab1(Y472~P,PRS1_PRS2!6).Grb2(cSH3!6,SH2s!2).p52Shc1(Y317~P!2,PTB!4).ErbB3(I_III!3,II!1,Y1328~P!4).HRG(EGFL!3)	Gab1kp48
349 EGF(EGFL!5).EGFR(I_III!5,II!1,T669~O).Gab1(Y619~O,PRS1_PRS2!6).Grb2(cSH3!6,SH2s!2).p52Shc1(Y317~P!2,PTB!4).ErbB3(I_III!3,II!1,Y1328~P!4).HRG(EGFL!3) -> EGF(EGFL!5).EGFR(I_III!5,II!1,T669~O).Gab1(Y619~P,PRS1_PRS2!6).Grb2(cSH3!6,SH2s!2).p52Shc1(Y317~P!2,PTB!4).ErbB3(I_III!3,II!1,Y1328~P!4).HRG(EGFL!3)    Gab1kp49
350 EGF(EGFL!5).EGFR(I_III!5,II!1,T669~O).Gab1(Y657~O,PRS1_PRS2!6).Grb2(cSH3!6,SH2s!2).p52Shc1(Y317~P!2,PTB!4).ErbB3(I_III!3,II!1,Y1328~P!4).HRG(EGFL!3) -> EGF(EGFL!5).EGFR(I_III!5,II!1,T669~O).Gab1(Y657~P,PRS1_PRS2!6).Grb2(cSH3!6,SH2s!2).p52Shc1(Y317~P!2,PTB!4).ErbB3(I_III!3,II!1,Y1328~P!4).HRG(EGFL!3)    Gab1kp50


# EGFR-ErbB4 where EGFR transphosphorylates Gab1-Grb2-Shc
351 EGF(EGFL!5).EGFR(I_III!5,II!1,T669~O).Gab1(Y447~O,PRS1_PRS2!6).Grb2(cSH3!6,SH2s!2).p52Shc1(Y317~P!2,PTB!4).ErbB4(I_III!3,II!1,Y1188~P!4).HRG(EGFL!3) -> EGF(EGFL!5).EGFR(I_III!5,II!1,T669~O).Gab1(Y447~P,PRS1_PRS2!6).Grb2(cSH3!6,SH2s!2).p52Shc1(Y317~P!2,PTB!4).ErbB4(I_III!3,II!1,Y1188~P!4).HRG(EGFL!3)    Gab1kp51
352 EGF(EGFL!5).EGFR(I_III!5,II!1,T669~O).Gab1(Y472~O,PRS1_PRS2!6).Grb2(cSH3!6,SH2s!2).p52Shc1(Y317~P!2,PTB!4).ErbB4(I_III!3,II!1,Y1188~P!4).HRG(EGFL!3) -> EGF(EGFL!5).EGFR(I_III!5,II!1,T669~O).Gab1(Y472~P,PRS1_PRS2!6).Grb2(cSH3!6,SH2s!2).p52Shc1(Y317~P!2,PTB!4).ErbB4(I_III!3,II!1,Y1188~P!4).HRG(EGFL!3)	Gab1kp52
353 EGF(EGFL!5).EGFR(I_III!5,II!1,T669~O).Gab1(Y619~O,PRS1_PRS2!6).Grb2(cSH3!6,SH2s!2).p52Shc1(Y317~P!2,PTB!4).ErbB4(I_III!3,II!1,Y1188~P!4).HRG(EGFL!3) -> EGF(EGFL!5).EGFR(I_III!5,II!1,T669~O).Gab1(Y619~P,PRS1_PRS2!6).Grb2(cSH3!6,SH2s!2).p52Shc1(Y317~P!2,PTB!4).ErbB4(I_III!3,II!1,Y1188~P!4).HRG(EGFL!3)    Gab1kp53
354 EGF(EGFL!5).EGFR(I_III!5,II!1,T669~O).Gab1(Y657~O,PRS1_PRS2!6).Grb2(cSH3!6,SH2s!2).p52Shc1(Y317~P!2,PTB!4).ErbB4(I_III!3,II!1,Y1188~P!4).HRG(EGFL!3) -> EGF(EGFL!5).EGFR(I_III!5,II!1,T669~O).Gab1(Y657~P,PRS1_PRS2!6).Grb2(cSH3!6,SH2s!2).p52Shc1(Y317~P!2,PTB!4).ErbB4(I_III!3,II!1,Y1188~P!4).HRG(EGFL!3)    Gab1kp54

355 EGF(EGFL!5).EGFR(I_III!5,II!1,T669~O).Gab1(Y447~O,PRS1_PRS2!6).Grb2(cSH3!6,SH2s!2).p52Shc1(Y317~P!2,PTB!4).ErbB4(I_III!3,II!1,Y1242~P!4).HRG(EGFL!3) -> EGF(EGFL!5).EGFR(I_III!5,II!1,T669~O).Gab1(Y447~P,PRS1_PRS2!6).Grb2(cSH3!6,SH2s!2).p52Shc1(Y317~P!2,PTB!4).ErbB4(I_III!3,II!1,Y1242~P!4).HRG(EGFL!3)    Gab1kp55
356 EGF(EGFL!5).EGFR(I_III!5,II!1,T669~O).Gab1(Y472~O,PRS1_PRS2!6).Grb2(cSH3!6,SH2s!2).p52Shc1(Y317~P!2,PTB!4).ErbB4(I_III!3,II!1,Y1242~P!4).HRG(EGFL!3) -> EGF(EGFL!5).EGFR(I_III!5,II!1,T669~O).Gab1(Y472~P,PRS1_PRS2!6).Grb2(cSH3!6,SH2s!2).p52Shc1(Y317~P!2,PTB!4).ErbB4(I_III!3,II!1,Y1242~P!4).HRG(EGFL!3)	Gab1kp56
357 EGF(EGFL!5).EGFR(I_III!5,II!1,T669~O).Gab1(Y619~O,PRS1_PRS2!6).Grb2(cSH3!6,SH2s!2).p52Shc1(Y317~P!2,PTB!4).ErbB4(I_III!3,II!1,Y1242~P!4).HRG(EGFL!3) -> EGF(EGFL!5).EGFR(I_III!5,II!1,T669~O).Gab1(Y619~P,PRS1_PRS2!6).Grb2(cSH3!6,SH2s!2).p52Shc1(Y317~P!2,PTB!4).ErbB4(I_III!3,II!1,Y1242~P!4).HRG(EGFL!3)    Gab1kp57
358 EGF(EGFL!5).EGFR(I_III!5,II!1,T669~O).Gab1(Y657~O,PRS1_PRS2!6).Grb2(cSH3!6,SH2s!2).p52Shc1(Y317~P!2,PTB!4).ErbB4(I_III!3,II!1,Y1242~P!4).HRG(EGFL!3) -> EGF(EGFL!5).EGFR(I_III!5,II!1,T669~O).Gab1(Y657~P,PRS1_PRS2!6).Grb2(cSH3!6,SH2s!2).p52Shc1(Y317~P!2,PTB!4).ErbB4(I_III!3,II!1,Y1242~P!4).HRG(EGFL!3)    Gab1kp58


# ErbB2-EGFR where ErbB2 transphosphorylates Gab1-Grb2-Shc
359 ErbB2(II!1).Gab1(Y447~O,PRS1_PRS2!6).Grb2(cSH3!6,SH2s!2).p52Shc1(Y317~P!2,PTB!4).EGFR(I_III!3,II!1,Y992~P!4).EGF(EGFL!3) -> ErbB2(II!1).Gab1(Y447~P,PRS1_PRS2!6).Grb2(cSH3!6,SH2s!2).p52Shc1(Y317~P!2,PTB!4).EGFR(I_III!3,II!1,Y992~P!4).EGF(EGFL!3)    Gab1kp59
360 ErbB2(II!1).Gab1(Y472~O,PRS1_PRS2!6).Grb2(cSH3!6,SH2s!2).p52Shc1(Y317~P!2,PTB!4).EGFR(I_III!3,II!1,Y992~P!4).EGF(EGFL!3) -> ErbB2(II!1).Gab1(Y472~P,PRS1_PRS2!6).Grb2(cSH3!6,SH2s!2).p52Shc1(Y317~P!2,PTB!4).EGFR(I_III!3,II!1,Y992~P!4).EGF(EGFL!3)    Gab1kp60
361 ErbB2(II!1).Gab1(Y619~O,PRS1_PRS2!6).Grb2(cSH3!6,SH2s!2).p52Shc1(Y317~P!2,PTB!4).EGFR(I_III!3,II!1,Y992~P!4).EGF(EGFL!3) -> ErbB2(II!1).Gab1(Y619~P,PRS1_PRS2!6).Grb2(cSH3!6,SH2s!2).p52Shc1(Y317~P!2,PTB!4).EGFR(I_III!3,II!1,Y992~P!4).EGF(EGFL!3)    Gab1kp61
362 ErbB2(II!1).Gab1(Y657~O,PRS1_PRS2!6).Grb2(cSH3!6,SH2s!2).p52Shc1(Y317~P!2,PTB!4).EGFR(I_III!3,II!1,Y992~P!4).EGF(EGFL!3) -> ErbB2(II!1).Gab1(Y657~P,PRS1_PRS2!6).Grb2(cSH3!6,SH2s!2).p52Shc1(Y317~P!2,PTB!4).EGFR(I_III!3,II!1,Y992~P!4).EGF(EGFL!3)    Gab1kp62

363 ErbB2(II!1).Gab1(Y447~O,PRS1_PRS2!6).Grb2(cSH3!6,SH2s!2).p52Shc1(Y317~P!2,PTB!4).EGFR(I_III!3,II!1,Y1086~P!4).EGF(EGFL!3) -> ErbB2(II!1).Gab1(Y447~P,PRS1_PRS2!6).Grb2(cSH3!6,SH2s!2).p52Shc1(Y317~P!2,PTB!4).EGFR(I_III!3,II!1,Y1086~P!4).EGF(EGFL!3)    Gab1kp63
364 ErbB2(II!1).Gab1(Y472~O,PRS1_PRS2!6).Grb2(cSH3!6,SH2s!2).p52Shc1(Y317~P!2,PTB!4).EGFR(I_III!3,II!1,Y1086~P!4).EGF(EGFL!3) -> ErbB2(II!1).Gab1(Y472~P,PRS1_PRS2!6).Grb2(cSH3!6,SH2s!2).p52Shc1(Y317~P!2,PTB!4).EGFR(I_III!3,II!1,Y1086~P!4).EGF(EGFL!3)    Gab1kp64
365 ErbB2(II!1).Gab1(Y619~O,PRS1_PRS2!6).Grb2(cSH3!6,SH2s!2).p52Shc1(Y317~P!2,PTB!4).EGFR(I_III!3,II!1,Y1086~P!4).EGF(EGFL!3) -> ErbB2(II!1).Gab1(Y619~P,PRS1_PRS2!6).Grb2(cSH3!6,SH2s!2).p52Shc1(Y317~P!2,PTB!4).EGFR(I_III!3,II!1,Y1086~P!4).EGF(EGFL!3)    Gab1kp65
366 ErbB2(II!1).Gab1(Y657~O,PRS1_PRS2!6).Grb2(cSH3!6,SH2s!2).p52Shc1(Y317~P!2,PTB!4).EGFR(I_III!3,II!1,Y1086~P!4).EGF(EGFL!3) -> ErbB2(II!1).Gab1(Y657~P,PRS1_PRS2!6).Grb2(cSH3!6,SH2s!2).p52Shc1(Y317~P!2,PTB!4).EGFR(I_III!3,II!1,Y1086~P!4).EGF(EGFL!3)    Gab1kp66

367 ErbB2(II!1).Gab1(Y447~O,PRS1_PRS2!6).Grb2(cSH3!6,SH2s!2).p52Shc1(Y317~P!2,PTB!4).EGFR(I_III!3,II!1,Y1114~P!4).EGF(EGFL!3) -> ErbB2(II!1).Gab1(Y447~P,PRS1_PRS2!6).Grb2(cSH3!6,SH2s!2).p52Shc1(Y317~P!2,PTB!4).EGFR(I_III!3,II!1,Y1114~P!4).EGF(EGFL!3)    Gab1kp67
368 ErbB2(II!1).Gab1(Y472~O,PRS1_PRS2!6).Grb2(cSH3!6,SH2s!2).p52Shc1(Y317~P!2,PTB!4).EGFR(I_III!3,II!1,Y1114~P!4).EGF(EGFL!3) -> ErbB2(II!1).Gab1(Y472~P,PRS1_PRS2!6).Grb2(cSH3!6,SH2s!2).p52Shc1(Y317~P!2,PTB!4).EGFR(I_III!3,II!1,Y1114~P!4).EGF(EGFL!3)    Gab1kp68
369 ErbB2(II!1).Gab1(Y619~O,PRS1_PRS2!6).Grb2(cSH3!6,SH2s!2).p52Shc1(Y317~P!2,PTB!4).EGFR(I_III!3,II!1,Y1114~P!4).EGF(EGFL!3) -> ErbB2(II!1).Gab1(Y619~P,PRS1_PRS2!6).Grb2(cSH3!6,SH2s!2).p52Shc1(Y317~P!2,PTB!4).EGFR(I_III!3,II!1,Y1114~P!4).EGF(EGFL!3)    Gab1kp69
370 ErbB2(II!1).Gab1(Y657~O,PRS1_PRS2!6).Grb2(cSH3!6,SH2s!2).p52Shc1(Y317~P!2,PTB!4).EGFR(I_III!3,II!1,Y1114~P!4).EGF(EGFL!3) -> ErbB2(II!1).Gab1(Y657~P,PRS1_PRS2!6).Grb2(cSH3!6,SH2s!2).p52Shc1(Y317~P!2,PTB!4).EGFR(I_III!3,II!1,Y1114~P!4).EGF(EGFL!3)    Gab1kp70

# ErbB2-EGFR where ErbB2 transphosphorylates Gab1-Grb2
371 ErbB2(II!1).Gab1(Y447~O,PRS1_PRS2!6).Grb2(cSH3!6,SH2r!4).EGFR(I_III!3,II!1,Y1068~P!4).EGF(EGFL!3) -> ErbB2(II!1).Gab1(Y447~P,PRS1_PRS2!6).Grb2(cSH3!6,SH2r!4).EGFR(I_III!3,II!1,Y1068~P!4).EGF(EGFL!3)    Gab1kp71
372 ErbB2(II!1).Gab1(Y472~O,PRS1_PRS2!6).Grb2(cSH3!6,SH2r!4).EGFR(I_III!3,II!1,Y1068~P!4).EGF(EGFL!3) -> ErbB2(II!1).Gab1(Y472~P,PRS1_PRS2!6).Grb2(cSH3!6,SH2r!4).EGFR(I_III!3,II!1,Y1068~P!4).EGF(EGFL!3)    Gab1kp72
373 ErbB2(II!1).Gab1(Y619~O,PRS1_PRS2!6).Grb2(cSH3!6,SH2r!4).EGFR(I_III!3,II!1,Y1068~P!4).EGF(EGFL!3) -> ErbB2(II!1).Gab1(Y619~P,PRS1_PRS2!6).Grb2(cSH3!6,SH2r!4).EGFR(I_III!3,II!1,Y1068~P!4).EGF(EGFL!3)    Gab1kp73
374 ErbB2(II!1).Gab1(Y657~O,PRS1_PRS2!6).Grb2(cSH3!6,SH2r!4).EGFR(I_III!3,II!1,Y1068~P!4).EGF(EGFL!3) -> ErbB2(II!1).Gab1(Y657~P,PRS1_PRS2!6).Grb2(cSH3!6,SH2r!4).EGFR(I_III!3,II!1,Y1068~P!4).EGF(EGFL!3)    Gab1kp74

375 ErbB2(II!1).Gab1(Y447~O,PRS1_PRS2!6).Grb2(cSH3!6,SH2r!4).EGFR(I_III!3,II!1,Y1114~P!4).EGF(EGFL!3) -> ErbB2(II!1).Gab1(Y447~P,PRS1_PRS2!6).Grb2(cSH3!6,SH2r!4).EGFR(I_III!3,II!1,Y1114~P!4).EGF(EGFL!3)    Gab1kp75
376 ErbB2(II!1).Gab1(Y472~O,PRS1_PRS2!6).Grb2(cSH3!6,SH2r!4).EGFR(I_III!3,II!1,Y1114~P!4).EGF(EGFL!3) -> ErbB2(II!1).Gab1(Y472~P,PRS1_PRS2!6).Grb2(cSH3!6,SH2r!4).EGFR(I_III!3,II!1,Y1114~P!4).EGF(EGFL!3)    Gab1kp76
377 ErbB2(II!1).Gab1(Y619~O,PRS1_PRS2!6).Grb2(cSH3!6,SH2r!4).EGFR(I_III!3,II!1,Y1114~P!4).EGF(EGFL!3) -> ErbB2(II!1).Gab1(Y619~P,PRS1_PRS2!6).Grb2(cSH3!6,SH2r!4).EGFR(I_III!3,II!1,Y1114~P!4).EGF(EGFL!3)    Gab1kp77
378 ErbB2(II!1).Gab1(Y657~O,PRS1_PRS2!6).Grb2(cSH3!6,SH2r!4).EGFR(I_III!3,II!1,Y1114~P!4).EGF(EGFL!3) -> ErbB2(II!1).Gab1(Y657~P,PRS1_PRS2!6).Grb2(cSH3!6,SH2r!4).EGFR(I_III!3,II!1,Y1114~P!4).EGF(EGFL!3)    Gab1kp78

379 ErbB2(II!1).Gab1(Y447~O,PRS1_PRS2!6).Grb2(cSH3!6,SH2r!4).EGFR(I_III!3,II!1,Y1148~P!4).EGF(EGFL!3) -> ErbB2(II!1).Gab1(Y447~P,PRS1_PRS2!6).Grb2(cSH3!6,SH2r!4).EGFR(I_III!3,II!1,Y1148~P!4).EGF(EGFL!3)    Gab1kp79
380 ErbB2(II!1).Gab1(Y472~O,PRS1_PRS2!6).Grb2(cSH3!6,SH2r!4).EGFR(I_III!3,II!1,Y1148~P!4).EGF(EGFL!3) -> ErbB2(II!1).Gab1(Y472~P,PRS1_PRS2!6).Grb2(cSH3!6,SH2r!4).EGFR(I_III!3,II!1,Y1148~P!4).EGF(EGFL!3)    Gab1kp80
381 ErbB2(II!1).Gab1(Y619~O,PRS1_PRS2!6).Grb2(cSH3!6,SH2r!4).EGFR(I_III!3,II!1,Y1148~P!4).EGF(EGFL!3) -> ErbB2(II!1).Gab1(Y619~P,PRS1_PRS2!6).Grb2(cSH3!6,SH2r!4).EGFR(I_III!3,II!1,Y1148~P!4).EGF(EGFL!3)    Gab1kp81
382 ErbB2(II!1).Gab1(Y657~O,PRS1_PRS2!6).Grb2(cSH3!6,SH2r!4).EGFR(I_III!3,II!1,Y1148~P!4).EGF(EGFL!3) -> ErbB2(II!1).Gab1(Y657~P,PRS1_PRS2!6).Grb2(cSH3!6,SH2r!4).EGFR(I_III!3,II!1,Y1148~P!4).EGF(EGFL!3)    Gab1kp82

383 ErbB2(II!1).Gab1(Y447~O,PRS1_PRS2!6).Grb2(cSH3!6,SH2r!4).EGFR(I_III!3,II!1,Y1173~P!4).EGF(EGFL!3) -> ErbB2(II!1).Gab1(Y447~P,PRS1_PRS2!6).Grb2(cSH3!6,SH2r!4).EGFR(I_III!3,II!1,Y1173~P!4).EGF(EGFL!3)    Gab1kp83
384 ErbB2(II!1).Gab1(Y472~O,PRS1_PRS2!6).Grb2(cSH3!6,SH2r!4).EGFR(I_III!3,II!1,Y1173~P!4).EGF(EGFL!3) -> ErbB2(II!1).Gab1(Y472~P,PRS1_PRS2!6).Grb2(cSH3!6,SH2r!4).EGFR(I_III!3,II!1,Y1173~P!4).EGF(EGFL!3)    Gab1kp84
385 ErbB2(II!1).Gab1(Y619~O,PRS1_PRS2!6).Grb2(cSH3!6,SH2r!4).EGFR(I_III!3,II!1,Y1173~P!4).EGF(EGFL!3) -> ErbB2(II!1).Gab1(Y619~P,PRS1_PRS2!6).Grb2(cSH3!6,SH2r!4).EGFR(I_III!3,II!1,Y1173~P!4).EGF(EGFL!3)    Gab1kp85
386 ErbB2(II!1).Gab1(Y657~O,PRS1_PRS2!6).Grb2(cSH3!6,SH2r!4).EGFR(I_III!3,II!1,Y1173~P!4).EGF(EGFL!3) -> ErbB2(II!1).Gab1(Y657~P,PRS1_PRS2!6).Grb2(cSH3!6,SH2r!4).EGFR(I_III!3,II!1,Y1173~P!4).EGF(EGFL!3)    Gab1kp86


# ErbB2-ErbB3 where ErbB2 transphosphorylates Gab1-Grb2-Shc
387 ErbB2(II!1).Gab1(Y447~O,PRS1_PRS2!5).Grb2(cSH3!5,SH2s!2).p52Shc1(Y317~P!2,PTB!4).ErbB3(I_III!3,II!1,Y1328~P!4).HRG(EGFL!3) -> ErbB2(II!1).Gab1(Y447~P,PRS1_PRS2!5).Grb2(cSH3!5,SH2s!2).p52Shc1(Y317~P!2,PTB!4).ErbB3(I_III!3,II!1,Y1328~P!4).HRG(EGFL!3)    Gab1kp87
388 ErbB2(II!1).Gab1(Y472~O,PRS1_PRS2!5).Grb2(cSH3!5,SH2s!2).p52Shc1(Y317~P!2,PTB!4).ErbB3(I_III!3,II!1,Y1328~P!4).HRG(EGFL!3) -> ErbB2(II!1).Gab1(Y472~P,PRS1_PRS2!5).Grb2(cSH3!5,SH2s!2).p52Shc1(Y317~P!2,PTB!4).ErbB3(I_III!3,II!1,Y1328~P!4).HRG(EGFL!3)    Gab1kp88
389 ErbB2(II!1).Gab1(Y619~O,PRS1_PRS2!5).Grb2(cSH3!5,SH2s!2).p52Shc1(Y317~P!2,PTB!4).ErbB3(I_III!3,II!1,Y1328~P!4).HRG(EGFL!3) -> ErbB2(II!1).Gab1(Y619~P,PRS1_PRS2!5).Grb2(cSH3!5,SH2s!2).p52Shc1(Y317~P!2,PTB!4).ErbB3(I_III!3,II!1,Y1328~P!4).HRG(EGFL!3)    Gab1kp89
390 ErbB2(II!1).Gab1(Y657~O,PRS1_PRS2!5).Grb2(cSH3!5,SH2s!2).p52Shc1(Y317~P!2,PTB!4).ErbB3(I_III!3,II!1,Y1328~P!4).HRG(EGFL!3) -> ErbB2(II!1).Gab1(Y657~P,PRS1_PRS2!5).Grb2(cSH3!5,SH2s!2).p52Shc1(Y317~P!2,PTB!4).ErbB3(I_III!3,II!1,Y1328~P!4).HRG(EGFL!3)    Gab1kp90


# ErbB2-ErbB4 where ErbB2 transphosphorylates Gab1-Grb2-Shc
391 ErbB2(II!1).Gab1(Y447~O,PRS1_PRS2!6).Grb2(cSH3!6,SH2s!2).p52Shc1(Y317~P!2,PTB!4).ErbB4(I_III!3,II!1,Y1188~P!4).HRG(EGFL!3) -> ErbB2(II!1).Gab1(Y447~P,PRS1_PRS2!6).Grb2(cSH3!6,SH2s!2).p52Shc1(Y317~P!2,PTB!4).ErbB4(I_III!3,II!1,Y1188~P!4).HRG(EGFL!3)    Gab1kp91
392 ErbB2(II!1).Gab1(Y472~O,PRS1_PRS2!6).Grb2(cSH3!6,SH2s!2).p52Shc1(Y317~P!2,PTB!4).ErbB4(I_III!3,II!1,Y1188~P!4).HRG(EGFL!3) -> ErbB2(II!1).Gab1(Y472~P,PRS1_PRS2!6).Grb2(cSH3!6,SH2s!2).p52Shc1(Y317~P!2,PTB!4).ErbB4(I_III!3,II!1,Y1188~P!4).HRG(EGFL!3)	Gab1kp92
393 ErbB2(II!1).Gab1(Y619~O,PRS1_PRS2!6).Grb2(cSH3!6,SH2s!2).p52Shc1(Y317~P!2,PTB!4).ErbB4(I_III!3,II!1,Y1188~P!4).HRG(EGFL!3) -> ErbB2(II!1).Gab1(Y619~P,PRS1_PRS2!6).Grb2(cSH3!6,SH2s!2).p52Shc1(Y317~P!2,PTB!4).ErbB4(I_III!3,II!1,Y1188~P!4).HRG(EGFL!3)    Gab1kp93
394 ErbB2(II!1).Gab1(Y657~O,PRS1_PRS2!6).Grb2(cSH3!6,SH2s!2).p52Shc1(Y317~P!2,PTB!4).ErbB4(I_III!3,II!1,Y1188~P!4).HRG(EGFL!3) -> ErbB2(II!1).Gab1(Y657~P,PRS1_PRS2!6).Grb2(cSH3!6,SH2s!2).p52Shc1(Y317~P!2,PTB!4).ErbB4(I_III!3,II!1,Y1188~P!4).HRG(EGFL!3)    Gab1kp94

395 ErbB2(II!1).Gab1(Y447~O,PRS1_PRS2!6).Grb2(cSH3!6,SH2s!2).p52Shc1(Y317~P!2,PTB!4).ErbB4(I_III!3,II!1,Y1242~P!4).HRG(EGFL!3) -> ErbB2(II!1).Gab1(Y447~P,PRS1_PRS2!6).Grb2(cSH3!6,SH2s!2).p52Shc1(Y317~P!2,PTB!4).ErbB4(I_III!3,II!1,Y1242~P!4).HRG(EGFL!3)    Gab1kp95
396 ErbB2(II!1).Gab1(Y472~O,PRS1_PRS2!6).Grb2(cSH3!6,SH2s!2).p52Shc1(Y317~P!2,PTB!4).ErbB4(I_III!3,II!1,Y1242~P!4).HRG(EGFL!3) -> ErbB2(II!1).Gab1(Y472~P,PRS1_PRS2!6).Grb2(cSH3!6,SH2s!2).p52Shc1(Y317~P!2,PTB!4).ErbB4(I_III!3,II!1,Y1242~P!4).HRG(EGFL!3)	Gab1kp96
397 ErbB2(II!1).Gab1(Y619~O,PRS1_PRS2!6).Grb2(cSH3!6,SH2s!2).p52Shc1(Y317~P!2,PTB!4).ErbB4(I_III!3,II!1,Y1242~P!4).HRG(EGFL!3) -> ErbB2(II!1).Gab1(Y619~P,PRS1_PRS2!6).Grb2(cSH3!6,SH2s!2).p52Shc1(Y317~P!2,PTB!4).ErbB4(I_III!3,II!1,Y1242~P!4).HRG(EGFL!3)    Gab1kp97
398 ErbB2(II!1).Gab1(Y657~O,PRS1_PRS2!6).Grb2(cSH3!6,SH2s!2).p52Shc1(Y317~P!2,PTB!4).ErbB4(I_III!3,II!1,Y1242~P!4).HRG(EGFL!3) -> ErbB2(II!1).Gab1(Y657~P,PRS1_PRS2!6).Grb2(cSH3!6,SH2s!2).p52Shc1(Y317~P!2,PTB!4).ErbB4(I_III!3,II!1,Y1242~P!4).HRG(EGFL!3)    Gab1kp98


# Transphosphorylation of Gab1 by a receptor
# ErbB4-EGFR where ErbB4 transphosphorylates Gab1-Grb2-Shc
399 HRG(EGFL!5).ErbB4(I_III!5,II!1).Gab1(Y447~O,PRS1_PRS2!6).Grb2(cSH3!6,SH2s!2).p52Shc1(Y317~P!2,PTB!4).EGFR(I_III!3,II!1,Y992~P!4).EGF(EGFL!3) -> HRG(EGFL!5).ErbB4(I_III!5,II!1).Gab1(Y447~P,PRS1_PRS2!6).Grb2(cSH3!6,SH2s!2).p52Shc1(Y317~P!2,PTB!4).EGFR(I_III!3,II!1,Y992~P!4).EGF(EGFL!3)    Gab1kp99
400 HRG(EGFL!5).ErbB4(I_III!5,II!1).Gab1(Y472~O,PRS1_PRS2!6).Grb2(cSH3!6,SH2s!2).p52Shc1(Y317~P!2,PTB!4).EGFR(I_III!3,II!1,Y992~P!4).EGF(EGFL!3) -> HRG(EGFL!5).ErbB4(I_III!5,II!1).Gab1(Y472~P,PRS1_PRS2!6).Grb2(cSH3!6,SH2s!2).p52Shc1(Y317~P!2,PTB!4).EGFR(I_III!3,II!1,Y992~P!4).EGF(EGFL!3)    Gab1kp100
401 HRG(EGFL!5).ErbB4(I_III!5,II!1).Gab1(Y619~O,PRS1_PRS2!6).Grb2(cSH3!6,SH2s!2).p52Shc1(Y317~P!2,PTB!4).EGFR(I_III!3,II!1,Y992~P!4).EGF(EGFL!3) -> HRG(EGFL!5).ErbB4(I_III!5,II!1).Gab1(Y619~P,PRS1_PRS2!6).Grb2(cSH3!6,SH2s!2).p52Shc1(Y317~P!2,PTB!4).EGFR(I_III!3,II!1,Y992~P!4).EGF(EGFL!3)    Gab1kp101
402 HRG(EGFL!5).ErbB4(I_III!5,II!1).Gab1(Y657~O,PRS1_PRS2!6).Grb2(cSH3!6,SH2s!2).p52Shc1(Y317~P!2,PTB!4).EGFR(I_III!3,II!1,Y992~P!4).EGF(EGFL!3) -> HRG(EGFL!5).ErbB4(I_III!5,II!1).Gab1(Y657~P,PRS1_PRS2!6).Grb2(cSH3!6,SH2s!2).p52Shc1(Y317~P!2,PTB!4).EGFR(I_III!3,II!1,Y992~P!4).EGF(EGFL!3)    Gab1kp102

403 HRG(EGFL!5).ErbB4(I_III!5,II!1).Gab1(Y447~O,PRS1_PRS2!6).Grb2(cSH3!6,SH2s!2).p52Shc1(Y317~P!2,PTB!4).EGFR(I_III!3,II!1,Y1086~P!4).EGF(EGFL!3) -> HRG(EGFL!5).ErbB4(I_III!5,II!1).Gab1(Y447~P,PRS1_PRS2!6).Grb2(cSH3!6,SH2s!2).p52Shc1(Y317~P!2,PTB!4).EGFR(I_III!3,II!1,Y1086~P!4).EGF(EGFL!3)    Gab1kp103
404 HRG(EGFL!5).ErbB4(I_III!5,II!1).Gab1(Y472~O,PRS1_PRS2!6).Grb2(cSH3!6,SH2s!2).p52Shc1(Y317~P!2,PTB!4).EGFR(I_III!3,II!1,Y1086~P!4).EGF(EGFL!3) -> HRG(EGFL!5).ErbB4(I_III!5,II!1).Gab1(Y472~P,PRS1_PRS2!6).Grb2(cSH3!6,SH2s!2).p52Shc1(Y317~P!2,PTB!4).EGFR(I_III!3,II!1,Y1086~P!4).EGF(EGFL!3)    Gab1kp104
405 HRG(EGFL!5).ErbB4(I_III!5,II!1).Gab1(Y619~O,PRS1_PRS2!6).Grb2(cSH3!6,SH2s!2).p52Shc1(Y317~P!2,PTB!4).EGFR(I_III!3,II!1,Y1086~P!4).EGF(EGFL!3) -> HRG(EGFL!5).ErbB4(I_III!5,II!1).Gab1(Y619~P,PRS1_PRS2!6).Grb2(cSH3!6,SH2s!2).p52Shc1(Y317~P!2,PTB!4).EGFR(I_III!3,II!1,Y1086~P!4).EGF(EGFL!3)    Gab1kp105
406 HRG(EGFL!5).ErbB4(I_III!5,II!1).Gab1(Y657~O,PRS1_PRS2!6).Grb2(cSH3!6,SH2s!2).p52Shc1(Y317~P!2,PTB!4).EGFR(I_III!3,II!1,Y1086~P!4).EGF(EGFL!3) -> HRG(EGFL!5).ErbB4(I_III!5,II!1).Gab1(Y657~P,PRS1_PRS2!6).Grb2(cSH3!6,SH2s!2).p52Shc1(Y317~P!2,PTB!4).EGFR(I_III!3,II!1,Y1086~P!4).EGF(EGFL!3)    Gab1kp106

407 HRG(EGFL!5).ErbB4(I_III!5,II!1).Gab1(Y447~O,PRS1_PRS2!6).Grb2(cSH3!6,SH2s!2).p52Shc1(Y317~P!2,PTB!4).EGFR(I_III!3,II!1,Y1114~P!4).EGF(EGFL!3) -> HRG(EGFL!5).ErbB4(I_III!5,II!1).Gab1(Y447~P,PRS1_PRS2!6).Grb2(cSH3!6,SH2s!2).p52Shc1(Y317~P!2,PTB!4).EGFR(I_III!3,II!1,Y1114~P!4).EGF(EGFL!3)    Gab1kp107
408 HRG(EGFL!5).ErbB4(I_III!5,II!1).Gab1(Y472~O,PRS1_PRS2!6).Grb2(cSH3!6,SH2s!2).p52Shc1(Y317~P!2,PTB!4).EGFR(I_III!3,II!1,Y1114~P!4).EGF(EGFL!3) -> HRG(EGFL!5).ErbB4(I_III!5,II!1).Gab1(Y472~P,PRS1_PRS2!6).Grb2(cSH3!6,SH2s!2).p52Shc1(Y317~P!2,PTB!4).EGFR(I_III!3,II!1,Y1114~P!4).EGF(EGFL!3)    Gab1kp108
409 HRG(EGFL!5).ErbB4(I_III!5,II!1).Gab1(Y619~O,PRS1_PRS2!6).Grb2(cSH3!6,SH2s!2).p52Shc1(Y317~P!2,PTB!4).EGFR(I_III!3,II!1,Y1114~P!4).EGF(EGFL!3) -> HRG(EGFL!5).ErbB4(I_III!5,II!1).Gab1(Y619~P,PRS1_PRS2!6).Grb2(cSH3!6,SH2s!2).p52Shc1(Y317~P!2,PTB!4).EGFR(I_III!3,II!1,Y1114~P!4).EGF(EGFL!3)    Gab1kp109
410 HRG(EGFL!5).ErbB4(I_III!5,II!1).Gab1(Y657~O,PRS1_PRS2!6).Grb2(cSH3!6,SH2s!2).p52Shc1(Y317~P!2,PTB!4).EGFR(I_III!3,II!1,Y1114~P!4).EGF(EGFL!3) -> HRG(EGFL!5).ErbB4(I_III!5,II!1).Gab1(Y657~P,PRS1_PRS2!6).Grb2(cSH3!6,SH2s!2).p52Shc1(Y317~P!2,PTB!4).EGFR(I_III!3,II!1,Y1114~P!4).EGF(EGFL!3)    Gab1kp110

# ErbB4-EGFR where ErbB4 transphosphorylates Gab1-Grb2
411 HRG(EGFL!5).ErbB4(I_III!5,II!1).Gab1(Y447~O,PRS1_PRS2!6).Grb2(cSH3!6,SH2r!4).EGFR(I_III!3,II!1,Y1068~P!4).EGF(EGFL!3) -> HRG(EGFL!5).ErbB4(I_III!5,II!1).Gab1(Y447~P,PRS1_PRS2!6).Grb2(cSH3!6,SH2r!4).EGFR(I_III!3,II!1,Y1068~P!4).EGF(EGFL!3)    Gab1kp111
412 HRG(EGFL!5).ErbB4(I_III!5,II!1).Gab1(Y472~O,PRS1_PRS2!6).Grb2(cSH3!6,SH2r!4).EGFR(I_III!3,II!1,Y1068~P!4).EGF(EGFL!3) -> HRG(EGFL!5).ErbB4(I_III!5,II!1).Gab1(Y472~P,PRS1_PRS2!6).Grb2(cSH3!6,SH2r!4).EGFR(I_III!3,II!1,Y1068~P!4).EGF(EGFL!3)    Gab1kp112
413 HRG(EGFL!5).ErbB4(I_III!5,II!1).Gab1(Y619~O,PRS1_PRS2!6).Grb2(cSH3!6,SH2r!4).EGFR(I_III!3,II!1,Y1068~P!4).EGF(EGFL!3) -> HRG(EGFL!5).ErbB4(I_III!5,II!1).Gab1(Y619~P,PRS1_PRS2!6).Grb2(cSH3!6,SH2r!4).EGFR(I_III!3,II!1,Y1068~P!4).EGF(EGFL!3)    Gab1kp113
414 HRG(EGFL!5).ErbB4(I_III!5,II!1).Gab1(Y657~O,PRS1_PRS2!6).Grb2(cSH3!6,SH2r!4).EGFR(I_III!3,II!1,Y1068~P!4).EGF(EGFL!3) -> HRG(EGFL!5).ErbB4(I_III!5,II!1).Gab1(Y657~P,PRS1_PRS2!6).Grb2(cSH3!6,SH2r!4).EGFR(I_III!3,II!1,Y1068~P!4).EGF(EGFL!3)    Gab1kp114

415 HRG(EGFL!5).ErbB4(I_III!5,II!1).Gab1(Y447~O,PRS1_PRS2!6).Grb2(cSH3!6,SH2r!4).EGFR(I_III!3,II!1,Y1114~P!4).EGF(EGFL!3) -> HRG(EGFL!5).ErbB4(I_III!5,II!1).Gab1(Y447~P,PRS1_PRS2!6).Grb2(cSH3!6,SH2r!4).EGFR(I_III!3,II!1,Y1114~P!4).EGF(EGFL!3)    Gab1kp115
416 HRG(EGFL!5).ErbB4(I_III!5,II!1).Gab1(Y472~O,PRS1_PRS2!6).Grb2(cSH3!6,SH2r!4).EGFR(I_III!3,II!1,Y1114~P!4).EGF(EGFL!3) -> HRG(EGFL!5).ErbB4(I_III!5,II!1).Gab1(Y472~P,PRS1_PRS2!6).Grb2(cSH3!6,SH2r!4).EGFR(I_III!3,II!1,Y1114~P!4).EGF(EGFL!3)    Gab1kp116
417 HRG(EGFL!5).ErbB4(I_III!5,II!1).Gab1(Y619~O,PRS1_PRS2!6).Grb2(cSH3!6,SH2r!4).EGFR(I_III!3,II!1,Y1114~P!4).EGF(EGFL!3) -> HRG(EGFL!5).ErbB4(I_III!5,II!1).Gab1(Y619~P,PRS1_PRS2!6).Grb2(cSH3!6,SH2r!4).EGFR(I_III!3,II!1,Y1114~P!4).EGF(EGFL!3)    Gab1kp117
418 HRG(EGFL!5).ErbB4(I_III!5,II!1).Gab1(Y657~O,PRS1_PRS2!6).Grb2(cSH3!6,SH2r!4).EGFR(I_III!3,II!1,Y1114~P!4).EGF(EGFL!3) -> HRG(EGFL!5).ErbB4(I_III!5,II!1).Gab1(Y657~P,PRS1_PRS2!6).Grb2(cSH3!6,SH2r!4).EGFR(I_III!3,II!1,Y1114~P!4).EGF(EGFL!3)    Gab1kp118

419 HRG(EGFL!5).ErbB4(I_III!5,II!1).Gab1(Y447~O,PRS1_PRS2!6).Grb2(cSH3!6,SH2r!4).EGFR(I_III!3,II!1,Y1148~P!4).EGF(EGFL!3) -> HRG(EGFL!5).ErbB4(I_III!5,II!1).Gab1(Y447~P,PRS1_PRS2!6).Grb2(cSH3!6,SH2r!4).EGFR(I_III!3,II!1,Y1148~P!4).EGF(EGFL!3)    Gab1kp119
420 HRG(EGFL!5).ErbB4(I_III!5,II!1).Gab1(Y472~O,PRS1_PRS2!6).Grb2(cSH3!6,SH2r!4).EGFR(I_III!3,II!1,Y1148~P!4).EGF(EGFL!3) -> HRG(EGFL!5).ErbB4(I_III!5,II!1).Gab1(Y472~P,PRS1_PRS2!6).Grb2(cSH3!6,SH2r!4).EGFR(I_III!3,II!1,Y1148~P!4).EGF(EGFL!3)    Gab1kp120
421 HRG(EGFL!5).ErbB4(I_III!5,II!1).Gab1(Y619~O,PRS1_PRS2!6).Grb2(cSH3!6,SH2r!4).EGFR(I_III!3,II!1,Y1148~P!4).EGF(EGFL!3) -> HRG(EGFL!5).ErbB4(I_III!5,II!1).Gab1(Y619~P,PRS1_PRS2!6).Grb2(cSH3!6,SH2r!4).EGFR(I_III!3,II!1,Y1148~P!4).EGF(EGFL!3)    Gab1kp121
422 HRG(EGFL!5).ErbB4(I_III!5,II!1).Gab1(Y657~O,PRS1_PRS2!6).Grb2(cSH3!6,SH2r!4).EGFR(I_III!3,II!1,Y1148~P!4).EGF(EGFL!3) -> HRG(EGFL!5).ErbB4(I_III!5,II!1).Gab1(Y657~P,PRS1_PRS2!6).Grb2(cSH3!6,SH2r!4).EGFR(I_III!3,II!1,Y1148~P!4).EGF(EGFL!3)    Gab1kp122

423 HRG(EGFL!5).ErbB4(I_III!5,II!1).Gab1(Y447~O,PRS1_PRS2!6).Grb2(cSH3!6,SH2r!4).EGFR(I_III!3,II!1,Y1173~P!4).EGF(EGFL!3) -> HRG(EGFL!5).ErbB4(I_III!5,II!1).Gab1(Y447~P,PRS1_PRS2!6).Grb2(cSH3!6,SH2r!4).EGFR(I_III!3,II!1,Y1173~P!4).EGF(EGFL!3)    Gab1kp123
424 HRG(EGFL!5).ErbB4(I_III!5,II!1).Gab1(Y472~O,PRS1_PRS2!6).Grb2(cSH3!6,SH2r!4).EGFR(I_III!3,II!1,Y1173~P!4).EGF(EGFL!3) -> HRG(EGFL!5).ErbB4(I_III!5,II!1).Gab1(Y472~P,PRS1_PRS2!6).Grb2(cSH3!6,SH2r!4).EGFR(I_III!3,II!1,Y1173~P!4).EGF(EGFL!3)    Gab1kp124
425 HRG(EGFL!5).ErbB4(I_III!5,II!1).Gab1(Y619~O,PRS1_PRS2!6).Grb2(cSH3!6,SH2r!4).EGFR(I_III!3,II!1,Y1173~P!4).EGF(EGFL!3) -> HRG(EGFL!5).ErbB4(I_III!5,II!1).Gab1(Y619~P,PRS1_PRS2!6).Grb2(cSH3!6,SH2r!4).EGFR(I_III!3,II!1,Y1173~P!4).EGF(EGFL!3)    Gab1kp125
426 HRG(EGFL!5).ErbB4(I_III!5,II!1).Gab1(Y657~O,PRS1_PRS2!6).Grb2(cSH3!6,SH2r!4).EGFR(I_III!3,II!1,Y1173~P!4).EGF(EGFL!3) -> HRG(EGFL!5).ErbB4(I_III!5,II!1).Gab1(Y657~P,PRS1_PRS2!6).Grb2(cSH3!6,SH2r!4).EGFR(I_III!3,II!1,Y1173~P!4).EGF(EGFL!3)    Gab1kp126


# ErbB4-ErbB2 where ErbB4 transphosphorylates Gab1-Grb2-Shc
427 HRG(EGFL!5).ErbB4(I_III!5,II!1).Gab1(Y447~O,PRS1_PRS2!6).Grb2(cSH3!6,SH2s!2).p52Shc1(Y317~P!2,PTB!4).ErbB2(II!1,Y1196~P!4) -> HRG(EGFL!5).ErbB4(I_III!5,II!1).Gab1(Y447~P,PRS1_PRS2!6).Grb2(cSH3!6,SH2s!2).p52Shc1(Y317~P!2,PTB!4).ErbB2(II!1,Y1196~P!4)    Gab1kp127
428 HRG(EGFL!5).ErbB4(I_III!5,II!1).Gab1(Y472~O,PRS1_PRS2!6).Grb2(cSH3!6,SH2s!2).p52Shc1(Y317~P!2,PTB!4).ErbB2(II!1,Y1196~P!4) -> HRG(EGFL!5).ErbB4(I_III!5,II!1).Gab1(Y472~P,PRS1_PRS2!6).Grb2(cSH3!6,SH2s!2).p52Shc1(Y317~P!2,PTB!4).ErbB2(II!1,Y1196~P!4)    Gab1kp128
429 HRG(EGFL!5).ErbB4(I_III!5,II!1).Gab1(Y619~O,PRS1_PRS2!6).Grb2(cSH3!6,SH2s!2).p52Shc1(Y317~P!2,PTB!4).ErbB2(II!1,Y1196~P!4) -> HRG(EGFL!5).ErbB4(I_III!5,II!1).Gab1(Y619~P,PRS1_PRS2!6).Grb2(cSH3!6,SH2s!2).p52Shc1(Y317~P!2,PTB!4).ErbB2(II!1,Y1196~P!4)    Gab1kp129
430 HRG(EGFL!5).ErbB4(I_III!5,II!1).Gab1(Y657~O,PRS1_PRS2!6).Grb2(cSH3!6,SH2s!2).p52Shc1(Y317~P!2,PTB!4).ErbB2(II!1,Y1196~P!4) -> HRG(EGFL!5).ErbB4(I_III!5,II!1).Gab1(Y657~P,PRS1_PRS2!6).Grb2(cSH3!6,SH2s!2).p52Shc1(Y317~P!2,PTB!4).ErbB2(II!1,Y1196~P!4)    Gab1kp130

431 HRG(EGFL!5).ErbB4(I_III!5,II!1).Gab1(Y447~O,PRS1_PRS2!6).Grb2(cSH3!6,SH2s!2).p52Shc1(Y317~P!2,PTB!4).ErbB2(II!1,Y1222~P!4) -> HRG(EGFL!5).ErbB4(I_III!5,II!1).Gab1(Y447~P,PRS1_PRS2!6).Grb2(cSH3!6,SH2s!2).p52Shc1(Y317~P!2,PTB!4).ErbB2(II!1,Y1222~P!4)    Gab1kp131
432 HRG(EGFL!5).ErbB4(I_III!5,II!1).Gab1(Y472~O,PRS1_PRS2!6).Grb2(cSH3!6,SH2s!2).p52Shc1(Y317~P!2,PTB!4).ErbB2(II!1,Y1222~P!4) -> HRG(EGFL!5).ErbB4(I_III!5,II!1).Gab1(Y472~P,PRS1_PRS2!6).Grb2(cSH3!6,SH2s!2).p52Shc1(Y317~P!2,PTB!4).ErbB2(II!1,Y1222~P!4)    Gab1kp132
433 HRG(EGFL!5).ErbB4(I_III!5,II!1).Gab1(Y619~O,PRS1_PRS2!6).Grb2(cSH3!6,SH2s!2).p52Shc1(Y317~P!2,PTB!4).ErbB2(II!1,Y1222~P!4) -> HRG(EGFL!5).ErbB4(I_III!5,II!1).Gab1(Y619~P,PRS1_PRS2!6).Grb2(cSH3!6,SH2s!2).p52Shc1(Y317~P!2,PTB!4).ErbB2(II!1,Y1222~P!4)    Gab1kp133
434 HRG(EGFL!5).ErbB4(I_III!5,II!1).Gab1(Y657~O,PRS1_PRS2!6).Grb2(cSH3!6,SH2s!2).p52Shc1(Y317~P!2,PTB!4).ErbB2(II!1,Y1222~P!4) -> HRG(EGFL!5).ErbB4(I_III!5,II!1).Gab1(Y657~P,PRS1_PRS2!6).Grb2(cSH3!6,SH2s!2).p52Shc1(Y317~P!2,PTB!4).ErbB2(II!1,Y1222~P!4)    Gab1kp134

435 HRG(EGFL!5).ErbB4(I_III!5,II!1).Gab1(Y447~O,PRS1_PRS2!6).Grb2(cSH3!6,SH2s!2).p52Shc1(Y317~P!2,PTB!4).ErbB2(II!1,Y1248~P!4) -> HRG(EGFL!5).ErbB4(I_III!5,II!1).Gab1(Y447~P,PRS1_PRS2!6).Grb2(cSH3!6,SH2s!2).p52Shc1(Y317~P!2,PTB!4).ErbB2(II!1,Y1248~P!4)    Gab1kp135
436 HRG(EGFL!5).ErbB4(I_III!5,II!1).Gab1(Y472~O,PRS1_PRS2!6).Grb2(cSH3!6,SH2s!2).p52Shc1(Y317~P!2,PTB!4).ErbB2(II!1,Y1248~P!4) -> HRG(EGFL!5).ErbB4(I_III!5,II!1).Gab1(Y472~P,PRS1_PRS2!6).Grb2(cSH3!6,SH2s!2).p52Shc1(Y317~P!2,PTB!4).ErbB2(II!1,Y1248~P!4)    Gab1kp136
437 HRG(EGFL!5).ErbB4(I_III!5,II!1).Gab1(Y619~O,PRS1_PRS2!6).Grb2(cSH3!6,SH2s!2).p52Shc1(Y317~P!2,PTB!4).ErbB2(II!1,Y1248~P!4) -> HRG(EGFL!5).ErbB4(I_III!5,II!1).Gab1(Y619~P,PRS1_PRS2!6).Grb2(cSH3!6,SH2s!2).p52Shc1(Y317~P!2,PTB!4).ErbB2(II!1,Y1248~P!4)    Gab1kp137
438 HRG(EGFL!5).ErbB4(I_III!5,II!1).Gab1(Y657~O,PRS1_PRS2!6).Grb2(cSH3!6,SH2s!2).p52Shc1(Y317~P!2,PTB!4).ErbB2(II!1,Y1248~P!4) -> HRG(EGFL!5).ErbB4(I_III!5,II!1).Gab1(Y657~P,PRS1_PRS2!6).Grb2(cSH3!6,SH2s!2).p52Shc1(Y317~P!2,PTB!4).ErbB2(II!1,Y1248~P!4)    Gab1kp138

# ErbB4-ErbB2 where ErbB4 transphosphorylates Gab1-Grb2
439 HRG(EGFL!5).ErbB4(I_III!5,II!1).Gab1(Y447~O,PRS1_PRS2!6).Grb2(cSH3!6,SH2r!4).ErbB2(II!1,Y1139~P!4) -> HRG(EGFL!5).ErbB4(I_III!5,II!1).Gab1(Y447~P,PRS1_PRS2!6).Grb2(cSH3!6,SH2r!4).ErbB2(II!1,Y1139~P!4)    Gab1kp139
440 HRG(EGFL!5).ErbB4(I_III!5,II!1).Gab1(Y472~O,PRS1_PRS2!6).Grb2(cSH3!6,SH2r!4).ErbB2(II!1,Y1139~P!4) -> HRG(EGFL!5).ErbB4(I_III!5,II!1).Gab1(Y472~P,PRS1_PRS2!6).Grb2(cSH3!6,SH2r!4).ErbB2(II!1,Y1139~P!4)    Gab1kp140
441 HRG(EGFL!5).ErbB4(I_III!5,II!1).Gab1(Y619~O,PRS1_PRS2!6).Grb2(cSH3!6,SH2r!4).ErbB2(II!1,Y1139~P!4) -> HRG(EGFL!5).ErbB4(I_III!5,II!1).Gab1(Y619~P,PRS1_PRS2!6).Grb2(cSH3!6,SH2r!4).ErbB2(II!1,Y1139~P!4)    Gab1kp141
442 HRG(EGFL!5).ErbB4(I_III!5,II!1).Gab1(Y657~O,PRS1_PRS2!6).Grb2(cSH3!6,SH2r!4).ErbB2(II!1,Y1139~P!4) -> HRG(EGFL!5).ErbB4(I_III!5,II!1).Gab1(Y657~P,PRS1_PRS2!6).Grb2(cSH3!6,SH2r!4).ErbB2(II!1,Y1139~P!4)    Gab1kp142

# ErbB4-ErbB3 where ErbB4 transphosphorylates Gab1-Grb2-Shc
443 HRG(EGFL!5).ErbB4(I_III!5,II!1).Gab1(Y447~O,PRS1_PRS2!6).Grb2(cSH3!6,SH2s!2).p52Shc1(Y317~P!2,PTB!4).ErbB3(I_III!3,II!1,Y1328~P!4).HRG(EGFL!3) -> HRG(EGFL!5).ErbB4(I_III!5,II!1).Gab1(Y447~P,PRS1_PRS2!6).Grb2(cSH3!6,SH2s!2).p52Shc1(Y317~P!2,PTB!4).ErbB3(I_III!3,II!1,Y1328~P!4).HRG(EGFL!3)    Gab1kp143
444 HRG(EGFL!5).ErbB4(I_III!5,II!1).Gab1(Y472~O,PRS1_PRS2!6).Grb2(cSH3!6,SH2s!2).p52Shc1(Y317~P!2,PTB!4).ErbB3(I_III!3,II!1,Y1328~P!4).HRG(EGFL!3) -> HRG(EGFL!5).ErbB4(I_III!5,II!1).Gab1(Y472~P,PRS1_PRS2!6).Grb2(cSH3!6,SH2s!2).p52Shc1(Y317~P!2,PTB!4).ErbB3(I_III!3,II!1,Y1328~P!4).HRG(EGFL!3)	Gab1kp144
445 HRG(EGFL!5).ErbB4(I_III!5,II!1).Gab1(Y619~O,PRS1_PRS2!6).Grb2(cSH3!6,SH2s!2).p52Shc1(Y317~P!2,PTB!4).ErbB3(I_III!3,II!1,Y1328~P!4).HRG(EGFL!3) -> HRG(EGFL!5).ErbB4(I_III!5,II!1).Gab1(Y619~P,PRS1_PRS2!6).Grb2(cSH3!6,SH2s!2).p52Shc1(Y317~P!2,PTB!4).ErbB3(I_III!3,II!1,Y1328~P!4).HRG(EGFL!3)    Gab1kp145
446 HRG(EGFL!5).ErbB4(I_III!5,II!1).Gab1(Y657~O,PRS1_PRS2!6).Grb2(cSH3!6,SH2s!2).p52Shc1(Y317~P!2,PTB!4).ErbB3(I_III!3,II!1,Y1328~P!4).HRG(EGFL!3) -> HRG(EGFL!5).ErbB4(I_III!5,II!1).Gab1(Y657~P,PRS1_PRS2!6).Grb2(cSH3!6,SH2s!2).p52Shc1(Y317~P!2,PTB!4).ErbB3(I_III!3,II!1,Y1328~P!4).HRG(EGFL!3)    Gab1kp146

# ErbB4-ErbB4 where ErbB4 transphosphorylates Gab1-Grb2-Shc
447 HRG(EGFL!5).ErbB4(I_III!5,II!1).Gab1(Y447~O,PRS1_PRS2!6).Grb2(cSH3!6,SH2s!2).p52Shc1(Y317~P!2,PTB!4).ErbB4(I_III!3,II!1,Y1188~P!4).HRG(EGFL!3) -> HRG(EGFL!5).ErbB4(I_III!5,II!1).Gab1(Y447~P,PRS1_PRS2!6).Grb2(cSH3!6,SH2s!2).p52Shc1(Y317~P!2,PTB!4).ErbB4(I_III!3,II!1,Y1188~P!4).HRG(EGFL!3)    Gab1kp147
448 HRG(EGFL!5).ErbB4(I_III!5,II!1).Gab1(Y472~O,PRS1_PRS2!6).Grb2(cSH3!6,SH2s!2).p52Shc1(Y317~P!2,PTB!4).ErbB4(I_III!3,II!1,Y1188~P!4).HRG(EGFL!3) -> HRG(EGFL!5).ErbB4(I_III!5,II!1).Gab1(Y472~P,PRS1_PRS2!6).Grb2(cSH3!6,SH2s!2).p52Shc1(Y317~P!2,PTB!4).ErbB4(I_III!3,II!1,Y1188~P!4).HRG(EGFL!3)	Gab1kp148
449 HRG(EGFL!5).ErbB4(I_III!5,II!1).Gab1(Y619~O,PRS1_PRS2!6).Grb2(cSH3!6,SH2s!2).p52Shc1(Y317~P!2,PTB!4).ErbB4(I_III!3,II!1,Y1188~P!4).HRG(EGFL!3) -> HRG(EGFL!5).ErbB4(I_III!5,II!1).Gab1(Y619~P,PRS1_PRS2!6).Grb2(cSH3!6,SH2s!2).p52Shc1(Y317~P!2,PTB!4).ErbB4(I_III!3,II!1,Y1188~P!4).HRG(EGFL!3)    Gab1kp149
450 HRG(EGFL!5).ErbB4(I_III!5,II!1).Gab1(Y657~O,PRS1_PRS2!6).Grb2(cSH3!6,SH2s!2).p52Shc1(Y317~P!2,PTB!4).ErbB4(I_III!3,II!1,Y1188~P!4).HRG(EGFL!3) -> HRG(EGFL!5).ErbB4(I_III!5,II!1).Gab1(Y657~P,PRS1_PRS2!6).Grb2(cSH3!6,SH2s!2).p52Shc1(Y317~P!2,PTB!4).ErbB4(I_III!3,II!1,Y1188~P!4).HRG(EGFL!3)    Gab1kp150

451 HRG(EGFL!5).ErbB4(I_III!5,II!1).Gab1(Y447~O,PRS1_PRS2!6).Grb2(cSH3!6,SH2s!2).p52Shc1(Y317~P!2,PTB!4).ErbB4(I_III!3,II!1,Y1242~P!4).HRG(EGFL!3) -> HRG(EGFL!5).ErbB4(I_III!5,II!1).Gab1(Y447~P,PRS1_PRS2!6).Grb2(cSH3!6,SH2s!2).p52Shc1(Y317~P!2,PTB!4).ErbB4(I_III!3,II!1,Y1242~P!4).HRG(EGFL!3)    Gab1kp151
452 HRG(EGFL!5).ErbB4(I_III!5,II!1).Gab1(Y472~O,PRS1_PRS2!6).Grb2(cSH3!6,SH2s!2).p52Shc1(Y317~P!2,PTB!4).ErbB4(I_III!3,II!1,Y1242~P!4).HRG(EGFL!3) -> HRG(EGFL!5).ErbB4(I_III!5,II!1).Gab1(Y472~P,PRS1_PRS2!6).Grb2(cSH3!6,SH2s!2).p52Shc1(Y317~P!2,PTB!4).ErbB4(I_III!3,II!1,Y1242~P!4).HRG(EGFL!3)	Gab1kp152
453 HRG(EGFL!5).ErbB4(I_III!5,II!1).Gab1(Y619~O,PRS1_PRS2!6).Grb2(cSH3!6,SH2s!2).p52Shc1(Y317~P!2,PTB!4).ErbB4(I_III!3,II!1,Y1242~P!4).HRG(EGFL!3) -> HRG(EGFL!5).ErbB4(I_III!5,II!1).Gab1(Y619~P,PRS1_PRS2!6).Grb2(cSH3!6,SH2s!2).p52Shc1(Y317~P!2,PTB!4).ErbB4(I_III!3,II!1,Y1242~P!4).HRG(EGFL!3)    Gab1kp153
454 HRG(EGFL!5).ErbB4(I_III!5,II!1).Gab1(Y657~O,PRS1_PRS2!6).Grb2(cSH3!6,SH2s!2).p52Shc1(Y317~P!2,PTB!4).ErbB4(I_III!3,II!1,Y1242~P!4).HRG(EGFL!3) -> HRG(EGFL!5).ErbB4(I_III!5,II!1).Gab1(Y657~P,PRS1_PRS2!6).Grb2(cSH3!6,SH2s!2).p52Shc1(Y317~P!2,PTB!4).ErbB4(I_III!3,II!1,Y1242~P!4).HRG(EGFL!3)    Gab1kp154


#Gab1 Dissociation
455 Gab1(PRS1_PRS2!1).Grb2(cSH3!1) -> Gab1(PRS1_PRS2) + Grb2(cSH3)	Gab1kp155


#Phospho Erk and Gab1 interaction
#  ERK2 binds with Gab1
456 ERK2(STkinase,T185~P,Y187~P) + Gab1(T312~O) -> ERK2(STkinase!1,T185~P,Y187~P).Gab1(T312~O!1) Gab1kp156
457 ERK2(STkinase,T185~P,Y187~P) + Gab1(S381~O) -> ERK2(STkinase!1,T185~P,Y187~P).Gab1(S381~O!1) Gab1kp157
458 ERK2(STkinase,T185~P,Y187~P) + Gab1(S454~O) -> ERK2(STkinase!1,T185~P,Y187~P).Gab1(S454~O!1) Gab1kp158
459 ERK2(STkinase,T185~P,Y187~P) + Gab1(T476~O) -> ERK2(STkinase!1,T185~P,Y187~P).Gab1(T476~O!1) Gab1kp159
460 ERK2(STkinase,T185~P,Y187~P) + Gab1(S581~O) -> ERK2(STkinase!1,T185~P,Y187~P).Gab1(S581~O!1) Gab1kp160
461 ERK2(STkinase,T185~P,Y187~P) + Gab1(S597~O) -> ERK2(STkinase!1,T185~P,Y187~P).Gab1(S597~O!1) Gab1kp161

#ERK2 phosphorylates Gab1
462 ERK2(STkinase!1,T185~P,Y187~P).Gab1(T312~O!1) -> ERK2(STkinase!1,T185~P,Y187~P).Gab1(T312~P!1)    Gab1kp162
463 ERK2(STkinase!1,T185~P,Y187~P).Gab1(S381~O!1) -> ERK2(STkinase!1,T185~P,Y187~P).Gab1(S381~P!1)    Gab1kp163
464 ERK2(STkinase!1,T185~P,Y187~P).Gab1(S454~O!1) -> ERK2(STkinase!1,T185~P,Y187~P).Gab1(S454~P!1)    Gab1kp164
465 ERK2(STkinase!1,T185~P,Y187~P).Gab1(T476~O!1) -> ERK2(STkinase!1,T185~P,Y187~P).Gab1(T476~P!1)    Gab1kp165
466 ERK2(STkinase!1,T185~P,Y187~P).Gab1(S581~O!1) -> ERK2(STkinase!1,T185~P,Y187~P).Gab1(S581~P!1)    Gab1kp166
467 ERK2(STkinase!1,T185~P,Y187~P).Gab1(S597~O!1) -> ERK2(STkinase!1,T185~P,Y187~P).Gab1(S597~P!1)    Gab1kp167

#Dissociation of ERK2 and phospho Gab1
468 ERK2(STkinase!1).Gab1(T312!1) -> ERK2(STkinase) + Gab1(T312)	Gab1kp168
469 ERK2(STkinase!1).Gab1(S381!1) -> ERK2(STkinase) + Gab1(S381)	Gab1kp168
470 ERK2(STkinase!1).Gab1(S454!1) -> ERK2(STkinase) + Gab1(S454)	Gab1kp168
471 ERK2(STkinase!1).Gab1(T476!1) -> ERK2(STkinase) + Gab1(T476)	Gab1kp168
472 ERK2(STkinase!1).Gab1(S581!1) -> ERK2(STkinase) + Gab1(S581)	Gab1kp168
473 ERK2(STkinase!1).Gab1(S597!1) -> ERK2(STkinase) + Gab1(S597)	Gab1kp168

#Intrinsic dephosphorylation of receptor phosphorylated Gab1 sites
474 Gab1(Y447~P) -> Gab1(Y447~O)	Gab1kp169
475 Gab1(Y472~P) -> Gab1(Y472~O)	Gab1kp169
476 Gab1(Y619~P) -> Gab1(Y619~O)	Gab1kp169
477 Gab1(Y657~P) -> Gab1(Y657~O)	Gab1kp169

#Intrinsic dephosphorylation of Erk2 phosphorylated Gab1 sites
478 Gab1(T312~P) -> Gab1(T312~O)	Gab1kp170
479 Gab1(S381~P) -> Gab1(S381~O)	Gab1kp170
480 Gab1(S454~P) -> Gab1(S454~O)	Gab1kp170
481 Gab1(T476~P) -> Gab1(T476~O)	Gab1kp170
482 Gab1(S581~P) -> Gab1(S581~O)	Gab1kp170
483 Gab1(S597~P) -> Gab1(S597~O)	Gab1kp170


#Gab1 binding to PIP3
484 Gab1(PH,PRS1_PRS2,S581~P) + PIP3(C3P,two~F) -> Gab1(PH!1,PRS1_PRS2,S581~P).PIP3(C3P!1,two~F)	Gab1kp171
485 Gab1(PH!1).PIP3(C3P!1) -> Gab1(PH) + PIP3(C3P) Gab1kp172

############## PI3K interactions ##############
# PI3K binds membrane localized Gab1-Grb2-Shc and Gab1-Grb2 at Y447,Y472, and Y619
# Y447
486 PI3K(G_p85_nSH2_cSH2,R_p85_nSH2_cSH2) + Gab1(Y447~P,T312~O!?,S381~O!?,S454~O!?,T476~O!?,S581~O!?,S597~O!?,PRS1_PRS2!1).Grb2(cSH3!1,SH2s!2).p52Shc1(Y317~O!2,PTB!+) -> PI3K(G_p85_nSH2_cSH2!3,R_p85_nSH2_cSH2).Gab1(Y447~P!3,T312~O!?,S381~O!?,S454~O!?,T476~O!?,S581~O!?,S597~O!?,PRS1_PRS2!1).Grb2(cSH3!1,SH2s!2).p52Shc1(Y317~O!2,PTB!+)     PI3Kkp1
487 PI3K(G_p85_nSH2_cSH2,R_p85_nSH2_cSH2) + Gab1(Y447~P,T312~O!?,S381~O!?,S454~O!?,T476~O!?,S581~O!?,S597~O!?,PRS1_PRS2!1).Grb2(cSH3!1,SH2r!+) -> PI3K(G_p85_nSH2_cSH2!2,R_p85_nSH2_cSH2).Gab1(Y447~P!2,T312~O!?,S381~O!?,S454~O!?,T476~O!?,S581~O!?,S597~O!?,PRS1_PRS2!1).Grb2(cSH3!1,SH2r!+)       PI3Kkp1

# Y472
488 PI3K(G_p85_nSH2_cSH2,R_p85_nSH2_cSH2) + Gab1(Y472~P,T312~O!?,S381~O!?,S454~O!?,T476~O!?,S581~O!?,S597~O!?,PRS1_PRS2!1).Grb2(cSH3!1,SH2s!2).p52Shc1(Y317~O!2,PTB!+) -> PI3K(G_p85_nSH2_cSH2!3,R_p85_nSH2_cSH2).Gab1(Y472~P!3,T312~O!?,S381~O!?,S454~O!?,T476~O!?,S581~O!?,S597~O!?,PRS1_PRS2!1).Grb2(cSH3!1,SH2s!2).p52Shc1(Y317~O!2,PTB!+)     PI3Kkp2
489 PI3K(G_p85_nSH2_cSH2,R_p85_nSH2_cSH2) + Gab1(Y472~P,T312~O!?,S381~O!?,S454~O!?,T476~O!?,S581~O!?,S597~O!?,PRS1_PRS2!1).Grb2(cSH3!1,SH2r!+) -> PI3K(G_p85_nSH2_cSH2!2,R_p85_nSH2_cSH2).Gab1(Y472~P!2,T312~O!?,S381~O!?,S454~O!?,T476~O!?,S581~O!?,S597~O!?,PRS1_PRS2!1).Grb2(cSH3!1,SH2r!+)       PI3Kkp2

# Y619
490 PI3K(G_p85_nSH2_cSH2,R_p85_nSH2_cSH2) + Gab1(Y619~P,T312~O!?,S381~O!?,S454~O!?,T476~O!?,S581~O!?,S597~O!?,PRS1_PRS2!1).Grb2(cSH3!1,SH2s!2).p52Shc1(Y317~O!2,PTB!+) -> PI3K(G_p85_nSH2_cSH2!3,R_p85_nSH2_cSH2).Gab1(Y619~P!3,T312~O!?,S381~O!?,S454~O!?,T476~O!?,S581~O!?,S597~O!?,PRS1_PRS2!1).Grb2(cSH3!1,SH2s!2).p52Shc1(Y317~O!2,PTB!+)     PI3Kkp3
491 PI3K(G_p85_nSH2_cSH2,R_p85_nSH2_cSH2) + Gab1(Y619~P,T312~O!?,S381~O!?,S454~O!?,T476~O!?,S581~O!?,S597~O!?,PRS1_PRS2!1).Grb2(cSH3!1,SH2r!+) -> PI3K(G_p85_nSH2_cSH2!2,R_p85_nSH2_cSH2).Gab1(Y619~P!2,T312~O!?,S381~O!?,S454~O!?,T476~O!?,S581~O!?,S597~O!?,PRS1_PRS2!1).Grb2(cSH3!1,SH2r!+)       PI3Kkp3

#PI3K binds membrane localized Gab1-PIP3
492 PI3K(G_p85_nSH2_cSH2,R_p85_nSH2_cSH2) + Gab1(PH!1,Y447~P,T312~O!?,S381~O!?,S454~O!?,T476~O!?,S581~O!?,S597~O!?).PIP3(C3P!1) -> PI3K(G_p85_nSH2_cSH2!2,R_p85_nSH2_cSH2).Gab1(PH!1,T312~O!?,S381~O!?,S454~O!?,T476~O!?,S581~O!?,S597~O!?,Y447~P!2).PIP3(C3P!1)	PI3Kkp1
493 PI3K(G_p85_nSH2_cSH2,R_p85_nSH2_cSH2) + Gab1(PH!1,Y472~P,T312~O!?,S381~O!?,S454~O!?,T476~O!?,S581~O!?,S597~O!?).PIP3(C3P!1) -> PI3K(G_p85_nSH2_cSH2!2,R_p85_nSH2_cSH2).Gab1(PH!1,T312~O!?,S381~O!?,S454~O!?,T476~O!?,S581~O!?,S597~O!?,Y472~P!2).PIP3(C3P!1)	PI3Kkp2
494 PI3K(G_p85_nSH2_cSH2,R_p85_nSH2_cSH2) + Gab1(PH!1,Y619~P,T312~O!?,S381~O!?,S454~O!?,T476~O!?,S581~O!?,S597~O!?).PIP3(C3P!1) -> PI3K(G_p85_nSH2_cSH2!2,R_p85_nSH2_cSH2).Gab1(PH!1,T312~O!?,S381~O!?,S454~O!?,T476~O!?,S581~O!?,S597~O!?,Y619~P!2).PIP3(C3P!1)	PI3Kkp3

# PI3K dissasociation from Gab1
495 PI3K(G_p85_nSH2_cSH2!1).Gab1(Y447!1) -> PI3K(G_p85_nSH2_cSH2) + Gab1(Y447) 	PI3Kkp4
496 PI3K(G_p85_nSH2_cSH2!1).Gab1(Y472!1) -> PI3K(G_p85_nSH2_cSH2) + Gab1(Y472) 	PI3Kkp4
497 PI3K(G_p85_nSH2_cSH2!1).Gab1(Y619!1) -> PI3K(G_p85_nSH2_cSH2) + Gab1(Y619) 	PI3Kkp4

#membrane bound PI3K Binding to active Ras
498 PI3K(p110_RBD,G_p85_nSH2_cSH2!3).Gab1(Y447~P!3,PRS1_PRS2!1).Grb2(cSH3!1,SH2s!2).p52Shc1(Y317~P!2,PTB!+) + KRas(g~GTP,GTPase) -> PI3K(p110_RBD!4,G_p85_nSH2_cSH2!3).Gab1(Y447~P!3,PRS1_PRS2!1).Grb2(cSH3!1,SH2s!2).p52Shc1(Y317~P!2,PTB!+).KRas(g~GTP,GTPase!4) 	PI3Kkp5
499 PI3K(p110_RBD,G_p85_nSH2_cSH2!2).Gab1(Y447~P!2,PRS1_PRS2!1).Grb2(cSH3!1,SH2r!+) + KRas(g~GTP,GTPase) -> PI3K(G_p85_nSH2_cSH2!2,p110_RBD!4).Gab1(Y447~P!2,PRS1_PRS2!1).Grb2(cSH3!1,SH2r!+).KRas(g~GTP,GTPase!4)	PI3Kkp5

500 PI3K(p110_RBD,G_p85_nSH2_cSH2!3).Gab1(Y472~P!3,PRS1_PRS2!1).Grb2(cSH3!1,SH2s!2).p52Shc1(Y317~P!2,PTB!+) + KRas(g~GTP,GTPase) -> PI3K(p110_RBD!4,G_p85_nSH2_cSH2!3).Gab1(Y472~P!3,PRS1_PRS2!1).Grb2(cSH3!1,SH2s!2).p52Shc1(Y317~P!2,PTB!+).KRas(g~GTP,GTPase!4) 	PI3Kkp5
501 PI3K(p110_RBD,G_p85_nSH2_cSH2!2).Gab1(Y472~P!2,PRS1_PRS2!1).Grb2(cSH3!1,SH2r!+) + KRas(g~GTP,GTPase) -> PI3K(G_p85_nSH2_cSH2!2,p110_RBD!4).Gab1(Y472~P!2,PRS1_PRS2!1).Grb2(cSH3!1,SH2r!+).KRas(g~GTP,GTPase!4)	PI3Kkp5

502 PI3K(p110_RBD,G_p85_nSH2_cSH2!3).Gab1(Y619~P!3,PRS1_PRS2!1).Grb2(cSH3!1,SH2s!2).p52Shc1(Y317~P!2,PTB!+) + KRas(g~GTP,GTPase) -> PI3K(p110_RBD!4,G_p85_nSH2_cSH2!3).Gab1(Y619~P!3,PRS1_PRS2!1).Grb2(cSH3!1,SH2s!2).p52Shc1(Y317~P!2,PTB!+).KRas(g~GTP,GTPase!4) 	PI3Kkp5
503 PI3K(p110_RBD,G_p85_nSH2_cSH2!2).Gab1(Y619~P!2,PRS1_PRS2!1).Grb2(cSH3!1,SH2r!+) + KRas(g~GTP,GTPase) -> PI3K(G_p85_nSH2_cSH2!2,p110_RBD!4).Gab1(Y619~P!2,PRS1_PRS2!1).Grb2(cSH3!1,SH2r!+).KRas(g~GTP,GTPase!4)	PI3Kkp5


#PI3K Dissociation from Ras
504 PI3K(p110_RBD!1).KRas(GTPase!1) -> PI3K(p110_RBD) + KRas(GTPase)	PI3Kkp6


#PI3K binds to ErbB3
505 PI3K(R_p85_nSH2_cSH2,G_p85_nSH2_cSH2) + ErbB3(Y1054~P) <-> PI3K(R_p85_nSH2_cSH2!1,G_p85_nSH2_cSH2).ErbB3(Y1054~P!1)	PI3Kkp7,PI3Kkm7
506 PI3K(R_p85_nSH2_cSH2,G_p85_nSH2_cSH2) + ErbB3(Y1197~P) <-> PI3K(R_p85_nSH2_cSH2!1,G_p85_nSH2_cSH2).ErbB3(Y1197~P!1)	PI3Kkp8,PI3Kkm8
507 PI3K(R_p85_nSH2_cSH2,G_p85_nSH2_cSH2) + ErbB3(Y1222~P) <-> PI3K(R_p85_nSH2_cSH2!1,G_p85_nSH2_cSH2).ErbB3(Y1222~P!1)	PI3Kkp9,PI3Kkm9
508 PI3K(R_p85_nSH2_cSH2,G_p85_nSH2_cSH2) + ErbB3(Y1260~P) <-> PI3K(R_p85_nSH2_cSH2!1,G_p85_nSH2_cSH2).ErbB3(Y1260~P!1)	PI3Kkp10,PI3Kkm10
509 PI3K(R_p85_nSH2_cSH2,G_p85_nSH2_cSH2) + ErbB3(Y1276~P) <-> PI3K(R_p85_nSH2_cSH2!1,G_p85_nSH2_cSH2).ErbB3(Y1276~P!1)	PI3Kkp11,PI3Kkm11
510 PI3K(R_p85_nSH2_cSH2,G_p85_nSH2_cSH2) + ErbB3(Y1289~P) <-> PI3K(R_p85_nSH2_cSH2!1,G_p85_nSH2_cSH2).ErbB3(Y1289~P!1)	PI3Kkp12,PI3Kkm12

#PI3K binds to ErbB4
511 PI3K(R_p85_nSH2_cSH2,G_p85_nSH2_cSH2) + ErbB4(Y1056~P) <-> PI3K(R_p85_nSH2_cSH2!1,G_p85_nSH2_cSH2).ErbB4(Y1056~P!1)	PI3Kkp13,PI3Kkm13

############## PIP3 interactions ##############

#creation of PIP3 by active PI3K-Gab1~rec

512 PI3K(G_p85_nSH2_cSH2!3).Gab1(Y447~P!3,PRS1_PRS2!1,PH).Grb2(cSH3!1,SH2s!2).p52Shc1(Y317~P!2,PTB!+) -> PIP3(C3P,two~F,loc~M) + PI3K(G_p85_nSH2_cSH2!3).Gab1(Y447~P!3,PRS1_PRS2!1,PH).Grb2(cSH3!1,SH2s!2).p52Shc1(Y317~P!2,PTB!+) 	PIP3kp1
513 PI3K(G_p85_nSH2_cSH2!3).Gab1(Y472~P!3,PRS1_PRS2!1,PH).Grb2(cSH3!1,SH2s!2).p52Shc1(Y317~P!2,PTB!+) -> PIP3(C3P,two~F,loc~M) + PI3K(G_p85_nSH2_cSH2!3).Gab1(Y472~P!3,PRS1_PRS2!1,PH).Grb2(cSH3!1,SH2s!2).p52Shc1(Y317~P!2,PTB!+)	PIP3kp1
514 PI3K(G_p85_nSH2_cSH2!3).Gab1(Y619~P!3,PRS1_PRS2!1,PH).Grb2(cSH3!1,SH2s!2).p52Shc1(Y317~P!2,PTB!+) -> PIP3(C3P,two~F,loc~M) + PI3K(G_p85_nSH2_cSH2!3).Gab1(Y619~P!3,PRS1_PRS2!1,PH).Grb2(cSH3!1,SH2s!2).p52Shc1(Y317~P!2,PTB!+)	PIP3kp1

515 PI3K(G_p85_nSH2_cSH2!3).Gab1(Y447~P!3,PRS1_PRS2!1,PH).Grb2(cSH3!1,SH2r!+) -> PIP3(C3P,two~F,loc~M) + PI3K(G_p85_nSH2_cSH2!3).Gab1(Y447~P!3,PRS1_PRS2!1,PH).Grb2(cSH3!1,SH2r!+)	PIP3kp1
516 PI3K(G_p85_nSH2_cSH2!3).Gab1(Y472~P!3,PRS1_PRS2!1,PH).Grb2(cSH3!1,SH2r!+) -> PIP3(C3P,two~F,loc~M) + PI3K(G_p85_nSH2_cSH2!3).Gab1(Y472~P!3,PRS1_PRS2!1,PH).Grb2(cSH3!1,SH2r!+)	PIP3kp1
517 PI3K(G_p85_nSH2_cSH2!3).Gab1(Y619~P!3,PRS1_PRS2!1,PH).Grb2(cSH3!1,SH2r!+) -> PIP3(C3P,two~F,loc~M) + PI3K(G_p85_nSH2_cSH2!3).Gab1(Y619~P!3,PRS1_PRS2!1,PH).Grb2(cSH3!1,SH2r!+)	PIP3kp1

#With PI3K bound to Ras
518 PI3K(G_p85_nSH2_cSH2!3,p110_RBD!+).Gab1(Y447~P!3,PRS1_PRS2!1).Grb2(cSH3!1,SH2s!2).p52Shc1(Y317~P!2,PTB!+) -> PIP3(C3P,two~F,loc~M) + PI3K(G_p85_nSH2_cSH2!3,p110_RBD!+).Gab1(Y447~P!3,PRS1_PRS2!1).Grb2(cSH3!1,SH2s!2).p52Shc1(Y317~P!2,PTB!+)	PIP3kp1_5
519 PI3K(G_p85_nSH2_cSH2!3,p110_RBD!+).Gab1(Y472~P!3,PRS1_PRS2!1).Grb2(cSH3!1,SH2s!2).p52Shc1(Y317~P!2,PTB!+) -> PIP3(C3P,two~F,loc~M) + PI3K(G_p85_nSH2_cSH2!3,p110_RBD!+).Gab1(Y472~P!3,PRS1_PRS2!1).Grb2(cSH3!1,SH2s!2).p52Shc1(Y317~P!2,PTB!+)	PIP3kp1_5
520 PI3K(G_p85_nSH2_cSH2!3,p110_RBD!+).Gab1(Y619~P!3,PRS1_PRS2!1).Grb2(cSH3!1,SH2s!2).p52Shc1(Y317~P!2,PTB!+) -> PIP3(C3P,two~F,loc~M) + PI3K(G_p85_nSH2_cSH2!3,p110_RBD!+).Gab1(Y619~P!3,PRS1_PRS2!1).Grb2(cSH3!1,SH2s!2).p52Shc1(Y317~P!2,PTB!+)	PIP3kp1_5

521 PI3K(G_p85_nSH2_cSH2!3,p110_RBD!+).Gab1(Y447~P!3,PRS1_PRS2!1).Grb2(cSH3!1,SH2r!+) -> PIP3(C3P,two~F,loc~M) + PI3K(G_p85_nSH2_cSH2!3,p110_RBD!+).Gab1(Y447~P!3,PRS1_PRS2!1).Grb2(cSH3!1,SH2r!+)	PIP3kp1_5
522 PI3K(G_p85_nSH2_cSH2!3,p110_RBD!+).Gab1(Y472~P!3,PRS1_PRS2!1).Grb2(cSH3!1,SH2r!+) -> PIP3(C3P,two~F,loc~M) + PI3K(G_p85_nSH2_cSH2!3,p110_RBD!+).Gab1(Y472~P!3,PRS1_PRS2!1).Grb2(cSH3!1,SH2r!+)	PIP3kp1_5
523 PI3K(G_p85_nSH2_cSH2!3,p110_RBD!+).Gab1(Y619~P!3,PRS1_PRS2!1).Grb2(cSH3!1,SH2r!+) -> PIP3(C3P,two~F,loc~M) + PI3K(G_p85_nSH2_cSH2!3,p110_RBD!+).Gab1(Y619~P!3,PRS1_PRS2!1).Grb2(cSH3!1,SH2r!+)	PIP3kp1_5

#creation of PIP3 by PI3k-Gab1-PIP3
524 PI3K(G_p85_nSH2_cSH2!1).Gab1(Y447~P!1,PH!+) -> PIP3(C3P,two~F,loc~M) + PI3K(G_p85_nSH2_cSH2!1).Gab1(Y447~P!1,PH!+)	PIP3kp1
525 PI3K(G_p85_nSH2_cSH2!1).Gab1(Y472~P!1,PH!+) -> PIP3(C3P,two~F,loc~M) + PI3K(G_p85_nSH2_cSH2!1).Gab1(Y472~P!1,PH!+)	PIP3kp1
526 PI3K(G_p85_nSH2_cSH2!1).Gab1(Y619~P!1,PH!+) -> PIP3(C3P,two~F,loc~M) + PI3K(G_p85_nSH2_cSH2!1).Gab1(Y619~P!1,PH!+)	PIP3kp1

#creation of PIP3 by PI3K-ErbB3
527 PI3K(R_p85_nSH2_cSH2!+) -> PIP3(C3P,two~F,loc~M) + PI3K(R_p85_nSH2_cSH2!+)	PIP3kp1

#PIP3 dephosphorylation
528 PIP3(C3P,two~F) -> PIP3(C3P,two~T) PIP3kp3

############## AKT and CDK1 interactions ##############

#Akt1 binding to PIP3
529 Akt1(PH) + PIP3(C3P,two~F) <-> Akt1(PH!1).PIP3(C3P!1,two~F)         Akt1kp1,Akt1km1

#PDK1 binding to PIP3
530 PDK1(PH) + PIP3(C3P,two~F) <-> PDK1(PH!1).PIP3(C3P!1,two~F)		Akt1kp2,Akt1km2

#PDK1-PIP3 binds to AKT-PIP3
531 PDK1(PH!+,STkinase) + Akt1(PH!+,T308~O) -> PDK1(PH!+,STkinase!1).Akt1(PH!+,T308~O!1)		Akt1kp3

#phosphorylation of AKT-PIP3 by CDK1-PIP3
532 PDK1(PH!+,STkinase!1).Akt1(PH!+,T308~O!1) -> PDK1(PH!+,STkinase!1).Akt1(PH!+,T308~P!1)		Akt1kp4

533 PDK1(STkinase!1).Akt1(T308!1) -> PDK1(STkinase) + Akt1(T308)	Akt1kp5

# Phosphorylation of S473 by an unknown kinase, this only occurs when T308 is phosphorylated
534 Akt1(T308~P!?,S473~O) -> Akt1(T308~P!?,S473~P)		Akt1kp6

# Activated Akt-1 phosphorylates Raf-1 at S259
# This is an inhibitory feedback loop from the AKT pathway to the ERK pathway
535 Akt1(T308~P!?,S473~P,STkinase) + Raf1(S259~O) -> Akt1(T308~P!?,S473~P,STkinase!1).Raf1(S259~O!1)	Akt1kp7
536 Akt1(T308~P!?,S473~P,STkinase!1).Raf1(S259~O!1) -> Akt1(T308~P!?,S473~P,STkinase!1).Raf1(S259~P!1)	Akt1kp8
537 Akt1(STkinase!1).Raf1(S259!1) -> Akt1(STkinase) + Raf1(S259)	Akt1kp9

# Intrinsic dephosphorylation of phosphorylated residues on AKT
538 Raf1(S259~P) -> Raf1(S259~O)	Akt1kp10

539 Akt1(T308~P) -> Akt1(T308~O)		Akt1kp11

540 Akt1(S473~P) -> Akt1(S473~O)		Akt1kp12

############## p120RasGAP interactions ##############
541 p120RasGAP(nSH2) + EGFR(Y992~P) <-> p120RasGAP(nSH2!1).EGFR(Y992~P!1)	rGAPkp1,rGAPkm1
542 p120RasGAP(nSH2!+,GAP) + KRas(GTPase,g~GTP) -> p120RasGAP(nSH2!+,GAP!1).KRas(GTPase!1,g~GTP)	rGAPkp2
543 p120RasGAP(GAP!1).KRas(GTPase!1,g~GTP) -> p120RasGAP(GAP!1).KRas(GTPase!1,g~GDP)	rGAPkp3
544 p120RasGAP(GAP!1).KRas(GTPase!1) -> p120RasGAP(GAP) + KRas(GTPase)	rGAPkp4

end reaction rules

#ACTIONS

writeXML();
}}}
```

```
See ''Gab1 (Grb2-associated binder 1)'' in [[Proteins]]
!BNGL
{{{Gab1(PH,PRS1_PRS2,T312~O~P,S381~O~P,Y447~O~P,S454~O~P,Y472~O~P,\
T476~O~P,S551~O~P,S597~O~P,Y627~O~P,Y659~O~P,loc~C)}}}
!Summary
Gab1 (represented by {{{Gab1}}} in the model) is an adapter protein that plays an important role in activation of ~PI3K.  Phosphotyrosines in Gab1 (Y447, Y472 and Y589) serve as docking sites for the regulatory subunit of ~PI3K (p85&alpha;).  The tyrosine residues Y447, Y472 and Y589 are substrates of EGFR and are each found in a ~YxxM motif, a motif recognized by ~SH2 domains. Gab1 interacts indirectly with ~ErbB family members via Grb2. The Grb2 binding sites in Gab1 are proline-rich sequences (~PRS1 and ~PRS2), which are recognized by the C-terminal ~SH3 domain of Grb2. Gab1 contains a PH domain, which interacts with ~PIP3 only when S552 in Gab1 is phosphorylated.  The residue T476 represents a substrate of ~ERK2; phosphorylation of T476 enhances p85 interaction with Gab1 via the pY472 docking site.  The Grb2-binding sites in Gab1 were mapped by Lock et al. (2000) and Lewitzky et al. (2001). The ERK substrate in Gab1 was discovered by Yu et al. (2001). For the role of Y447, Y472 and Y589 and Gab1 in ~EGFR-mediated activation of ~PI3K signaling, see Mattoon et al. (2004).
!DOG
[img[DOGS/Gab1.jpg]]
!Reading
*[[OMIM ID 604439 | http://www.ncbi.nlm.nih.gov/omim/604439 ]]
*Lewitzky M, Kardinal C, Gehring NH, Schmidt EK, Konkol B, Eulitz M, Birchmeier W, Schaeper U, Feller SM (2001) The C-terminal ~SH3 domain of the adapter protein Grb2 binds with high affinity to sequences in Gab1 and ~SLP-76 which lack the ~SH3-typical ~P-x-x-P core motif. Oncogene 20:1052-1062. [[PMID: 11314042 | http://www.ncbi.nlm.nih.gov/pubmed/11314042]]
*Liu Y, Rohrschneider LR (2002) The gift of Gab. FEBS Lett 515:1-7. [[PMID: 11943184 | http://www.ncbi.nlm.nih.gov/pubmed/11943184]]
*Lock LS, Royal I, Naujokas MA, Park M (2000) Identification of an atypical Grb2 carboxyl-terminal ~SH3 domain binding site in Gab docking proteins reveals Grb2-dependent and -independent recruitment of Gab1 to receptor tyrosine kinases. J Biol Chem 275:31536-31545. [[PMID: 10913131 | http://www.ncbi.nlm.nih.gov/pubmed/10913131]]
*Mattoon DR, Lamothe B, Lax I, Schlessinger J (2004) The docking protein ~GabI is the primary mediator of ~EGF-stimulated activation of the ~PI-3K/Akt cell survival pathway. BMC Biology 2:24. [[PMID: 15550174 | http://www.ncbi.nlm.nih.gov/pubmed/15550174]]
*Yu CF, Roshan B, Liu ZX, Cantley LG (2001) ERK regulates the hepatocyte growth factor-mediated interaction of Gab1 and the phosphatidylinositol 3-kinase. J Biol Chem 276:32552-32558. [[PMID: 11445578 | http://www.ncbi.nlm.nih.gov/pubmed/11445578]]
```

```
''Gab1 reversibly binds Grb2'' (see Arrow 16 in [[Contact Map]])
{{{Gab1(PRS1_PRS2)+Grb2(cSH3)<->Gab1(PRS1_PRS2!1).Grb2(cSH3!1)}}}
!Rules
{{{
301 Gab1(PRS1_PRS2) + Grb2(cSH3,SH2s!2).p52Shc1(Y317~P!2,PTB!+) -> \
 Gab1(PRS1_PRS2!1).Grb2(cSH3!1,SH2s!2).p52Shc1(Y317~P!2,PTB!+)     Gab1kp1

302 Gab1(PRS1_PRS2) + Grb2(cSH3,SH2r!+) -> \
 Gab1(PRS1_PRS2!1).Grb2(cSH3!1,SH2r!+)       Gab1kp1

455 Gab1(PRS1_PRS2!1).Grb2(cSH3!1) -> \
 Gab1(PRS1_PRS2) + Grb2(cSH3)	Gab1kp155
}}}
```

```
''~PI3K reversibly binds Gab1'' (see Arrow 21 in [[Contact Map]])
{{{PI3K(G_p85_nSH2_cSH2)+Gab1(Y447~P)<->PI3K(G_p85_nSH2_cSH2!1).Gab1(Y447~P!1)}}}
{{{PI3K(G_p85_nSH2_cSH2)+Gab1(Y472~P)<->PI3K(G_p85_nSH2_cSH2!1).Gab1(Y472~P!1)}}}
{{{PI3K(G_p85_nSH2_cSH2)+Gab1(Y589~P)<->PI3K(G_p85_nSH2_cSH2!1).Gab1(Y589~P!1)}}}
!Rules
{{{
486 PI3K(G_p85_nSH2_cSH2,R_p85_nSH2_cSH2) + Gab1(Y447~P,T312~O!?,S381~O!?,S454~O!?,T476~O!?,S581~O!?,S597~O!?,PRS1_PRS2!1).Grb2(cSH3!1,SH2s!2).p52Shc1(Y317~O!2,PTB!+) -> \
 PI3K(G_p85_nSH2_cSH2!3,R_p85_nSH2_cSH2).Gab1(Y447~P!3,T312~O!?,S381~O!?,S454~O!?,T476~O!?,S581~O!?,S597~O!?,PRS1_PRS2!1).Grb2(cSH3!1,SH2s!2).p52Shc1(Y317~O!2,PTB!+)     PI3Kkp1

487 PI3K(G_p85_nSH2_cSH2,R_p85_nSH2_cSH2) + Gab1(Y447~P,T312~O!?,S381~O!?,S454~O!?,T476~O!?,S581~O!?,S597~O!?,PRS1_PRS2!1).Grb2(cSH3!1,SH2r!+) -> \
 PI3K(G_p85_nSH2_cSH2!2,R_p85_nSH2_cSH2).Gab1(Y447~P!2,T312~O!?,S381~O!?,S454~O!?,T476~O!?,S581~O!?,S597~O!?,PRS1_PRS2!1).Grb2(cSH3!1,SH2r!+)       PI3Kkp1

488 PI3K(G_p85_nSH2_cSH2,R_p85_nSH2_cSH2) + Gab1(Y472~P,T312~O!?,S381~O!?,S454~O!?,T476~O!?,S581~O!?,S597~O!?,PRS1_PRS2!1).Grb2(cSH3!1,SH2s!2).p52Shc1(Y317~O!2,PTB!+) -> \
 PI3K(G_p85_nSH2_cSH2!3,R_p85_nSH2_cSH2).Gab1(Y472~P!3,T312~O!?,S381~O!?,S454~O!?,T476~O!?,S581~O!?,S597~O!?,PRS1_PRS2!1).Grb2(cSH3!1,SH2s!2).p52Shc1(Y317~O!2,PTB!+)     PI3Kkp2

489 PI3K(G_p85_nSH2_cSH2,R_p85_nSH2_cSH2) + Gab1(Y472~P,T312~O!?,S381~O!?,S454~O!?,T476~O!?,S581~O!?,S597~O!?,PRS1_PRS2!1).Grb2(cSH3!1,SH2r!+) -> \
 PI3K(G_p85_nSH2_cSH2!2,R_p85_nSH2_cSH2).Gab1(Y472~P!2,T312~O!?,S381~O!?,S454~O!?,T476~O!?,S581~O!?,S597~O!?,PRS1_PRS2!1).Grb2(cSH3!1,SH2r!+)       PI3Kkp2

490 PI3K(G_p85_nSH2_cSH2,R_p85_nSH2_cSH2) + Gab1(Y619~P,T312~O!?,S381~O!?,S454~O!?,T476~O!?,S581~O!?,S597~O!?,PRS1_PRS2!1).Grb2(cSH3!1,SH2s!2).p52Shc1(Y317~O!2,PTB!+) -> \
 PI3K(G_p85_nSH2_cSH2!3,R_p85_nSH2_cSH2).Gab1(Y619~P!3,T312~O!?,S381~O!?,S454~O!?,T476~O!?,S581~O!?,S597~O!?,PRS1_PRS2!1).Grb2(cSH3!1,SH2s!2).p52Shc1(Y317~O!2,PTB!+)     PI3Kkp3

491 PI3K(G_p85_nSH2_cSH2,R_p85_nSH2_cSH2) + Gab1(Y619~P,T312~O!?,S381~O!?,S454~O!?,T476~O!?,S581~O!?,S597~O!?,PRS1_PRS2!1).Grb2(cSH3!1,SH2r!+) -> \
 PI3K(G_p85_nSH2_cSH2!2,R_p85_nSH2_cSH2).Gab1(Y619~P!2,T312~O!?,S381~O!?,S454~O!?,T476~O!?,S581~O!?,S597~O!?,PRS1_PRS2!1).Grb2(cSH3!1,SH2r!+)       PI3Kkp3

492 PI3K(G_p85_nSH2_cSH2,R_p85_nSH2_cSH2) + Gab1(PH!1,Y447~P,T312~O!?,S381~O!?,S454~O!?,T476~O!?,S581~O!?,S597~O!?).PIP3(C3P!1) -> \
 PI3K(G_p85_nSH2_cSH2!2,R_p85_nSH2_cSH2).Gab1(PH!1,T312~O!?,S381~O!?,S454~O!?,T476~O!?,S581~O!?,S597~O!?,Y447~P!2).PIP3(C3P!1)	PI3Kkp1

493 PI3K(G_p85_nSH2_cSH2,R_p85_nSH2_cSH2) + Gab1(PH!1,Y472~P,T312~O!?,S381~O!?,S454~O!?,T476~O!?,S581~O!?,S597~O!?).PIP3(C3P!1) -> \
 PI3K(G_p85_nSH2_cSH2!2,R_p85_nSH2_cSH2).Gab1(PH!1,T312~O!?,S381~O!?,S454~O!?,T476~O!?,S581~O!?,S597~O!?,Y472~P!2).PIP3(C3P!1)	PI3Kkp2

494 PI3K(G_p85_nSH2_cSH2,R_p85_nSH2_cSH2) + Gab1(PH!1,Y619~P,T312~O!?,S381~O!?,S454~O!?,T476~O!?,S581~O!?,S597~O!?).PIP3(C3P!1) -> \
 PI3K(G_p85_nSH2_cSH2!2,R_p85_nSH2_cSH2).Gab1(PH!1,T312~O!?,S381~O!?,S454~O!?,T476~O!?,S581~O!?,S597~O!?,Y619~P!2).PIP3(C3P!1)	PI3Kkp3

495 PI3K(G_p85_nSH2_cSH2!1).Gab1(Y447!1) -> \
 PI3K(G_p85_nSH2_cSH2) + Gab1(Y447) 	PI3Kkp4

496 PI3K(G_p85_nSH2_cSH2!1).Gab1(Y472!1) -> \
 PI3K(G_p85_nSH2_cSH2) + Gab1(Y472) 	PI3Kkp4

497 PI3K(G_p85_nSH2_cSH2!1).Gab1(Y619!1) -> \
 PI3K(G_p85_nSH2_cSH2) + Gab1(Y619) 	PI3Kkp4
}}}
```

```
''Gab1 reversibly binds ~PIP3'' (see Arrow 24 in [[Contact Map]])
{{{Gab1(PH)+PIP3(C3P)<->Gab1(PH!1).PIP3(C3P!1)}}}
!Rules
{{{
484 Gab1(PH,PRS1_PRS2,S581~P) + PIP3(C3P,two~F) -> \
 Gab1(PH!1,PRS1_PRS2,S581~P).PIP3(C3P!1,two~F)	Gab1kp171

485 Gab1(PH!1).PIP3(C3P!1) -> \
 Gab1(PH) + PIP3(C3P) Gab1kp172
}}}
```

```
To get started with this blank [[TiddlyWiki]], you'll need to modify the following tiddlers:
* [[SiteTitle]] & [[SiteSubtitle]]: The title and subtitle of the site, as shown above (after saving, they will also appear in the browser title bar)
* [[MainMenu]]: The menu (usually on the left)
* [[DefaultTiddlers]]: Contains the names of the tiddlers that you want to appear when the TiddlyWiki is opened
You'll also need to enter your username for signing your edits: <<option txtUserName>>
```

```
See ''Grb2 (growth factor receptor-bound protein 2)'' in [[Proteins]]
!BNGL
{{{Grb2(SH2r,SH2s,cSH3,nSH3,loc~C)}}}
!Summary
Grb2 (represented by {{{Grb2}}} in the model) is an adapter protein that contains an N-terminal ~SH3 domain (represented by {{{nSH3}}} in the model), an ~SH2 domain, and a C-terminal ~SH3 domain (represented by {{{cSH3}}} in the model).  The ~SH2 domain is represented by {{{SH2r}}} and {{{SH2s}}} in the model.  It is convenient to introduce these two virtual components to represent the ~SH2 domain because it reduces the number of rules needed to capture the interactions of Grb2.  The virtual component {{{SH2r}}} is used to represent the ~SH2 domain bound to an ~ErbB family member.  The virtual component {{{SH2s}}} is used to represent the ~SH2 domain bound to Shc.
!DOG
[img[DOGS/Grb2.jpg]]
!Reading
*[[OMIM ID 108355 | http://www.ncbi.nlm.nih.gov/omim/108355]]
*Schlessinger J (1994) ~SH2/~SH3 signaling proteins. Curr Opin Genet Dev 4:25-30. [[PMID: 8193536 | http://www.ncbi.nlm.nih.gov/pubmed/8193536]]
```

```
''Grb2 reversibly binds Sos1'' (see Arrow 14 in [[Contact Map]])
{{{Grb2(nSH3)+Sos1(PRS)<->Grb2(nSH3!1).Sos1(PRS!1)}}}
!Rules
{{{
216 Sos1(PRS) + Grb2(nSH3,cSH3) <-> \
 Sos1(PRS!1).Grb2(nSH3!1,cSH3)	Scafoldkp2,Scafoldkm2

217 Sos1(PRS) + Grb2(nSH3,cSH3!2).Gab1(PRS1_PRS2!2) <-> \
 Sos1(PRS!1).Grb2(nSH3!1,cSH3!2).Gab1(PRS1_PRS2!2)   Scafoldkp3,Scafoldkm3
}}}
```

```
''Grb2 reversibly binds p52Shc1'' (see Arrow 15 in [[Contact Map]])
{{{Grb2(SH2)+p52Shc1(Y317~P)<->Grb2(SH2!1).p52Shc1(Y317~P!1)}}}
!Rules
{{{
215 Grb2(SH2r,SH2s) + p52Shc1(Y317~P) <-> \
 Grb2(SH2r,SH2s!1).p52Shc1(Y317~P!1)       Scafoldkp1,Scafoldkm1
}}}
```

```
See ''~HRG-&alpha; (neuregulin, isoform 1)'' in [[Proteins]]
!Definition of molecule type in BNGL
{{{HRG(EGFL,deg~F~T,loc~Ex~En)}}}
!Summary
~NRG1 isoform 1 or ~HRG-&alpha; (represented by {{{HRG}}} in the model) is a ligand of ~ErbB3.  It contains an ~EGF-like (EGFL) domain (see [[Domains]]), which is responsible for the interaction with ~ErbB3. There are many isoforms of ~NRG1, such as ~HRG-&beta;1 (isoform 6).  The ~UniProt canonical sequence is that of ~HRG-&alpha; (isoform 1).  In the model, HRG is found in one of two compartments: the extracellular (Ex) compartment or the endocytic (En) compartment.  The component {{{loc}}} is introduced to track the location of HRG.  The internal state of {{{loc}}} ({{{Ex}}} or {{{En}}}) indicates the location of HRG. In the model, HRG is internalized when it is bound to ~ErbB3 and degraded after it is internalized.  The component {{{deg}}} is introduced to track HRG degradation. The internal state of {{{deg}}} indicates whether HRG is intact ({{{F}}}) or degraded ({{{T}}}).
!DOG
[img[DOGS/HRG.jpg]]
!Reading
*[[OMIM ID 142445 | http://www.ncbi.nlm.nih.gov/omim/142445]]
*Nagata K (2010) Studies of the structure-activity relationships of peptides and proteins involved in growth and development based on their three-dimensional structures. Biosci Biotechnol Biochem 74:462-470. [[PMID: 20208353 | http://www.ncbi.nlm.nih.gov/pubmed/20208353]]
```

```
The model considers the direct binding interactions, catalytic activities, exchange activities, and transport and degradative processes listed in the table below.  These interactions/activities/processes are modeled by four classes of rules: 1) binding rules, 2) catalytic rules, 3) exchange rules, and 4) transport rules.  See the Rules tiddlers for listings of these rules.

|List of interactions/activities/processes considered in the model (numbers in the first column refer to arrows in the [[Contact Map]])|c
|Arrow |Type |Description |h
|1 |Binding |EGF reversibly binds EGFR |
|2 |Binding |~HRG-&alpha; reversibly binds ~ErbB3, ~ErbB4 |
|3 |Binding |EGFR homodimerizaton, EGFR reversibly binds ~ErbB2, ~ErbB3, ~ErbB4 |
|4 |Binding |~ErbB2 homodimerization, ~ErbB2 reversibly binds EGFR, ~ErbB3, ~ErbB4 |
|5 |Binding |~ErbB3 homodimerization, ~ErbB3 reversibly binds EGFR, ~ErbB2, ~ErbB4 |
|6 |Binding |~ErbB4 homodimerization, ~ErbB4 reversibly binds EGFR, ~ErbB2, ~ErbB3 |
|7 |Catalytic |EGFR catalyzes phosphorylation of EGFR, ~ErbB2, ~ErbB3 ~ErbB4, p52^^Shc1^^, and Gab1 |
|8 |Catalytic |~ErbB2 catalyzes phosphorylation of EGFR, ~ErbB3, ~ErbB4, p52^^Shc1^^, and Gab1 |
|9 |Catalytic |~ErbB4 catalyzes phosphorylation of EGFR, ~ErbB2, ~ErbB3, ~ErbB4, p52^^Shc1^^, and Gab1 |
|10 |Binding |p120^^~RasGAP^^ reversibly binds EGFR |
|11 |Binding |Grb2 reversibly binds EGFR and ~ErbB2 |
|12 |Binding |p52^^Shc1^^ reversibly binds ~ErbB receptors (EGFR, ~ErbB2, ~ErbB3, and ~ErbB4) |
|13 |Catalytic |~GTPase activity of ~K-Ras:p120^^~RasGAP^^ complex |
|14 |Binding |Grb2 reversibly binds Sos1 |
|15 |Binding |Grb2 reversibly binds p52^^Shc1^^ |
|16 |Binding |Grb2 reversibly binds Gab1 |
|17 |Exchange |Guanine nucleotide exchange activity of Sos1 |
|18 |Binding |~K-Ras reversibly binds Sos1 |
|19 |Binding |~K-Ras reversibly binds ~PI3K |
|20 |Binding |~K-Ras reversibly binds Raf-1, promoting phosphorylation of Raf-1 |
|21 |Binding |~PI3K reversibly binds ~ErbB3, ~ErbB4, Gab1 |
|22 |Catalytic |Raf-1 catalyzes phosphorylation of ~MEK1 |
|23 |Catalytic |~PI3K catalyzes phosphorylation of ~PIP2 to generate ~PIP3 |
|24 |Binding |Gab1 reversibly binds ~PIP3 |
|25 |Binding |~MEK1 reversibly binds ~ERK2 |
|26 |Catalytic |~ERK2 catalyzes phosphorylation of EGFR, Raf-1, ~MEK1, Gab1, and Sos1 |
|27 |Catalytic |~MEK1 catalyzes phosphorylation of ~ERK2 |
|28 |Catalytic |Akt1 catalyzes phosphorylation of Raf-1 |
|29 |Binding |Akt1 reversibly binds ~PIP3 |
|30 |Binding |~PDK1 reversibly binds ~PIP3 |
|31 |Catalytic |~PDK1 catalyzes phosphorylation of Akt1, enabling phosphorylation of S473 in Akt1 |
|- |Transport |Endocytosis, degradation of ligand (EGF and ~HRG-&alpha;) via endocytic pathway, and ~ErbB receptor recycling |

The four collapsable tables below provide additional information about receptor-proximal interactions, activities, and processes.

+++[<strong>Table 1.</strong> Ligand-receptor binding and receptor dimerization]
|Interactions and processes considered in the model that involve ligand-receptor binding and receptor dimerization|c
|Description |Arrow |h
|EGF reversibly binds EGFR |1 |
|~HRG-&alpha; reversibly binds ~ErbB3 |2 |
|~HRG-&alpha; reversibly binds ~ErbB4 |2 |
|Reversible formation of EGFR homodimers |3 |
|Reversible formation of ~EGFR-ErbB2 heterodimers |4 |
|Reversible formation of ~EGFR-ErbB3 heterodimers |5 |
|Reversible formation of ~EGFR-ErbB4 heterodimers |6 |
|Reversible formation of ~ErbB2 homodimers |4 |
|Reversible formation of ~ErbB2-ErbB3 heterodimers |5 |
|Reversible formation of ~ErbB2-ErbB4 heterodimers |6 |
|Reversible formation of ~ErbB3 homodimers |5 |
|Reversible formation of ~ErbB3-ErbB4 heterodimers |6 |
|Reversible formation of ~ErbB4 homodimers |6 |
===

+++[<strong>Table 2.</strong> Receptor trafficking]
|Interactions and processes considered in the model that involve endocytosis|c
|Description |h
|Internalization of dimeric EGFR |
|Internalization of dimeric ~ErbB2 |
|Internalization of dimeric ~ErbB3 |
|Internalization of dimeric ~ErbB4 |
|Internalization of bound EGF |
|Internalization of bound ~HRG-&alpha; |
|Release of EGF from endosomal EGFR|
|Release of ~HRG-&alpha; from endosomal ~ErbB3 |
|Release of ~HRG-&alpha; from endosomal ~ErbB4 |
|Lysosomal degradation of EGF |
|Lysosomal degradation of ~HRG-&alpha; |
|Dissociation of endosomal EGFR homodimers |
|Dissociation of endosomal ~EGFR-ErbB2 heterodimers  |
|Dissociation of endosomal ~EGFR-ErbB3 heterodimers  |
|Dissociation of endosomal ~EGFR-ErbB4 heterodimers  |
|Dissociation of endosomal ~ErbB2 homodimers |
|Dissociation of endosomal ~ErbB2-ErbB3 heterodimers  |
|Dissociation of endosomal ~ErbB2-ErbB4 heterodimers  |
|Dissociaton of endosomal ~ErbB3-ErbB3 homodimers |
|Dissociaton of endosomal ~ErbB3-ErbB4 heterodimers |
|Dissociaton of endosomal ~ErbB4-ErbB4 homodimers |
|Recycling of endosomal EGFR to the plasma membrane  |
|Recycling of endosomal ~ErbB2 to the plasma membrane  |
|Recycling of endosomal ~ErbB3 to the plasma membrane |
|Recycling of endosomal ~ErbB4 to the plasma membrane |
===

+++[<strong>Table 3.</strong> ErbB-catalyzed phosphorylation of Y sites in ErbB receptors]
|Interactions and processes considered in the model that involve ~ErbB-catalyzed phosphorylation of Y sites in ~ErbB receptors|c
|Description |Arrow(s) |h
|EGFR/~ErbB2/~ErbB4-mediated phosphorylation of Y992, Y1068, Y1086, Y1114, Y1148, and Y1173 in EGFR |7, 8, 9 |
|EGFR/~ErbB4-mediated phosphorylation of Y1139, Y1196, Y1222, and Y1248 in ~ErbB2 |7, 9 |
|EGFR/~ErbB2/~ErbB4-mediated phosphorylation of Y1054, Y1197, Y1222, Y1260, Y1289, and Y1328 in ~ErbB3 |7, 8, 9 |
|EGFR/~ErbB2/~ErbB4-mediated phosphorylation of Y1056, Y1188, and Y1242 in ~ErbB4 |7, 8, 9 |
===

+++[<strong>Table 4.</strong> Dephosphorylation of Y sites in ErbB receptors]
|Interactions and processes considered in the model that involve dephosphorylation of Y sites in ~ErbB receptors|c
|Description |h
|Dephosphorylation of Y992, Y1068, Y1086, Y1114, Y1148, and Y1173 in EGFR |
|Dephosphorylation of Y1139, Y1196, Y1222, and Y1248 in ~ErbB2 |
|Dephosphorylation of Y1054, Y1197, Y1222, Y1260, Y1276, Y1289, and Y1328 in ~ErbB3 |
|Dephosphorylation of Y1056, Y1188, and Y1242 in ~ErbB4 |
===
```

```
This document is a [[TiddlyWiki | http://www.tiddlywiki.com/]], which consists of tiddlers (pages that can be opened and closed).  It serves to annotate a rule-based model for ~ErbB-mediated activation of ERK and AKT.  In a rule-based model, graphs are used to represent molecules, and graph-rewriting rules are used to represent molecular interactions.  For a review of rule-based modeling, see [[Hlavacek et al. (2006) | http://stke.sciencemag.org/cgi/content/full/sigtrans;2006/344/re6?ijkey=g7njdDmsvuHUw&keytype=ref&siteid=sigtrans recent review]] [//Science's STKE// ''2006'':re6]. The model accounts for four members of the ~ErbB family of receptors (EGFR, ~ErbB2, ~ErbB3 and ~ErbB4) and two ~ErbB ligands: epidermal growth factor (EGF), which is a ligand of EGFR, and heregulin (aka neuregulin 1 or ~NRG1), which is a ligand of ~ErbB3. An overview/visualization of the model is provided by a [[contact map|Contact Map]].  In the contact map, boxes represent proteins, arrows represent binding and catalytic interactions, and flags represent post-translational modifications considered in the model.  Each arrow in the map represents a set of rules.  Each rule within a given set provides a formal representation of a common interaction in a distinct molecular context. Note that not all rules of the model are illustrated in the contact map. Tiddlers in the menu at left provide additional information about the model.  The Metadata tiddlers list the [[compartments|Compartments]], molecules ([[metabolites|Metabolites]] and [[proteins|Proteins]]), and molecular [[interactions|Interactions]] considered in the model, as well as the [[domains|Domains]], [[linear motifs|Linear Motifs]], and [[phosphorylation sites|Phosphorylation Sites]] of proteins considered in the model.  The Molecule Types tiddlers together annotate 18 molecule type graphs, which are key formal elements of the model specification.  The Rules tiddlers provide information about the 544 rules of the model and link the 31 arrows of the contact map to sets of rules.  There are rules for binding interactions, catalytic interactions, guanine nucleotide exchange, and receptor trafficking, which includes rules for endocytosis of receptors and receptor-bound ligands, ligand degradation in the endocytic compartment, and receptor recycling from the endocytic compartment to the plasma membrane compartment.  The menu at left also provides access to the [[full model specification|Full Model Specification]], including parameter values.  The model was specified using the ~BioNetGen language (BNGL).  For a detailed description of BNGL, see [[Faeder et al. (2009) | http://www.ccbb.pitt.edu/Faculty/Faeder/Publications/Reprints/Faeder_2009.pdf]] [//Methods Mol. Biol.// ''500'':113-167]. The full model must be simulated using a network-free method. [[NFsim | http://emonet.biology.yale.edu/nfsim/]] is a software tool that can be used to simulate the model.

The following people have contributed to the development and analysis of the model (alphabetical order):
*Meraj Aziz
*[[Michael E. Berens | http://www.tgen.org/research/index.cfm?pageid=77&peopleid=13]]
*James A. Cahill
*Matthew S. Creamer
*[[Haiyong Han | http://www.tgen.org/research/index.cfm?pageid=77&peopleid=67]]
*[[William S. Hlavacek | http://www.t6.lanl.gov/wish/]]
*[[Richard G. Posner | http://www.tgen.org/research/index.cfm?pageid=77&peopleid=687&view=profile]]
*Edward C. Stites
*Chin Wee Tan
*[[Daniel D. Von Hoff | http://www.tgen.org/research/index.cfm?pageid=77&peopleid=65]]

[[Tips]]
```

```
|The proteins listed in this table are ''not'' explicitly included in the model; however, each of these proteins is related to a protein included in the model. We assume that the different isoforms of a protein (e.g., ~MEK1 and ~MEK2) are indistinguishable.|c
|Protein Name |~UniProt ID |Gene Name |Comment |h
|~HRG-?1 |[[Q02297 | http://www.uniprot.org/uniprot/Q02297]] |//~NRG1//	|Growth factor, isoform 6 |
|Sos2 |[[Q07890 | http://www.uniprot.org/uniprot/Q07890]] |//~SOS2// |GEF |
|p85?	|[[O00459 | http://www.uniprot.org/uniprot/O00459]] |//~PIK3R2//	|Regulatory subunit of class IA ~PI3K |
|Akt2	|[[P31751 | http://www.uniprot.org/uniprot/P31751]] |//~AKT2//	|S/T kinase |
|Akt3	|[[Q9Y243 | http://www.uniprot.org/uniprot/Q9Y243]] |//~AKT3//	|S/T kinase |
|~H-Ras	|[[P01112 | http://www.uniprot.org/uniprot/P01112]] |//HRAS//	|~GTPase |
|~N-Ras	|[[P01111 | http://www.uniprot.org/uniprot/P01111]] |//NRAS//	|~GTPase|
|~A-Raf |[[P10398 | http://www.uniprot.org/uniprot/P10398]] |//ARAF// |MAPKKK |
|~B-Raf	|[[P15056 | http://www.uniprot.org/uniprot/P15056]] |//BRAF//	|MAPKKK|
|~MEK2	|[[P36507 | http://www.uniprot.org/uniprot/P36507]] |//~MAP2K2//	|MAPKK, dual-specificity kinase (Y and S/T substrates)|
|~ERK1	|[[P27361 | http://www.uniprot.org/uniprot/P27361]] |//~MAPK3//	|MAPK|
```

```
See ''~K-Ras'' in [[Proteins]]
!BNGL
{{{KRas(GTPase,g~GDP~GTP,loc~M)}}}
!Summary
~K-Ras (represented by {{{KRas}}} in the model) is a small ~GTPase switch.  The Ras family of proteins is composed of ~H-Ras, ~N-Ras and ~K-Ras, which is commonly mutated in cancer cells.  We take ~K-Ras to be representative of the Ras family members.
!DOG
[img[DOGS/KRas.jpg]]
!Reading
*[[OMIM ID 190070 | http://www.ncbi.nlm.nih.gov/omim/190070]]
*Downward J (2003) Targeting Ras signalling pathways in cancer therapy. Nat Rev Cancer 3:11-22. [[PMID: 12509763 | http://www.ncbi.nlm.nih.gov/pubmed/12509763]]
```

```
''~PI3K reversibly binds ~KRas'' (see Arrow 19 in [[Contact Map]])
{{{PI3K(p110_RBD)+KRas(GTPase)<->PI3K(p110_RBD!1).KRas(GTPase!1)}}}
!Rules
{{{
498 PI3K(p110_RBD,G_p85_nSH2_cSH2!3).Gab1(Y447~P!3,PRS1_PRS2!1).Grb2(cSH3!1,SH2s!2).p52Shc1(Y317~P!2,PTB!+) + KRas(g~GTP,GTPase) -> \
 PI3K(p110_RBD!4,G_p85_nSH2_cSH2!3).Gab1(Y447~P!3,PRS1_PRS2!1).Grb2(cSH3!1,SH2s!2).p52Shc1(Y317~P!2,PTB!+).KRas(g~GTP,GTPase!4) 	PI3Kkp5

499 PI3K(p110_RBD,G_p85_nSH2_cSH2!2).Gab1(Y447~P!2,PRS1_PRS2!1).Grb2(cSH3!1,SH2r!+) + KRas(g~GTP,GTPase) -> \
 PI3K(G_p85_nSH2_cSH2!2,p110_RBD!4).Gab1(Y447~P!2,PRS1_PRS2!1).Grb2(cSH3!1,SH2r!+).KRas(g~GTP,GTPase!4)	PI3Kkp5

500 PI3K(p110_RBD,G_p85_nSH2_cSH2!3).Gab1(Y472~P!3,PRS1_PRS2!1).Grb2(cSH3!1,SH2s!2).p52Shc1(Y317~P!2,PTB!+) + KRas(g~GTP,GTPase) -> \
 PI3K(p110_RBD!4,G_p85_nSH2_cSH2!3).Gab1(Y472~P!3,PRS1_PRS2!1).Grb2(cSH3!1,SH2s!2).p52Shc1(Y317~P!2,PTB!+).KRas(g~GTP,GTPase!4) 	PI3Kkp5

501 PI3K(p110_RBD,G_p85_nSH2_cSH2!2).Gab1(Y472~P!2,PRS1_PRS2!1).Grb2(cSH3!1,SH2r!+) + KRas(g~GTP,GTPase) -> \
 PI3K(G_p85_nSH2_cSH2!2,p110_RBD!4).Gab1(Y472~P!2,PRS1_PRS2!1).Grb2(cSH3!1,SH2r!+).KRas(g~GTP,GTPase!4)	PI3Kkp5

502 PI3K(p110_RBD,G_p85_nSH2_cSH2!3).Gab1(Y619~P!3,PRS1_PRS2!1).Grb2(cSH3!1,SH2s!2).p52Shc1(Y317~P!2,PTB!+) + KRas(g~GTP,GTPase) -> \
 PI3K(p110_RBD!4,G_p85_nSH2_cSH2!3).Gab1(Y619~P!3,PRS1_PRS2!1).Grb2(cSH3!1,SH2s!2).p52Shc1(Y317~P!2,PTB!+).KRas(g~GTP,GTPase!4) 	PI3Kkp5

503 PI3K(p110_RBD,G_p85_nSH2_cSH2!2).Gab1(Y619~P!2,PRS1_PRS2!1).Grb2(cSH3!1,SH2r!+) + KRas(g~GTP,GTPase) -> \
 PI3K(G_p85_nSH2_cSH2!2,p110_RBD!4).Gab1(Y619~P!2,PRS1_PRS2!1).Grb2(cSH3!1,SH2r!+).KRas(g~GTP,GTPase!4)	PI3Kkp5

504 PI3K(p110_RBD!1).KRas(GTPase!1) -> \
 PI3K(p110_RBD) + KRas(GTPase)	PI3Kkp6
}}}
```

```
''Raf1 reversibly binds ~KRas'' (see Arrow 20 in [[Contact Map]])
{{{Raf1(RBD)+KRas(GTPase)<->Raf1(RBD!1).KRas(GTPase!1)}}}
!Rules
{{{
227 KRas(g~GTP,GTPase) + Raf1(RBD) -> \
 KRas(g~GTP,GTPase!1).Raf1(RBD!1)       MAPKkp1

233 Raf1(RBD!1).KRas(GTPase!1) -> \
  Raf1(RBD) + KRas(GTPase)              MAPKkp7
}}}
```

```
''Sos1 reversibly binds ~KRas'' (see Arrow 18 in [[Contact Map]])
{{{Sos1(GEF)+KRas(GTPase)<->Sos1(GEF!1).KRas(GTPase!1)}}}
{{{Sos1(REM)+KRas(GTPase)<->Sos1(REM!1).KRas(GTPase!1)}}}
!Rules
{{{
218 Sos1(PRS!2,GEF,S1132~O,S1167~O,S1178~O,S1193~O).Grb2(nSH3!2,SH2r!+) + KRas(GTPase,g~GDP) -> \
 Sos1(PRS!2,GEF!3,S1132~O,S1167~O,S1178~O,S1193~O).Grb2(nSH3!2,SH2r!+).KRas(GTPase!3,g~GDP)       Sos1kp1

219 Sos1(PRS!2,GEF,S1132~O,S1167~O,S1178~O,S1193~O).Grb2(nSH3!2,SH2s!1).p52Shc1(PTB!+,Y317~P!1) + KRas(GTPase,g~GDP) -> \
 Sos1(PRS!2,GEF!3,S1132~O,S1167~O,S1178~O,S1193~O).Grb2(nSH3!2,SH2s!1).p52Shc1(PTB!+,Y317~P!1).KRas(GTPase!3,g~GDP)     Sos1kp1

220 Sos1(PRS!2,REM,S1132~O,S1167~O,S1178~O,S1193~O).Grb2(nSH3!2,SH2r!+) + KRas(GTPase,g~GTP) -> \
 Sos1(PRS!2,REM!3,S1132~O,S1167~O,S1178~O,S1193~O).Grb2(nSH3!2,SH2r!+).KRas(GTPase!3,g~GTP)     Sos1kp3

221 Sos1(PRS!2,REM,S1132~O,S1167~O,S1178~O,S1193~O).Grb2(nSH3!2,SH2s!1).p52Shc1(PTB!+,Y317~P!1) + KRas(GTPase,g~GTP) -> \
 Sos1(PRS!2,REM!3,S1132~O,S1167~O,S1178~O,S1193~O).Grb2(nSH3!2,SH2s!1).p52Shc1(PTB!+,Y317~P!1).KRas(GTPase!3,g~GTP)     Sos1kp3

225 Sos1(GEF!3).KRas(GTPase!3) -> \
 Sos1(GEF) + KRas(GTPase)       Sos1kp8
}}}
```

```
|The linear motifs listed in this table are considered in the model|c
|Short Name |Full Motif Name |Regular Expression |Comment |h
|G |G motifs (~G1-G5) |G1 is a P-loop (G....GK[TS])), G2 is T35, G3 is D..G, G4 is NK.D, and G5 is SAK |The GDP/GTP binding site in Ras proteins is discontinuous, T35 is part of switch I, G60 in G3 is part of switch II |
|PRS |proline-rich sequence |[RKY]..P..P |Ligand of class I ~SH3 domains |
|PRR |proline-rich region |see above |Multiple proline-rich sequences |
|&delta; |&delta;-domain or docking (D) domain |Two or more basic residues (K or R) followed by [LI].[LI] |Important for interaction with ERK |
|CD |Common docking domain |Two acidic residues, D..[DE] |Important for interaction with MEK, substrate specificity |

|Motif |Protein |Unique Name |Sequence |h
|G |~K-Ras |G1 |^^10^^GAGGVGKS^^17^^ |
|~|~|G2 |T35 |
|~|~|G3 |^^57^^DTAG^^60^^ |
|~|~|G4 |^^116^^NKCD^^119^^ |
|~|~|G5 |^^145^^SAK^^147^^ |
|PRS |Gab1 |~PxxP |^^340^^IPPPRPPKP^^348^^ |
|~|~|~PxxxR |^^517^^PPVDRNLKP^^525^^ |
|PRR |Sos1 |~PRS1 |^^1151^^PPVPPR^^1156^^ |
|~|~|~PRS2 |^^1179^^PPAIPPR^^1185^^ |
|~|~|~PRS3 |^^1211^^PPLLPPR^^1217^^ |
|~|~|~PRS4 |^^1290^^PPVPPR^^1295^^ |
|&delta; |~MEK1 |D |^^1^^MPKKKPTPIQLNPNP^^15^^ |
|CD |~ERK2 |CD |^^313^^LEQYYDPSDEPIAE^^326^^ |

The [[ELM | http://elm.eu.org/]] resource is an excellent source of additional information about linear motifs and functional sites in proteins.
```

```
See ''~MEK1 (dual specificity mitogen-activated protein kinase kinase 1)'' in [[Proteins]]
!BNGL
{{{MEK1(delta,S218~O~P,S222~O~P,T292~O~P,loc~C)}}}
!Summary
~MEK1 (represented by {{{MEK1}}} in the model) is a dual specificity MAP kinase kinase.  The residues S218 and S222 are substrates of Raf-1.  These residues correspond to S222 and S226 in ~MEK2, which is closely related to ~MEK1.  The residue T292 is a substrate of ~ERK1/2.  The so-called &delta; domain (aka D domain) is actually a linear motif.
!DOG
[img[DOGS/MEK1.jpg]]
!Reading
*[[OMIM ID 176872 | http://www.ncbi.nlm.nih.gov/omim/176872]]
*Dhillon AS, Hagan S, Rath O, Kolch W (2007) MAP kinase signalling pathways in cancer. Oncogene 26:3279-3290. [[PMID: 17496922 | http://www.ncbi.nlm.nih.gov/pubmed/17496922]]
```

```
''~MEK1 catalyzes phosphorylation of ~ERK2'' (see Arrow 27 in [[Contact Map]])
{{{ERK2(T185~O)->ERK2(T185~P)}}}
{{{ERK2(Y187~O)->ERK2(Y187~P)}}}
!Rules
{{{
248 ERK2(CD!1,T185~O).MEK1(delta!1,S218~P!?,S222~P!?,T292~O) -> \
 ERK2(CD!1,T185~P).MEK1(delta!1,S218~P!?,S222~P!?,T292~O)        MAPKkp17

249 ERK2(CD!1,Y187~O).MEK1(delta!1,S218~P!?,S222~P!?,T292~O) -> \
 ERK2(CD!1,Y187~P).MEK1(delta!1,S218~P!?,S222~P!?,T292~O)        MAPKkp18
}}}
```

```
[[Introduction]]
!!!
[[Contact Map]]
!!!
+++[Metadata <small>&raquo;</small>]
&nbsp;[[Compartments]]
&nbsp;[[Proteins]]
&nbsp;[[Domains]]
&nbsp;[[Linear Motifs]]
&nbsp;[[Phosphorylation Sites]]
&nbsp;[[Metabolites]]
&nbsp;[[Interactions]]
===
!!!
+++[Molecule Types <small>&raquo;</small>]
&nbsp;<html><small><strong>Ligands</strong></small></html>
&nbsp;[[EGF]]
&nbsp;[[HRG]]
&nbsp;<html><small><strong>Receptors</strong></small></html>
&nbsp;[[EGFR]]
&nbsp;[[ErbB2]]
&nbsp;[[ErbB3]]
&nbsp;[[ErbB4]]
&nbsp;<html><small><strong>Adapters</strong></small></html>
&nbsp;[[Grb2]]
&nbsp;[[p52Shc1]]
&nbsp;[[Gab1]]
&nbsp;<html><small><strong>in Ras/MAPK pathway</strong></small></html>
&nbsp;[[p120RasGAP]]
&nbsp;[[Sos1]]
&nbsp;[[KRas]]
&nbsp;[[Raf1]]
&nbsp;[[MEK1]]
&nbsp;[[ERK2]]
&nbsp;<html><small><strong>in PI3K/AKT pathway</strong></small></html>
&nbsp;[[PI3K]]
&nbsp;[[PIP3]]
&nbsp;[[PDK1]]
&nbsp;[[Akt1]]

===
!!!
+++[Rules <small>&raquo;</small>]
+++[&nbsp;<strong><small>for Binding</small></strong> <small>&raquo;</small>]
&nbsp;[[Akt1:PIP3]]
&nbsp;[[EGF:EGFR]]
&nbsp;[[EGFR:EGFR]]
&nbsp;[[EGFR:ErbB2]]
&nbsp;[[EGFR:ErbB3]]
&nbsp;[[EGFR:p120RasGAP]]
&nbsp;[[ErbB:Grb2]]
&nbsp;[[ErbB:p52Shc1]]
&nbsp;[[ErbB2:ErbB2]]
&nbsp;[[ErbB2:ErbB3]]
&nbsp;[[ErbB3:ErbB3]]
&nbsp;[[ErbB3:HRG]]
&nbsp;[[ErbB3:PI3K]]
&nbsp;[[ERK2:MEK1]]
&nbsp;[[Gab1:Grb2]]
&nbsp;[[Gab1:PI3K]]
&nbsp;[[Gab1:PIP3]]
&nbsp;[[Grb2:p52Shc1]]
&nbsp;[[Grb2:Sos1]]
&nbsp;[[KRas:PI3K]]
&nbsp;[[KRas:Raf1]]
&nbsp;[[KRas:Sos1]]
&nbsp;[[PDK1:PIP3]]

===

+++[&nbsp;<strong><small>for Catalysis</small></strong> <small>&raquo;</small>]
&nbsp;[[Akt1 Activity]]
&nbsp;[[EGFR Activity]]
&nbsp;[[ErbB2 Activity]]
&nbsp;[[ERK2 Activity]]
&nbsp;[[MEK1 Activity]]
&nbsp;[[p120RasGAP Activity]]
&nbsp;[[Raf1 Activity]]
&nbsp;[[Raf1-targeted Activity]]
&nbsp;[[PDK1 Activity]]
&nbsp;[[PI3K Activity]]
&nbsp;[[Phosphatase Activity]]
===

+++[&nbsp;<strong><small>for Exchange</small></strong> <small>&raquo;</small>]
&nbsp;[[Sos1 Activity]]
===

+++[&nbsp;<strong><small>for Transport</small></strong> <small>&raquo;</small>]
&nbsp;[[Receptor Trafficking]]
===

===
!!!
[[Full Model Specification]]
!!!
```

```
Matthew S. Creamer
Intern
Clinical Translational Research Division
Translational Genomics Research Institute
13208 E. Shea Blvd., Suite 110
Scottsdale, AZ 85259
E-mail: [[msc72@nau.edu | mailto:msc72@nau.edu]]
```

```
|Metabolite Name |KEGG ID |Comment |h
|~PtdIns(4,5)P2 or ~PIP2	|[[C04637 | http://www.genome.jp/dbget-bin/www_bget?cpd:C04637]] |Phospholipid |
|~PtdIns(3,4,5)P3 or ~PIP3	|[[C05981 | http://www.genome.jp/dbget-bin/www_bget?cpd:C05981]] |Phospholipid |
|GDP	|[[C00035 | http://www.genome.jp/dbget-bin/www_bget?cpd:C00035]] |Purine nucleotide |
|GTP	|[[C00044 | http://www.genome.jp/dbget-bin/www_bget?cpd:C00044]] |Purine nucleotide |

The [[KEGG | http://www.genome.jp/kegg/]] and [[ChEBI | http://www.ebi.ac.uk/chebi/]] databases are excellent sources of additional information about small-molecule metabolites.
```

```
/***
|Name|NestedSlidersPlugin|
|Source|http://www.TiddlyTools.com/#NestedSlidersPlugin|
|Documentation|http://www.TiddlyTools.com/#NestedSlidersPluginInfo|
|Version|2.4.9|
|Author|Eric Shulman|
|License|http://www.TiddlyTools.com/#LegalStatements|
|~CoreVersion|2.1|
|Type|plugin|
|Description|show content in nest-able sliding/floating panels, without creating separate tiddlers for each panel's content|
!!!!!Documentation
>see [[NestedSlidersPluginInfo]]
!!!!!Configuration
<<<
<<option chkFloatingSlidersAnimate>> allow floating sliders to animate when opening/closing
>Note: This setting can cause 'clipping' problems in some versions of InternetExplorer.
>In addition, for floating slider animation to occur you must also allow animation in general (see [[AdvancedOptions]]).
<<<
!!!!!Revisions
<<<
2008.11.15 - 2.4.9 in adjustNestedSlider(), don't make adjustments if panel is marked as 'undocked' (CSS class).  In onClickNestedSlider(), SHIFT-CLICK docks panel (see [[MoveablePanelPlugin]])
|please see [[NestedSlidersPluginInfo]] for additional revision details|
2005.11.03 - 1.0.0 initial public release.  Thanks to RodneyGomes, GeoffSlocock, and PaulPetterson for suggestions and experiments.
<<<
!!!!!Code
***/
//{{{
version.extensions.NestedSlidersPlugin= {major: 2, minor: 4, revision: 9, date: new Date(2008,11,15)};

// options for deferred rendering of sliders that are not initially displayed
if (config.options.chkFloatingSlidersAnimate===undefined)
	config.options.chkFloatingSlidersAnimate=false; // avoid clipping problems in IE

// default styles for 'floating' class
setStylesheet(".floatingPanel { position:absolute; z-index:10; padding:0.5em; margin:0em; \
	background-color:#eee; color:#000; border:1px solid #000; text-align:left; }","floatingPanelStylesheet");

// if removeCookie() function is not defined by TW core, define it here.
if (window.removeCookie===undefined) {
	window.removeCookie=function(name) {
		document.cookie = name+'=; expires=Thu, 01-Jan-1970 00:00:01 UTC; path=/;';
	}
}

config.formatters.push( {
	name: "nestedSliders",
	match: "\\n?\\+{3}",
	terminator: "\\s*\\={3}\\n?",
	lookahead: "\\n?\\+{3}(\\+)?(\\([^\\)]*\\))?(\\!*)?(\\^(?:[^\\^\\*\\@\\[\\>]*\\^)?)?(\\*)?(\\@)?(?:\\{\\{([\\w]+[\\s\\w]*)\\{)?(\\[[^\\]]*\\])?(\\[[^\\]]*\\])?(?:\\}{3})?(\\#[^:]*\\:)?(\\>)?(\\.\\.\\.)?\\s*",
	handler: function(w)
		{
			lookaheadRegExp = new RegExp(this.lookahead,"mg");
			lookaheadRegExp.lastIndex = w.matchStart;
			var lookaheadMatch = lookaheadRegExp.exec(w.source)
			if(lookaheadMatch && lookaheadMatch.index == w.matchStart)
			{
				var defopen=lookaheadMatch[1];
				var cookiename=lookaheadMatch[2];
				var header=lookaheadMatch[3];
				var panelwidth=lookaheadMatch[4];
				var transient=lookaheadMatch[5];
				var hover=lookaheadMatch[6];
				var buttonClass=lookaheadMatch[7];
				var label=lookaheadMatch[8];
				var openlabel=lookaheadMatch[9];
				var panelID=lookaheadMatch[10];
				var blockquote=lookaheadMatch[11];
				var deferred=lookaheadMatch[12];

				// location for rendering button and panel
				var place=w.output;

				// default to closed, no cookie, no accesskey, no alternate text/tip
				var show="none"; var cookie=""; var key="";
				var closedtext=">"; var closedtip="";
				var openedtext="<"; var openedtip="";

				// extra "+", default to open
				if (defopen) show="block";

				// cookie, use saved open/closed state
				if (cookiename) {
					cookie=cookiename.trim().slice(1,-1);
					cookie="chkSlider"+cookie;
					if (config.options[cookie]==undefined)
						{ config.options[cookie] = (show=="block") }
					show=config.options[cookie]?"block":"none";
				}

				// parse label/tooltip/accesskey: [label=X|tooltip]
				if (label) {
					var parts=label.trim().slice(1,-1).split("|");
					closedtext=parts.shift();
					if (closedtext.substr(closedtext.length-2,1)=="=")
						{ key=closedtext.substr(closedtext.length-1,1); closedtext=closedtext.slice(0,-2); }
					openedtext=closedtext;
					if (parts.length) closedtip=openedtip=parts.join("|");
					else { closedtip="show "+closedtext; openedtip="hide "+closedtext; }
				}

				// parse alternate label/tooltip: [label|tooltip]
				if (openlabel) {
					var parts=openlabel.trim().slice(1,-1).split("|");
					openedtext=parts.shift();
					if (parts.length) openedtip=parts.join("|");
					else openedtip="hide "+openedtext;
				}

				var title=show=='block'?openedtext:closedtext;
				var tooltip=show=='block'?openedtip:closedtip;

				// create the button
				if (header) { // use "Hn" header format instead of button/link
					var lvl=(header.length>5)?5:header.length;
					var btn = createTiddlyElement(createTiddlyElement(place,"h"+lvl,null,null,null),"a",null,buttonClass,title);
					btn.onclick=onClickNestedSlider;
					btn.setAttribute("href","javascript:;");
					btn.setAttribute("title",tooltip);
				}
				else
					var btn = createTiddlyButton(place,title,tooltip,onClickNestedSlider,buttonClass);
				btn.innerHTML=title; // enables use of HTML entities in label

				// set extra button attributes
				btn.setAttribute("closedtext",closedtext);
				btn.setAttribute("closedtip",closedtip);
				btn.setAttribute("openedtext",openedtext);
				btn.setAttribute("openedtip",openedtip);
				btn.sliderCookie = cookie; // save the cookiename (if any) in the button object
				btn.defOpen=defopen!=null; // save default open/closed state (boolean)
				btn.keyparam=key; // save the access key letter ("" if none)
				if (key.length) {
					btn.setAttribute("accessKey",key); // init access key
					btn.onfocus=function(){this.setAttribute("accessKey",this.keyparam);}; // **reclaim** access key on focus
				}
				btn.setAttribute("hover",hover?"true":"false");
				btn.onmouseover=function(ev) {
					// optional 'open on hover' handling
					if (this.getAttribute("hover")=="true" && this.sliderPanel.style.display=='none') {
						document.onclick.call(document,ev); // close transients
						onClickNestedSlider(ev); // open this slider
					}
					// mouseover on button aligns floater position with button
					if (window.adjustSliderPos) window.adjustSliderPos(this.parentNode,this,this.sliderPanel);
				}

				// create slider panel
				var panelClass=panelwidth?"floatingPanel":"sliderPanel";
				if (panelID) panelID=panelID.slice(1,-1); // trim off delimiters
				var panel=createTiddlyElement(place,"div",panelID,panelClass,null);
				panel.button = btn; // so the slider panel know which button it belongs to
				btn.sliderPanel=panel; // so the button knows which slider panel it belongs to
				panel.defaultPanelWidth=(panelwidth && panelwidth.length>2)?panelwidth.slice(1,-1):"";
				panel.setAttribute("transient",transient=="*"?"true":"false");
				panel.style.display = show;
				panel.style.width=panel.defaultPanelWidth;
				panel.onmouseover=function(event) // mouseover on panel aligns floater position with button
					{ if (window.adjustSliderPos) window.adjustSliderPos(this.parentNode,this.button,this); }

				// render slider (or defer until shown)
				w.nextMatch = lookaheadMatch.index + lookaheadMatch[0].length;
				if ((show=="block")||!deferred) {
					// render now if panel is supposed to be shown or NOT deferred rendering
					w.subWikify(blockquote?createTiddlyElement(panel,"blockquote"):panel,this.terminator);
					// align floater position with button
					if (window.adjustSliderPos) window.adjustSliderPos(place,btn,panel);
				}
				else {
					var src = w.source.substr(w.nextMatch);
					var endpos=findMatchingDelimiter(src,"+++","===");
					panel.setAttribute("raw",src.substr(0,endpos));
					panel.setAttribute("blockquote",blockquote?"true":"false");
					panel.setAttribute("rendered","false");
					w.nextMatch += endpos+3;
					if (w.source.substr(w.nextMatch,1)=="\n") w.nextMatch++;
				}
			}
		}
	}
)

function findMatchingDelimiter(src,starttext,endtext) {
	var startpos = 0;
	var endpos = src.indexOf(endtext);
	// check for nested delimiters
	while (src.substring(startpos,endpos-1).indexOf(starttext)!=-1) {
		// count number of nested 'starts'
		var startcount=0;
		var temp = src.substring(startpos,endpos-1);
		var pos=temp.indexOf(starttext);
		while (pos!=-1)  { startcount++; pos=temp.indexOf(starttext,pos+starttext.length); }
		// set up to check for additional 'starts' after adjusting endpos
		startpos=endpos+endtext.length;
		// find endpos for corresponding number of matching 'ends'
		while (startcount && endpos!=-1) {
			endpos = src.indexOf(endtext,endpos+endtext.length);
			startcount--;
		}
	}
	return (endpos==-1)?src.length:endpos;
}
//}}}
//{{{
window.onClickNestedSlider=function(e)
{
	if (!e) var e = window.event;
	var theTarget = resolveTarget(e);
	while (theTarget && theTarget.sliderPanel==undefined) theTarget=theTarget.parentNode;
	if (!theTarget) return false;
	var theSlider = theTarget.sliderPanel;
	var isOpen = theSlider.style.display!="none";

	// if SHIFT-CLICK, dock panel first (see [[MoveablePanelPlugin]])
	if (e.shiftKey && config.macros.moveablePanel) config.macros.moveablePanel.dock(theSlider,e);

	// toggle label
	theTarget.innerHTML=isOpen?theTarget.getAttribute("closedText"):theTarget.getAttribute("openedText");
	// toggle tooltip
	theTarget.setAttribute("title",isOpen?theTarget.getAttribute("closedTip"):theTarget.getAttribute("openedTip"));

	// deferred rendering (if needed)
	if (theSlider.getAttribute("rendered")=="false") {
		var place=theSlider;
		if (theSlider.getAttribute("blockquote")=="true")
			place=createTiddlyElement(place,"blockquote");
		wikify(theSlider.getAttribute("raw"),place);
		theSlider.setAttribute("rendered","true");
	}

	// show/hide the slider
	if(config.options.chkAnimate && (!hasClass(theSlider,'floatingPanel') || config.options.chkFloatingSlidersAnimate))
		anim.startAnimating(new Slider(theSlider,!isOpen,e.shiftKey || e.altKey,"none"));
	else
		theSlider.style.display = isOpen ? "none" : "block";

	// reset to default width (might have been changed via plugin code)
	theSlider.style.width=theSlider.defaultPanelWidth;

	// align floater panel position with target button
	if (!isOpen && window.adjustSliderPos) window.adjustSliderPos(theSlider.parentNode,theTarget,theSlider);

	// if showing panel, set focus to first 'focus-able' element in panel
	if (theSlider.style.display!="none") {
		var ctrls=theSlider.getElementsByTagName("*");
		for (var c=0; c<ctrls.length; c++) {
			var t=ctrls[c].tagName.toLowerCase();
			if ((t=="input" && ctrls[c].type!="hidden") || t=="textarea" || t=="select")
				{ try{ ctrls[c].focus(); } catch(err){;} break; }
		}
	}
	var cookie=theTarget.sliderCookie;
	if (cookie && cookie.length) {
		config.options[cookie]=!isOpen;
		if (config.options[cookie]!=theTarget.defOpen) window.saveOptionCookie(cookie);
		else window.removeCookie(cookie); // remove cookie if slider is in default display state
	}

	// prevent SHIFT-CLICK from being processed by browser (opens blank window... yuck!)
	// prevent clicks *within* a slider button from being processed by browser
	// but allow plain click to bubble up to page background (to close transients, if any)
	if (e.shiftKey || theTarget!=resolveTarget(e))
		{ e.cancelBubble=true; if (e.stopPropagation) e.stopPropagation(); }
	Popup.remove(); // close open popup (if any)
	return false;
}
//}}}
//{{{
// click in document background closes transient panels
document.nestedSliders_savedOnClick=document.onclick;
document.onclick=function(ev) { if (!ev) var ev=window.event; var target=resolveTarget(ev);

	if (document.nestedSliders_savedOnClick)
		var retval=document.nestedSliders_savedOnClick.apply(this,arguments);
	// if click was inside a popup... leave transient panels alone
	var p=target; while (p) if (hasClass(p,"popup")) break; else p=p.parentNode;
	if (p) return retval;
	// if click was inside transient panel (or something contained by a transient panel), leave it alone
	var p=target; while (p) {
		if ((hasClass(p,"floatingPanel")||hasClass(p,"sliderPanel"))&&p.getAttribute("transient")=="true") break;
		p=p.parentNode;
	}
	if (p) return retval;
	// otherwise, find and close all transient panels...
	var all=document.all?document.all:document.getElementsByTagName("DIV");
	for (var i=0; i<all.length; i++) {
		 // if it is not a transient panel, or the click was on the button that opened this panel, don't close it.
		if (all[i].getAttribute("transient")!="true" || all[i].button==target) continue;
		// otherwise, if the panel is currently visible, close it by clicking it's button
		if (all[i].style.display!="none") window.onClickNestedSlider({target:all[i].button})
		if (!hasClass(all[i],"floatingPanel")&&!hasClass(all[i],"sliderPanel")) all[i].style.display="none";
	}
	return retval;
};
//}}}
//{{{
// adjust floating panel position based on button position
if (window.adjustSliderPos==undefined) window.adjustSliderPos=function(place,btn,panel) {
	if (hasClass(panel,"floatingPanel") && !hasClass(panel,"undocked")) {
		// see [[MoveablePanelPlugin]] for use of 'undocked'
		var rightEdge=document.body.offsetWidth-1;
		var panelWidth=panel.offsetWidth;
		var left=0;
		var top=btn.offsetHeight;
		if (place.style.position=="relative" && findPosX(btn)+panelWidth>rightEdge) {
			left-=findPosX(btn)+panelWidth-rightEdge; // shift panel relative to button
			if (findPosX(btn)+left<0) left=-findPosX(btn); // stay within left edge
		}
		if (place.style.position!="relative") {
			var left=findPosX(btn);
			var top=findPosY(btn)+btn.offsetHeight;
			var p=place; while (p && !hasClass(p,'floatingPanel')) p=p.parentNode;
			if (p) { left-=findPosX(p); top-=findPosY(p); }
			if (left+panelWidth>rightEdge) left=rightEdge-panelWidth;
			if (left<0) left=0;
		}
		panel.style.left=left+"px"; panel.style.top=top+"px";
	}
}
//}}}
//{{{
// TW2.1 and earlier:
// hijack Slider stop handler so overflow is visible after animation has completed
Slider.prototype.coreStop = Slider.prototype.stop;
Slider.prototype.stop = function()
	{ this.coreStop.apply(this,arguments); this.element.style.overflow = "visible"; }

// TW2.2+
// hijack Morpher stop handler so sliderPanel/floatingPanel overflow is visible after animation has completed
if (version.major+.1*version.minor+.01*version.revision>=2.2) {
	Morpher.prototype.coreStop = Morpher.prototype.stop;
	Morpher.prototype.stop = function() {
		this.coreStop.apply(this,arguments);
		var e=this.element;
		if (hasClass(e,"sliderPanel")||hasClass(e,"floatingPanel")) {
			// adjust panel overflow and position after animation
			e.style.overflow = "visible";
			if (window.adjustSliderPos) window.adjustSliderPos(e.parentNode,e.button,e);
		}
	};
}
//}}}
```

```
See ''~PDK1 (3-phosphoinositide-dependent protein kinase 1)'' in [[Proteins]]
!BNGL
{{{PDK1(PH,STkinase,loc~C)}}}
!Summary
~PDK1 is a S/T kinase.  It contains a pleckstrin homology (PH) domain, which recognizes ~PIP3.
!DOG
[img[DOGS/PDK1.jpg]]
!Reading
*[[OMIM ID 605213 | http://www.ncbi.nlm.nih.gov/omim/605213]]
*Biondi RM (2004) Phosphoinositide-dependent protein kinase 1, a sensor of protein conformation. Trends Biochem Sci 29:136-142. [[PMID: 15003271 | http://www.ncbi.nlm.nih.gov/pubmed/15003271]]
```

```
''~PDK1 catalyzes phosphorylation of Akt1'' (see Arrow 31 in [[Contact Map]])
{{{Akt1(T308~O)->Akt1(T308~P)}}}
{{{Akt1(S473~O)->Akt1(S473~P)}}}
!Rules
{{{
532 PDK1(PH!+,STkinase!1).Akt1(PH!+,T308~O!1) -> \
 PDK1(PH!+,STkinase!1).Akt1(PH!+,T308~P!1)		Akt1kp4

534 Akt1(T308~P!?,S473~O) -> \
 Akt1(T308~P!?,S473~P)		Akt1kp6
}}}
```

```
''~PDK1 reversibly binds ~PIP3'' (see Arrow 30 in [[Contact Map]])
{{{PDK1(PH)+PIP3(C3P)<->PDK1(PH!1).PIP3(C3P!1)}}}
!Rules
{{{
530 PDK1(PH) + PIP3(C3P,two~F) <-> \
 PDK1(PH!1).PIP3(C3P!1,two~F)		Akt1kp2,Akt1km2
}}}
```

```
See ''Class IA ~PI3K (phophoinositide 3-kinase composed of p85&alpha; and p110&alpha;)'' in [[Proteins]]
!BNGL
{{{PI3K(lipid_kinase,R_p85_nSH2_cSH2,G_p85_nSH2_cSH2,p110_RBD,loc~C)}}}
!Summary
The term "~PI3K" refers to a family of phosphoinositide 3-kinases.  In ~ErbB signaling, class IA ~PI3K is important.  A class IA ~PI3K is composed of two subunits: 1) a regulatory subunit (p85&alpha; , p55&alpha;, p50&alpha;, p85&beta;, or p55&gamma;) that contains two tandem ~SH2 domains and other components and 2) a catalytic subunit (p110&alpha;, p110&beta; or p110&delta;) that contains a Ras binding domain (RBD), a catalytic domain (~PI3Kc), and other components.  The ~SH2 domains recognize ~YxxM motifs.  We assume that the p85&alpha;-p110&alpha; heterodimer is representative of the class IA ~PI3K involved in ~ErbB signaling.
!DOG
[img[DOGS/p85alpha.jpg]]
[img[DOGS/p110alpha.jpg]]
!Reading
*[[OMIM ID 171833 | http://www.ncbi.nlm.nih.gov/omim/171833]] (p85&alpha;)
*[[OMIM ID 171834 | http://www.ncbi.nlm.nih.gov/omim/171834]] (p110&alpha;)
*Amzel LM, Huang CH, Mandelker D, Lengauer C, Gabelli SB, Vogelstein B (2008) Structural comparisons of class I phosphoinositide 3-kinases. Nat Rev Cancer 8:665-669. [[PMID: 18633356 | http://www.ncbi.nlm.nih.gov/pubmed/18633356]]
```

```
''~PI3K catalyzes phosphorylation of ~PIP2 to generate ~PIP3'' (see Arrow 23 in [[Contact Map]])
{{{0->PIP3(C3P)}}}
!Rules
{{{
512 PI3K(G_p85_nSH2_cSH2!3).Gab1(Y447~P!3,PRS1_PRS2!1,PH).Grb2(cSH3!1,SH2s!2).p52Shc1(Y317~P!2,PTB!+) -> \
 PIP3(C3P,two~F,loc~M) + PI3K(G_p85_nSH2_cSH2!3).Gab1(Y447~P!3,PRS1_PRS2!1,PH).Grb2(cSH3!1,SH2s!2).p52Shc1(Y317~P!2,PTB!+) 	PIP3kp1

513 PI3K(G_p85_nSH2_cSH2!3).Gab1(Y472~P!3,PRS1_PRS2!1,PH).Grb2(cSH3!1,SH2s!2).p52Shc1(Y317~P!2,PTB!+) -> \
 PIP3(C3P,two~F,loc~M) + PI3K(G_p85_nSH2_cSH2!3).Gab1(Y472~P!3,PRS1_PRS2!1,PH).Grb2(cSH3!1,SH2s!2).p52Shc1(Y317~P!2,PTB!+)	PIP3kp1

514 PI3K(G_p85_nSH2_cSH2!3).Gab1(Y619~P!3,PRS1_PRS2!1,PH).Grb2(cSH3!1,SH2s!2).p52Shc1(Y317~P!2,PTB!+) -> \
 PIP3(C3P,two~F,loc~M) + PI3K(G_p85_nSH2_cSH2!3).Gab1(Y619~P!3,PRS1_PRS2!1,PH).Grb2(cSH3!1,SH2s!2).p52Shc1(Y317~P!2,PTB!+)	PIP3kp1

515 PI3K(G_p85_nSH2_cSH2!3).Gab1(Y447~P!3,PRS1_PRS2!1,PH).Grb2(cSH3!1,SH2r!+) -> \
 PIP3(C3P,two~F,loc~M) + PI3K(G_p85_nSH2_cSH2!3).Gab1(Y447~P!3,PRS1_PRS2!1,PH).Grb2(cSH3!1,SH2r!+)	PIP3kp1

516 PI3K(G_p85_nSH2_cSH2!3).Gab1(Y472~P!3,PRS1_PRS2!1,PH).Grb2(cSH3!1,SH2r!+) -> \
 PIP3(C3P,two~F,loc~M) + PI3K(G_p85_nSH2_cSH2!3).Gab1(Y472~P!3,PRS1_PRS2!1,PH).Grb2(cSH3!1,SH2r!+)	PIP3kp1

517 PI3K(G_p85_nSH2_cSH2!3).Gab1(Y619~P!3,PRS1_PRS2!1,PH).Grb2(cSH3!1,SH2r!+) -> \
 PIP3(C3P,two~F,loc~M) + PI3K(G_p85_nSH2_cSH2!3).Gab1(Y619~P!3,PRS1_PRS2!1,PH).Grb2(cSH3!1,SH2r!+)	PIP3kp1

518 PI3K(G_p85_nSH2_cSH2!3,p110_RBD!+).Gab1(Y447~P!3,PRS1_PRS2!1).Grb2(cSH3!1,SH2s!2).p52Shc1(Y317~P!2,PTB!+) -> \
 PIP3(C3P,two~F,loc~M) + PI3K(G_p85_nSH2_cSH2!3,p110_RBD!+).Gab1(Y447~P!3,PRS1_PRS2!1).Grb2(cSH3!1,SH2s!2).p52Shc1(Y317~P!2,PTB!+)	PIP3kp1_5

519 PI3K(G_p85_nSH2_cSH2!3,p110_RBD!+).Gab1(Y472~P!3,PRS1_PRS2!1).Grb2(cSH3!1,SH2s!2).p52Shc1(Y317~P!2,PTB!+) -> \
 PIP3(C3P,two~F,loc~M) + PI3K(G_p85_nSH2_cSH2!3,p110_RBD!+).Gab1(Y472~P!3,PRS1_PRS2!1).Grb2(cSH3!1,SH2s!2).p52Shc1(Y317~P!2,PTB!+)	PIP3kp1_5

520 PI3K(G_p85_nSH2_cSH2!3,p110_RBD!+).Gab1(Y619~P!3,PRS1_PRS2!1).Grb2(cSH3!1,SH2s!2).p52Shc1(Y317~P!2,PTB!+) -> \
 PIP3(C3P,two~F,loc~M) + PI3K(G_p85_nSH2_cSH2!3,p110_RBD!+).Gab1(Y619~P!3,PRS1_PRS2!1).Grb2(cSH3!1,SH2s!2).p52Shc1(Y317~P!2,PTB!+)	PIP3kp1_5

521 PI3K(G_p85_nSH2_cSH2!3,p110_RBD!+).Gab1(Y447~P!3,PRS1_PRS2!1).Grb2(cSH3!1,SH2r!+) -> \
 PIP3(C3P,two~F,loc~M) + PI3K(G_p85_nSH2_cSH2!3,p110_RBD!+).Gab1(Y447~P!3,PRS1_PRS2!1).Grb2(cSH3!1,SH2r!+)	PIP3kp1_5

522 PI3K(G_p85_nSH2_cSH2!3,p110_RBD!+).Gab1(Y472~P!3,PRS1_PRS2!1).Grb2(cSH3!1,SH2r!+) -> \
 PIP3(C3P,two~F,loc~M) + PI3K(G_p85_nSH2_cSH2!3,p110_RBD!+).Gab1(Y472~P!3,PRS1_PRS2!1).Grb2(cSH3!1,SH2r!+)	PIP3kp1_5

523 PI3K(G_p85_nSH2_cSH2!3,p110_RBD!+).Gab1(Y619~P!3,PRS1_PRS2!1).Grb2(cSH3!1,SH2r!+) -> \
 PIP3(C3P,two~F,loc~M) + PI3K(G_p85_nSH2_cSH2!3,p110_RBD!+).Gab1(Y619~P!3,PRS1_PRS2!1).Grb2(cSH3!1,SH2r!+)	PIP3kp1_5

524 PI3K(G_p85_nSH2_cSH2!1).Gab1(Y447~P!1,PH!+) -> \
 PIP3(C3P,two~F,loc~M) + PI3K(G_p85_nSH2_cSH2!1).Gab1(Y447~P!1,PH!+)	PIP3kp1

525 PI3K(G_p85_nSH2_cSH2!1).Gab1(Y472~P!1,PH!+) -> \
 PIP3(C3P,two~F,loc~M) + PI3K(G_p85_nSH2_cSH2!1).Gab1(Y472~P!1,PH!+)	PIP3kp1

526 PI3K(G_p85_nSH2_cSH2!1).Gab1(Y619~P!1,PH!+) -> \
 PIP3(C3P,two~F,loc~M) + PI3K(G_p85_nSH2_cSH2!1).Gab1(Y619~P!1,PH!+)	PIP3kp1

527 PI3K(R_p85_nSH2_cSH2!+) -> \
 PIP3(C3P,two~F,loc~M) + PI3K(R_p85_nSH2_cSH2!+)	PIP3kp1
}}}
```

```
''~PtdIns(3,4,5)P3'' (see [[Metabolites]])
!BNGL
{{{PIP3(C3P,two~F~T,loc~M)}}}
!Summary
~PtdIns(3,4,5)P3 (represented by {{{PIP3}}} in the model) is the product of ~PI3K-catalyzed phosphorylation of ~PtdIns(4,5)P2 (aka ~PIP2).  The chemical structure of ~PIP3 is shown below.  The {{{PIP3}}} component {{{C3P}}} represents the carbon atom to which a phosphate group is attached as a result of ~PI3K activity.

[img[PIP3.jpg]]
```

```
''Dephosphorylation of S/T/Y residues'' (see [[Phosphorylation Sites]]) ''by phosphatases'' (not shown in [[Contact Map]])
{{{Protein(S~P)->Protein(S~O)}}}
{{{Protein(T~P)->Protein(T~O)}}}
{{{Protein(Y~P)->Protein(Y~O)}}}
!Rules
{{{
156 EGFR(Y992~P) -> \
 EGFR(Y992~O) Phosphokm1

157 EGFR(Y1068~P) -> \
 EGFR(Y1068~O) Phosphokm2

158 EGFR(Y1086~P) -> \
 EGFR(Y1086~O) Phosphokm3

159 EGFR(Y1114~P) -> \
 EGFR(Y1114~O) Phosphokm4

160 EGFR(Y1148~P) -> \
 EGFR(Y1148~O) Phosphokm5

161 EGFR(Y1173~P) -> \
 EGFR(Y1173~O) Phosphokm6

162 ErbB2(Y1139~P) -> \
 ErbB2(Y1139~O) Phosphokm7

163 ErbB2(Y1196~P) -> \
 ErbB2(Y1196~O) Phosphokm8

164 ErbB2(Y1222~P) -> \
 ErbB2(Y1222~O) Phosphokm9

165 ErbB2(Y1248~P) -> \
 ErbB2(Y1248~O) Phosphokm10

166 ErbB3(Y1054~P) -> \
 ErbB3(Y1054~O) Phosphokm11

167 ErbB3(Y1197~P) -> \
 ErbB3(Y1197~O) Phosphokm12

168 ErbB3(Y1222~P) -> \
 ErbB3(Y1222~O) Phosphokm13

169 ErbB3(Y1260~P) -> \
 ErbB3(Y1260~O) Phosphokm14

170 ErbB3(Y1276~P) -> \
 ErbB3(Y1276~O) Phosphokm15

171 ErbB3(Y1289~P) -> \
 ErbB3(Y1289~O) Phosphokm16

172 ErbB3(Y1328~P) -> \
 ErbB3(Y1328~O) Phosphokm17

173 ErbB4(Y1056~P) -> \
 ErbB4(Y1056~O) Phosphokm18

174 ErbB4(Y1188~P) -> \
 ErbB4(Y1188~O) Phosphokm19

175 ErbB4(Y1242~P) -> \
 ErbB4(Y1242~O) Phosphokm20

214 p52Shc1(Y317~P) -> \
 p52Shc1(Y317~O)    Shc1km14

224 KRas(GTPase,g~GTP) -> \
 KRas(GTPase,g~GDP) Sos1kp7

234 Raf1(S296~P) -> \
 Raf1(S296~O)     MAPKkp8

235 Raf1(S338~P) -> \
 Raf1(S338~O)     MAPKkp8

236 Raf1(Y341~P) -> \
 Raf1(Y341~O)     MAPKkp8

237 Raf1(T491~P) -> \
 Raf1(T491~O)     MAPKkp8

238 Raf1(S494~P) -> \
 Raf1(S494~O)     MAPKkp8

245 MEK1(S218~P) -> \
 MEK1(S218~O)     MAPKkp15

246 MEK1(S222~P) -> \
 MEK1(S222~O)       MAPKkp15

251 ERK2(Y187~P) -> \
 ERK2(Y187~O)       MAPKkp20

252 ERK2(T185~P) -> \
 ERK2(T185~O)       MAPKkp20

277 Sos1(S1132~P) -> \
 Sos1(S1132~O) MAPKkp45

278 Sos1(S1167~P) -> \
 Sos1(S1167~O) MAPKkp45

279 Sos1(S1178~P) -> \
 Sos1(S1178~O) MAPKkp45

280 Sos1(S1193~P) -> \
 Sos1(S1193~O) MAPKkp45

281 EGFR(T669~P) -> \
 EGFR(T669~O)	MAPKkp45

282 Raf1(S29~P) -> \
 Raf1(S29~O)	MAPKkp45

283 Raf1(S43~P) -> \
 Raf1(S43~O)	MAPKkp45

284 Raf1(S289~P) -> \
 Raf1(S289~O)	MAPKkp45

285 Raf1(S301~P) -> \
 Raf1(S301~O)	MAPKkp45

286 Raf1(S471~P) -> \
 Raf1(S471~O)	MAPKkp45

287 Raf1(S642~P) -> \
 Raf1(S642~O)	MAPKkp45

288 MEK1(T292~P) -> \
 MEK1(T292~O) MAPKkp45

289 ERK2(STkinase!1).Sos1(S1132!1) -> \
 ERK2(STkinase) + Sos1(S1132)	MAPKkp49

290 ERK2(STkinase!1).Sos1(S1167!1) -> \
 ERK2(STkinase) + Sos1(S1167)	MAPKkp49

291 ERK2(STkinase!1).Sos1(S1178!1) -> \
 ERK2(STkinase) + Sos1(S1178)	MAPKkp49

292 ERK2(STkinase!1).Sos1(S1193!1) -> \
 ERK2(STkinase) + Sos1(S1193)	MAPKkp49

293 ERK2(STkinase!1).EGFR(T669!1) -> \
 ERK2(STkinase) + EGFR(T669)	MAPKkp50

294 ERK2(STkinase!1).Raf1(S29!1) -> \
 ERK2(STkinase) + Raf1(S29)	MAPKkp51

295 ERK2(STkinase!1).Raf1(S43!1) -> \
 ERK2(STkinase) + Raf1(S43)	MAPKkp51

296 ERK2(STkinase!1).Raf1(S289!1) -> \
 ERK2(STkinase) + Raf1(S289)	MAPKkp51

297 ERK2(STkinase!1).Raf1(S301!1) -> \
 ERK2(STkinase) + Raf1(S301)	MAPKkp51

298 ERK2(STkinase!1).Raf1(S471!1) -> \
 ERK2(STkinase) + Raf1(S471)	MAPKkp51

299 ERK2(STkinase!1).Raf1(S642!1) -> \
 ERK2(STkinase) + Raf1(S642)	MAPKkp51

300 ERK2(STkinase!1).MEK1(T292!1) -> \
 ERK2(STkinase) + MEK1(T292)	MAPKkp52

474 Gab1(Y447~P) -> \
 Gab1(Y447~O)	Gab1kp169

475 Gab1(Y472~P) -> \
 Gab1(Y472~O)	Gab1kp169

476 Gab1(Y619~P) -> \
 Gab1(Y619~O)	Gab1kp169

477 Gab1(Y657~P) -> \
 Gab1(Y657~O)	Gab1kp169

478 Gab1(T312~P) -> \
 Gab1(T312~O)	Gab1kp170

479 Gab1(S381~P) -> \
 Gab1(S381~O)	Gab1kp170

480 Gab1(S454~P) -> \
 Gab1(S454~O)	Gab1kp170

481 Gab1(T476~P) -> \
 Gab1(T476~O)	Gab1kp170

482 Gab1(S581~P) -> \
 Gab1(S581~O)	Gab1kp170

483 Gab1(S597~P) -> \
 Gab1(S597~O)	Gab1kp170

528 PIP3(C3P,two~F) -> \
 PIP3(C3P,two~T) PIP3kp3

538 Raf1(S259~P) -> \
 Raf1(S259~O)	Akt1kp10

539 Akt1(T308~P) -> \
 Akt1(T308~O)		Akt1kp10

540 Akt1(S473~P) -> \
 Akt1(S473~O)		Akt1kp10
}}}
```

```
|Protein Name |~UniProt ID |Gene Name |Comment |h
|~PTP1B	|P18031 |//~PTPN1//	|Protein tyrosine phosphatase |
|TCPTP	|P17706 |//~PTPN2//	|Protein tyrosine phosphatase |
|~SHP-1	|P29350 |//~PTPN6//	|Protein tyrosine phosphatase |
|~SHP-2	|Q06124 |//~PTPN11//	|Protein tyrosine phosphatase |
|~RPTP-?	|Q15262 |//PTPRK//	|Receptor-type tyrosine-protein phosphatase |
|~RPTP-?	|Q13332 |//PTPRS//	|Receptor-type tyrosine-protein phosphatase |
|PTEN	|P60484 |//PTEN//	|Lipid phosphatase (loss in 40% of pancreas tumors) |
|~DUSP2	|Q05923 |//~DUSP2//	|Nuclear MKP |
|~DUSP3	|P51452 |//~DUSP3//	|Atypical DUSP |
|~DUSP4	|Q13115 |//~DUSP4//	|Nuclear MKP |
|~DUSP5	|Q16690 |//~DUSP5//	|Nuclear MKP |
|~DUSP6	|Q16828 |//~DUSP6//	|Cytoplasmic MKP |
|~DUSP7	|Q16829 |//~DUSP7//	|Cytoplasmic MKP |
|~DUSP9	|Q99956 |//~DUSP9//	|Cytoplasmic MKP |
|PHLPP	|O60346 |//~PHLPP1//	|S/T phosphatase |
|~DUSP14	|O95147 |//~DUSP14//	|MKP, role in proliferation of pancreatic ? cells |
|~PP2A	|-	|-	|Heterotrimeric S/T phosphatase |
|~PR65?	|P30153 |//~PPP2R1A//	|Scaffolding subunit (subunit A) |
|~PP2Ac?	|P67775 |//~PPP2CA//	|Catalytic subunit (subunit C) |
|B55?	|P63151 |//~PPP2R2A//	|Regulatory subunit (subunit B family member) |
|B56?	|Q15173 |//~PPP2R5B// |Regulatory subunit (subunit B’ family member) |
|B56?	|Q13362 |//~PPP2R5C//	|Regulatory subunit (subunit B’ family member) |
```

```
|The model accounts for 55 sites of phosphorylation in 11 proteins; these S/T/Y sites are listed in this table |c
|Name |Numbering Note |Context |Kinase(s) |Binding Partner(s) |h
|>|>|>|>|[[EGFR | http://www.uniprot.org/uniprot/P00533]] |
|T669 |T693 in ~UniProt (+24) |{{{LQERELVEPLTPSGEAPNQAL}}} |~ERK2 |-|
|Y992 |Y1016 in ~UniProt (+24) |{{{DMDDVVDADEYLIPQQGFFSS}}} |EGFR, ~ErbB2, ~ErbB4 |p120 ~RasGAP, p52 Shc1 |
|Y1068 |Y1092 in ~UniProt (+24) |{{{IDDTFLPVPEYINQSVPKRPA}}} |EGFR, ~ErbB2, ~ErbB4 |Grb2 |
|Y1086 |Y1110 in ~UniProt (+24) |{{{RPAGSVQNPVYHNQPLNPAPS}}} |EGFR, ~ErbB2, ~ErbB4 |p52 Shc1 |
|Y1114 |Y1138 in ~UniProt (+24) |{{{PHSTAVGNPEYLNTVQPTCVN}}} |EGFR, ~ErbB2 |Grb2, p52 Shc1 |
|Y1148 |Y1172 in ~UniProt (+24) |{{{SHQISLDNPDYQQDFFPKEAK}}} |EGFR, ~ErbB2, ~ErbB4 |Grb2 |
|Y1173 |Y1197 in ~UniProt (+24) |{{{FKGSTAENAEYLRVAPQSSEF}}} |EGFR, ~ErbB2, ~ErbB4 |Grb2 |
|>|>|>|>|[[ErbB2 | http://www.uniprot.org/uniprot/P04626]] |
|Y1139 |- |{{{APLTCSPQPEYVNQPDVRPQP}}} |EGFR, ~ErbB4 |Grb2 |
|Y1196 |- |{{{AFGGAVENPEYLTPQGGAAPQ}}} |EGFR, ~ErbB4 |p52 Shc1 |
|Y1222 |- |{{{AFSPAFDNLYYWDQDPPERGA}}} |EGFR, ~ErbB4 |p52 Shc1 |
|Y1248 |- |{{{KGTPTAENPEYLGLDVPV}}} |EGFR, ~ErbB4 |p52 Shc1 |
|>|>|>|>|[[ErbB3 | http://www.uniprot.org/uniprot/P21860]] |
|Y1051 |Y1054 in ~UniProt (+3) |{{{SQSLLSPSSGYMPMNQGNLGE}}} |EGFR, ~ErbB2, ~ErbB4 |p85&alpha; |
|Y1194 |Y1197 in ~UniProt (+3) |{{{GTEEEDEDEEYEYMNRRRRHS}}} |EGFR, ~ErbB2, ~ErbB4 |p85&alpha; |
|Y1219 |Y1222 in ~UniProt (+3) |{{{PRPSSLEELGYEYMDVGSDLS}}} |EGFR, ~ErbB2, ~ErbB4 |p85&alpha; |
|Y1257 |Y1260 in ~UniProt (+3) |{{{PTAGTTPDEDYEYMNRQRDGG}}} |EGFR, ~ErbB2, ~ErbB4 |p85&alpha; |
|Y1273 |Y1276 in ~UniProt (+3) |{{{QRDGGGPGGDYAAMGACPASE}}} |EGFR, ~ErbB2, ~ErbB4 |p85&alpha; |
|Y1286 |Y1289 in ~UniProt (+3) |{{{MGACPASEQGYEEMRAFQGPG}}} |EGFR, ~ErbB2, ~ErbB4 |p85&alpha; |
|Y1325 |Y1328 in ~UniProt (+3) |{{{ATDSAFDNPDYWHSRLFPKAN}}} |EGFR, ~ErbB2, ~ErbB4 |p52 Shc1 |
|>|>|>|>|[[ErbB4 | http://www.uniprot.org/uniprot/P04626]] |
|Y1056 |- |{{{SEIGHSPPPAYTPMSGNQFVY}}} |EGFR, ~ErbB2, ~ErbB4 |p85&alpha; |
|Y1188 |- |{{{GDLQALDNPEYHNASNGPPKA}}} |EGFR, ~ErbB2, ~ErbB4 |p52 Shc1 |
|Y1242 |- |{{{KAKKAFDNPDYWNHSLPPRST}}} |EGFR, ~ErbB2, ~ErbB4 |p52 Shc1 |
|>|>|>|>|[[p52 Shc1 | http://www.uniprot.org/uniprot/P29353 ]] |
|Y317 |Y427 in p66 Shc1 |{{{PGRELFDDPSYVNVQNLDKAR}}} |EGFR, ~ErbB2, ~ErbB4 |Grb2 |
|>|>|>|>|[[Sos1 | http://www.uniprot.org/uniprot/Q07889]] |
|S1132 |- |{{{IQVTLPHGPRSASVSSISLTK}}} |~ERK2 |- |
|S1167 |- |{{{RRPESAPAESSPSKIMSKHLD}}} |~ERK2 |- |
|S1178 |- |{{{PSKIMSKHLDSPPAIPPRQPT}}} |~ERK2 |- |
|S1193 |- |{{{PPRQPTSKAYSPRYSISDRTS}}} |~ERK2 |- |
|>|>|>|>|[[Gab1 | http://www.uniprot.org/uniprot/Q13480]] |
|T312 |- |{{{HVSISYDIPPTPGNTYQIPRT}}} |~ERK2 |- |
|S381 |- |{{{SSYCIPTAGMSPSRSNTISTV}}} |~ERK2 |- |
|Y447 |- |{{{SVSSEELDENYVPMNPNSPPR}}} |EGFR, ~ErbB2, ~ErbB4 |p85&alpha; |
|S454 |- |{{{DENYVPMNPNSPPRQHSSSFT}}} |~ERK2 |- |
|Y472 |- |{{{SFTEPIQEANYVPMTPGTFDF}}} |EGFR, ~ErbB2, ~ErbB4 |p85&alpha; |
|T476 |- |{{{PIQEANYVPMTPGTFDFSSFG}}} |~ERK2 |- |
|S551 |- |{{{EWEELQAPVRSPITRSFARDS}}} |~ERK2 |- |
|S597 |- |{{{ENYVPMNPNLSSEDPNLFGSN}}} |~ERK2 |- |
|Y627 |- |{{{IKPKGDKQVEYLDLDLDSGKS}}} |EGFR, ~ErbB2, ~ErbB4 |p85&alpha; |
|Y659 |- |{{{GSSVADERVDYVVVDQQKTLA}}} |EGFR, ~ErbB2, ~ErbB4 |- |
|>|>|>|>|[[Akt1 | http://www.uniprot.org/uniprot/P31749]] |
|T308 |- |{{{EGIKDGATMKTFCGTPEYLAP}}} |~PDK1 |- |
|S473 |- |{{{SERRPHFPQFSYSASGTA}}} |- |- |
|>|>|>|>|[[Raf-1 | http://www.uniprot.org/uniprot/P04049]] |
|S29 |- |{{{DAVFDGSSCISPTIVQQFGYQ}}} |~ERK2 |- |
|S43 |- |{{{VQQFGYQRRASDDGKLTDPSK}}} |~ERK2 |- |
|S259 |- |{{{GSLSQRQRSTSTPNVHMVSTT}}} |Akt1 |- |
|S289 |- |{{{DAIRSHSESASPSALSSSPNN}}} |~ERK2 |- |
|S296 |- |{{{ESASPSALSSSPNNLSPTGWS}}} |- |- |
|S301 |- |{{{SALSSSPNNLSPTGWSQPKTP}}} |~ERK2 |- |
|S338 |- |{{{NKIRPRGQRDSSYYWEIEASE}}} |- |- |
|Y341 |- |{{{RPRGQRDSSYYWEIEASEVML}}} |- |- |
|S471 |- |{{{AKNIIHRDMKSNNIFLHEGLT}}} |~ERK2 |- |
|T491 |- |{{{TVKIGDFGLATVKSRWSGSQQ}}} |- |- |
|S494 |- |{{{IGDFGLATVKSRWSGSQQVEQ}}} |- |- |
|S642 |- |{{{EDINACTLTTSPRLPVF}}} |~ERK2 |- |
|>|>|>|>|[[MEK1 | http://www.uniprot.org/uniprot/Q02750]] |
|S218 |S222 in ~MEK2 |{{{DFGVSGQLIDSMANSFVGTRS}}} |Raf-1 |- |
|S222 |S226 in ~MEK2 |{{{SGQLIDSMANSFVGTRSYMSP}}} |Raf-1 |- |
|T292 |- |{{{DAAETPPRPRTPGRPLSSYGM}}} |~ERK2 |- |
|>|>|>|>|[[ERK2 | http://www.uniprot.org/uniprot/P28482]] |
|T185 |T202 in ~ERK1 |{{{DPDHDHTGFLTEYVATRWYRA}}} |~MEK1 |- |
|Y187 |Y204 in ~ERK1 |{{{DHDHTGFLTEYVATRWYRAPE}}} |~MEK1 |- |

The [[Phospho.ELM | http://phospho.elm.eu.org/index.html]] database is an excellent source of additional information about S/T/Y phosphorylation sites.  [[PTMScout | http://ptmscout.mit.edu/]] provides a nice interface for examining mass spectrometry (MS)-based proteomic data about post-translational modifications.
```

```
|The model accounts for the 18 proteins listed in this table|c
|Protein Name |~UniProt ID |OMIM ID |Gene Name |Comment |h
|EGF	|[[P01133 | http://www.uniprot.org/uniprot/P01133]] |[[131530 | http://www.ncbi.nlm.nih.gov/omim/131530]] |//EGF// |Growth factor |
|~HRG-? |[[Q02297 | http://www.uniprot.org/uniprot/Q02297]] |[[142445 | http://www.ncbi.nlm.nih.gov/omim/142445]] |//~NRG1// |Growth factor, isoform 1 |
|EGFR |[[P00533 | http://www.uniprot.org/uniprot/P00533]] |[[131550 | http://www.ncbi.nlm.nih.gov/omim/131550]] |//EGFR// |~ErbB-family receptor tyrosine kinase |
|~ErbB2	|[[P04626 | http://www.uniprot.org/uniprot/P04626]] |[[164870 | http://www.ncbi.nlm.nih.gov/omim/164870]] |//~ERBB2//	|~ErbB-family receptor tyrosine kinase |
|~ErbB3	|[[P21860 | http://www.uniprot.org/uniprot/P21860]] |[[190151 | http://www.ncbi.nlm.nih.gov/omim/190151]] |//~ERBB3//	|~ErbB-family receptor tyrosine kinase |
|~ErbB4	|[[Q15303 | http://www.uniprot.org/uniprot/Q15303]] |[[600543 | http://www.ncbi.nlm.nih.gov/omim/600543]] |//~ERBB4//	|~ErbB-family receptor tyrosine kinase |
|p52^^Shc1^^	|[[P29353 | http://www.uniprot.org/uniprot/P29353 ]] |[[600560 | http://www.ncbi.nlm.nih.gov/omim/600560]] |//~SHC1//	|Adapter protein - other isoforms are p46^^Shc1^^ and p66^^Shc1^^ |
|Grb2	|[[P62993 | http://www.uniprot.org/uniprot/P62993]] |[[108355 | http://www.ncbi.nlm.nih.gov/omim/108355]] |//~GRB2//	|Adapter protein |
|Sos1	|[[Q07889 | http://www.uniprot.org/uniprot/Q07889]] |[[182530 | http://www.ncbi.nlm.nih.gov/omim/182530]] |//~SOS1//	|GEF |
|Gab1	|[[Q13480 | http://www.uniprot.org/uniprot/Q13480]] |[[604439 | http://www.ncbi.nlm.nih.gov/omim/604439]] |//~GAB1//	|Adapter protein |
|~PI3K	|-	|-	|- |Phosphoinositide 3-kinase (heterodimer) |
|p85?	|[[P27986 | http://www.uniprot.org/uniprot/P27986]] |[[171833 | http://www.ncbi.nlm.nih.gov/omim/171833]] |//~PIK3R1//	|Regulatory subunit of class IA ~PI3K - other isoforms are p55? and p50? |
|p110?	|[[P42336 | http://www.uniprot.org/uniprot/P42336]] |[[171834 | http://www.ncbi.nlm.nih.gov/omim/171834]] |//~PIK3CA//	|Catalytic subunit of class IA ~PI3K - others are p110? and p110? |
|~PDK1	|[[O15530 | http://www.uniprot.org/uniprot/O15530]] |[[605213 | http://www.ncbi.nlm.nih.gov/omim/605213]] |//~PDPK1//	|S/T kinase |
|Akt1 |[[P31749 | http://www.uniprot.org/uniprot/P31749]] |[[164730 | http://www.ncbi.nlm.nih.gov/omim/164730]] |//~AKT1//	|S/T kinase (a.k.a. protein kinase B, PKB) |
|~K-Ras	|[[P01116 | http://www.uniprot.org/uniprot/P01116]] |[[190070 | http://www.ncbi.nlm.nih.gov/omim/190070]] |//KRAS//	|~GTPase (aka ~K-Ras4A, ~K-Ras4B) |
|p120^^~RasGAP^^	|[[P20936| http://www.uniprot.org/uniprot/P20936]] |[[139150 | http://www.ncbi.nlm.nih.gov/omim/139150]] |//~RASA1//	|GAP|
|Raf-1	|[[P04049 | http://www.uniprot.org/uniprot/P04049]] |[[164760 | http://www.ncbi.nlm.nih.gov/omim/164760]] |//~RAF1//	|MAPKKK|
|~MEK1	|[[Q02750 | http://www.uniprot.org/uniprot/Q02750]] |[[176872 | http://www.ncbi.nlm.nih.gov/omim/176872]] |//~MAP2K1//	|MAPKK, dual-specificity kinase (Y and S/T substrates)|
|~ERK2	|[[P28482 | http://www.uniprot.org/uniprot/P28482]] |[[176948 | http://www.ncbi.nlm.nih.gov/omim/176948]] |//~MAPK1//	|MAPK|

Many of the proteins considered in the model are found in different isoforms.  The [[Isoforms]] tiddler provides a list of some of these related proteins.

The model does not explicitly account for phosphatases.  The [[Phosphatases]] tiddler provides a list of phosphatases for future consideration.
```

```
See ''Raf-1'' in [[Proteins]]
!BNGL
{{{Raf1(RBD,STkinase,S29~O~P,S43~O~P,S259~O~P,S289~O~P,S296~O~P,S301~O~P,S338~O~P,\
Y341~O~P,S471~O~P,T491~O~P,S494~O~P,S642~O~P,loc~C)}}}
!Summary
Raf-1 is S/T kinase; it is an effector of Ras.  The Raf family is composed of ~A-Raf, ~B-Raf and Raf-1, which is also called c-Raf.  We take Raf-1 to be representative of the family.
!DOG
[img[DOGS/Raf1.jpg]]
!Reading
*[[OMIM ID 164760 | http://www.ncbi.nlm.nih.gov/omim/164760]]
*Dougherty MK, Müller J, Ritt DA, Zhou M, Zhou XZ, Copeland TD, Conrads TP, Veenstra TD, Lu KP, Morrison DK (2005) Regulation of Raf-1 by direct feedback phosphorylation. Mol Cell 17:215-224. [[PMID: 15664191 | http://www.ncbi.nlm.nih.gov/pubmed/15664191]]
```

```
''Raf1 catalyzes phosphorylation of ~MEK1'' (see Arrow 22 in [[Contact Map]])
{{{MEK1(S218~O)->MEK1(S218~P)}}}
{{{MEK1(S222~O)->MEK1(S222~P)}}}
!Rules
{{{
241 Raf1(STkinase!1,S29~O,S43~O,S259~O!?,S289~O,S296~P,S301~O,S338~P,Y341~P,S471~O,T491~P,S494~P,S642~O).MEK1(S218~O!1) -> \
 Raf1(STkinase!1,S29~O,S43~O,S259~O!?,S289~O,S296~P,S301~O,S338~P,Y341~P,S471~O,T491~P,S494~P,S642~O).MEK1(S218~P!1) MAPKkp11

242 Raf1(STkinase!1,S29~O,S43~O,S259~O!?,S289~O,S296~P,S301~O,S338~P,Y341~P,S471~O,T491~P,S494~P,S642~O).MEK1(S222~O!1) -> \
 Raf1(STkinase!1,S29~O,S43~O,S259~O!?,S289~O,S296~P,S301~O,S338~P,Y341~P,S471~O,T491~P,S494~P,S642~O).MEK1(S222~P!1) MAPKkp12
}}}
```

```
''Raf1-targeted kinase activity'' (see Arrow 20 in [[Contact Map]])
{{{Raf1(S296~O)->Raf1(S296~P)}}}
{{{Raf1(S338~O)->Raf1(S338~P)}}}
{{{Raf1(Y341~O)->Raf1(Y341~P)}}}
{{{Raf1(T491~O)->Raf1(T491~P)}}}
{{{Raf1(S494~O)->Raf1(S494~P)}}}
!Rules
{{{
228 KRas(GTPase!1,g~GTP).Raf1(RBD!1,S296~O) -> \
 KRas(GTPase!1,g~GTP).Raf1(RBD!1,S296~P)          MAPKkp2

229 KRas(GTPase!1,g~GTP).Raf1(RBD!1,S338~O) -> \
 KRas(GTPase!1,g~GTP).Raf1(RBD!1,S338~P)          MAPKkp3

230 KRas(GTPase!1,g~GTP).Raf1(RBD!1,Y341~O) -> \
 KRas(GTPase!1,g~GTP).Raf1(RBD!1,Y341~P)          MAPKkp4

231 KRas(GTPase!1,g~GTP).Raf1(RBD!1,T491~O) -> \
 KRas(GTPase!1,g~GTP).Raf1(RBD!1,T491~P)          MAPKkp5

232 KRas(GTPase!1,g~GTP).Raf1(RBD!1,S494~O) -> \
 KRas(GTPase!1,g~GTP).Raf1(RBD!1,S494~P)          MAPKkp6
}}}
```

```
''Endocytosis, ligand degradation, and receptor recycling'' (see location tags in [[Contact Map]])
!Rules
{{{
53 EGFR(I_III!1,II!3,loc~M).EGF(EGFL!1,loc~Ex).EGFR(I_III!2,II!3,loc~M).EGF(EGFL!2,loc~Ex) -> \
 EGFR(I_III!1,II!3,loc~En).EGF(EGFL!1,loc~En).EGFR(I_III!2,II!3,loc~En).EGF(EGFL!2,loc~En)	Intkp1

54 EGFR(I_III!1,II!2,loc~M).EGF(EGFL!1,loc~Ex).EGFR(I_III,II!2,loc~M) -> \
 EGFR(I_III!1,II!2,loc~En).EGF(EGFL!1,loc~En).EGFR(I_III,II!2,loc~En)	Intkp1

55 EGFR(I_III,II!2,loc~M).EGFR(I_III,II!2,loc~M) -> \
 EGFR(I_III,II!2,loc~En).EGFR(I_III,II!2,loc~En)	Intkp1

56 EGFR(I_III!1,II!2,loc~M).EGF(EGFL!1,loc~Ex).ErbB2(II!2,loc~M) -> \
 EGFR(I_III!1,II!2,loc~En).EGF(EGFL!1,loc~En).ErbB2(II!2,loc~En)	Intkp1

57 EGFR(I_III,II!2,loc~M).ErbB2(II!2,loc~M) -> \
 EGFR(I_III,II!2,loc~En).ErbB2(II!2,loc~En)	Intkp1

58 EGFR(I_III!1,II!3,loc~M).EGF(EGFL!1,loc~Ex).ErbB3(I_III!2,II!3,loc~M).HRG(EGFL!2,loc~Ex) -> \
 EGFR(I_III!1,II!3,loc~En).EGF(EGFL!1,loc~En).ErbB3(I_III!2,II!3,loc~En).HRG(EGFL!2,loc~En)	Intkp1

59 EGFR(I_III,II!3,loc~M).ErbB3(I_III!2,II!3,loc~M).HRG(EGFL!2,loc~Ex) -> \
 EGFR(I_III,II!3,loc~En).ErbB3(I_III!2,II!3,loc~En).HRG(EGFL!2,loc~En)	Intkp1

60 EGFR(I_III!1,II!3,loc~M).EGF(EGFL!1,loc~Ex).ErbB3(I_III,II!3,loc~M) -> \
 EGFR(I_III!1,II!3,loc~En).EGF(EGFL!1,loc~En).ErbB3(I_III,II!3,loc~En)	Intkp1

61 EGFR(I_III,II!3,loc~M).ErbB3(I_III,II!3,loc~M) -> \
 EGFR(I_III,II!3,loc~En).ErbB3(I_III,II!3,loc~En)	Intkp1

62 EGFR(I_III!1,II!3,loc~M).EGF(EGFL!1,loc~Ex).ErbB4(I_III!2,II!3,loc~M).HRG(EGFL!2,loc~Ex) -> \
 EGFR(I_III!1,II!3,loc~En).EGF(EGFL!1,loc~En).ErbB4(I_III!2,II!3,loc~En).HRG(EGFL!2,loc~En)	Intkp1

63 EGFR(I_III,II!3,loc~M).ErbB4(I_III!2,II!3,loc~M).HRG(EGFL!2,loc~Ex) -> \
 EGFR(I_III,II!3,loc~En).ErbB4(I_III!2,II!3,loc~En).HRG(EGFL!2,loc~En)	Intkp1

64 EGFR(I_III!1,II!3,loc~M).EGF(EGFL!1,loc~Ex).ErbB4(I_III,II!3,loc~M) -> \
 EGFR(I_III!1,II!3,loc~En).EGF(EGFL!1,loc~En).ErbB4(I_III,II!3,loc~En)	Intkp1

65 EGFR(I_III,II!3,loc~M).ErbB4(I_III,II!3,loc~M) -> \
 EGFR(I_III,II!3,loc~En).ErbB4(I_III,II!3,loc~En)	Intkp1

66 ErbB2(II!1,loc~M).ErbB2(II!1,loc~M) -> \
 ErbB2(II!1,loc~En).ErbB2(II!1,loc~En) Intkp1

67 ErbB3(I_III!1,II!2,loc~M).HRG(EGFL!1,loc~Ex).ErbB2(II!2,loc~M) -> \
 ErbB3(I_III!1,II!2,loc~En).HRG(EGFL!1,loc~En).ErbB2(II!2,loc~En)	Intkp1

68 ErbB3(I_III,II!2,loc~M).ErbB2(II!2,loc~M) -> \
 ErbB3(I_III,II!2,loc~En).ErbB2(II!2,loc~En)	Intkp1

69 ErbB4(I_III!1,II!2,loc~M).HRG(EGFL!1,loc~Ex).ErbB2(II!2,loc~M) -> \
 ErbB4(I_III!1,II!2,loc~En).HRG(EGFL!1,loc~En).ErbB2(II!2,loc~En)	Intkp1

70 ErbB4(I_III,II!2,loc~M).ErbB2(II!2,loc~M) -> \
 ErbB4(I_III,II!2,loc~En).ErbB2(II!2,loc~En)	Intkp1

71 ErbB3(I_III!1,II!2,loc~M).HRG(EGFL!1,loc~Ex).ErbB3(I_III!3,II!2,loc~M).HRG(EGFL!3,loc~Ex) -> \
 ErbB3(I_III!1,II!2,loc~En).HRG(EGFL!1,loc~En).ErbB3(I_III!3,II!2,loc~En).HRG(EGFL!3,loc~En)	Intkp1

72 ErbB3(I_III!1,II!2,loc~M).HRG(EGFL!1,loc~Ex).ErbB3(I_III,II!2,loc~M) -> \
 ErbB3(I_III!1,II!2,loc~En).HRG(EGFL!1,loc~En).ErbB3(I_III,II!2,loc~En)	Intkp1

73 ErbB3(I_III,II!2,loc~M).ErbB3(I_III,II!2,loc~M) -> \
 ErbB3(I_III,II!2,loc~En).ErbB3(I_III,II!2,loc~En)	Intkp1

74 ErbB3(I_III!1,II!2,loc~M).HRG(EGFL!1,loc~Ex).ErbB4(I_III!3,II!2,loc~M).HRG(EGFL!3,loc~Ex) -> \
 ErbB3(I_III!1,II!2,loc~En).HRG(EGFL!1,loc~En).ErbB4(I_III!3,II!2,loc~En).HRG(EGFL!3,loc~En)	Intkp1

75 ErbB3(I_III!1,II!2,loc~M).HRG(EGFL!1,loc~Ex).ErbB4(I_III,II!2,loc~M) -> \
 ErbB3(I_III!1,II!2,loc~En).HRG(EGFL!1,loc~En).ErbB4(I_III,II!2,loc~En)	Intkp1

76 ErbB3(I_III,II!2,loc~M).ErbB4(I_III!1,II!2,loc~M).HRG(EGFL!1,loc~Ex) -> \
 ErbB3(I_III,II!2,loc~En).ErbB4(I_III!1,II!2,loc~En).HRG(EGFL!1,loc~En)	Intkp1

77 ErbB3(I_III,II!2,loc~M).ErbB4(I_III,II!2,loc~M) -> \
 ErbB3(I_III,II!2,loc~En).ErbB4(I_III,II!2,loc~En)	Intkp1

78 ErbB4(I_III!1,II!2,loc~M).HRG(EGFL!1,loc~Ex).ErbB4(I_III!3,II!2,loc~M).HRG(EGFL!3,loc~Ex) -> \
 ErbB4(I_III!1,II!2,loc~En).HRG(EGFL!1,loc~En).ErbB4(I_III!3,II!2,loc~En).HRG(EGFL!3,loc~En)	Intkp1

79 ErbB4(I_III!1,II!2,loc~M).HRG(EGFL!1,loc~Ex).ErbB4(I_III,II!2,loc~M) -> \
 ErbB4(I_III!1,II!2,loc~En).HRG(EGFL!1,loc~En).ErbB4(I_III,II!2,loc~En)	Intkp1

80 ErbB4(I_III,II!2,loc~M).ErbB4(I_III,II!2,loc~M) -> \
 ErbB4(I_III,II!2,loc~En).ErbB4(I_III,II!2,loc~En)	Intkp1

81 EGFR(I_III!1,loc~En).EGF(EGFL!1,deg~F,loc~En) -> \
 EGFR(I_III,loc~En) + EGF(EGFL,deg~F,loc~En)	iLigkp1

82 ErbB3(I_III!1,loc~En).HRG(EGFL!1,deg~F,loc~En) -> \
 ErbB3(I_III,loc~En) + HRG(EGFL,deg~F,loc~En)	iLigkp2

83 ErbB4(I_III!1,loc~En).HRG(EGFL!1,deg~F,loc~En) -> \
 ErbB4(I_III,loc~En) + HRG(EGFL,deg~F,loc~En)	iLigkp3

84 EGF(EGFL,deg~F,loc~En) -> \
 EGF(EGFL,deg~T,loc~En)	iLigkp4

85 HRG(EGFL,deg~F,loc~En) -> \
 HRG(EGFL,deg~T,loc~En)	iLigkp4

86 EGFR(II!3,loc~En).EGFR(II!3,loc~En) -> \
 EGFR(II,loc~En) + EGFR(II,loc~En)		iDimkp1

87 EGFR(II!2,loc~En).ErbB2(II!2,loc~En) -> \
 EGFR(II,loc~En) + ErbB2(II,loc~En)	iDimkp2

88 EGFR(II!3,loc~En).ErbB3(II!3,loc~En) -> \
 EGFR(II,loc~En) + ErbB3(II,loc~En)	iDimkp3

89 EGFR(II!3,loc~En).ErbB4(II!3,loc~En) -> \
 EGFR(II,loc~En) + ErbB4(II,loc~En)	iDimkp4

90 ErbB2(II!2,loc~En).ErbB2(II!2,loc~En) -> \
 ErbB2(II,loc~En) + ErbB2(II,loc~En)	iDimkp5

91 ErbB2(II!2,loc~En).ErbB3(II!2,loc~En) -> \
 ErbB2(II,loc~En) + ErbB3(II,loc~En)	iDimkp6

92 ErbB2(II!2,loc~En).ErbB4(II!2,loc~En) -> \
 ErbB2(II,loc~En) + ErbB4(II,loc~En)	iDimkp7

93 ErbB3(II!2,loc~En).ErbB3(II!2,loc~En) -> \
 ErbB3(II,loc~En) + ErbB3(II,loc~En)	iDimkp8

94 ErbB3(II!2,loc~En).ErbB4(II!2,loc~En) -> \
 ErbB3(II,loc~En) + ErbB4(II,loc~En)	iDimkp9

95 ErbB4(II!2,loc~En).ErbB4(II!2,loc~En) -> \
 ErbB4(II,loc~En) + ErbB4(II,loc~En)	iDimkp10

96 EGFR(I_III,II,loc~En) -> \
 EGFR(I_III,II,loc~M)	Intkp15

97 ErbB2(II,loc~En) -> \
 ErbB2(II,loc~M)	Intkp15

98 ErbB3(I_III,II,loc~En) -> \
 ErbB3(I_III,II,loc~M)	Intkp15

99 ErbB4(I_III,II,loc~En) -> \
 ErbB4(I_III,II,loc~M)	Intkp15
}}}
```

```
annotation of a rule-based model for ~ErbB-mediated activation of ERK and AKT
```

```
ModelGuideWiki
```

```
See ''Sos1 (son of sevenless homolog 1)'' in [[Proteins]]
!BNGL
{{{Sos1(PRS,REM,GEF,S1132~O~P,S1167~O~P,S1178~O~P,S1193~O~P,loc~C)}}}
!Summary
Sos1 (represented by {{{Sos1}}} in the model) is a guanine nucleotide exchange factor (GEF).  It contains a ~RasGEF domain, which is also called a ~CDC25 homology domain, and a Ras exchange motif (REM), which binds ~GDP- and ~GTP-loaded Ras.  Sos1 contains a C-terminal proline-rich region (PRR), where several proline-rich sequences (~PRSs) are present.  These sequences are matched by the regular expression PP.{1,2}PPR, and they interact with the N-terminal ~SH3 domain of Grb2. In the PRR, there are four serine residues (S1132, S1167, S1178 and S1193), which are substrates of ~ERK1/2.  Phosphorylation of the serine residues in the PRR of Sos1 interferes in the ~Grb2-Sos1 interaction.
!DOG
[img[DOGS/Sos1.jpg]]
!Reading
*[[OMIM ID 182530 | http://www.ncbi.nlm.nih.gov/omim/182530]]
*Bos JL, Rehmann H, Wittinghofer A (2007) ~GEFs and ~GAPs: critical elements in the control of small G proteins. Cell 129:865-877. [[PMID: 17540168 | http://www.ncbi.nlm.nih.gov/pubmed/17540168]]
*Bernards A, Settleman J (2007) ~GEFs in growth factor signaling. Growth Factors 25:355-361. [[PMID: 18236214 | http://www.ncbi.nlm.nih.gov/pubmed/18236214]]
```

```
''Guanine nucleotide exchange activity of Sos1'' (see Arrow 17 in [[Contact Map]])
{{{KRas(g~GDP)->KRas(g~GTP)}}}
!Rules
{{{
222 Sos1(REM,GEF!3,S1132~O,S1167~O,S1178~O,S1193~O).KRas(GTPase!3,g~GDP) <-> \
 Sos1(REM,GEF!3,S1132~O,S1167~O,S1178~O,S1193~O).KRas(GTPase!3,g~GTP)     Sos1kp5,Sos1km5

223 Sos1(REM!+,GEF!3,S1132~O,S1167~O,S1178~O,S1193~O).KRas(GTPase!3,g~GDP) <-> \
 Sos1(REM!+,GEF!3,S1132~O,S1167~O,S1178~O,S1193~O).KRas(GTPase!3,g~GTP)     Sos1kp6,Sos1km6
}}}
```

```
.nestedsliderbutton{
border: 1px solid #aaa;
}
```

```
/***
|''Name:''|TagsTreePlugin|
|''Description:''|Displays tags hierachy as a tree of tagged tiddlers.<br>Can be used to create dynamic outline navigation.|
|''Version:''|1.0.1|
|''Date:''|Jan 04,2008|
|''Source:''|http://visualtw.ouvaton.org/VisualTW.html|
|''Author:''|Pascal Collin|
|''License:''|[[BSD open source license|License]]|
|''~CoreVersion:''|2.1.0|
|''Browser:''|Firefox 2.0; InternetExplorer 6.0|
!Demo
On the plugin [[homepage|http://visualtw.ouvaton.org/VisualTW.html]] :
*Try to tag some <<newTiddler>> with a tag displayed in the menu and edit MainMenu.
*Look at some tags like [[Plugins]] or [[menu]].
!Installation
#import the plugin,
#save and reload,
#optionally, edit TagsTreeStyleSheet.
! Usage
{{{<<tagsTree>>}}} macro accepts the following //optional// parameters.
|!#|!parameter|!description|!by default|
|1|{{{root}}}|Uses {{{root}}} tag as tree root|- In a //tiddler// content or template : uses the tiddler as root tag.<br>- In the //page// content or template (by ex MainMenu) : displays all untagged tags.|
|2|{{{excludeTag}}}|Excludes all such tagged tiddlers from the tree|Uses default excludeLists tag|
|3|{{{level}}}|Expands nodes until level {{{level}}}.<br>Value {{{0}}} hides expand/collapse buttons.|Nodes are collapsed on first level|
|4|{{{depth}}}|Hierachy depth|6 levels depth (H1 to H6 header styles)|
|5|{{{sortField}}}|Alternate sort field. By example : "index".|Sorts tags and tiddlers alphabetically (on their title)|
|6|{{{labelField}}}|Alertnate label field. By example : "label".|Displays tiddler's title|

!Useful addons
*[[FieldsEditorPlugin]] : //create//, //edit//, //view// and //delete// commands in toolbar <<toolbar fields>>.
*[[TaggerPlugin]] : Provides a drop down listing current tiddler tags, and allowing toggling of tags.
!Advanced Users
You can change the global defaults for TagsTreePlugin, like default {{{level}}} value or level styles, by editing or overriding the first config.macros.tagsTree attributes below.
!Code
***/
//{{{
config.macros.tagsTree = {
	expand : "+",
	collapse : "–",
	depth : 6,
	level : 1,
	sortField : "",
	labelField : "",
	styles : ["h1","h2","h3","h4","h5","h6"],
	trees : {}
}

config.macros.tagsTree.handler = function(place,macroName,params,wikifier,paramString,tiddler)
{
	var root = params[0] ? params[0] : (tiddler ? tiddler.title : null);
	var excludeTag = params[1] ? params[1] : "excludeTagsTree";
	var level = params[2] ? params[2] : config.macros.tagsTree.level;
	var depth = params[3] ? params[3] : config.macros.tagsTree.depth;
	var sortField = params[4] ? params[4] : config.macros.tagsTree.sortField;
	var labelField = params[5] ? params[5] : config.macros.tagsTree.labelField;
	var showButtons = (level>0);
	var id = config.macros.tagsTree.getId(place);
	if (config.macros.tagsTree.trees[id]==undefined) config.macros.tagsTree.trees[id]={};
	config.macros.tagsTree.createSubTree(place,id,root,excludeTag,[],level>0 ? level : 1,depth, sortField, labelField,showButtons);
}

config.macros.tagsTree.createSubTree = function(place, id, root, excludeTag, ancestors, level, depth, sortField, labelField,showButtons){
	var childNodes = root ? this.getChildNodes(root, ancestors) : this.getRootTags(excludeTag);
	var isOpen = (level>0) || (!showButtons);
	if (root && this.trees[id][root]!=undefined) isOpen = this.trees[id][root];
	if (root && ancestors.length) {
		var t = store.getTiddler(root);
		if (childNodes.length && depth>0) {
			var wrapper = createTiddlyElement(place , this.styles[Math.min(Math.max(ancestors.length,1),6)-1],null,"branch");
			if (showButtons) {
				b = createTiddlyButton(wrapper, isOpen ? config.macros.tagsTree.collapse : config.macros.tagsTree.expand, null, config.macros.tagsTree.onClick);
				b.setAttribute("treeId",id);
				b.setAttribute("tiddler",root);
			}
			createTiddlyText(createTiddlyLink(wrapper, root),t&&labelField ? t.fields[labelField] ? t.fields[labelField] : root : root);
		}
		else
			createTiddlyText(createTiddlyLink(place, root,false,"leaf"),t&&labelField ? t.fields[labelField] ? t.fields[labelField] : root : root);
	}
	if (childNodes.length && depth) {
		var d = createTiddlyElement(place,"div",null,"subtree");
		d.style.display= isOpen ? "block" : "none";
		if (sortField)
			childNodes.sort(function(a, b){
				var fa=a.fields[sortField];
				var fb=b.fields[sortField];
				return (fa==undefined && fb==undefined) ? a.title < b.title ? -1 : a.title > b.title ? 1 : 0 : (fa==undefined && fb!=undefined) ? 1 :(fa!=undefined && fb==undefined) ? -1 : fa < fb ? -1 : fa > fb ? 1 : 0;
			})
		for (var cpt=0; cpt<childNodes.length; cpt++)
			this.createSubTree(d, id, childNodes[cpt].title, excludeTag, ancestors.concat(root), level-1, depth-1, sortField, labelField, showButtons);
	}
}

config.macros.tagsTree.onClick = function(e){
	var id = this.getAttribute("treeId");
	var tiddler = this.getAttribute("tiddler");
	var n = this.parentNode.nextSibling;
	var isOpen = n.style.display != "none";
	if(config.options.chkAnimate && anim && typeof Slider == "function")
		anim.startAnimating(new Slider(n,!isOpen,null,"none"));
	else
		n.style.display = isOpen ? "none" : "block";
	this.firstChild.nodeValue = isOpen ? config.macros.tagsTree.expand : config.macros.tagsTree.collapse;
	config.macros.tagsTree.trees[id][tiddler]=!isOpen;
	return false;
}

config.macros.tagsTree.getChildNodes = function(root ,ancestors){
	var childs = store.getTaggedTiddlers(root);
	var result = new Array();
	for (var cpt=0; cpt<childs.length; cpt++)
		if (childs[cpt].title!=root && ancestors.indexOf(childs[cpt].title)==-1) result.push(childs[cpt]);
	return result;
}

config.macros.tagsTree.getRootTags = function(excludeTag){
	var tags = store.getTags(excludeTag);
	tags.sort(function(a,b) {return a[0].toLowerCase() < b[0].toLowerCase() ? -1 : (a[0].toLowerCase() == b[0].toLowerCase() ? 0 : +1);});
	var result = new Array();
	for (var cpt=0; cpt<tags.length; cpt++) {
		var t = store.getTiddler(tags[cpt][0]);
		if (!t || t.tags.length==0) result.push(t ? t : {title:tags[cpt][0],fields:{}});
	}
	return result;
}

config.macros.tagsTree.getId = function(element){
	while (!element.id && element.parentNode) element=element.parentNode;
	return element.id ? element.id : "<html>";
}

config.shadowTiddlers.TagsTreeStyleSheet = "/*{{{*/\n";
config.shadowTiddlers.TagsTreeStyleSheet +=".leaf, .subtree {display:block; margin-left : 0.5em}\n";
config.shadowTiddlers.TagsTreeStyleSheet +=".subtree {margin-bottom:0.5em}\n";
config.shadowTiddlers.TagsTreeStyleSheet +="#mainMenu {text-align:left}\n";
config.shadowTiddlers.TagsTreeStyleSheet +=".branch .button {border:1px solid #DDD; color:#AAA;font-size:9px;padding:0 2px;margin-right:0.3em;vertical-align:middle;text-align:center;}\n";
config.shadowTiddlers.TagsTreeStyleSheet +="/*}}}*/";

store.addNotification("TagsTreeStyleSheet", refreshStyles);

config.shadowTiddlers.MainMenu="<<tagsTree>>"

config.shadowTiddlers.PageTemplate = config.shadowTiddlers.PageTemplate.replace(/id='mainMenu' refresh='content' /,"id='mainMenu' refresh='content' force='true' ")

//}}}
```

```
To edit the main menu (at left), go to the [[MainMenu]] tiddler.

If you wish to make edits, you should enter a username in the [[GettingStarted]] tiddler so that your edits are signed.

''It may be convenient to have only one tiddler open at a time.  If so, close a tiddler (by clicking on `close' at top right) before opening another tiddler.''

Here's a menu heading with a custom style:
See [[StyleSheet]]
+++{{nestedsliderbutton{[test]}}}
something
[[Domains]]
===

Here's an example of a nested slider:
+++[Intro >>][Intro]>
   [[Compartments]]
   [[Domains]]
   +++[+ Interactions][- Interactions]>
      [[InteractionsPart1]]
      [[InteractionsPart2]]
      ...
   ===
   +++[+ SubMenu2][- SubMenu2]>
      item
      item
      ...
   ===
===

<<tagsTree Menu "" 1 4>>
```

```
See ''p120^^~RasGAP^^'' in [[Proteins]]
!BNGL
{{{p120RasGAP(nSH2,GAP)}}}
!Summary
p120^^~RasGAP^^ (represented by {{{p120RasGAP}}} in the model) is a Ras ~GTPase activating protein (GAP).  The component {{{nSH2}}} represents the N-terminal ~SH2 domain of p120^^~RasGAP^^.
!DOG
[img[DOGS/p120RasGAP.jpg]]
!Reading
*[[OMIM ID 139150 | http://www.ncbi.nlm.nih.gov/omim/139150]]
*Pamonsinlapatham P, ~Hadj-Slimane R, Lepelletier Y, Allain B, toccafondi M, Garbay C, Raynaud F (2009) ~P120-Ras ~GTPase activating protein (~RasGAP): A multi-interacting protein in downstream signaling. Biochimie 91:320-328. [[PMID: 19022332 | http://www.ncbi.nlm.nih.gov/pubmed/19022332]]
```

```
''~GTPase activity of p120RasGAP:~KRas complex'' (see Arrow 13 in [[Contact Map]])
{{{KRas(g~GTP)->KRas(g~GDP)}}}
!Rules
{{{
542 p120RasGAP(nSH2!+,GAP) + KRas(GTPase,g~GTP) -> \
 p120RasGAP(nSH2!+,GAP!1).KRas(GTPase!1,g~GTP)	rGAPkp2

543 p120RasGAP(GAP!1).KRas(GTPase!1,g~GTP) -> \
 p120RasGAP(GAP!1).KRas(GTPase!1,g~GDP)	rGAPkp3

544 p120RasGAP(GAP!1).KRas(GTPase!1) -> \
 p120RasGAP(GAP) + KRas(GTPase)	rGAPkp4
}}}
```

```
See ''p52^^Shc1^^ (~SH2 domain-containing-transforming protein C1)'' in [[Proteins]]
!BNGL
{{{p52Shc1(PTB,Y317~O~P,loc~C)}}}
!Summary
Shc1 is an adapter protein.  There are three isoforms of Shc1 generated through alternative splicing (p46, p52 and p66).  We take p52^^Shc1^^ to be representative of the three isoforms.  The residue Y317 is a substrate of EGFR.  Shc1 can interact with phosphotyrosines in ~ErbB family members via its phosphotyrosine binding (PTB) domain and via its Src homology 2 (~SH2) domain.  Only the PTB domain is considered in the model.

!DOG
[img[DOGS/p52Shc1.jpg]]
!Reading
*[[OMIM ID 600560 | http://www.ncbi.nlm.nih.gov/omim/600560]]
*Ravichandran KS (2001) Signaling via Shc family adapter proteins. Oncogene 20:6322-6330. [[PMID: 11607835 | http://www.ncbi.nlm.nih.gov/pubmed/11607835]]
```
