# Supplementary figures and images for: Specification, annotation, visualization and simulation of a large rule-based model for ERBB receptor signaling
Source: BMC Syst Biol. 2012 Aug 22;6:107. doi: 10.1186/1752-0509-6-107 (PMC3485121; doi:10.1186/1752-0509-6-107)

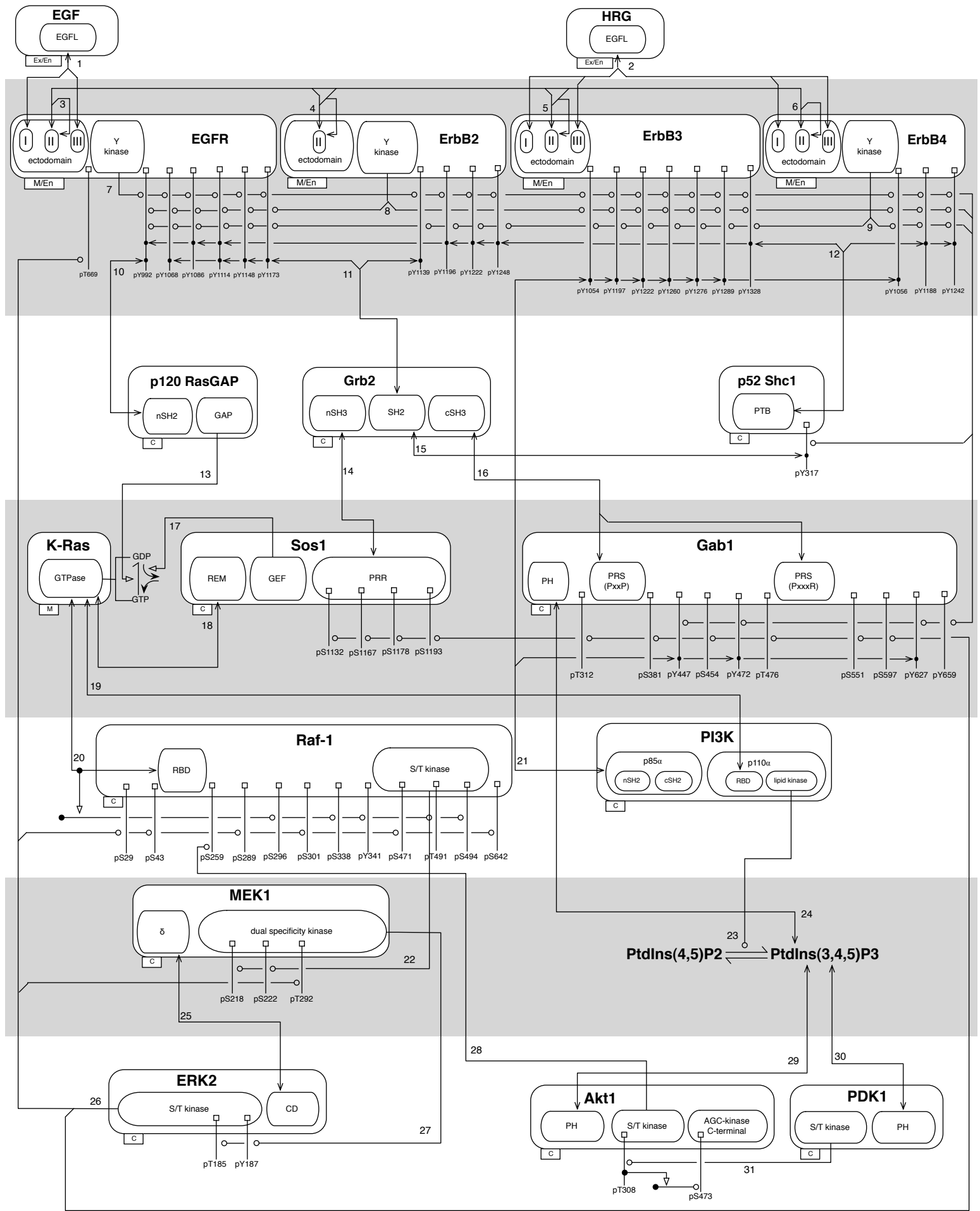

Supplement: Additional file 2 — ModelGuideWiki.zip. This archive file provides a copy of the files available online (https://modeling.tgen.org). These files serve to annotate the model. (ZIP 759 kb) [file 1752-0509-6-107-S2.zip › ModelGuideWiki/ContactMap.pdf]

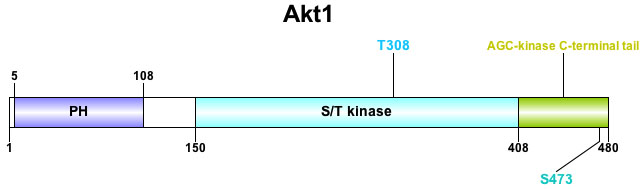

Supplement: Additional file 2 — ModelGuideWiki.zip. This archive file provides a copy of the files available online (https://modeling.tgen.org). These files serve to annotate the model. (ZIP 759 kb) [file 1752-0509-6-107-S2.zip › ModelGuideWiki/DOGS/Akt1.jpg]

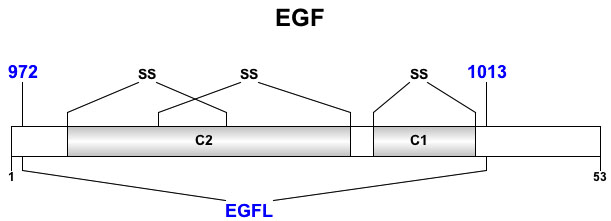

Supplement: Additional file 2 — ModelGuideWiki.zip. This archive file provides a copy of the files available online (https://modeling.tgen.org). These files serve to annotate the model. (ZIP 759 kb) [file 1752-0509-6-107-S2.zip › ModelGuideWiki/DOGS/EGF.jpg]

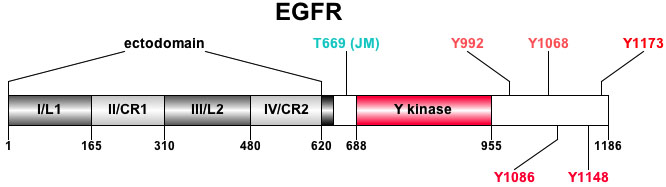

Supplement: Additional file 2 — ModelGuideWiki.zip. This archive file provides a copy of the files available online (https://modeling.tgen.org). These files serve to annotate the model. (ZIP 759 kb) [file 1752-0509-6-107-S2.zip › ModelGuideWiki/DOGS/EGFR.jpg]

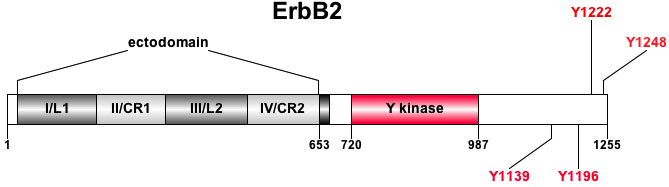

Supplement: Additional file 2 — ModelGuideWiki.zip. This archive file provides a copy of the files available online (https://modeling.tgen.org). These files serve to annotate the model. (ZIP 759 kb) [file 1752-0509-6-107-S2.zip › ModelGuideWiki/DOGS/ErbB2.jpg]

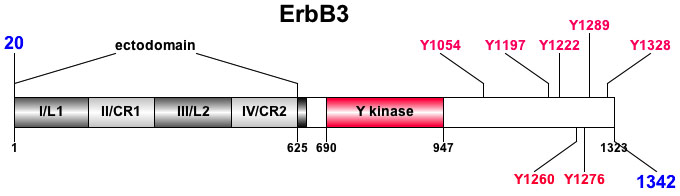

Supplement: Additional file 2 — ModelGuideWiki.zip. This archive file provides a copy of the files available online (https://modeling.tgen.org). These files serve to annotate the model. (ZIP 759 kb) [file 1752-0509-6-107-S2.zip › ModelGuideWiki/DOGS/ErbB3.jpg]

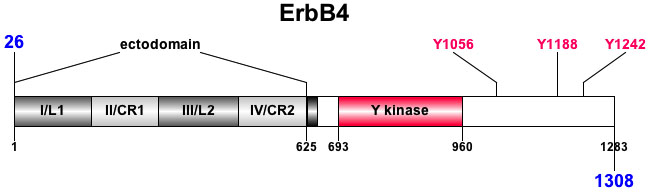

Supplement: Additional file 2 — ModelGuideWiki.zip. This archive file provides a copy of the files available online (https://modeling.tgen.org). These files serve to annotate the model. (ZIP 759 kb) [file 1752-0509-6-107-S2.zip › ModelGuideWiki/DOGS/ErbB4.jpg]

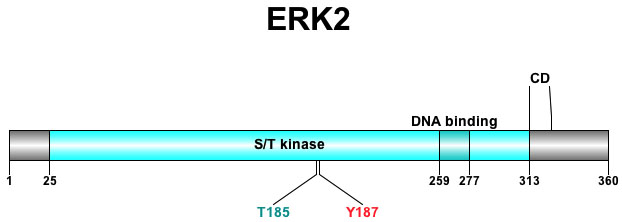

Supplement: Additional file 2 — ModelGuideWiki.zip. This archive file provides a copy of the files available online (https://modeling.tgen.org). These files serve to annotate the model. (ZIP 759 kb) [file 1752-0509-6-107-S2.zip › ModelGuideWiki/DOGS/ERK2.jpg]

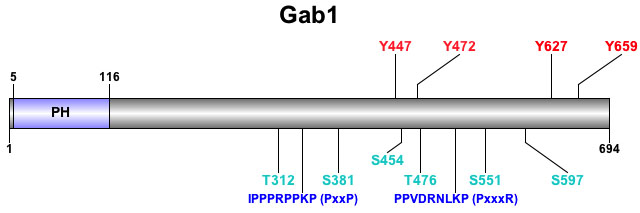

Supplement: Additional file 2 — ModelGuideWiki.zip. This archive file provides a copy of the files available online (https://modeling.tgen.org). These files serve to annotate the model. (ZIP 759 kb) [file 1752-0509-6-107-S2.zip › ModelGuideWiki/DOGS/Gab1.jpg]

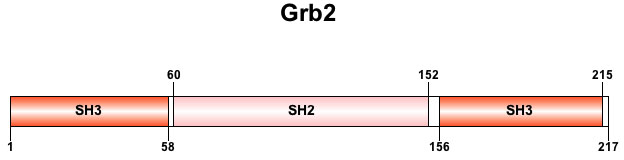

Supplement: Additional file 2 — ModelGuideWiki.zip. This archive file provides a copy of the files available online (https://modeling.tgen.org). These files serve to annotate the model. (ZIP 759 kb) [file 1752-0509-6-107-S2.zip › ModelGuideWiki/DOGS/Grb2.jpg]

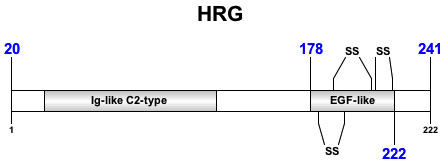

Supplement: Additional file 2 — ModelGuideWiki.zip. This archive file provides a copy of the files available online (https://modeling.tgen.org). These files serve to annotate the model. (ZIP 759 kb) [file 1752-0509-6-107-S2.zip › ModelGuideWiki/DOGS/HRG.jpg]

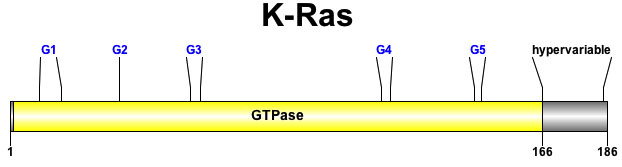

Supplement: Additional file 2 — ModelGuideWiki.zip. This archive file provides a copy of the files available online (https://modeling.tgen.org). These files serve to annotate the model. (ZIP 759 kb) [file 1752-0509-6-107-S2.zip › ModelGuideWiki/DOGS/KRas.jpg]

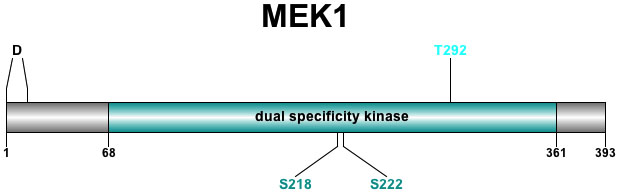

Supplement: Additional file 2 — ModelGuideWiki.zip. This archive file provides a copy of the files available online (https://modeling.tgen.org). These files serve to annotate the model. (ZIP 759 kb) [file 1752-0509-6-107-S2.zip › ModelGuideWiki/DOGS/MEK1.jpg]

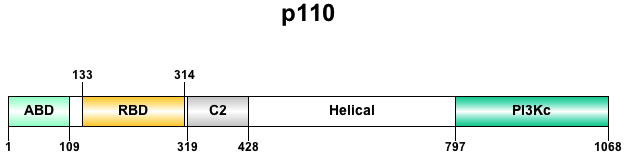

Supplement: Additional file 2 — ModelGuideWiki.zip. This archive file provides a copy of the files available online (https://modeling.tgen.org). These files serve to annotate the model. (ZIP 759 kb) [file 1752-0509-6-107-S2.zip › ModelGuideWiki/DOGS/p110alpha.jpg]

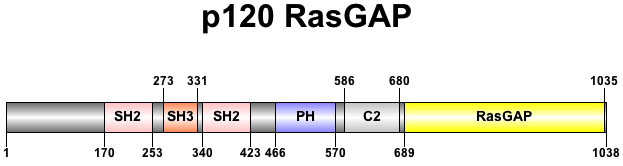

Supplement: Additional file 2 — ModelGuideWiki.zip. This archive file provides a copy of the files available online (https://modeling.tgen.org). These files serve to annotate the model. (ZIP 759 kb) [file 1752-0509-6-107-S2.zip › ModelGuideWiki/DOGS/p120RasGAP.jpg]

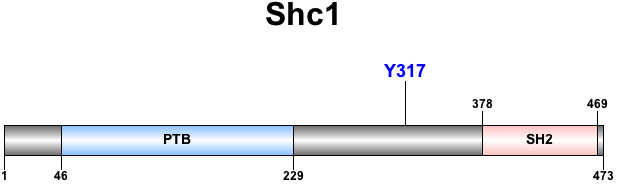

Supplement: Additional file 2 — ModelGuideWiki.zip. This archive file provides a copy of the files available online (https://modeling.tgen.org). These files serve to annotate the model. (ZIP 759 kb) [file 1752-0509-6-107-S2.zip › ModelGuideWiki/DOGS/p52Shc1.jpg]

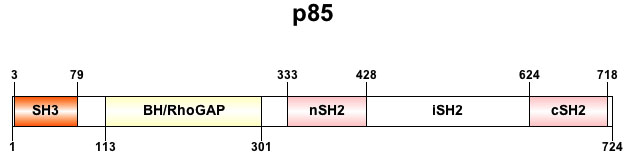

Supplement: Additional file 2 — ModelGuideWiki.zip. This archive file provides a copy of the files available online (https://modeling.tgen.org). These files serve to annotate the model. (ZIP 759 kb) [file 1752-0509-6-107-S2.zip › ModelGuideWiki/DOGS/p85alpha.jpg]

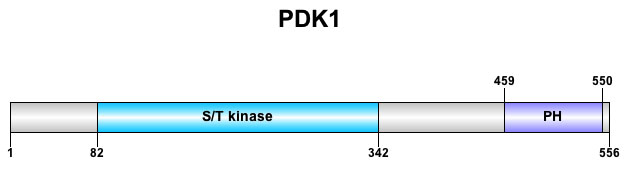

Supplement: Additional file 2 — ModelGuideWiki.zip. This archive file provides a copy of the files available online (https://modeling.tgen.org). These files serve to annotate the model. (ZIP 759 kb) [file 1752-0509-6-107-S2.zip › ModelGuideWiki/DOGS/PDK1.jpg]

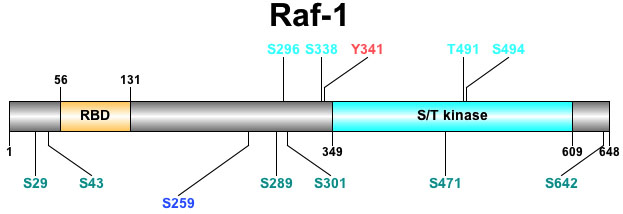

Supplement: Additional file 2 — ModelGuideWiki.zip. This archive file provides a copy of the files available online (https://modeling.tgen.org). These files serve to annotate the model. (ZIP 759 kb) [file 1752-0509-6-107-S2.zip › ModelGuideWiki/DOGS/Raf1.jpg]

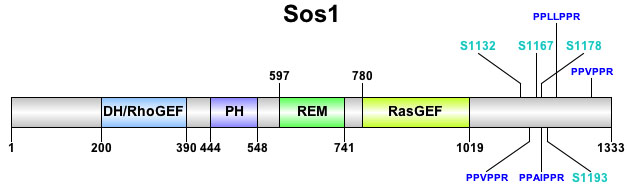

Supplement: Additional file 2 — ModelGuideWiki.zip. This archive file provides a copy of the files available online (https://modeling.tgen.org). These files serve to annotate the model. (ZIP 759 kb) [file 1752-0509-6-107-S2.zip › ModelGuideWiki/DOGS/Sos1.jpg]

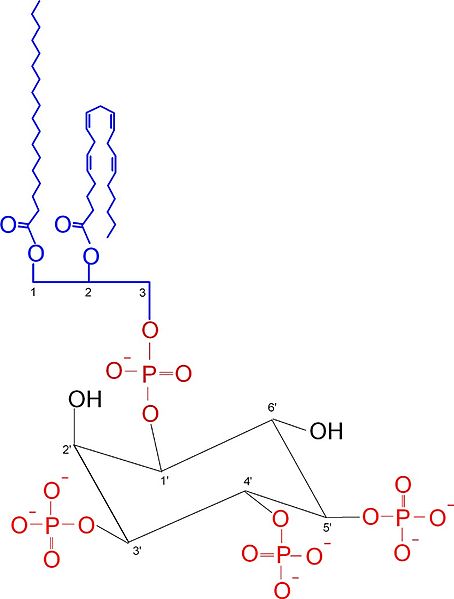

Supplement: Additional file 2 — ModelGuideWiki.zip. This archive file provides a copy of the files available online (https://modeling.tgen.org). These files serve to annotate the model. (ZIP 759 kb) [file 1752-0509-6-107-S2.zip › ModelGuideWiki/PIP3.jpg]
